# Chiral Pyrazolinylidene Complexes for Asymmetric Catalysis

**DOI:** 10.1002/chem.71426

**Published:** 2026-07-12

**Authors:** Felix Möller, Nemrud Demirel, Thomas Krüger, Linus Plohnke, Jennifer Schröder, Sergei I. Ivlev, Eric Meggers

**Affiliations:** ^1^ Fachbereich Chemie Philipps‐Universität Marburg Marburg Germany

**Keywords:** asymmetric catalysis, aziridination, *N*‐heterocyclic carbenes, nitrenoid, ruthenium

## Abstract

The strong σ‐donating properties of *N*‐heterocyclic carbenes (NHCs) have established them as key ligands in homogeneous catalysis. However, among this class of ligands, pyrazolinylidenes remain largely unexplored. Herein, we introduce the development of the first chiral pyrazolinylidene transition metal complex for asymmetric catalysis. Specifically, a chiral‐at‐ruthenium catalyst incorporating bidentate *N*‐(2‐pyridyl)‐substituted pyrazolin‐5‐ylidene ligands within a *C*_2_‐symmetric octahedral framework is reported. The modular ligand synthesis enables systematic variation at both the pyridine and carbene backbones, providing access to a series of air‐stable ruthenium(II) complexes. Spectroscopic analysis indicates enhanced σ‐donor strength compared to related imidazolin‐2‐ylidene and mesoionic carbene congeners, contributing to increased electron density at the metal center and a pronounced *trans*effect. The resulting complexes were evaluated in the enantioselective intramolecular aziridination of an alkene‐tethered *N*‐benzoyloxyurea, affording a bicyclic urea‐based aziridine containing a quaternary stereocenter in up to 95% yield and 98% enantiomeric excess. These findings reveal pyrazolin‐5‐ylidenes as powerful ligands for chiral transition metal catalysis and establish a robust catalyst scaffold to be applied to other asymmetric transformations.

## Graphical abstract

The synthesis and application of pyrazolin‐5‐ylidene NHC chiral‐at‐ruthenium catalysts is reported. The strong donating pyrazolin‐5‐ylidene ligands led to highly electron rich ruthenium complexes, which showed excellent catalytic activity and enantioselectivity in the intramolecular aziridination of an alkene.

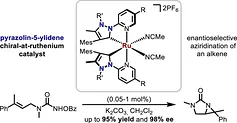

## 1 Introduction

The steric and electronic tunability of *N*‐heterocyclic carbenes (NHCs) has made them powerful tools for the development of homogeneous transition‐metal catalysts [1, 2]. While early work in the field of organometallic NHC complexes was mainly focused on the investigation of ligands that are stabilized by two adjacent heteroatoms such as imidazolin‐2‐ylidenes, NHC ligands with reduced heteroatom stabilization gained significant interest, since their different electronic properties were used to realize new catalytic applications [3, 4]. These less‐stabilized NHC ligands include classes like abnormal/mesoionic (aNHC/MIC), remote (rNHC) and other non‐classical NHC with only one heteroatom adjacent to the carbene center [5]. These classes of ligands also include strongly σ‐donating pyrazolin‐3‐ylidenes and pyrazolin‐5‐ylidenes, which both contain the same pyrazole‐based core structure, whereby their nomenclature depends on the corresponding substitution pattern on the nitrogen atoms [6, 7]. Since pioneering work by Öfele et al. [8], surprisingly these NHCs have not attracted much interest, especially concerning applications in transition metal catalysts, until Herrmann and coworkers reported the synthesis of a palladium pyrazolin‐3‐ylidene complex and its application in a Mizoroki–Heck reaction (Figure 1) [6]. Compared to the analogous imidazolin‐2‐ylidene complex, higher yields by a factor of 2 could be obtained [6]. Bernhammer and Huynh later introduced the synthesis and characterization of a series of pyrazolin‐5‐ylidene palladium complexes, including the first examples for hetero‐bis(NHC) complexes, which showed catalytic activity in the direct arylation of pentafluorobenzene [7].

**FIGURE 1:**
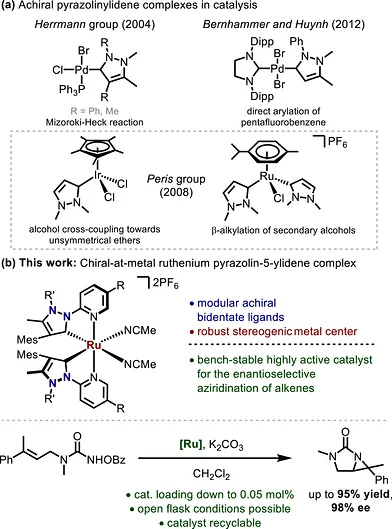
Pyrazolinylidene transition metal complexes in catalysis: (a) previous work and (b) this study.

Beyond these examples, catalytic applications of pyrazolinylidene half‐sandwich iridium and ruthenium complexes were reported by the group of Peris [9, 10]. In the investigated alcohol cross‐coupling towards nonsymmetric ethers or the β‐alkylation of secondary alcohols with primary alcohols, the pyrazolin‐3‐ylidene complexes provided satisfactory activities, comparable to other nNHC and aNHC metal catalysts. Overall, reports of the synthesis of complexes bearing pyrazolin‐3‐ or pyrazolin‐5‐ylidene NHCs and their catalytic applications are scarce and limited to achiral metal complexes [6, 7, 9, 10]. The development of chiral pyrazolinylidene transition‐metal complexes for asymmetric catalysis remains elusive. Due to their exceptionally strong σ‐donation, however, such complexes hold significant promise for providing unique electronic properties at the metal center and enabling unprecedented reactivity and selectivity in asymmetric transformations.

Herein, we report the first examples of chiral pyrazolinylidene transition metal complexes and their application in asymmetric catalysis. Specifically, pyrazolin‐5‐ylidene ligands are incorporated into an octahedral chiral‐at‐ruthenium scaffold, in which chirality arises from the stereogenic metal center [11, 12, 13]. The strong σ‐donating character of the pyrazolinylidenes enhances the electron density at the ruthenium center, leading to increased catalytic activity for the asymmetric aziridination of an alkene to afford an urea‐based bicyclic aziridine in yields of up to 95% and with excellent enantioselectivities of up to 98% ee. Despite the highly electron‐rich nature of the ruthenium center, the catalyst exhibits remarkable robustness, providing high performance even under open‐flask conditions and allowing recovery and reuse without any loss of reactivity or enantioselectivity.

## 2 Results and Discussion

### 2.1 Complex Design

Recently, we developed a series of chiral‐at‐ruthenium catalysts, where a ruthenium(II) center is coordinated by two bidentate *N*‐(2‐pyridyl)‐substituted *N*‐heterocyclic carbenes and two monodentate acetonitriles [14, 15]. In this *C*_2_‐symmetric structural framework, all ligands are achiral and the overall chirality arises from the helical arrangement of the two bidentate NHC ligands. The stereogenic metal center adopts either a Λ‐configuration (left‐handed helix) or Δ‐configuration (right‐handed helix). By including a strongly σ‐donating NHC and a π‐accepting pyridine, the bidentate ligands lead to a maximized ligand field stabilization energy and configurationally inertness of the metal center. At the same time the metal center is accessible for catalysis due to the labile monodentate acetonitriles, whose coordination is destabilized by the *trans*‐effect of the NHC‐moiety. Complemented by a mesityl group, contributing to additional stability due to interligand π–π‐stacking towards the pyridine groups, this design provides an efficient platform for the synthesis of active and robust ruthenium catalysts for asymmetric catalysis [14, 15]. In particular, it was shown that these chiral‐at‐ruthenium catalysts and their structural related ruthenium‐MIC complexes are valuable for stereocontrolled nitrene‐mediated C(sp^3^)–H functionalization reactions [16, 17, 18, 19, 20, 21, 22, 23, 24, 25, 26]. The structural benefits of this framework were therefore exploited to synthesize the first chiral pyrazolin‐5‐ylidene ruthenium catalysts. Notably, pyrazolin‐3‐ and pyrazolin‐5‐ylidenes have so far not been part of a bidentate ligand system, providing the opportunity to explore its influence on the metal center as part of a chelate ligand.

### 2.2 Ligand and Racemic Ruthenium Complex Synthesis

Our investigation commenced with the ligand synthesis, starting from commercially available pyrazolyl‐boronic ester **1**, which underwent a Suzuki cross‐coupling with mesityl iodide to afford pyrazole **2** in 74% yield (Scheme 1a). Nucleophilic aromatic substitutions (S_N_Ar) of pyrazole **2** with 2‐bromopyridine derivatives did surprisingly not afford the desired pyrazolium compounds. Instead, the reactions afforded pyrazoles **3a–c**, indicating a demethylation reaction under these harsh reaction conditions. While coupling of the pyrazole core with the non‐substituted 2‐bromopyridine and its trifluoromethyl derivative proceeded in satisfactory yields (67% and 74%), attempts to react 2‐bromo‐5‐(trimethylsilyl)pyridine with **2** failed. Since a sterically demanding group such as trimethylsilyl at the pyridine moiety seemed desirable for catalytic applications, an alternative route towards pyrazole **3d** was conducted. S_N_Ar of 2,5‐dibromopyridine gave pyrazole **3c** in moderate yields of 44%, which was then subjected to a lithiation and subsequent reaction with trimethylsilyl chloride to afford **3d** in 69% yield. The unexpected demethylation reaction then gave us the opportunity not only to methylate the pyrazole core but also to perform a phenylation. In both cases, by following modified procedures of our group for the methylation and phenylation of triazoles [18], the pyrazolium ligands **4a–d** could be obtained in yields of 27%–77% over two steps. Besides the reduced nucleophilicity, which is established for electron‐deficient pyrazoles [27], we assume that steric hindrance and the pyridine *N*‐functionalization as competing reaction are responsible for the only modest yields.

**SCHEME 1:**
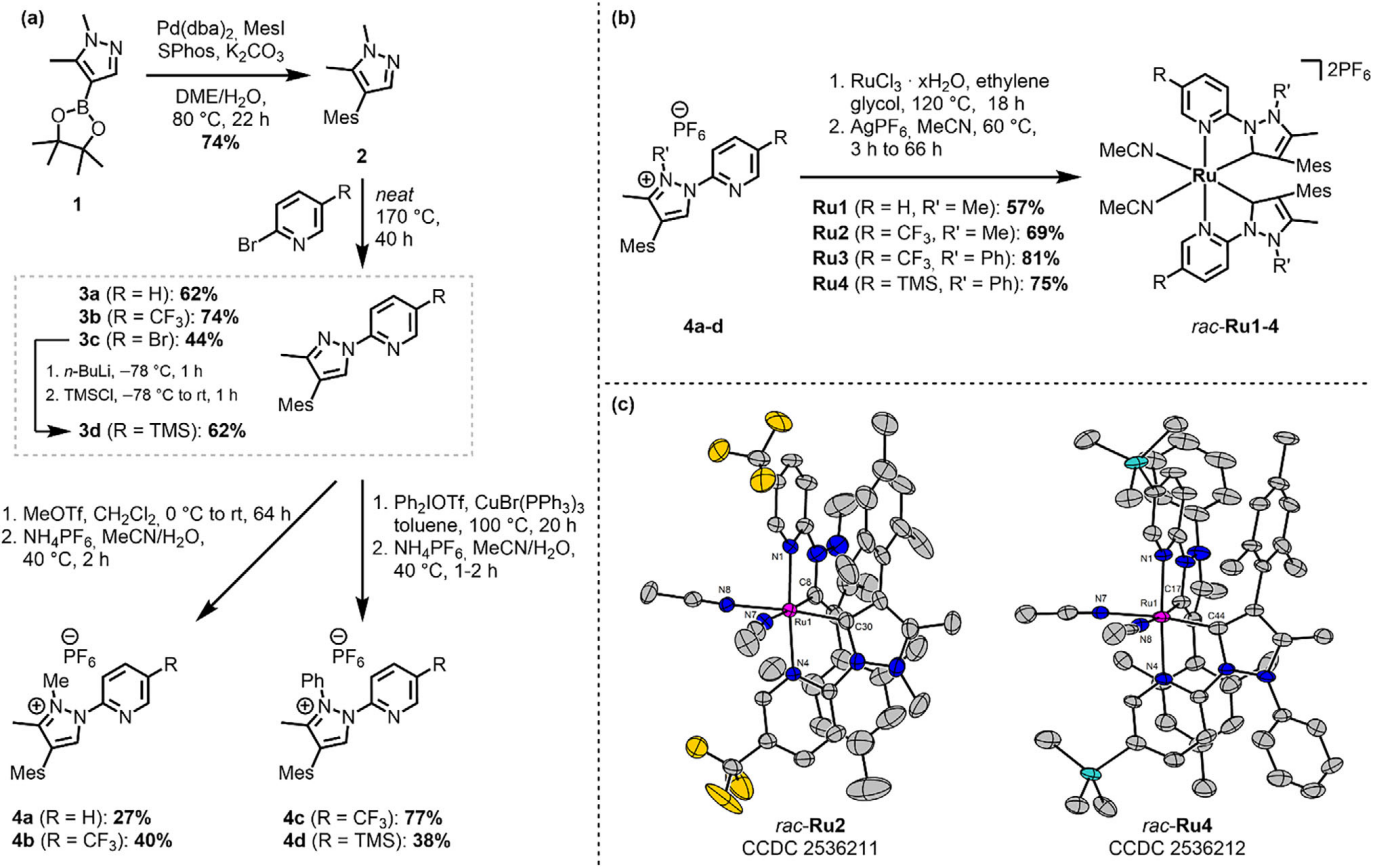
(a) Synthesis of the pyrazolium ligands **4a–d** and (b) pyrazolin‐5‐ylidene NHC ruthenium complexes *rac*‐**Ru1‐4**. (c) Crystal structure of *rac*‐**Ru2** and *rac*‐**Ru4**. The PF_6_^–^ anions, solvent molecules and hydrogen atoms are not shown. Displacement ellipsoids are shown at 50% probability level at 100 K.

The σ‐donor properties of the ligands were estimated by a determination of the ^1^J_CH_ coupling constants of the azolium carbon atom, since its correlation with the σ‐donor strength of the respective carbene has been reported for various NHC ligands [28, 29]. The determined ^1^J_CH_ coupling constants of **4a**–**d** are in the range of 195.2–199.2 Hz (see Supporting Information for more details) and show high agreement with values for the azolium salt of other monodentate pyrazolinylidene ligands [30, 31]. The differences of the ^1^J_CH_ coupling constants within the group of synthesized pyrazolium salts **4a–d** are very small, so that the influence of the pyrazol or pyridine substitution pattern cannot be rationalized unambiguously. However, a comparison with other NHC ligands from our group, which are based on the same *N*‐(2‐pyridyl)‐substituted *N*‐heterocyclic framework, indicates that the pyrazolin‐5‐ylidene NHC ligands **4a–d** are stronger σ‐donors than their structural related nNHC and MIC congeners [32].

For the synthesis of the pyrazolin‐5‐ylidene ruthenium complexes, the pyrazolium ligands **4a–d** were reacted with RuCl_3_ xH_2_O at 120°C in ethylene glycol overnight and subsequent treated with AgPF_6_ in acetonitrile at 60°C (Scheme 1b). As a result, *rac*‐**Ru1‐4** were successfully obtained. In contrast to previous work of our group on NHC and MIC ruthenium complexes, a reaction temperature of 120°C was necessary, since higher temperature led to diminished yields or even full decomposition [11, 12, 13, 18]. Overall, the complexes *rac*‐**Ru1‐4** proved to be air‐stable and showed good overall stability in coordinating and noncoordinating solvents at room temperature. When stored in acetonitrile at 4°C, no signs of decomposition could be observed by NMR spectroscopy after the time period of four weeks (see Supporting Information for details). The structures of *rac*‐**Ru2** and *rac*‐**Ru4** were confirmed by single crystal x‐ray diffraction analysis (Scheme 1c). The strong *trans*‐effect of the pyrazolin‐5‐ylidene NHCs and the resulting lability of the MeCN ligands is indicated by the elongated Ru─N bonds (*rac*‐**Ru2**: 2.129(5) and 2.132(5) Å; *rac*‐**Ru4**: 2.102(7) and 2.116(7) Å).

### 2.3 Non‐Racemic Complexes

Chiral resolution of the racemic complexes was performed with *rac*‐**Ru2** and *rac*‐**Ru3** by following a chiral‐auxiliary approach [33, 34]. Specifically, the chiral *N*‐benzoylsulfinamide (*S*)‐**Aux1** [35] proved to be superior compared to salicyloxazoline [34] or bis(oxazoline) [36] ligands and provided under basic conditions the diastereomerically pure complexes Δ‐(*R*)‐**RuAux2a** and Δ‐(*R*)‐**RuAux3a** in yields of 23% and 44%, respectively (Scheme 2). Note that upon coordination of (*S*)‐**Aux1** to the ruthenium, according to the Cahn–Ingold–Prelog rules, the assignment of the absolute configuration at the sulfur stereocenter changes from *S* to *R*. The corresponding Λ‐(*R*) diastereomers could not be isolated after column chromatographic purification, probably due to their thermodynamic instability. Instead, enantioenriched Λ‐**Ru2** and Λ‐**Ru3** were obtained in yields of 38% and 27%, respectively. However, the isolated complexes were significantly contaminated with their Δ‐isomer for Λ‐**Ru3** (67% ee) or obtained almost in a racemic form in the case of Λ‐**Ru2** (1% ee). A possible explanation for this could be the overall reduced stability of these auxiliary complexes, with the coordinating chiral auxiliary being very labile due to its *trans*‐position relative to the pyrazolin‐5‐ylidene NHCs. This lability rendered the use of basic aluminum oxide for column chromatographic purification necessary, although even with an optimized procedure, minor loss of the auxiliary with Δ‐(*R*)‐**RuAux2a** and Δ‐(*R*)‐**RuAux3a** could not be fully prevented. Another possible explanation, especially for the reaction of *rac*‐**Ru2** with (*S*)‐**Aux1**, which provided Δ‐(*R*)‐**RuAux2a** in a low yield (23%) and almost racemic isolated Λ‐**Ru2** (1% ee), could be an incomplete conversion or insufficient stability under the reaction conditions.

**SCHEME 2:**
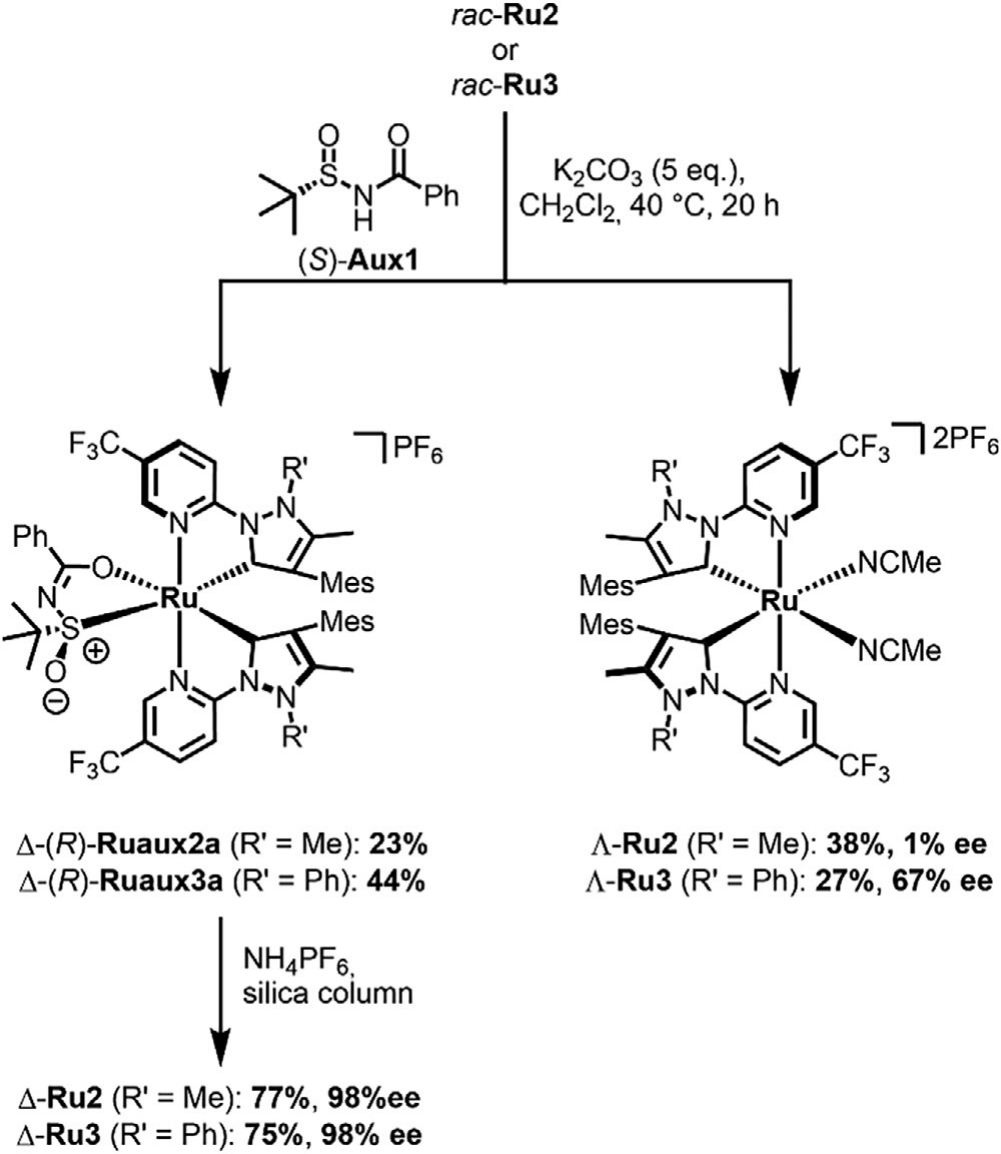
Chiral auxiliary‐mediated synthesis of the non‐racemic ruthenium complexes Δ‐**Ru2** and Δ‐**Ru3**.

With Δ‐(*R*)‐**RuAux2a** and Δ‐(*R*)‐**RuAux3a** in hand, subsequent cleavage of the chiral auxiliary was conducted by utilizing its high acid lability and performing column chromatography of the complexes on silica with a NH_4_PF_6_ plug to afford Δ‐**Ru2** and Δ‐**Ru3** in yields of 77% and 75%, respectively (Scheme 2). It was found that the diastereomeric purity of the used auxiliary complex and the rather mild conditions of this cleavage method were important for obtaining an enantiomeric excess of 98% ee for both complexes. In contrast, applying our previously reported auxiliary cleavage procedures [18] resulted in slightly diminished enantiomeric purities for Δ‐ and Λ‐**Ru2** (95%–97% ee). In analogy to this two‐step procedure, the corresponding Λ‐configured complexes can be obtained with high enantiomeric excess by employing (*R*)‐**Aux1** as demonstrated for Λ‐**Ru2** (see Supporting Information for details).

CD spectra of the mirror‐imaged complexes Λ‐ and Δ‐**Ru2** are displayed in Figure 2. The enantiopurity of all non‐racemic complexes was determined indirectly by using ^19^F NMR spectroscopy after coordination of the chiral auxiliary (*S*)‐**Aux2** (Figure 3a) [18]. As both complex enantiomers react to the thermodynamic stable auxiliary complexes Λ‐(*S*)‐**Ru** and Δ‐(*S*)‐**Ru** in the presence of NEt_3_ as base at 50°C in CD_2_Cl_2_, their ratio can be determined by integration of the signal of the fluorine group on the coordinating auxiliary of each diastereomer (see Supporting Information for details). Thus, direct conclusions on the ratio of enantiomers can be made. As shown for a sample of *rac*‐**Ru3**, next to excess free auxiliary ligand (*S*)‐**Aux2** (yellow dot), both diastereomers Λ‐(*S*)‐**RuAux3b** (red dot) and Δ‐(*S*)‐**RuAux3b** (blue dot), which were obtained in a 1:1 mixture, can be observed in the ^19^F NMR spectrum (Figure 3b). In the case of the enantioenriched samples of Λ‐**Ru3** and Δ‐**Ru3**, a diastereomeric excess of 67% and 98% was determined for the respective formed diastereomers. The structure for Δ‐(*S*)‐**RuAux3b**, resulting from Δ‐**Ru3**, could be confirmed from single crystal x‐ray diffraction analysis (Figure 3c), thereby also verifying the absolute configuration at the ruthenium center.

**FIGURE 2:**
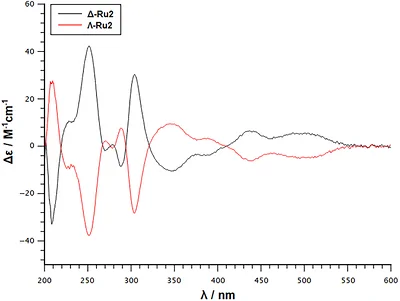
CD‐Spectra of Δ‐**Ru2** and Λ‐**Ru2** in MeCN (0.25 mM).

**FIGURE 3:**
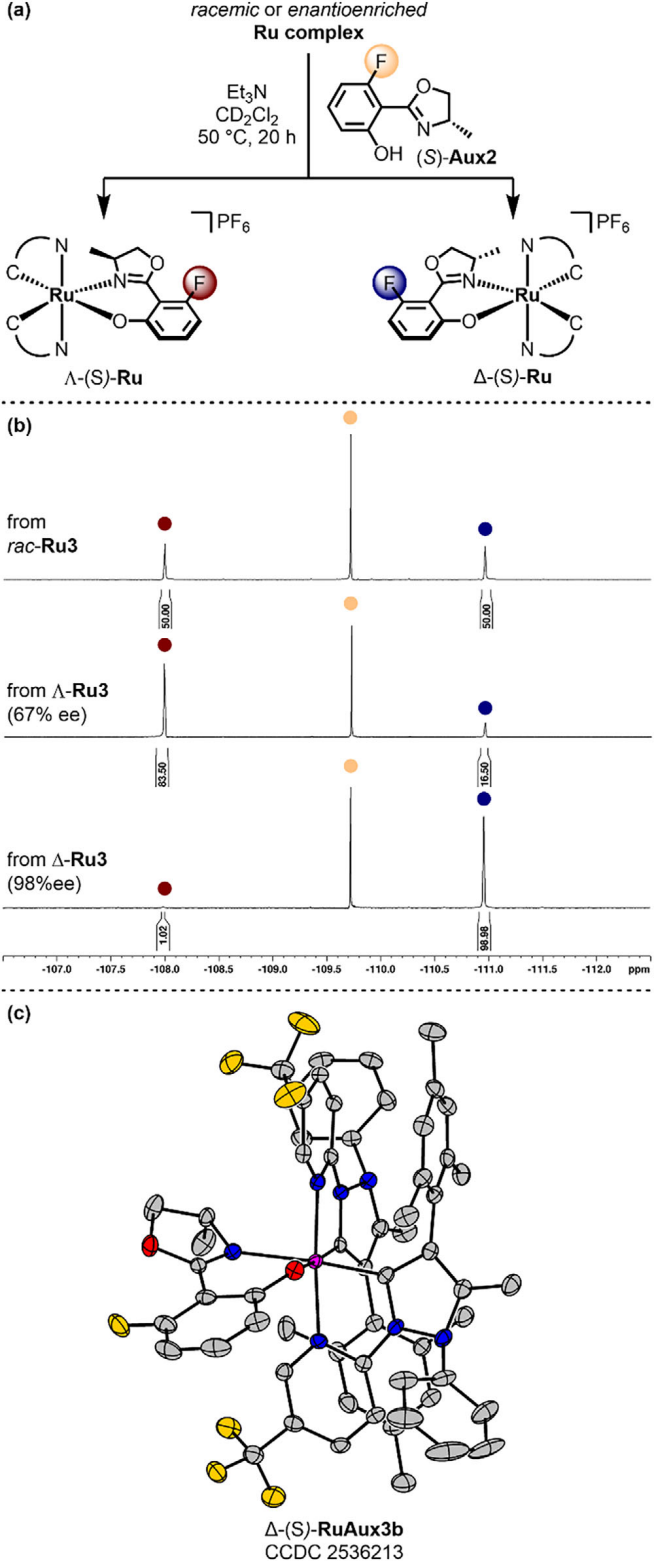
(a) Determination of the enantiomeric purity of the non‐racemic ruthenium complexes by reaction with the chiral ligand (*S*)‐**Aux2**, followed by measurement of a ^19^F NMR spectrum. (b) Excerpts of the ^19^F NMR spectra obtained from the reactions of *rac*‐**Ru3**, Λ‐ and Δ‐**Ru3** with (*S*)‐**Aux2**. (c) Crystal structure of Δ‐(*S*)‐**RuAux3b**. The PF_6_^−^ anion and hydrogen atoms are not shown. Displacement ellipsoids are shown at 50% probability level at 100 K.

### 2.4 Racemic and Enantioselective Catalysis

Imidazolidin‐2‐ones are important motifs that can be found in many natural products and biologically active drugs [37, 38, 39, 40, 41]. Furthermore, their use as chiral auxiliaries and the possibility to obtain chiral vicinal diamines as valuable building blocks in a single step makes them an interesting scaffold for the development of new catalytic methods [42, 43, 44]. Recently our group reported the enantioselective intramolecular C(sp^3^)–H amination of *N*‐benzoyloxyurea, providing chiral imidazolidin‐2‐ones by using a chiral‐at‐metal ruthenium catalyst [16]. For the mechanism of this reaction, the formation of a triplet ruthenium nitrenoid intermediate was proposed, which undergoes a hydrogen atom transfer followed by a radical recombination. As was shown recently by Berhal and Prestat for an iron complex, these formed intermediate metal nitrenoid species can also react with a double bond, affording imidazolidin‐2‐ones in an intramolecular amino‐oxygenation or their corresponding fused aziridine form [45, 46]. However, methods to obtain these cyclic imidazolidin‐2‐ones by functionalization of alkenes have only been reported in a diastereoselective manner [45, 46, 47].

We started our investigation of this reaction by using the *E*‐configured *N*‐benzoyloxyurea **5** as substrate and choosing dichloromethane as solvent and potassium carbonate as base (Table 1). After 3 h, *rac*‐**Ru1** as catalyst (1 mol%) showed full conversion and afforded the imidazolidin‐2‐one aziridine derivative **6** in an NMR yield of 90% as single diastereomer (entry 1). While remaining catalyst and salts are removed by a precipitation procedure, the aziridine **6** was obtained with high purity and side products could only be observed in traces in the ^1^H NMR spectrum. Notably, also the formation of the imidazolidin‐2‐one product, which would result from an azirine ring opening, was not detected [45, 46, 47]. By changing the catalyst to *rac*‐**Ru2**, which bears a CF_3_ group at the two pyridine moieties, a slightly better yield of 91% was afforded (entry 2). Direct comparison with *rac*‐**Ru3** revealed that the phenyl modification of the pyrazolin‐5‐ylidene NHC backbone is beneficial, leading to a yield of 95% for aziridine **6** (entry 3). Modification of the complex by an implementation of the sterically more demanding TMS at the pyridine moieties (*rac*‐**Ru4**) led to a significantly diminished yield of 76%, while still showing full conversion under standard conditions (entry 4). Overall, the complexes *rac*‐**Ru1‐4** provided superior results compared to the imidazolin‐2‐ylidene ruthenium complex *rac*‐**RuIm**, which led to a conversion of only 18% and a yield of 13% after 3 h (entry 5). Even though this recently reported class of nNHC Ru complexes demonstrated excellent catalytic performance in the C(sp^3^)–H amination of *N*‐benzoyloxyurea [16] or a structural related ring‐closing aminooxygenation [17], it provides inferior results for the here investigated aziridination in terms of catalytic activity and selectivity, as seen in the diminished conversion and yield. Since design and structural framework of *rac*‐**RuIm** are similar especially to the complexes *rac*‐**Ru2** and *rac*‐**Ru3**, we speculate that the enhancement of activity and selectivity can be attributed to the increased electron density at the ruthenium center as a result of the pyrazolin‐5‐ylidene NHC moieties.

**TABLE 1:** Racemic and enantioselective ruthenium catalyzed intramolecular aziridination of alkene **5**.^a^, ^b^

	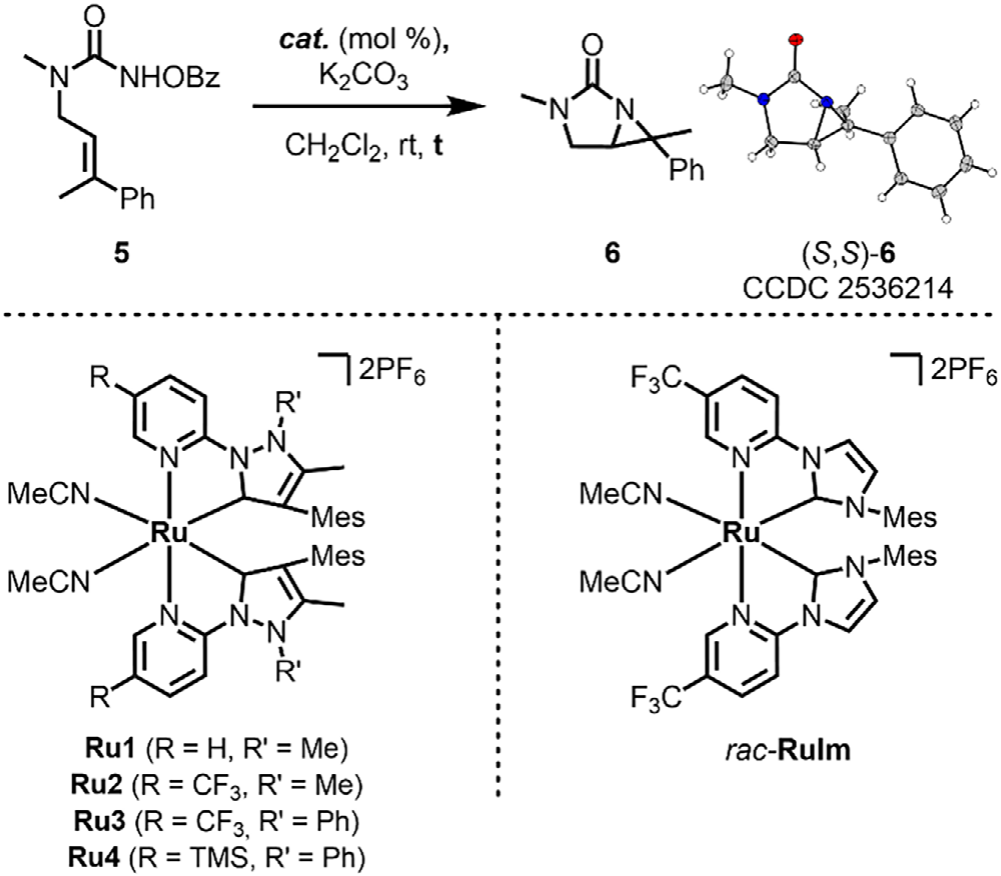				
Entry	Catalyst (mol%)	t (h)	Conversion (%)^c^	Yield (%)^c^	ee (%)^b^ ^,^ ^d^
1	*rac‐* **Ru1** [1.0]	3	>99	90	—
2	*rac‐* **Ru2** [1.0]	3	>99	91	—
3	*rac‐* **Ru3** [1.0]	3	>99	95	—
4	*rac‐* **Ru4** [1.0]	3	>99	57	—
5	*rac‐* **RuIm** [1.0]	3	18	13	—
6	Δ‐**Ru2** [1.0]	3	>99	91	98
7	Δ‐**Ru2** [0.5]	3	88	76	98
8	Δ‐**Ru2** [0.1]	68	98	71	98
9	Δ‐**Ru2** [0.05]	68	69	32	98
10	Δ‐**Ru3** [1.0]	3	>99	95	98
11^e^	Δ‐**Ru3** [1.0]	3	>99	94	98
12^f^	Δ‐**Ru3** [1.0]	16	>99	95	98
13	Δ‐**Ru3** [0.1]	68	>99	76	98
14^g^	Δ‐**Ru3** [0.1]	48	>99	76	98

^a^ Conditions: An oven‐dried Schlenk flask was charged with substrate **5** (0.10 mmol) and K_2_CO_3_ (0.30 mmol) under nitrogen atmosphere and catalyst (0.5–1.0 mol%) and dichloromethane (0.1 m) or a freshly prepared stock solution of the catalyst in dichloromethane (for entries with 0.1 and 0.05 mol%) was added. The resulting solution was stirred for the indicated time at the indicated temperature.

^b^ (*S*,*S*)‐**6** was obtained as major enantiomer. Its relative and absolute configuration was assigned by single crystal X‐ray diffraction (Displacement ellipsoids are shown at 50 % probability level at 100 K. The hydrogen atoms are shown with arbitrary radii).

^c^ Yield and conversion were determined via ^1^H NMR with 1,3,5‐trimethoxybenzene as internal standard.

^d^ Determined by HPLC analysis on a chiral stationary phase.

^e^ Reaction was performed under air in HPLC‐grade solvent.

^f^ Recycled catalyst was used (no additional purification conducted).

^g^ Reaction was performed at 40°C.

Chiral aziridines are highly versatile synthetic building blocks [48, 49]. We therefore continued the investigation of the enantioselective version of this aziridination with the complexes Δ‐**Ru2** and Δ‐**Ru3**, which were obtained from the chiral auxiliary mediated resolution with an enantiomeric excess of 98%. When Δ‐**Ru2** was used under the previously applied conditions, aziridine (*S*,*S*)‐**6** was obtained in 91% yield and 98% ee as a single diastereomer (Table 1, entry 6). The relative and absolute configuration of this product was assigned by single crystal x‐ray diffraction. To determine the influence of lower catalyst loading on yield and enantioselectivity, the reaction was conducted with 0.5 mol% of Δ‐**Ru2**, showing that a reaction time of 3 h is not sufficient to ensure full conversion, thus also leading to a reduced yield of 76% (Entry 7). While the overall conversion and selectivity seemed diminished, the enantioselectivity did not seem to be negatively impacted by the reduced catalyst loading. This finding was consistent with further experiments, using 0.1 and 0.05 mol% of Δ‐**Ru2** (entry 8 and 9). Despite an elongated reaction time of 68 h, almost full conversion could only be obtained with 0.1 mol% catalyst and in both experiments lower yields ([0.1 mol%]: 71%; [0.05 mol%]: 32%) were determined, while the enantiomeric excess of (*S*,*S*)‐**6** remained unchanged at 98%. In accordance with the findings of the racemic experiments, the use of Δ‐**Ru3** as catalyst afforded (*S*,*S*)‐**6** in 95% yield (entry 10). The catalyst also led to a determined enantiomeric excess of 98%, indicating that a change in the pyrazolin‐5‐ylidene substitution pattern (methylated backbone for Δ‐**Ru2** and phenylated backbone for Δ‐**Ru3**) does not affect the asymmetric induction negatively. To investigate the robustness of the catalyst system, the reaction was conducted with Δ‐**Ru3** under “open flask” conditions with HPLC grade solvent (entry 11). Surprisingly, only a minor decrease in the yield with 94% for (*S*,*S*)‐**6** was observed, whereas the enantioselectivity was not negatively impacted (98% ee). Since the catalyst is precipitated and removed by filtration before yield determination, it was attempted to reuse it in another experiment (entry 12). By adjusting the reaction time to 16 h, the recycled catalyst still afforded the product in a yield of 95% and enantiomeric excess of 98%. At a lower catalyst loading of 0.1 mol%, Δ‐**Ru3** provided (*S*,*S*)‐**6** in a yield of 76% (entry 13), which is in accordance with the trend for the comparison of Δ‐**Ru2** and Δ‐**Ru3**, obtained at 1 mol% catalyst loading (entry 6 and 8). Similarly, the enantiomeric excess of 98% ee was not affected by the catalyst loading, even when the reaction was conducted at 40°C to achieve full conversion already after 48 h (entry 14).

## 3 Conclusion

In conclusion, we have developed the first chiral pyrazolinylidene transition metal catalysts and demonstrated their efficacy in asymmetric catalysis. This was achieved by incorporating chelating pyrazolin‐5‐ylidene ligands [50] into a *C*_2_‐symmetric octahedral chiral‐at‐ruthenium scaffold. Spectroscopic data indicate enhanced σ‐donor strength relative to related imidazolin‐2‐ylidene and mesoionic carbene systems, resulting in increased electron density at the ruthenium center and a pronounced *trans*effect. These electronic features translate into superior catalytic performance in the enantioselective intramolecular aziridination of an alkene‐tethered *N*‐benzoyloxyurea, affording a bicyclic urea‐based chiral aziridine containing a quaternary stereocenter in up to 95% yield and 98% ee. An expanded substrate scope to evaluate scope and limitations of this enantioselective aziridination, including comparisons with related NHC and MIC catalysts, will be addressed in future work. Notably, the pyrazolinylidene catalysts display high robustness, operating efficiently under open‐flask conditions and allowing recovery and reuse without loss of activity or enantioselectivity. This study establishes pyrazolin‐5‐ylidenes as powerful ligands for chiral transition metal catalysis and opens new opportunities for the design of highly electron‐rich asymmetric catalysts. Future work in our laboratory will investigate the application of this new class of ruthenium complexes for other catalytic asymmetric transformations and the use of pyrazolin‐5‐ylidene NHC ligands for chiral catalysts of 3d metals.

## Conflicts of Interest

The authors declare no conflicts of interest.

## Supporting information



The authors have cited additional references within the Supporting Information [51, 52, 53, 54, 55, 56, 57, 58, 59, 60, 61, 62, 63, 64, 65, 66]. The SI includes detailed experimental procedures, analytical data, figures and tables (Figures S1‐S64, Tables S1‐S5), NMR spectra, CD spectra, UV/Vis spectra and HPLC traces. Deposition numbers 2536211 (for *rac*‐**Ru2**), 2536212 (for *rac*‐**Ru4**), 2536213 (for Δ‐(*S*)‐**RuAux3b**) and 2536213 (for (*S*,*S*)‐**6**) contain the supplementary crystallographic data for this paper. These data are provided free of charge by the joint Cambridge Crystallographic Data Centre and Fachinformationszentrum Karlsruhe Access Structures service.
**Supporting File 1**: chem71426‐sup‐0001‐SuppMat.pdf

**Supporting File 2**: chem71426‐sup‐0002‐DataFiles.zip

## Data Availability

The data that supports the findings of this study are available in the supplementary material of this article.
