# Supplementary material for: Chiral Pyrazolinylidene Complexes for Asymmetric Catalysis
Source: Chemistry. 2026 Jul 12;32(39):e71426. doi: 10.1002/chem.71426 (PMC13715465; doi:10.1002/chem.71426)
Supplement: Supplementary file 1 [file CHEM-32-e71426-s002.pdf]

Supporting Information

Chiral Pyrazolinylidene Complexes for Asymmetric Catalysis

Felix Möller, Nemrud Demirel, Thomas Krüger, Linus Plohnke, Jennifer Schröder, Sergei I. Ivlev and Eric Meggers

Fachbereich Chemie, Philipps-Universität Marburg,
Hans-Meerwein-Strasse 4, 35043 Marburg, Germany

*Email: meggers@chemie.uni-marburg.de

Table of Contents

1. General Information S2
2. Ligand Synthesis S3
3. Synthesis of Racemic Ruthenium Complexes S10
4. Auxiliary Complex Synthesis and Auxiliary Cleavage S13
5. Determination of the Enantiomeric Excess of the Ru Complexes by ^{19}F NMR S17
6. Investigation of the Electronic Properties of the Pyrazolium Ligands S20
7. Stability Investigations of *rac*-**Ru1** in CD_3CN S23
8. Substrate Synthesis S24
9. Ruthenium Catalyzed Enantioselective Aziridination S26
10. NMR Spectra S28
11. Chiral HPLC Traces S52
12. CD-Spectra S53
13. UV-Visible Spectra S55
14. Single Crystal X-Ray Diffraction S56
15. References S64

1. General Information

All reactions were conducted under a nitrogen atmosphere in oven-dried glassware unless noted otherwise. Solvents were distilled under a nitrogen atmosphere from calcium hydride (MeCN, CH_2Cl_2) or sodium/benzophenone (Et_2O, toluene) prior to use. Reagents that were purchased from commercial suppliers were used without further purification. Flash column chromatography was performed with silica gel 60 M from Macherey-Nagel (irregular shaped, 230−400 mesh, pH 6.8, pore volume: 0.81 mL g^{-1}, mean pore size: 66 Å, specific surface: 492 m^2 g^{-1}, particle size distribution: 0.5% < 25 μm and 1.7% > 71 μm, water content: 1.6%) or basic aluminum oxide from Honeywell/Fluka (Brockmann I, activated, ~150 mesh, pH 9.5, mean pore size: 58 Å, specific surface: 205 m^2 g^{-1}) or by automated column chromatography on a Büchi Pure C-810 Flash Chromatography system. ^{1}H NMR, ^{13}C {^{1}H} NMR and ^{19}F{^{1}H} NMR spectra were recorded on a Bruker AV NEO 300 MHz, AV II 300 MHz, AV III 300 MHz, AV III 500 MHz, or AV III HD 500 MHz spectrometer at ambient temperature. Chemical shift values *δ* are reported in ppm with the solvent resonance as internal standard. ^{19}F{^{1}H} NMR spectra were calibrated to trichlorofluoromethane ($CFCl_3$, δ = 0 ppm) as external standard. IR spectra were recorded on a Bruker Alpha FT−IR spectrometer. High-resolution mass spectrometry was performed on a Finnigan LTQ-FT Ultra mass spectrometer (Thermo Fischer Scientific) using ESI as ionization source. CD spectra were acquired with a JASCO J-810 CD spectropolarimeter (parameters: 600−200 nm, 1 nm bandwidth, 50 nm min^{-1} scanning speed, accumulation of 3 scans). UV/Vis absorbance spectra were recorded on a Thermo Fisher Scientific Multiscan GO spectrophotometer. Chiral HPLC was performed on a Shimadzu Prominence LC-2030C.

Compounds (*S*)- and (*R*)-**Aux1**,[1] (*S*)-**Aux2**,[2] **S1**[3] and Ph_2IOTf[4] were synthesized following literature procedures.

2. Ligand Synthesis

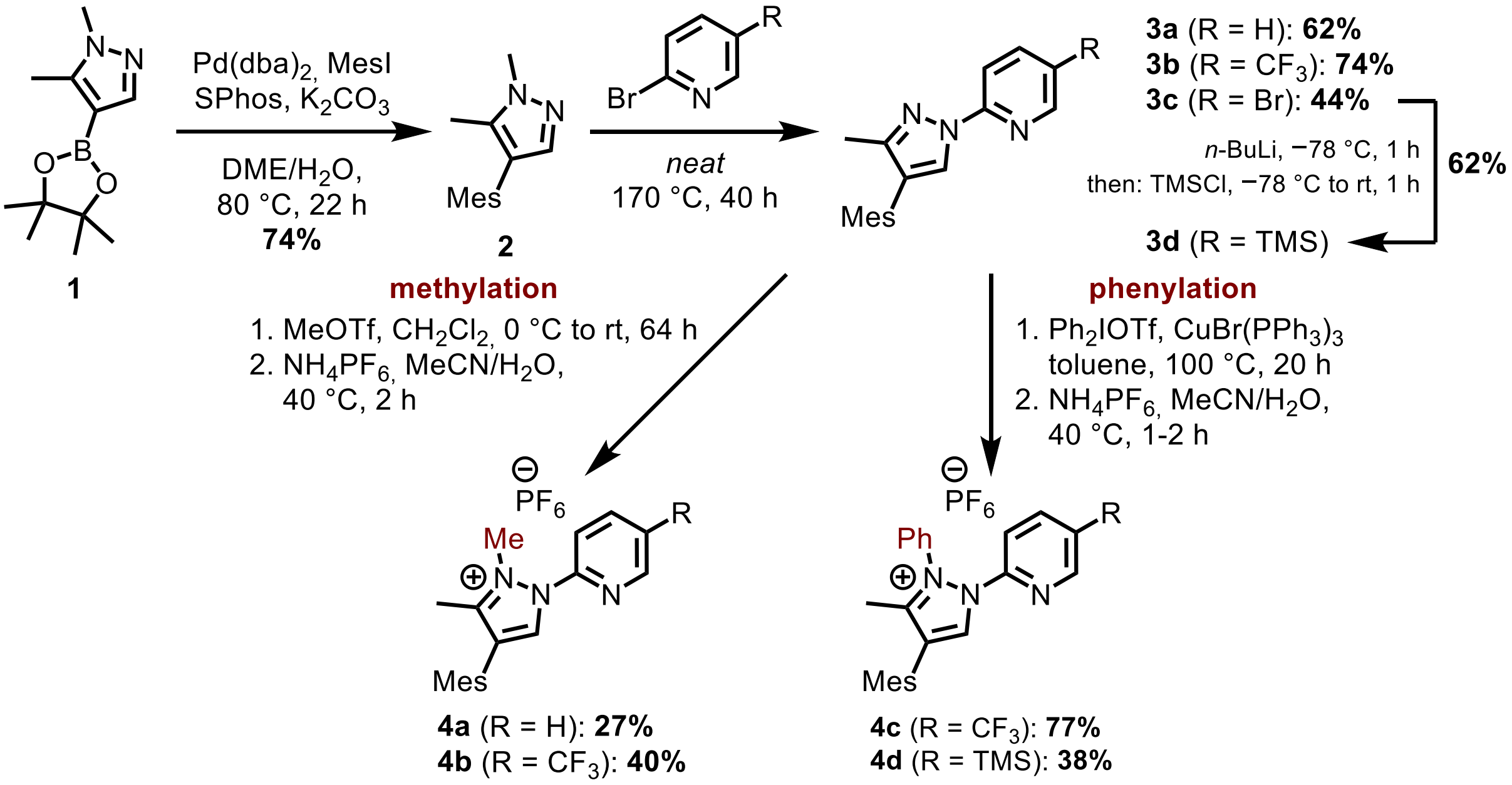

Scheme 1: Synthetic route to the pyrazolium ligands **4a-d**.

Synthesis of 4-mesityl-1,5-dimethyl-1H-pyrazole (2)

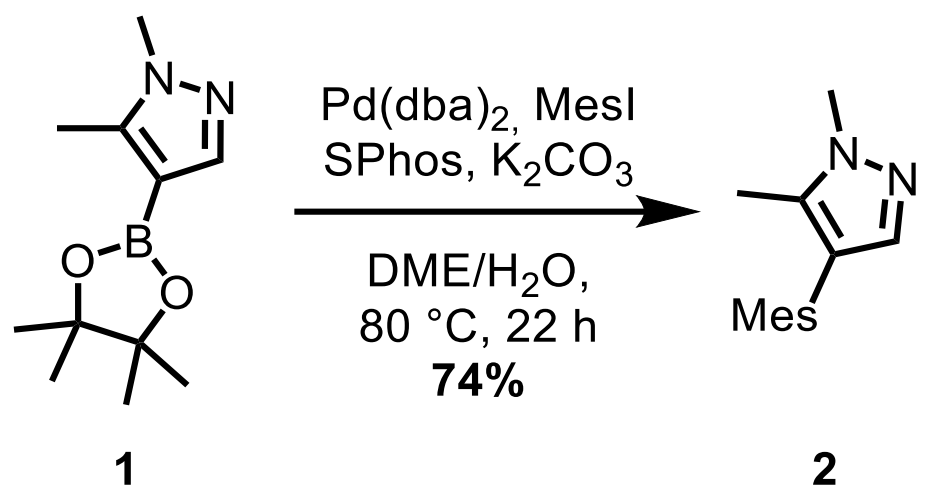

Pyrazole **1** (5.40 g, 24.3 mmol, 1.00 eq.), Mesityl iodide (7.48 g, 30.4 mmol, 1.25 eq.), K_2CO_3 (10.1 g, 72.9 mmol, 3.00 eq.), $Pd(dba)_2$ (454 mg, 0.790 mmol, 0.03 eq.) and SPhos (599 mg, 1.46 mmol, 0.06 eq.) were dissolved in a freshly degassed mixture of 1,2-dimethoxyethane (30.0 mL) and H_2O (30.0 mL) under an atmosphere of nitrogen. The resulting solution was stirred at 80 °C for 22 h. After cooling to room temperature, the mixture was extracted with ethyl acetate (4 × 30.0 mL) and the combined organic phases were dried over Na_2SO_4 and filtered. The solvent was removed under reduced pressure and the crude product was purified by column chromatography on silica (*n*-hexane/ethyl acetate 95:5 → 90:10 → 85:15 → 70:30 → 50:50) to obtain mesityl pyrazole **2** (3.86 g, 18.0 mmol, 74%) as yellow solid.

Synthetic note: A fast change of the solvent gradient towards a more polar mobile phase in column chromatography can result in contamination with the side product pinacol, which elutes directly after the desired product. If necessary, residues of pinacol can be removed by following a procedure of Aggarwal *et al.*.[5]

^{1}H NMR (300 MHz, $CDCl_3$, 300 K): δ = 7.25 (s, 1H), 6.93 (s, 2H), 3.86 (s, 3H), 2.32 (s, 3H), 2.03 (s, 6H), 2.03 (s, 3H) ppm. **^{13}C NMR** (75 MHz, CD_3CN, 300 K): δ = 138.2, 138.1, 136.8, 135.7, 128.1, 118.3, 36.6, 21.1, 20.9, 9.8 ppm. **IR** (neat): $\tilde{\nu}$ = 3411 (w), 3092 (w), 2943 (m), 2917 (m), 2857 (w), 2731 (w), 2198 (w), 2160 (w), 2135 (w), 1725 (w), 1613 (w), 1571 (w), 1485 (m), 1435 (m), 1391 (s), 1353 (w), 1292 (w), 1214 (w), 1178 (w), 1135 (w), 1112 (w), 1015 (m), 931 (s), 850 (s), 758 (w), 732 (w), 702 (w), 674 (w), 639 (w), 577 (m), 554 (w), 505 (w), 481 (w), 441 (w) cm^{-1}. **HRMS** (ESI+): *m/z* calc. for $C_{14}H_{19}N_2$ $[M + H]^+$: 215.1543, found: 215.1542.

Synthesis of the pyrazoles 3a-3c

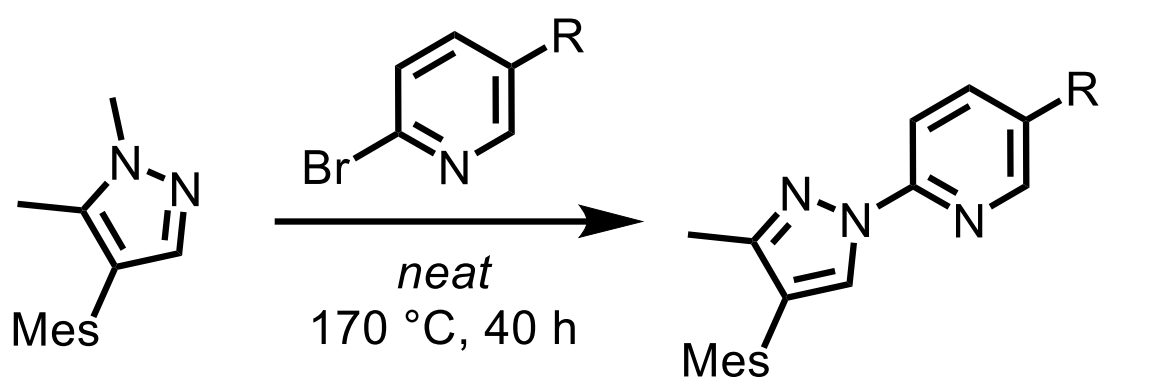

General procedure: Following a modified procedure of Zheng *et al.*,[6] a sealed flask was charged with mesityl pyrazole **2** (1.00 eq.) and the corresponding 2-bromopyridine (1.00 eq.) and the mixture was heated at 170 °C for 40 h. After cooling to room temperature, the dark brown solution solidified and was suspended in dichloromethane. Ethyl acetate was added and the suspension was filtered over celite. The solvent was removed under reduced pressure and the crude product was purified by column chromatography on silica (*n*-hexane/ethyl acetate 100:0 → 90:10 or *n*-pentane/ethyl acetate 100:0 → 90:10) to give the corresponding pyrazole.

2-(4-Mesityl-3-methyl-1H-pyrazol-1-yl)pyridine (3a)

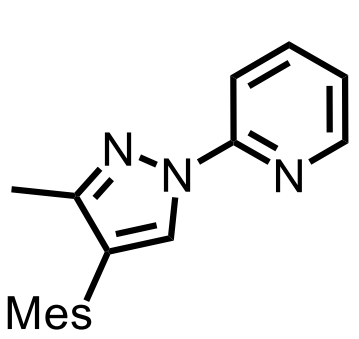

3a

By following the general procedure, pyrazole **3a** (1.30 g, 4.68 mmol, 62%) was obtained as colorless oil starting from 2-bromopyridine (1.19 g, 7.51 mmol, 1.00 eq.) and pyrazole **2** (1.61 g, 7.51 mmol, 1.00 eq.). **^{1}H NMR** (300 MHz, $CDCl_3$, 300 K): δ = 8.40 (dd, J = 2.1, 0.9 Hz, 1H), 8.39 (s, 1H), 8.02 (d, J = 8.3 Hz, 1H), 7.79 (ddd, J = 8.3, 7.3, 1.9 Hz, 1H), 7.12 (ddd, J = 7.3, 4.9, 1.1 Hz, 1H), 6.99 (s, 2H), 2.36 (s, 3H), 2.18 (s, 3H), 2.14 (s, 6H) ppm. **^{13}C NMR** (75 MHz, $CDCl_3$, 300 K): δ = 151.6, 150.6, 148.0, 138.4, 137.7, 137.0, 128.7, 128.0, 126.0, 121.5, 120.6, 111.8, 21.0, 20.7, 12.3 ppm. **IR** (neat): $\tilde{\nu}$ = 2948 (w), 2920 (w), 2856 (w), 2732 (w), 1726 (w), 1614 (w), 1594 (m), 1574 (m), 1494 (m), 1469 (s), 1451 (s), 1393 (m), 1376 (m), 1346 (s), 1241 (m), 1194 (m), 1159 (w), 1144 (w), 1096 (w), 1063 (m), 1033 (w), 1007 (m), 992 (m), 953 (s), 910 (w), 881 (w), 850 (m), 828 (w), 775 (s), 756 (w), 733 (m), 703 (w), 677 (m), 647 (w), 621 (w), 585 (m), 555 (w), 514 (w), 408 (w) cm^{-1}. **HRMS** (ESI+): *m/z* calc. for $C_{18}H_{19}N_3Na$ $[M + Na]^+$: 300.1471, found: 300.1466.

2-(4-Mesityl-3-methyl-1H-pyrazol-1-yl)-5-(trifluoromethyl)pyridine (3b)

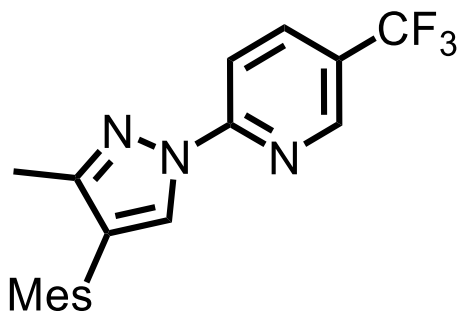

3b

By following the general procedure, pyrazole **3b** (699 mg, 2.02 mmol, 74%) was obtained as off-white solid starting from 2-Bromo-5-(trifluoromethyl)pyridine (617 mg, 2.73 mmol, 1.00 eq.) and pyrazole **2** (585 mg, 2.73 mmol, 1.00 eq.). ^{1}**H NMR** (300 MHz, $CDCl_3$, 300 K): δ = 8.66 – 8.64 (m, 1H), 8.35 (s, 1H), 8.09 (d, J = 8.7 Hz, 1H), 8.02 (dd, J = 8.8, 2.3 Hz, 1H), 6.97 (s, 2H), 2.34 (s, 3H), 2.14 (s, 3H), 2.09 (s, 6H) ppm. ^{13}**C NMR** (75 MHz, $CDCl_3$, 300 K): δ = 153.7, 152.3, 145.8 (q, J = 4.4 Hz), 137.8, 137.5, 135.9 (q, J = 3.3 Hz), 128.4, 128.3, 126.7, 123.8 (q, J = 271.6 Hz), 123.5 (q, J = 33.3 Hz), 122.8, 111.6, 21.2, 20.8, 12.5 ppm. ^{19}**F NMR** (282 MHz, $CDCl_3$, 300 K): δ = -61.88 (s, CF_3) ppm. **IR** (neat): $\tilde{\nu}$ = 2923 (w), 2859 (w), 1607 (m), 1576 (w), 1504 (m), 1487 (w), 1431 (m), 1380 (w), 1319 (s), 1247 (w), 1195 (w), 1161 (m), 1126 (s), 1082 (m), 1055 (m), 1012 (m), 947 (m), 910 (w), 845 (m), 804 (w), 762 (w), 733 (w), 705 (w), 686 (w), 640 (w), 593 (w), 521 (w), 472 (w), 426 (w) cm^{-1}. **HRMS** (ESI+): *m/z* calc. for $C_{19}H_{18}F_3N_3Na$ $[M + Na]^+$: 368.1339, found: 368.1345.

5-Bromo-2-(4-mesityl-3-methyl-1H-pyrazol-1-yl)pyridine (3c)

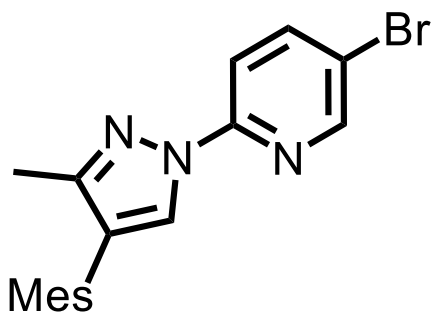

3c

By following the general procedure, pyrazole **3c** (781 mg, 2.19 mmol, 44%) was obtained as colorless solid starting from 2,5-dibromopyridine (1.18 g, 4.98 mmol, 1.00 eq.) and pyrazole **2** (1.07 g, 4.98 mmol, 1.00 eq.). ***Synthetic note*:** Residual 2,5-dibromopyridine, which contaminated pyrazole **3c** after column chromatography was removed by sublimation at 80 °C *in vacuo*. ^{1}**H NMR** (300 MHz, $CDCl_3$, 300 K): δ = 8.43 (dd, J = 1.9, 1.2 Hz, 1H), 8.31 (s, 1H), 7.91 – 7.87 (m, 2H), 6.98 (s, 2H), 2.36 (s, 3H), 2.15 (s, 3H), 2.12 (s, 6H) ppm. ^{13}**C NMR** (75 MHz, $CDCl_3$, 300 K): δ = 151.3, 150.4, 149.0, 141.1, 137.8, 137.3, 128.6, 128.2, 126.2, 122.1, 116.3, 113.3, 21.2, 20.8, 12.4 ppm. **IR** (neat): $\tilde{\nu}$ = 2948 (w), 2920 (w), 2856 (w), 2731 (w), 2656 (w), 2244 (w), 2046 (w), 1934 (w), 1657 (w), 1613 (w), 1571 (s), 1494 (m), 1461 (s), 1421 (s), 1376 (m), 1345 (s), 1248 (m), 1193 (m), 1159 (w), 1124 (w), 1091 (m), 1059 (m), 1032 (w), 1006 (m), 950 (s), 908 (m), 850 (m), 828 (s), 761 (w), 730 (s), 706 (m), 649 (w), 633 (w), 598 (w), 585 (m), 571 (w) cm^{-1}. **HRMS** (ESI+): *m/z* calc. for $C_{18}H_{18}BrN_3Na$ $[M + Na]^+$: 378.0582, found: 378.0564.

Synthesis of 2-(4-mesityl-3-methyl-1H-pyrazol-1-yl)-5-(trimethylsilyl)-pyridine (3d)

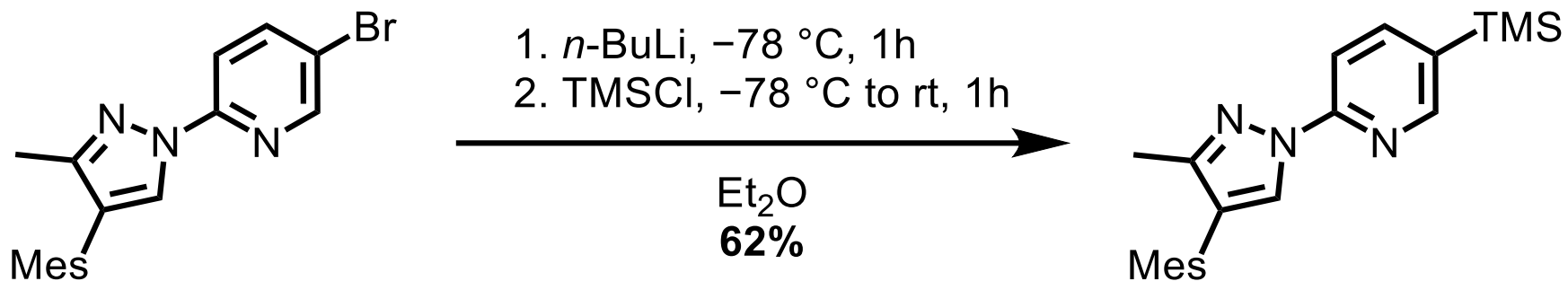

3c **3d**

Following a modified procedure of Stange *et al.*,[7] pyrazole **3c** (150 mg, 421 µmol, 1.00 eq.) was dissolved in diethyl ether (1.30 mL) under an atmosphere of nitrogen and the solution was cooled to −78 °C. Then, *n*-butyllithium (1.6 M, 236 µL, 421 µmol, 1.00 eq.) was added dropwise and the reaction mixture was stirred at −78 °C, before trimethylsilyl chloride (53.8 µL, 421 µmol, 1.00 eq.) was added. The solution was allowed to warm to room temperature within one hour and was then quenched with the addition of water (5.00 mL), The aqueous phase was extracted with diethylether (3 × 10.0 mL) and the combined organic phases were dried over Na_2SO_4 and filtered. The solvent was removed under reduced pressure and the crude product was purified by column chromatography on silica (*n*-pentane/dichloromethane 20:1 → 10:1 → 5:1 → 1:1) to obtain pyrazole **3d** (90.9 mg, 260 µmol, 62%) as yellow solid with small non-separable impurities.

^{1}H NMR (300 MHz, $CDCl_3$, 300 K): δ = 8.49 – 8.48 (m, 1H), 8.38 (s, 1H), 7.98 (d, J = 8.2 Hz, 1H), 7.91 (d, J = 7.8, 1.5 Hz, 1H), 6.98 (s, 2H), 2.35 (s, 3H), 2.16 (s, 3H), 2.12 (s, 6H), 0.34 (s, 9H) ppm. **^{13}C NMR** (75 MHz, $CDCl_3$, 300 K): δ = 152.5, 152.0, 150.8, 143.7, 137.8, 137.2, 131.7, 128.9, 128.1, 126.1, 121.6, 111.3, 21.2, 20.8, 12.5, -1.1 ppm. **IR** (neat): $\tilde{\nu}$ = 2953 (w), 2922 (w), 2859 (w), 1573 (s), 1495 (m), 1475 (m), 1445 (m), 1411 (m), 376 (w), 1345 (m), 1248 (m), 1197 (w), 1159 (w), 1131 (w), 1119 (m), 1092 (w), 1060 (w), 1019 (w), 1007 (w), 976 (w), 953 (m), 910 (w), 828 (s), 765 (m), 733 (m), 705 (w), 696 (w), 641 (w), 629 (w), 598 (w), 587 (w), 528 (w), 474 (w), 410 (w) cm^{-1}. **HRMS** (ESI+): *m/z* calc. for $C_{21}H_{28}N_3Si$ $[M + H]^+$: 350.2053, found: 350.2035.

Synthesis of the pyrazolium ligands 4a-4d

Methylation towards **4a** and **4b**:

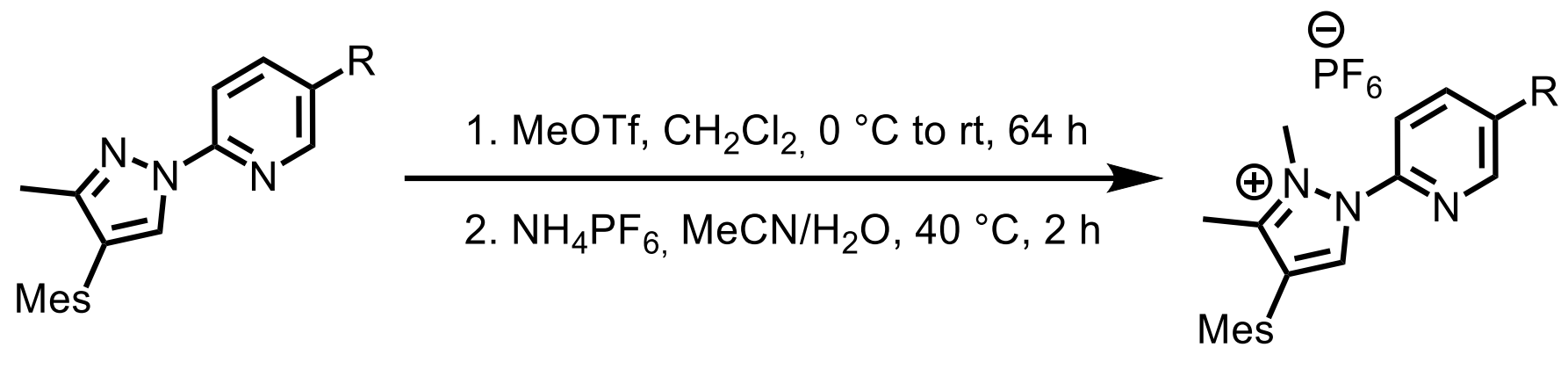

3a (R = H)
3b (R = CF_3)

4a (R = H): **27%**
4b (R = CF_3): **40%**

General procedure: Following a modified procedure of Demirel *et al.*,[8] the corresponding pyrazole (1.00 eq.) was dissolved in dichloromethane (0.4 M) under an atmosphere of nitrogen and the solution was cooled to 0 °C. Methyl triflate (1.20 eq.) was added and the solution was allowed to warm to room temperature over a time period of 16-64 h. Then NH_4PF_6 (10.0 eq.) and a mixture (1:1) of acetonitrile (0.4 M) and water (0.4 M) was added. The solution was stirred at 40 °C for 2 h. The aqueous phase was extracted with dichloromethane and then combined organic phases were dried over Na_2SO_4 and filtered. The solvent was removed under reduced pressure and the crude product was purified by column chromatography on silica (CH_2Cl_2/MeOH 100:0 → 98:2; or CH_2Cl_2/EtOAc 100:0 → 70:30) to give the corresponding pyrazolium ligand.

Synthetic note: Unreacted pyrazole can be reisolated during column chromatography or additional methyl triflate (0.4 eq.) may be added at room temperature for complete conversion. However, in the case of pyrazole **3a**, also the formation of side products resulting from an undesired pyridine methylation is increased.

4-Mesityl-2,3-dimethyl-1-(pyridin-2-yl)-1H-pyrazol-2-ium hexafluorophosphate (4a)

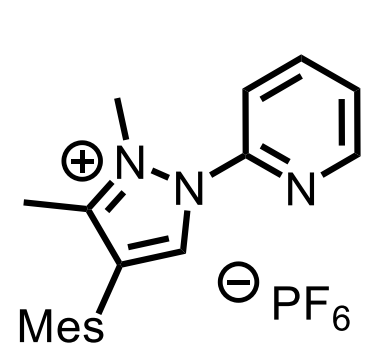

4a

By following the general procedure, pyrazolium **4a** (220 mg, 503 µmol, 27%) was obtained as off-white solid starting from pyrazole **3a** (523 mg, 1.88 mmol, 1.00 eq.). **^{1}H NMR** (300 MHz, $CDCl_3$, 300 K): δ = 8.69 (dd, J = 4.8, 1.6 Hz, 1H), 8.17 – 8.14 (m, 1H), 7.98 (d, J = 8.0 Hz, 1H), 7.94 (s, 1H), 7.65 (dd, J = 7.5, 4.8 Hz, 1H), 6.99 (s, 2H), 4.12 (s, 3H), 2.36 (s, 3H), 2.34 (s, 3H), 2.11 (s, 6H) ppm. **^{13}C NMR** (75 MHz, $CDCl_3$, 300 K): δ = 150.0, 148.9, 146.3, 141.1, 139.7, 138.1, 134.7, 128.9, 127.0, 123.3, 122.1, 122.1, 36.7, 21.2, 20.7, 10.8 ppm. **^{19}F NMR** (282 MHz, $CDCl_3$, 300 K): δ = -73.13 (d, J = 712.2 Hz, PF_6) ppm. **IR** (neat): $\tilde{\nu}$ = 3110 (w), 2922 (w), 2262 (w), 2177 (w), 1613 (w), 1591 (w), 1568 (w), 1472 (m), 1439 (s), 1409 (w), 1382 (m), 1347 (w), 1300 (w), 1248 (w), 1191 (m), 1134 (w), 1056 (m), 1036 (m), 996 (m), 875 (s), 781 (s), 728 (s), 666 (w), 647 (w),

631 (w), 604 (w), 547 (w), 521 (w), 481 (s), 448 (s), 412 (w) cm^{-1}. **HRMS** (ESI+): *m/z* calc. for $C_{19}H_{22}N_3$ [M - PF_6]$^+$: 292.1808, found: 292.1803.

4-Mesityl-2,3-dimethyl-1-(5-(trifluoromethyl)pyridin-2-yl)-1H-pyrazol-2-ium hexafluorophosphate (4b)

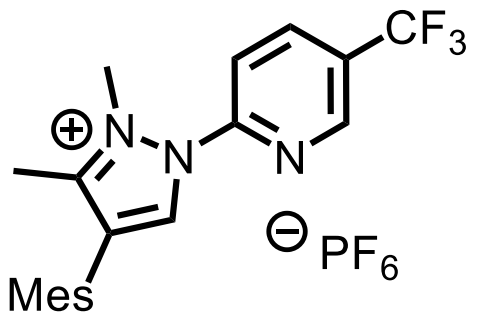

4b

By following the general procedure, pyrazolium **4b** (296 mg, 586 μmol, 40%) was obtained as off-white solid starting from pyrazole **3b** (500 mg, 1.45 mmol, 1.00 eq.) **^{1}H NMR** (500 MHz, CD_3CN, 300 K): δ = 9.06 (dd, *J* = 1.7, 0.8 Hz, 1H), 8.49 (dd, *J* = 8.4, 1.7 Hz, 1H), 8.31 (s, 1H), 7.93 (d, *J* = 8.5 Hz, 1H), 7.05 (s, 2H), 4.07 (s, 3H), 2.33 (s, 3H), 2.29 (s, 3H), 2.09 (s, 6H) ppm. **^{13}C NMR** (126 MHz, CD_3CN, 300 K): δ = 151.3, 145.0, 148.1 (q, *J* = 4.0 Hz), 140.4, 139.4 (q, *J* = 3.4 Hz), 139.0, 136.8, 129.5, 129.1 (q, *J* = 33.8 Hz), 124.7, 124.0 (q, *J* = 272.2 Hz), 122.6, 121.9, 38.0, 21.2, 20.7, 11.3 ppm. **^{19}F NMR** (282 MHz, CD_3CN, 300 K): δ = -63.03 (s, CF_3), -72.97 (d, *J* = 706.1 Hz, PF_6) ppm. **IR** (neat): $\tilde{\nu}$ = 3123 (w), 2925 (w), 2262 (w), 1676 (w), 1601 (w), 1487 (w), 1439 (w), 1401 (w), 1383 (m), 1327 (s), 1256 (w), 1171 (m), 1134 (s), 1083 (s), 1019 (m), 972 (w), 827 (s), 751 (w), 738 (w), 699 (w), 646 (w), 621 (w), 594 (w), 557 (s), 453 (w), 428 (w) cm^{-1}. **HRMS** (ESI+): *m/z* calc. for $C_{20}H_{21}F_3N_3$ [M - PF_6]$^+$: 360.1682, found: 360.1677.

Phenylation towards **4c** and **4d**:

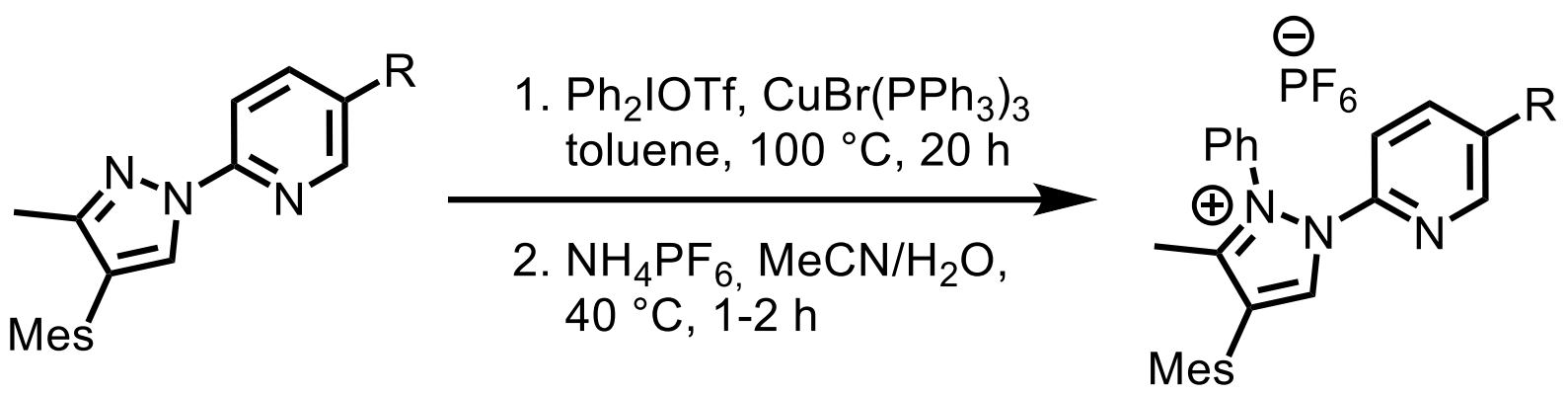

3b (R = CF_3)
3d (R = TMS)

4c (R = CF_3): **77%**
4d (R = TMS): **38%**

General procedure: Following a modified procedure of Demirel *et al.*,[8] the corresponding pyrazole (1.00 eq.), Ph_2IOTf (1.30 eq.) and $CuBr(PPh_3)_3$ (0.10 eq.) was dissolved in toluene (0.4 M) under an atmosphere of nitrogen and the solution was stirred at 100 °C for 20 h. After cooling to room temperature, the mixture was filtered over celite and rinsed with toluene or dichloromethane. The solvent was removed under reduced pressure and the residue was suspended in a mixture of acetonitrile/water (1:1, 0.1 M). NH_4PF_6 (10.0 eq.) was added and the solution was stirred at 40 °C for 1-2 h. The aqueous phase was extracted with dichloromethane and then the combined organic phases were dried over Na_2SO_4 and filtered. The solvent was removed under reduced pressure and the crude product was purified by column chromatography on silica (CH_2Cl_2/EtOAc 100:0 → 70:30) to give the corresponding pyrazolium ligand.

4-Mesityl-3-methyl-2-phenyl-1-(5-(trifluoromethyl)pyridin-2-yl)-1H-pyrazol-2-ium hexafluorophosphate (4c)

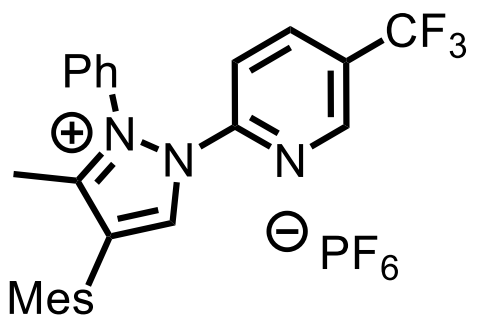

4c

By following the general procedure, pyrazolium **4c** (500 mg, 882 µmol, 77%) was obtained as off-white solid starting from pyrazole **3b** (393 mg, 1.14 mmol, 1.00 eq.). **^{1}H NMR** (300 MHz, $CDCl_3$, 300 K): δ = 8.73 – 8.66 (m, 1H), 8.37 (s, 1H), 8.08 (dd, *J* = 8.3, 2.1 Hz, 1H), 7.90 (d, *J* = 8.5 Hz, 1H), 7.66 – 7.57 (m, 5H), 7.03 (s, 2H), 2.36 (s, 3H), 2.25 (s, 3H), 2.22 (s, 6H) ppm. **^{13}C NMR** (75 MHz, $CDCl_3$, 300 K): δ = 151.6, 148.3, 146.5 (q, *J* = 4.1 Hz), 139.8, 138.1, 137.6 (q, *J* = 3.1 Hz), 137.1, 132.7, 131.9, 130.8, 128.9, 128.7 (q, *J* = 33.9 Hz), 128.2, 123.0, 122.7, 122.5 (q, *J* = 273.3 Hz), 121.3, 21.2, 20.6, 11.5 ppm. **^{19}F NMR** (282 MHz, $CDCl_3$, 300 K): δ = -62.52 (s, C*F*$_3$), -72.58 (d, *J* = 712.8 Hz, P*F*$_6$) ppm. **IR** (neat): $\tilde{\nu}$ = 3117 (w), 2925 (w), 2856 (w), 1683 (w), 1599 (w), 1482 (w), 1458 (w), 1438 (w), 1382 (w), 1327 (s), 1269 (w), 1247 (w), 1211 (w), 1172 (m), 1137 (m), 1076 (m), 1036 (w), 1019 (w), 972 (w), 939 (w), 825 (s), 776 (m), 732 (s), 696 (m), 649 (w), 624 (w), 607 (w), 555 (s), 528 (w), 472 (w), 448 (w), 431 (w) cm^{-1}. **HRMS** (ESI+): *m/z* $C_{25}H_{23}F_3N_3$ [M - PF_6]$^+$: 422.1839, found: 422.1836.

4-Mesityl-3-methyl-2-phenyl-1-(5-(trimethylsilyl)pyridin-2-yl)-1H-pyrazol-2-ium hexafluorophosphate (4d)

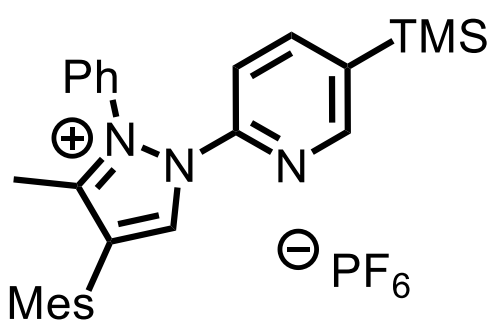

4d

By following the general procedure, pyrazolium **4d** (210 mg, 368 µmol, 38%) was obtained as off-white solid starting from pyrazole **3d** (342 mg, 978 µmol, 1.00 eq.). **^{1}H NMR** (300 MHz, $CDCl_3$, 300 K): δ = 8.47 (d, *J* = 1.0 Hz, 1H), 8.25 (s, 1H), 7.90 (dd, *J* = 8.0, 1.9 Hz, 1H), 7.63 – 7.52 (m, 6H), 7.00 (s, 2H), 2.34 (s, 3H), 2.21 (s, 9H), 0.28 (s, 9H) ppm. **^{13}C NMR** (75 MHz, $CDCl_3$, 300 K): δ = 153.5, 150.2, 146.4, 145.2, 139.7, 138.2, 136.5, 132.4, 132.1, 130.7, 128.9, 128.3, 123.1, 122.4, 120.6, 21.2, 20.7, 11.4, −1.5 ppm. **^{19}F NMR** (282 MHz, $CDCl_3$, 300 K): δ = -72.80 (d, *J* = 712.7 Hz, P*F*$_6$) ppm. **IR** (neat): $\tilde{\nu}$ = 3111 (w), 2960 (w), 2270 (w), 2022 (w), 1732 (w), 1613 (w), 1571 (w), 1490 (w), 1459 (w), 1438 (w), 1413 (w), 1383 (w), 1346 (w), 1293 (w), 1256 (w), 1211 (w), 1188 (w), 1125 (w), 1091 (w), 1026 (w), 1015 (w), 972 (w), 911 (m), 827 (s), 759 (w), 726 (s), 699 (m), 647 (m), 629 (w), 603 (w), 555 (s), 530 (w), 485 (w), 418 (w) cm^{-1}. **HRMS** (ESI+): *m/z* calc. for $C_{27}H_{32}N_3Si$ [M - PF_6]$^+$: 426.2366, found: 426.2351.

3. Synthesis of Racemic Ruthenium Complexes

General procedure for the complexation of the pyrazolium ligands 4a-4d

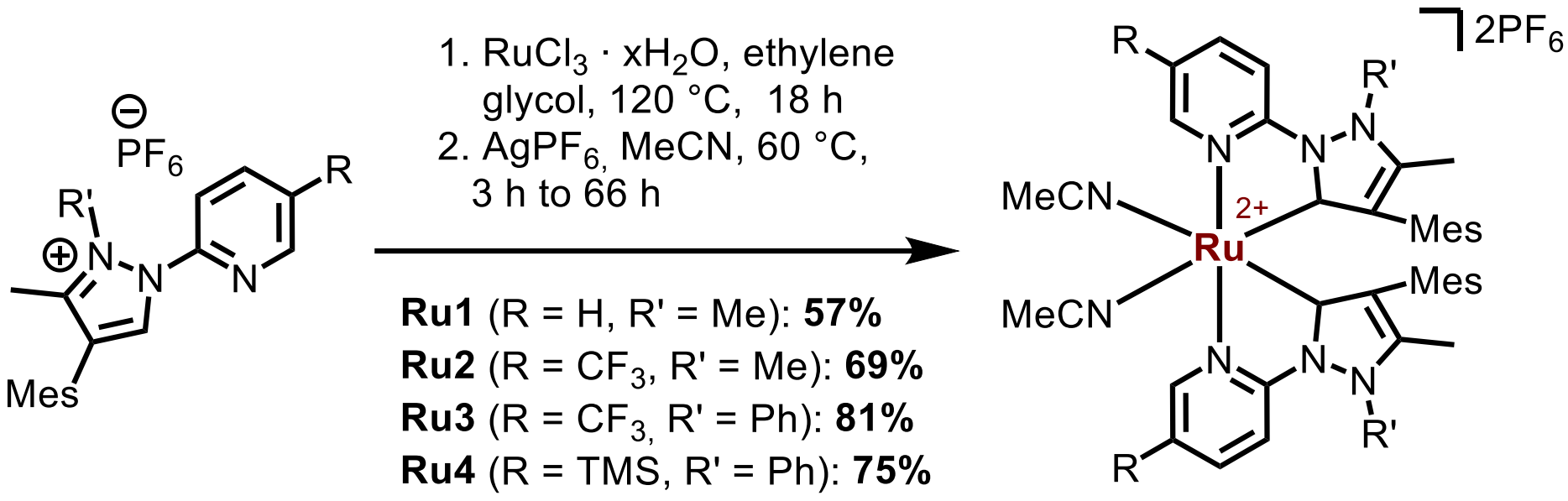

4a-d *rac*-**Ru1-4**

General procedure: In an oven-dried Schlenk tube, $RuCl_3 \cdot xH_2O$ (1.00 eq.) and ligand (2.05 eq.) were suspended in freshly degassed ethylene glycol (0.04 M) under an atmosphere of nitrogen. The reaction mixture was placed in a preheated metal block and was stirred at 120 °C for 20 h. After cooling to room temperature, saturated aqueous NH_4PF_6 solution (at least 10 ml/ 0.1 mmol [Ru]) was added. The red precipitate was filtered over celite and washed with H_2O (50 mL/0.1 mmol [Ru]) and dried on the celite plug, while the colorless supernatant and washing solution was discarded. Afterwards the precipitate was redissolved with dichloromethane and the red solution was dried over Na_2SO_4 and filtered. The solvent was removed under reduced pressure and the residue was dried in an oven-dried Schlenk tube. Then $AgPF_6$ (2.50 eq.) was added, the solids were dissolved in acetonitrile (0.04 M) and the resulting mixture was heated to 60 °C for 3 to 66 h under an atmosphere of nitrogen. After cooling to room temperature, the solvent was removed under reduced pressure and the residue was suspended in dichloromethane. The yellow-orange suspension was filtered over celite. The solvent was removed under reduced pressure and the crude product was purified by column chromatography silica (acetonitrile/dichloromethane 25:1 → 20:1 → 15:1 → 10:1 → 5:1 → 2:1) to give *rac*-**Ru1-4** as yellow to red solids.

Synthetic note: For the initial metalation a teflon-sealed schlenk tube proved to be advantageous compared to flask with silicon greased joints (Figure S1, A). Furthermore, it is important to ensure full precipitation of the complex with the saturated aqueous NH_4PF_6 solution after initial complexation, which results in a colorless to light yellow filtrate (B). If the filtrate is bright yellow/red, complex species may be still present and the aqueous phase has to be extracted with dichloromethane to avoid loss of product. After the first step the complexes are usually obtained as a dark red solution (C), which changes to yellow/orange during the second step as a consequence of the $AgPF_6$ treatment (D). Column chromatography with a slow gradient is necessary to afford the corresponding complex with sufficient purity (E), since small impurities elute directly in front and behind the desired complex (red arrows).

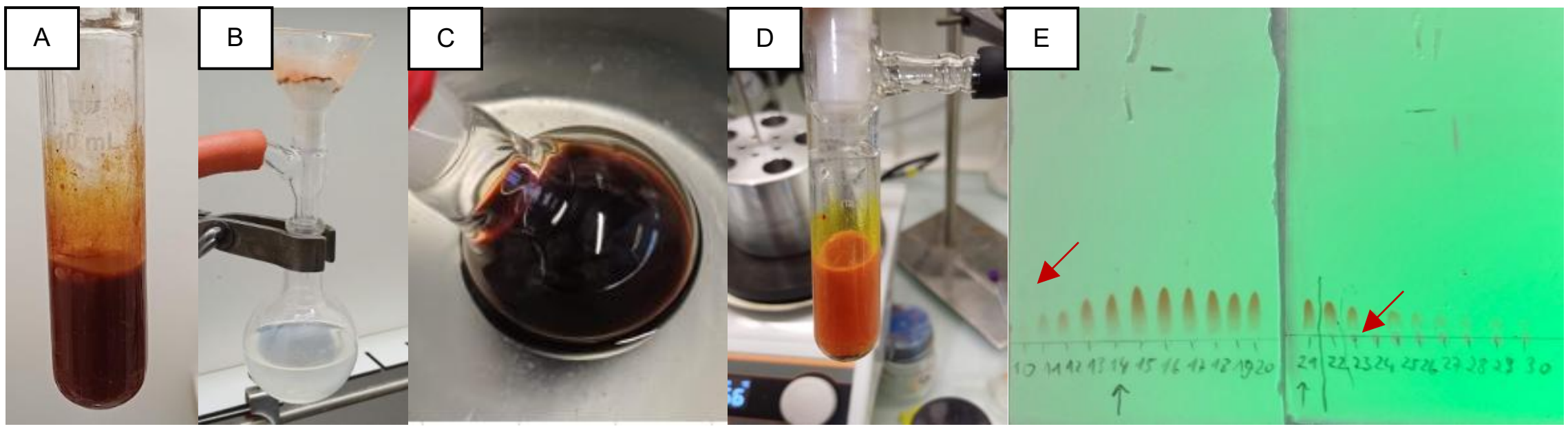

Figure S1: Pictures of crucial steps during the reaction.

***rac*-Ru1**

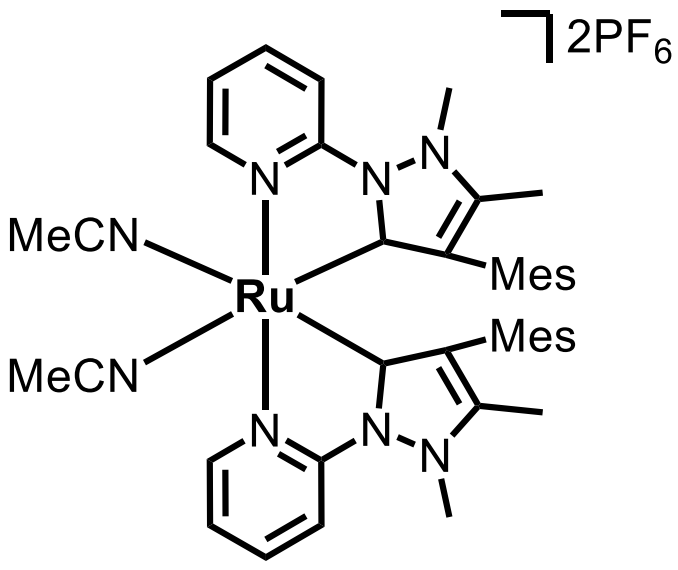

rac-**Ru1**

By following the general procedure, *rac*-**Ru1** (120 mg, 114 µmol, 57%) was obtained as yellow solid starting from $RuCl_3 \cdot xH_2O$ (41.6 mg, 201 µmol, 1.00 eq.) and pyrazolium **4a** (180 mg, 411 µmol, 2.05 eq.). **^{1}H NMR** (300 MHz, CD_3CN, 300 K): δ = 8.52 (ddd, J = 5.7, 1.7, 0.7 Hz, 2H), 7.73 (ddd, J = 9.0, 7.4, 1.7 Hz, 2H), 7.62 (dt, J = 8.4, 0.9 Hz, 2H), 6.93 (ddd, J = 7.1, 5.7, 1.2 Hz, 2H), 6.60 (s, 2H), 6.56 (s, 2H), 4.12 (s, 6H), 2.19 (s, 6H), 1.97 (s, 6H), 1.94 (s, 6H), 1.33 (s, 6H) ppm. **^{13}C NMR** (75 MHz, CD_3CN, 300 K): δ = 200.1, 153.2, 153.1, 151.0, 138.9, 138.5, 138.0, 137.6, 129.4, 129.3, 129.3, 128.8, 122.3, 111.5, 39.0, 21.1, 20.7, 20.5, 10.5 ppm. **^{19}F NMR** (282 MHz, CD_3CN, 300 K): δ = -72.61 (d, J = 707.4 Hz, PF_6) ppm. **IR** (neat): $\tilde{\nu}$ = 2922 (w), 2252 (w), 1606 (w), 1570 (w), 1543 (w), 1475 (m), 1461 (m), 1389 (w), 1370 (m), 1336 (w), 1319 (m), 1256 (w), 1213 (w), 1188 (w), 1161 (w), 1134 (w), 1111 (w), 1035 (w), 1012 (w), 831 (s), 795 (m), 761 (m), 739 (w), 663 (w), 637 (w), 621 (w), 557 (s), 519 (w), 451 (w) cm^{-1}. **HRMS** (ESI+): *m/z* calc. for $C_{42}H_{48}N_8Ru$ [M - $2PF_6$]$^{2+}$: 381.1523, found: 381.1517.

***rac*-Ru2**

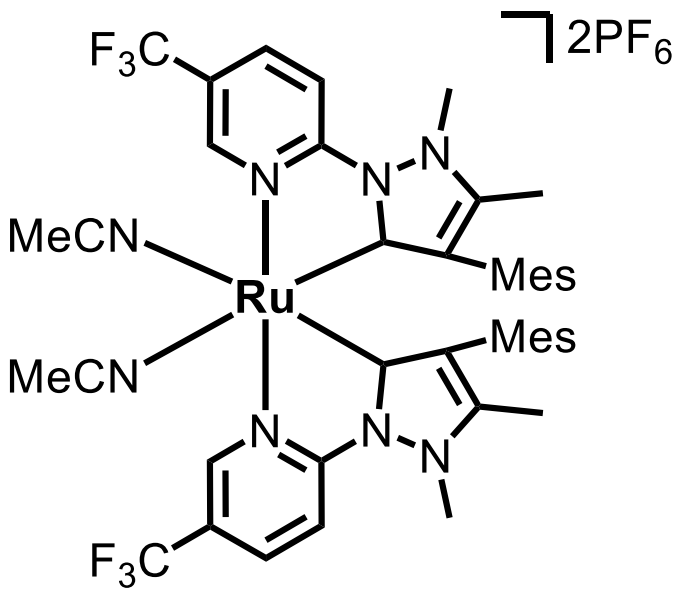

rac-**Ru2**

By following the general procedure, *rac*-**Ru2** (158 mg, 133 µmol, 69%) was obtained as red solid starting from $RuCl_3 \cdot xH_2O$ (40.0 mg, 193 µmol, 1.00 eq.) and pyrazolium **4b** (200 mg, 395 µmol, 2.05 eq.). **^{1}H NMR** (300 MHz, CD_3CN, 300 K): δ = 8.78 – 8.77 (m, 2H), 7.99 (dd, J = 9.0, 2.3 Hz, 2H), 7.81 (d, J = 9.1 Hz, 2H), 6.64 (s, 2H), 6.63 (s, 2H), 4.15 (s, 6H), 2.18 (s, 6H), 1.96 (s, 6H), 1.92 (s, 6H), 1.34 (s, 6H) ppm. **^{13}C NMR** (126 MHz, CD_3CN, 300 K): δ = 198.8, 155.5, 152.8, 149.8 (q, J = 4.1 Hz), 138.9, 138.8, 137.7, 135.6 (q, J = 3.4 Hz), 129.5, 129.4, 129.1, 128.8, 124.4 (q, J = 34.0 Hz), 123.6 (q, J = 271.3 Hz), 112.0, 39.3, 21.0, 20.6, 20.4, 10.6 ppm. **^{19}F NMR** (282 MHz, CD_3CN, 300 K): δ = -62.83 (s, CF_3), -72.61 (d, J = 707.4 Hz, PF_6) ppm. **IR** (neat): $\tilde{\nu}$ = 2917 (w), 2850 (w), 1614 (w), 1491 (w), 1415 (w), 1376

(w), 1329 (s), 1260 (w), 1137 (m), 1092 (m), 1019 (w), 838 (s), 738 (w), 680 (w), 558 (m) cm^{-1}. **HRMS** (ESI+): *m/z* calc. for $C_{44}H_{46}F_6N_8Ru$ $[M - 2PF_6]^{2+}$: 451.1297, found: 451.1389.

***rac*-Ru3**

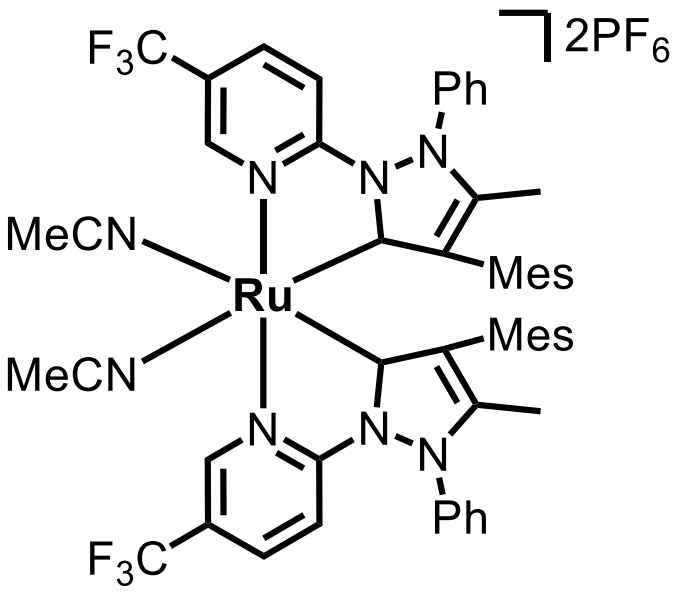

rac-**Ru3**

By following the general procedure, *rac*-**Ru3** (160 mg, 122 μmol, 81%) was obtained as red solid starting from $RuCl_3 \cdot xH_2O$ (31.2 mg, 150 μmol, 1.00 eq.) and pyrazolium **4c** (175 mg, 308 μmol, 2.05 eq.). **^{1}H NMR** (300 MHz, CD_3CN, 300 K): δ = 8.82 – 8.71 (m, 2H), 7.93 – 7.77 (m, 6H), 7.70 – 7.61 (m, 4H), 7.24 (d, *J* = 8.0 Hz, 2H), 6.66 (s, 2H), 6.64 (s, 2H), 6.37 (d, *J* = 9.0 Hz, 2H), 2.11 (s, 6H), 1.99 (s, 6H), 1.75 (s, 6H), 1.66 (s, 6H) ppm. **^{13}C NMR** (75 MHz, CD_3CN, 300 K): δ = 204.5, 155.2, 155.0, 149.7 (q, *J* = 4.6 Hz), 139.1, 138.4, 137.5, 136.6, 136.0 (q, *J* = 3.5 Hz), 133.1, 132.4, 132.2, 131.2, 129.7, 129.5, 128.4, 128.1, 127.8, 124.7 (q, *J* = 34.3 Hz), 123.5 (q, *J* = 271.6 Hz), 112.1, 20.9, 20.8, 20.6, 10.9 ppm. **^{19}F NMR** (282 MHz, CD_3CN, 300 K): δ = -62.72 (s, CF_3), -72.82 (d, *J* = 712.3 Hz, PF_6) ppm. **IR** (neat): $\tilde{\nu}$ = 2920 (w), 2851 (w), 2253 (w), 2181 (w), 2071 (w), 1976 (w), 1614 (w), 1543 (w), 1490 (w), 1415 (w), 1368 (w), 1327 (s), 1251 (w), 1175 (w), 1138 (w), 1082 (w), 1032 (w), 1007 (w), 923 (w), 841 (s), 785 (w), 738 (w), 700 (w), 626 (w), 558 (w), 514 (w), 484 (w) cm^{-1}. **HRMS** (ESI+): *m/z* calc. for $C_{54}H_{50}F_6N_8Ru$ $[M - 2PF_6]^{2+}$: 513.1555, found: 513.1547.

***rac*-Ru4**

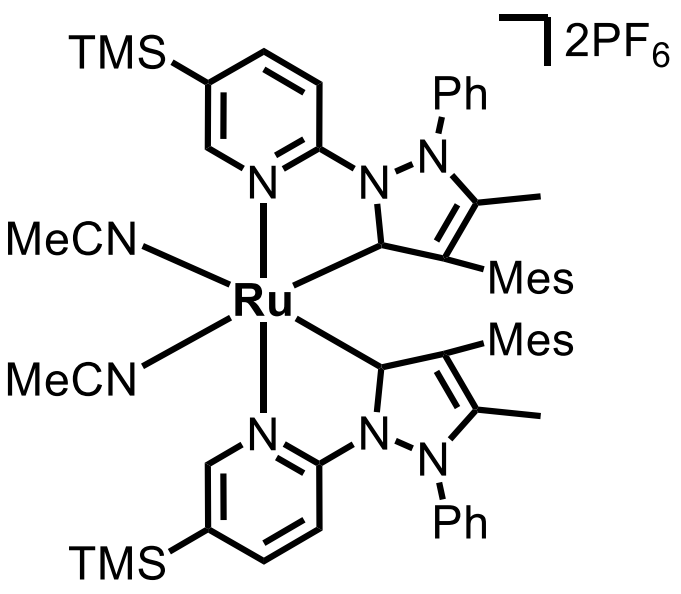

rac-**Ru4**

By following the general procedure, *rac*-**Ru4** (67.3 mg, 50.8 μmol, 75%) was obtained as orange solid starting from $RuCl_3 \cdot xH_2O$ (14.0 mg, 67.5 μmol, 1.00 eq.) and pyrazolium **4d** (79.1 mg, 138 μmol, 2.05 eq.). **^{1}H NMR** (300 MHz, CD_3CN, 300 K): δ = 8.61 (dd, *J* = 1.7, 0.8 Hz, 2H), 7.90 – 7.73 (m, 6H), 7.63 (t, *J* = 7.5 Hz, 2H), 7.42 (dd, *J* = 8.4, 1.7 Hz, 2H), 7.16 (dd, *J* = 7.9, 2.1 Hz, 2H), 6.67 (s, 2H), 6.54 (s, 2H), 6.10 (dd, *J* = 8.4, 0.8 Hz, 2H), 2.09 (s, 6H), 1.95 (s, 6H), 1.69 (s, 12H), 0.34 (s, 9H) ppm. **^{13}C NMR** (75 MHz, CD_3CN, 300 K): δ = 204.6, 155.7, 153.4, 153.1, 143.0, 138.4, 138.3, 137.4, 137.3, 134.2, 132.7, 132.2, 132.0, 131.0, 129.4, 129.1, 128.5, 128.4, 127.5, 111.1, 21.2, 21.0, 20.9, 10.7, -1.4 ppm. **^{19}F NMR** (282 MHz, CD_3CN, 300 K): δ = -72.88 (d, *J* = 706.4 Hz, PF_6) ppm. **IR** (neat): $\tilde{\nu}$ = 2955 (w), 2923 (w), 2196 (w), 2165 (w), 1613 (w), 1586 (w), 1482 (w), 1392 (w), 1373 (w), 1342 (w), 1320 (w), 1253 (w), 1200 (w), 1157 (w), 1096 (w), 1027 (w), 1007 (w), 967 (w), 838 (s), 776 (w), 761 (w), 735 (w), 705 (w), 608 (w), 558 (w), 541 (w), 429 (w), 408 (w) cm^{-1}. **HRMS** (ESI+): *m/z* calc. for $C_{58}H_{68}N_8Si_2Ru$ $[M - 2PF_6]^{2+}$: 517.2074, found: 517.2075.

4. Auxiliary Complex Synthesis and Auxiliary Cleavage

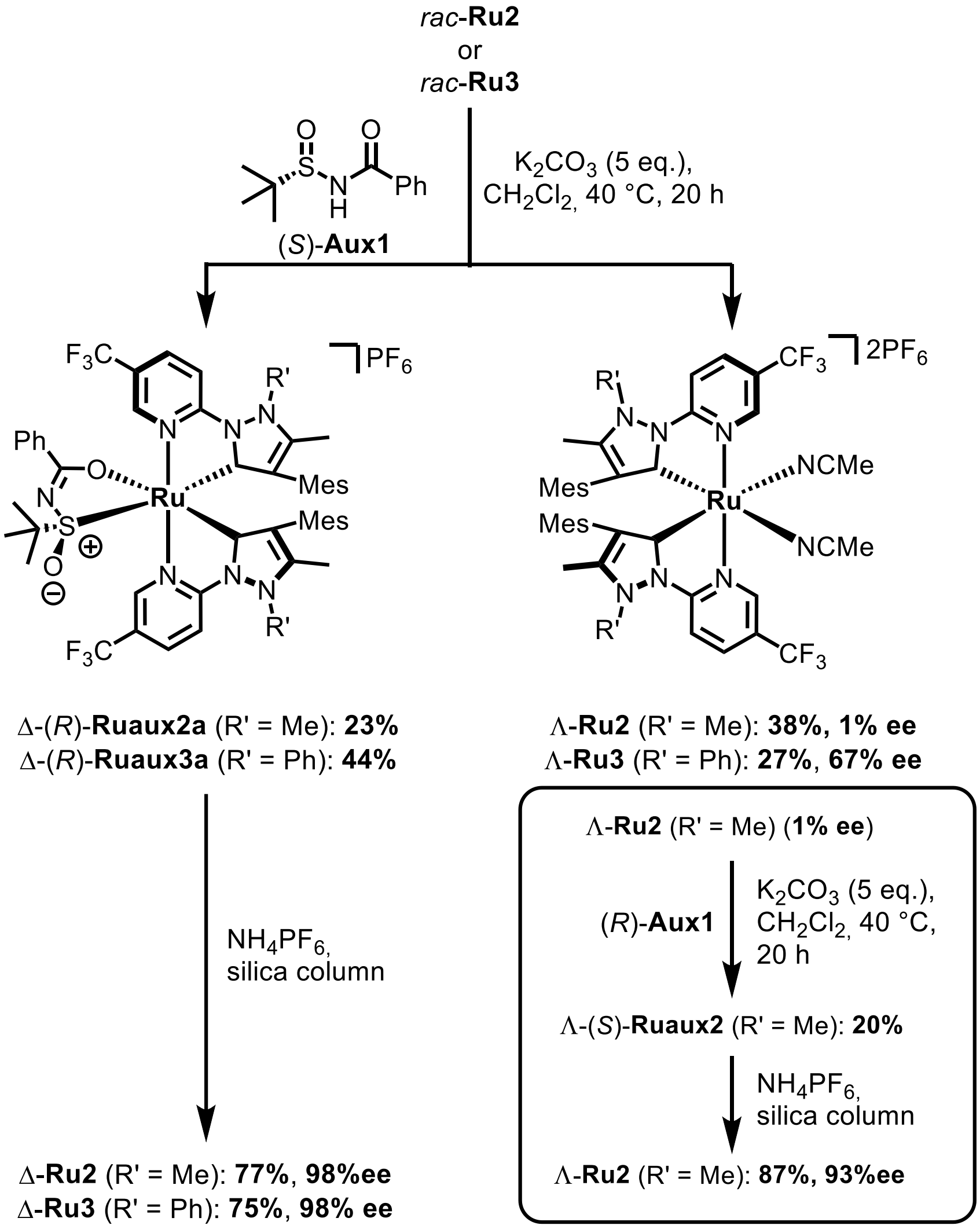

General procedure for the auxiliary complex synthesis:

The corresponding ruthenium complex (*rac*-**Ru2,** *rac*-**Ru3** or Λ-**Ru2** (1%ee); 1.00 eq.), the chiral sulfinamide auxiliary ((*S*)-**Aux1** or (*R*)-**Aux1**; 2.00 eq.) and K_2CO_3 (5.00 eq.) were suspended in dichloromethane (0.04 M) under nitrogen atmosphere in a pressure tube. The reaction mixture was stirred at 40 °C for 20 h and after cooling to room temperature directly purified by column chromatography on basic aluminum oxide (CH_2Cl_2/MeCN gradient). While the desired auxiliary complexes (F1) elute early at a gradient of 25:1 → 20:1 → 15:1 → 10:1, the enantioenriched acetonitrile complexes (F2) can be obtained by further change to a more polar gradient 5:1 → 3:1 → 1:1. Additionally, after removing the solvent under reduced pressure, the residues containing the enantioenriched acetonitrile complexes were taken, suspended in a mixture of dichloromethane/acetonitrile (20:1) and filtered over celite, to remove traces of salt which elute at a polar gradient.

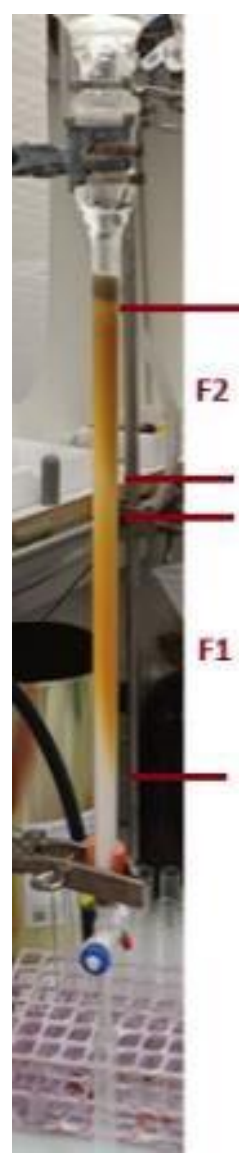

Synthetic note: A slow gradient during column chromatography is necessary to obtain the desired auxiliary complex in good diastereomeric purity and avoid contamination of small amounts of the other (unstable) diastereomer, which could hamper the enantiomeric excess of the acetonitrile complex after auxiliary cleavage. The overall increased lability of the coordinating auxiliary may also lead to partial decomposition, when the auxiliary complex is stored in non-coordinating solvent for a prolonged period of time.

Δ-(*R*)-Ruaux2a

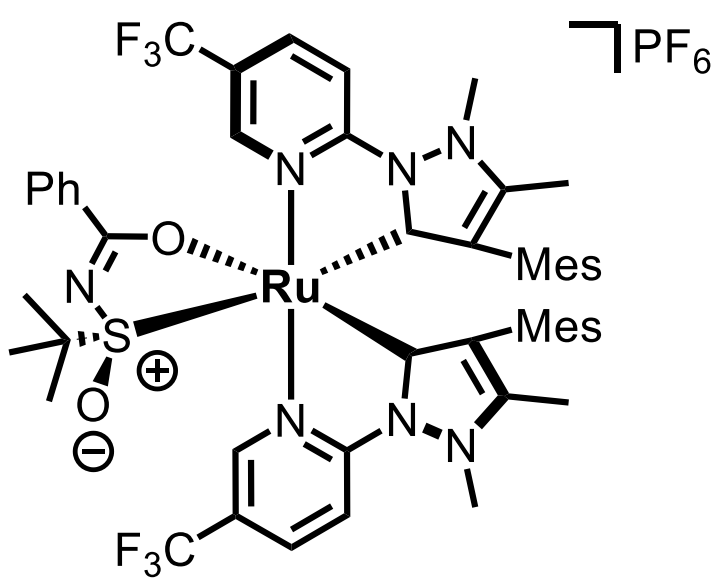

Δ-(*R*)-**Ruaux2a**

By following the general procedure, Δ-(*R*)-**Ruaux2a** (6.7 mg, 5.63 µmol, 23%) and Λ-**Ru2** (11.0 mg, 9.23 µmol, 38%, 1% ee) were obtained as orange solids starting from *rac*-**Ru2** (28.8 mg, 24.2 µmol, 1.00 eq.). Spectroscopic data of Λ-**Ru2** were consistent with *rac*-**Ru2**.

Analytical data for Δ-(*R*)-**Ruaux2a**: **^{1}H NMR** (300 MHz, CD_3CN, 300 K): δ = 10.91 (s, 1H), 8.21 (s, 1H), 8.12 – 8.08 (m, 2H), 7.90 – 7.85 (m, 2H), 7.79 – 7.75 (m, 2H), 7.51 – 7.45 (m, 1H), 7.39 (dd, *J* = 8.2, 6.6 Hz, 2H), 6.70 (s, 1H), 6.62 (s, 2H), 6.59 (s, 1H), 4.19 (s, 3H), 4.10 (s, 3H), 2.17 (s, 3H), 2.16 (s, 3H), 2.11 (s, 6H), 1.98 (s, 3H), 1.89 (s, 3H), 1.36 (s, 6H), 0.69 (s, 9H) ppm. **^{13}C NMR** (126 MHz, CD_3CN, 300 K): δ = 206.3, 195.6, 180.4, 155.9, 155.5, 155.1, 153.5 (q, *J* = 4.5 Hz), 153.3, 148.7 (q, *J* = 4.8 Hz), 139.4, 138.7, 138.7, 138.6, 137.5, 137.4, 135.6 (q, *J* = 3.5 Hz), 135.6, 135.2 (q, *J* = 3.3 Hz), 132.3, 130.8, 130.2, 129.8, 129.7, 129.5, 129.1, 129.1, 129.0, 128.9, 124.0 (*J* = 271.5 Hz), 123.5 (*J* = 34.0 Hz), 123.4 (q, *J* = 34.0 Hz), 123.2 (q, *J* = 271.5 Hz), 112.6, 111.6, 66.6, 61.0, 39.9, 39.5, 23.7, 21.2, 21.0, 20.9, 20.9, 20.4, 20.3, 10.8, 10.4 ppm. **^{19}F NMR** (282 MHz, CD_3CN, 300 K): δ = -62.73 (s, CF_3), -63.59 (s, CF_3), -72.92 (d, *J* = 706.2 Hz, PF_6) ppm. **IR** (neat): $\tilde{\nu}$ = 2923 (w), 2857 (w), 1682 (w), 1614 (w), 1540 (w), 1482 (m), 1436 (w), 1415 (w), 1366 (m), 1326 (s), 1246 (w), 1208 (w), 1178 (m), 1134 (m), 1091 (m), 1030 (w), 1010 (w), 970 (w), 939 (w), 839 (s), 785 (m), 740 (w), 715 (w), 679 (w), 637 (w), 621 (w), 590 (w), 557 (m), 508 (w), 476 (w), 435 (w) cm^{-1}. **HRMS** (ESI+): *m/z* calc. for $C_{51}H_{54}F_6N_7O_2SRu$ [M - PF_6]$^+$: 1044.3007, found: 1044.2978. **CD** (MeCN): λ, nm (Δε, M^{-1} cm^{-1}) 215 (18), 256 (−57), 287 (9), 306 (−47), 360 (15), 476 (−10).

Λ-(*S*)-Ruaux2a

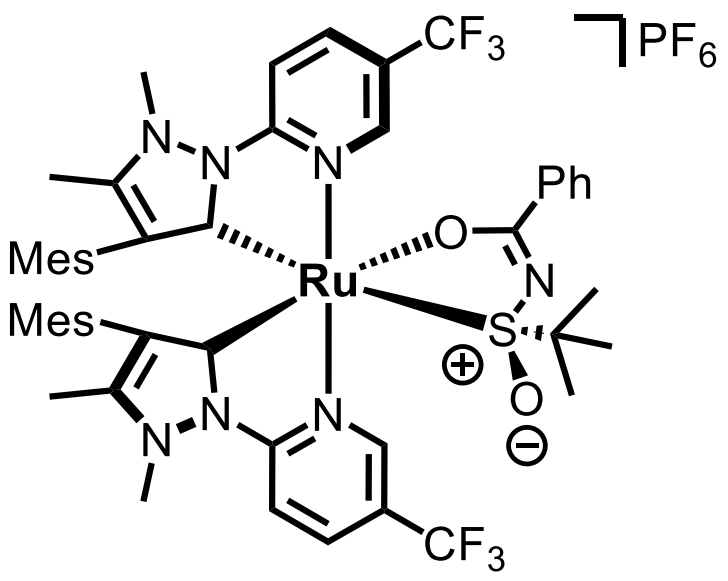

Λ-(*S*)-**Ruaux2a**

By following the general procedure, Λ-(*S*)-**Ruaux2a** (7.6 mg, 3.26 µmol, 20%) was obtained as orange solid starting from enantioenriched Λ-**Ru2** (1% ee, 38.8 mg, 32.6 µmol, 1.00 eq.). Spectroscopic data of Λ-(*S*)-**Ruaux2a** were consistent with its enantiomer Δ-(*R*)-**Ruaux2a**. **CD** (MeCN): λ, nm (Δε, M^{-1} cm^{-1}) 215 (−27), 256 (88), 287 (−14), 306 (73), 360 (−22), 476 (15).

Δ-(*R*)-Ruaux3a

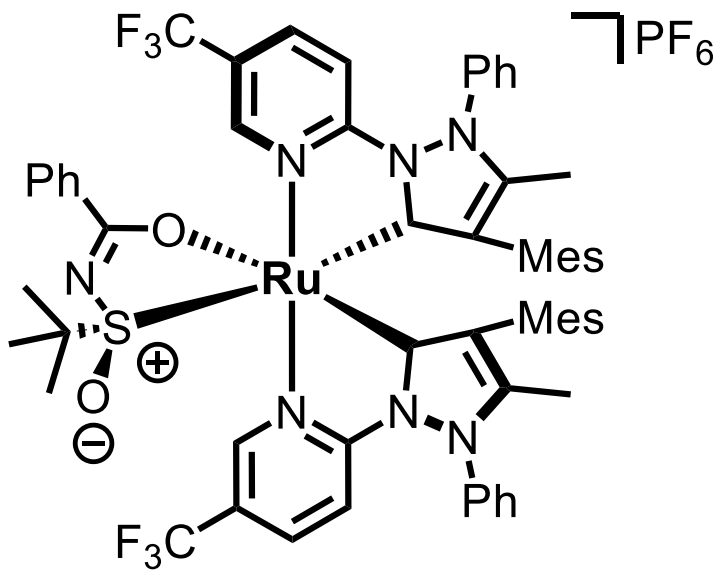

Δ-(*R*)-**Ruaux3a**

By following the general procedure, Δ-(*R*)-**Ruaux3a** (22.3 mg, 17.0 µmol, 44%) and Λ-**Ru3** (13.6 mg, 10.3 µmol, 27%) were obtained as orange solids starting from *rac*-**Ru3** (50.3 mg, 38.2 mmol, 1.00 eq.) Spectroscopic data of Λ-**Ru3** were consistent with *rac*-**Ru3**.

Analytical data for Δ-(*R*)-**Ruaux3a**: **^{1}H NMR** (300 MHz, CD_3CN, 300 K): *δ* = 10.74 – 10.73 (m, 1H), 8.29 – 8.27 (m, 1H), 8.21 – 8.18 (m, 2H), 7.88 – 7.69 (m, 7H), 7.61 (t, J = 7.7 Hz, 1H), 7.54 – 7.48 (m, 2H), 7.46 – 7.40 (m, 3H), 7.17 (d, J = 7.2 Hz, 2H), 6.70 (s, 1H), 6.65 (s, 1H), 6.61 (s, 1H), 6.56 (s, 1H), 6.52 (d, J = 9.0 Hz, 1H), 6.43 (d, J = 9.0 Hz, 1H), 2.17 (s, 3H), 2.14 (s, 3H), 2.10 (s, 3H), 2.09 (s, 6H), 1.74 (s, 3H), 1.72 (s, 3H), 1.68 (s, 3H), 1.61 (s, 3H), 0.83 (s, 9H) ppm. **^{13}C NMR** (126 MHz, CD_3CN, 300 K): *δ* = 207.7, 203.0, 180.6, 155.9, 155.8, 155.0, 154.9, 153.3 (q, *J* = 4.6 Hz), 148.5 (q, *J* = 4.7 Hz), 138.9, 138.9, 138.7, 138.5, 137.4, 137.3, 137.1, 136.6, 135.7 (q, *J* = 3.2 Hz), 135.6 (q, *J* = 3.4 Hz), 135.3, 133.0, 132.7, 132.7, 132.5, 132.5, 132.4, 132.1, 131.7, 131.5, 130.4, 129.9, 129.8, 129.8, 129.4, 129.2, 128.9, 128.4, 128.4, 128.1, 127.6, 127.1, 122.8 (q, *J* = 271.6 Hz), 122.7 (q, *J* = 34.3 Hz), 122.6 (q, *J* = 34.3 Hz), 122.1 (q, *J* = 271.6 Hz), 112.4, 112.1, 66.6, 54.9, 23.9, 21.3, 20.9, 20.8, 20.8, 20.5, 10.9, 10.8 ppm. **^{19}F NMR** (282 MHz, CD_3CN, 300 K): *δ* = -62.86 (s, CF_3), -63.81 (s, CF_3), -72.91 (d, *J* = 706.4 Hz; PF_6) ppm. **IR** (neat): $\tilde{\nu}$ = 2923 (w), 1614 (w), 1543 (w), 1487 (w), 1436 (w), 1415 (w), 1366 (w), 1326 (m), 1247 (w), 1200 (w), 1174 (w), 1137 (w), 1114 (w), 1082 (w), 1030 (w), 1006 (w), 963 (w), 936 (w), 842 (m), 786 (w), 738 (w), 716 (w), 624 (w), 596 (w), 557 (w), 509 (w), 484 (w), 431 (w) cm^{-1}. **HRMS** (ESI+): *m/z* calc. for $C_{61}H_{58}F_6N_7O_2SRu$ $[M - PF_6]^+$: 1168.3331, found: 1168.3337. **CD** (MeCN): λ, nm (Δε, M^{-1} cm^{-1}) 212 (59), 259 (−40), 277 (1), 286 (−1), 306 (−53), 354 (14), 393 (10), 471 (−17).

General procedure for the auxiliary cleavage: The corresponding auxiliary complex (Δ-(*R*)-**Ruaux2a**, Λ-(*S*)-**Ruaux2a** or Δ-(*R*)-**Ruaux3a**; 1.00 eq.) was dissolved in a mixture of dichloromethane/acetonitrile (20:1) and applied on a packed silica column with a NH_4PF_6 Plug (at least 1 cm). Immediate cleavage on the plug and silica can be observed by a color change from dark to lighter orange. After sufficient time for the cleavage, elution of the desired complexes was accelerated by applying slight positive pressure and changing the gradient (CH_2Cl_2/MeCN 20:1 → 15:1 → 10:1 → 5:1). Then, the solvent was removed under reduced pressure and the residue was taken up in dichloromethane/acetonitrile (20:1) and filtered over celite to remove traces of salt which elute at a polar gradient. The enantiopurity of the complexes were determined via the ^{19}F NMR method.

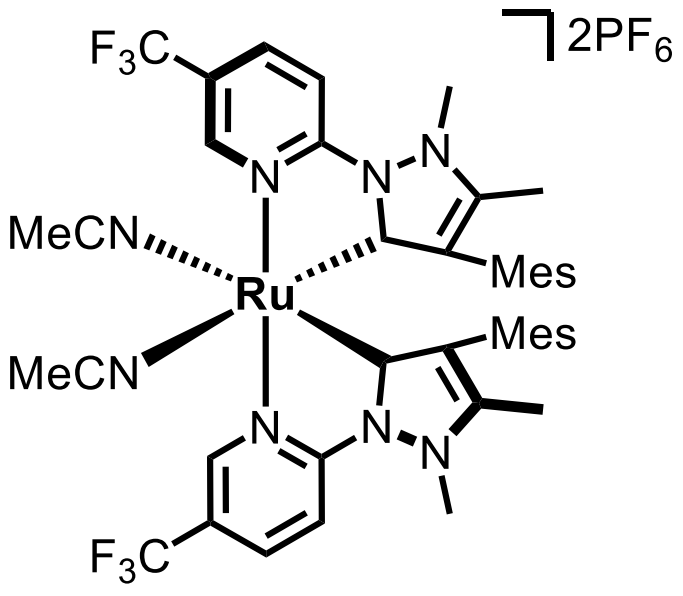

Δ-**Ru2**

Δ-**Ru2**:

By following the general procedure, Δ-**Ru2** (98% ee, 5.3 mg, 4.45 µmol, 77%) was obtained as orange solid starting from Δ-(*R*)-**Ruaux2a** (6.9 mg, 5.80 µmol, 1.00 eq.). Spectroscopic data of Δ-**Ru2** were consistent with *rac*-**Ru2**. **CD** (MeCN): λ, nm (Δε, M^{-1} cm^{-1}) 208 (−32), 251 (42), 269 (−2), 279 (1), 288 (−9), 304 (30), 347 (−10), 438 (6), 460 (3), 503 (5).

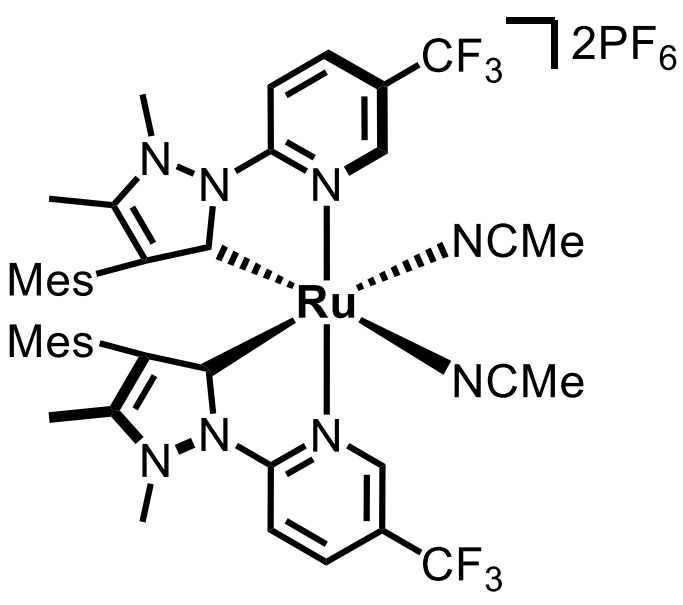

Λ-**Ru2**

Λ-**Ru2**:

By following the general procedure, Λ-**Ru2** (93% ee, 6.4 mg, 6.14 µmol, 87%) was obtained as orange solid starting from Λ-(*S*)-**Ruaux2a** (7.3 mg, 6.14 µmol, 1.00 eq.). Spectroscopic data of Λ-**Ru2** were consistent with *rac*-**Ru2**. ***Synthetic note*:** Small impurities after column chromatography of the auxiliary complex presumably led to the slightly diminished enantiomeric purity compared to Δ-**Ru2**. **CD** (MeCN): λ, nm (Δε, M^{-1} cm^{-1}) 208 (27), 251 (−38), 269 (2), 279 (−1), 288 (8), 304 (−28), 347 (9), 438 (−6), 460 (−3), 503 (−5).

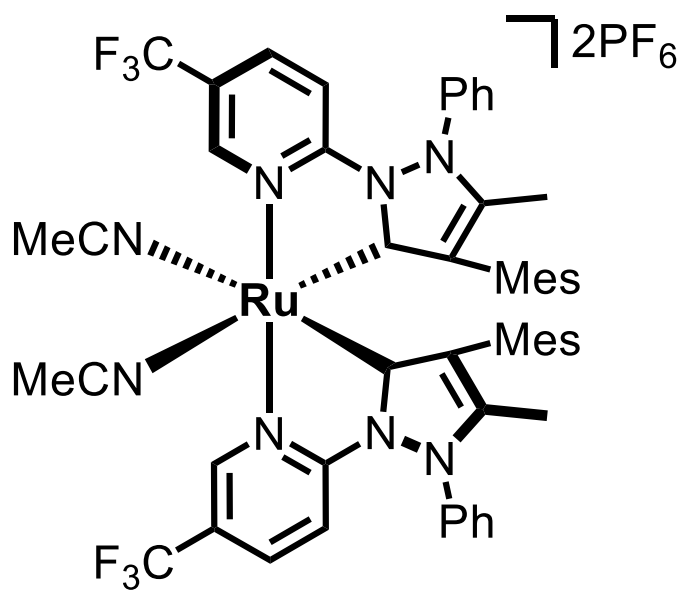

Δ-**Ru3**

Δ-**Ru3**:

By following the general procedure, Δ-**Ru3** (98% ee, 15.1 mg, 11.5 µmol, 75%) was obtained as orange solid starting from Δ-(*R*)-**Ruaux3a** (20.1 mg, 15.3 µmol, 1.00 eq.). Spectroscopic data of Δ-**Ru3** were consistent with *rac*-**Ru3**. **CD** (MeCN): λ, nm (Δε, M^{-1} cm^{-1}) 253 (−26), 271(3), 286 (−7), 304 (−45), 344 (12), 372 (1), 389 (5), 437 (−17).

5. Determination of the Enantiomeric Excess of the Ruthenium Complexes by ^{19}F NMR

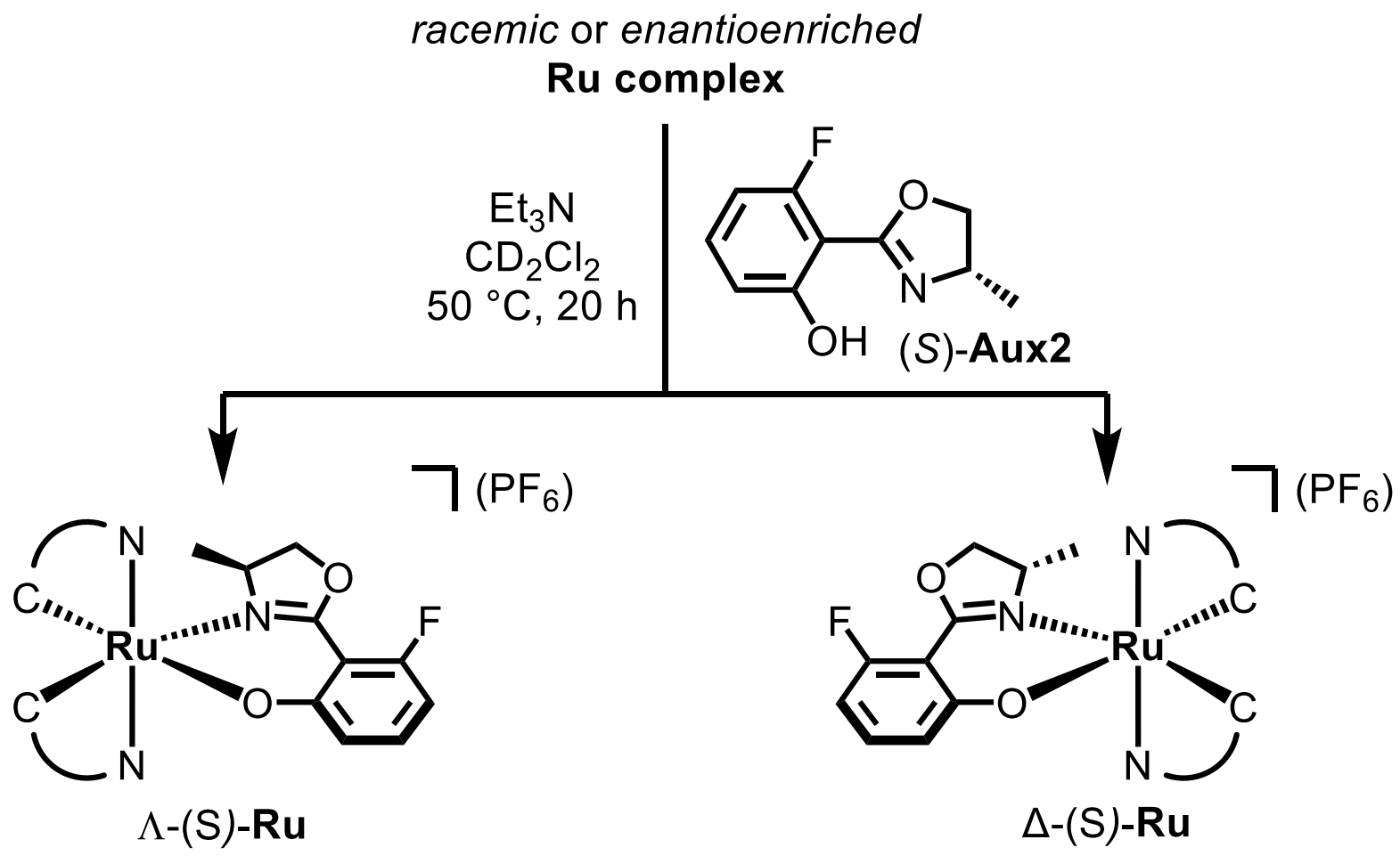

The enantiopurity of all non-racemic complexes was determined indirectly by using ^{19}F NMR spectroscopy after coordination of the chiral auxiliary (*S*)-**Aux2**. As both complex enantiomers react to the thermodynamic stable auxiliary complexes Λ-(*S*)-**Ru** and Δ-(*S*)-**Ru**, their ratio can be determined by integration of the signal of the fluorine group on the coordinating auxiliary of each diastereomer. Thus, direct conclusions on the ratio of enantiomers can be made.

For this, by following a modified procedure of Demirel *et al.*,[8] the corresponding ruthenium complex (*rac*- or Λ- or Δ-; 2.50 µmol, 1.00 eq.) and (*S*)-**Aux2** (0.51 mg, 2.63 µmol, 1.05 eq.) were dissolved in CD_2Cl_2 (0.50 mL, 0.005 M) and triethylamine (5.2 µL, 3.75 µmol, 1.50 eq.) was added. The solution was stirred at 50 °C for 20 h and a ^{19}F NMR spectrum (300 MHz, 300 K, 1000 scans) was subsequently measured.

Mixture (d.r.: 50:50) of Λ-(*S*)-**Ruaux2b** and Δ-(*S*)-**Ruaux2b** resulting from *rac*-**Ru2**:

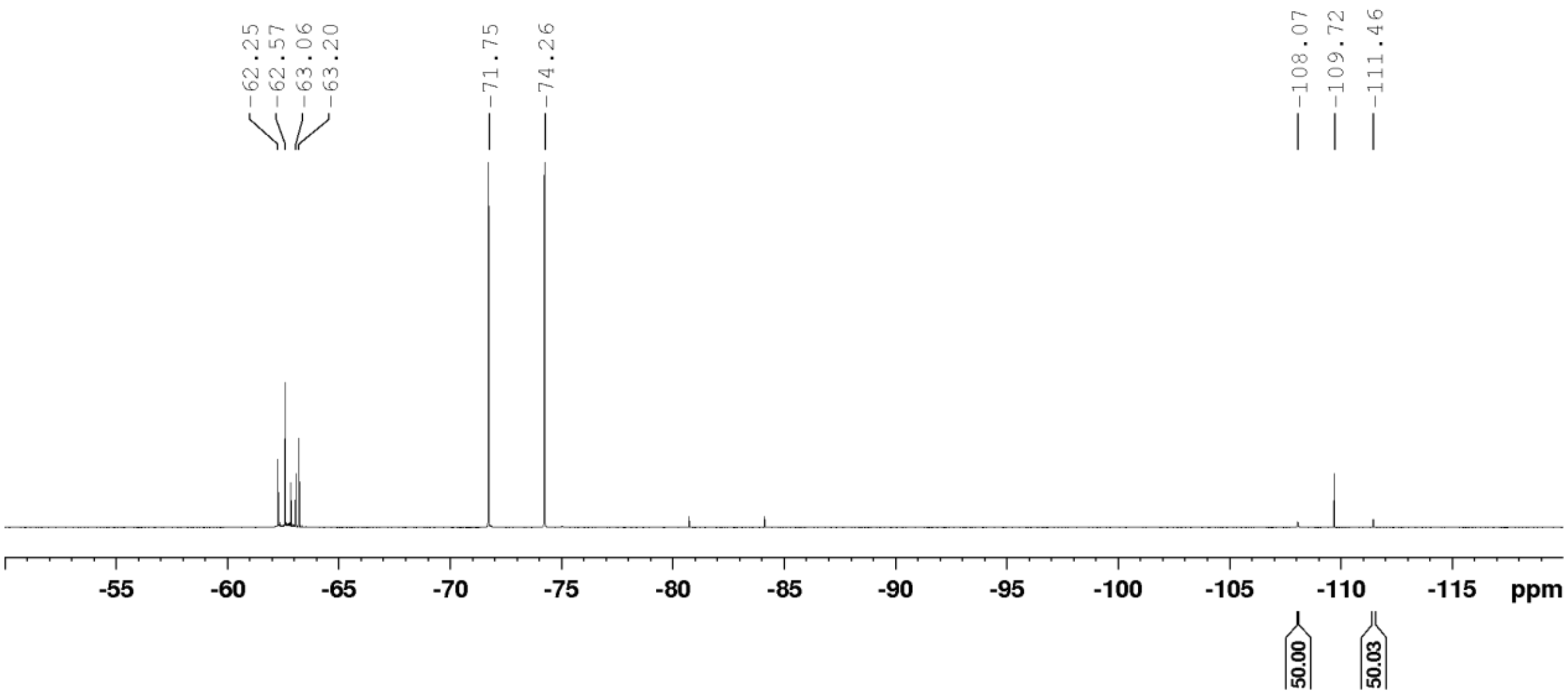

Λ-(*S*)-**Ruaux2b** resulting from enantioenriched Λ-**Ru2** (1% ee):

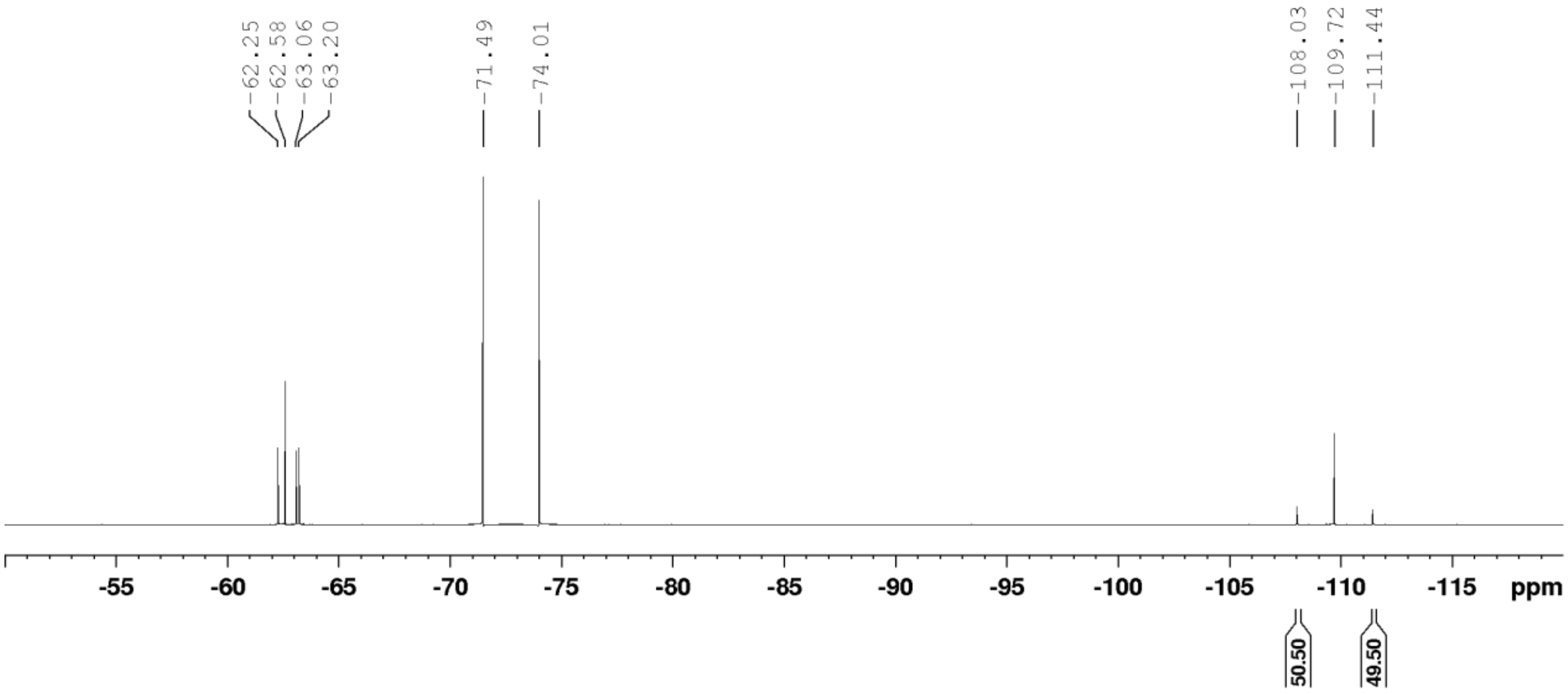

Λ-(*S*)-**Ruaux2b** resulting from enantioenriched Λ-**Ru2** (93% ee):

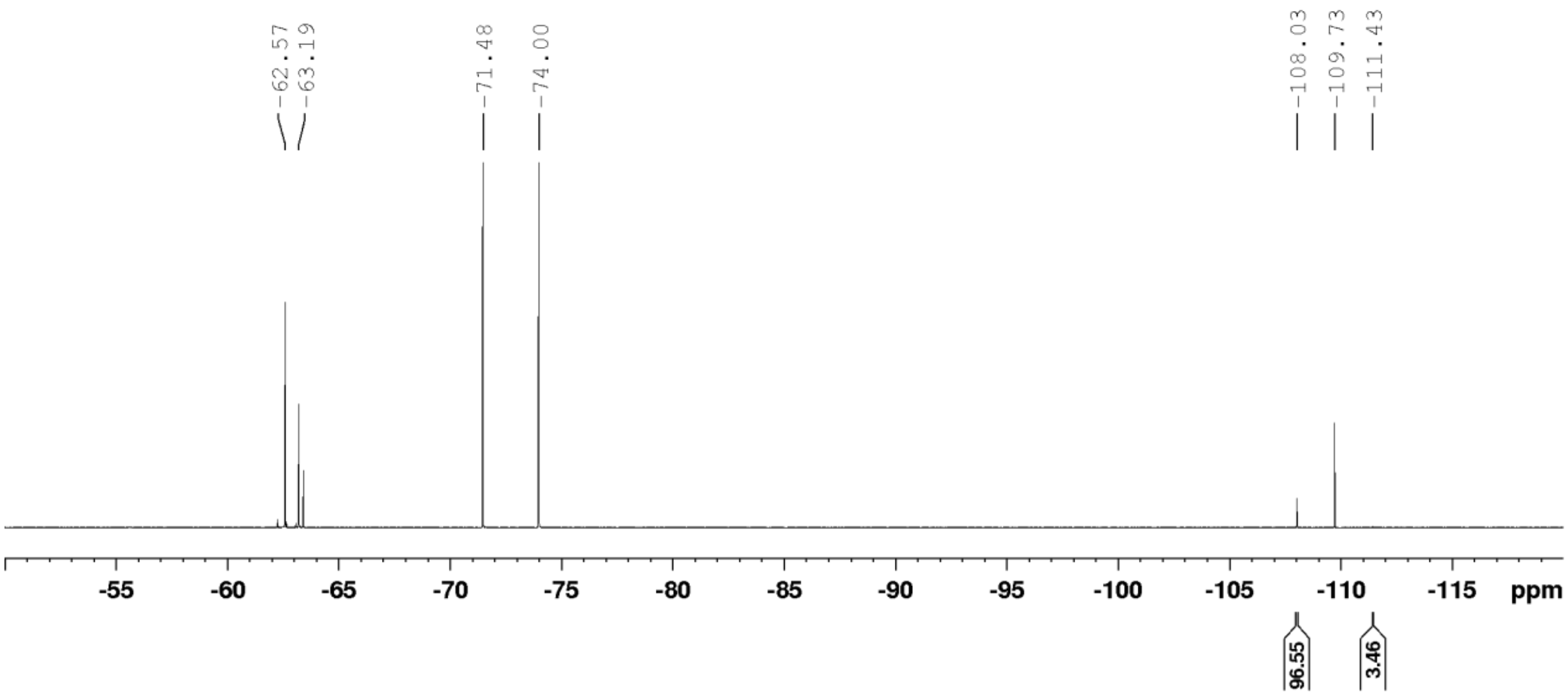

Δ-(*S*)-**Ruaux2b** resulting from enantioenriched Δ-**Ru2** (98% ee):

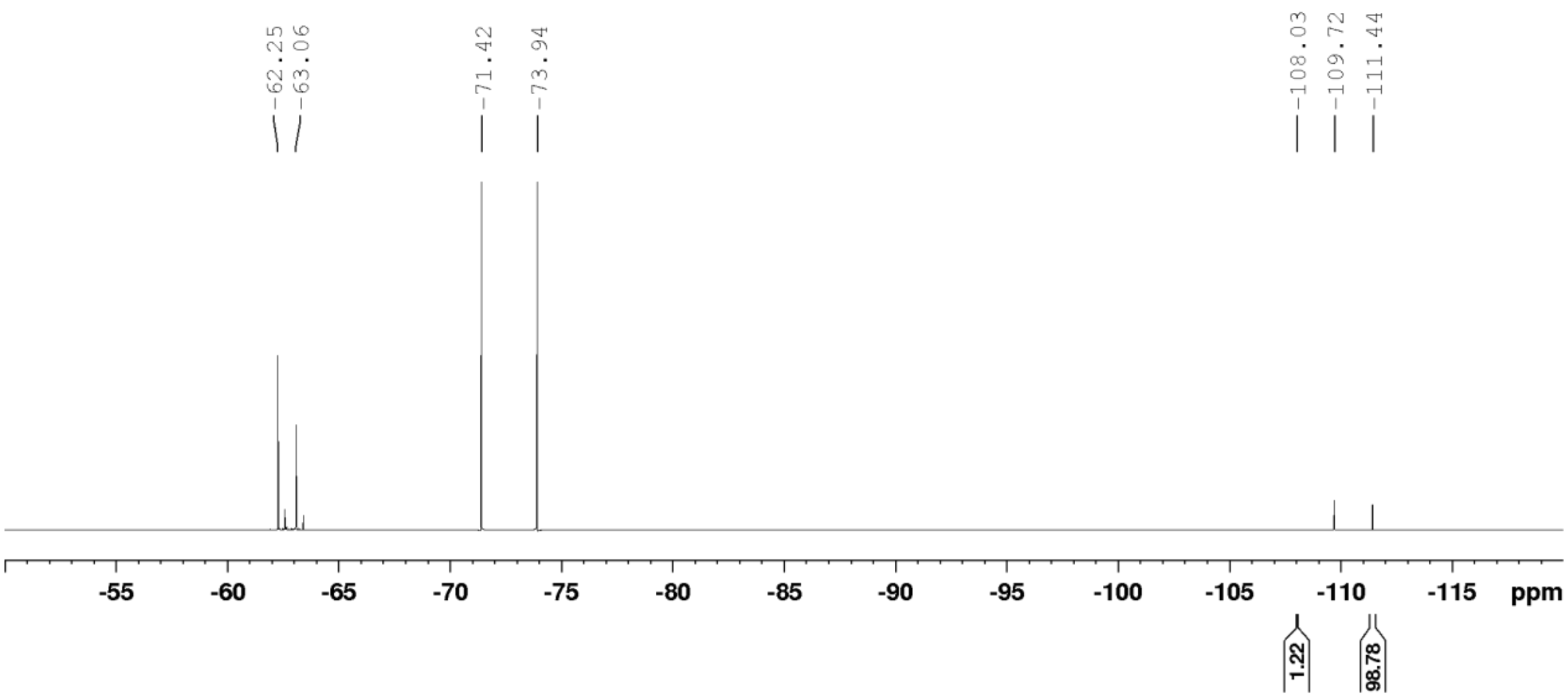

Mixture (d.r.: 50:50) of Λ-(*S*)-**Ruaux3b** and Δ-(*S*)-**Ruaux3b** resulting from *rac*-**Ru3**:

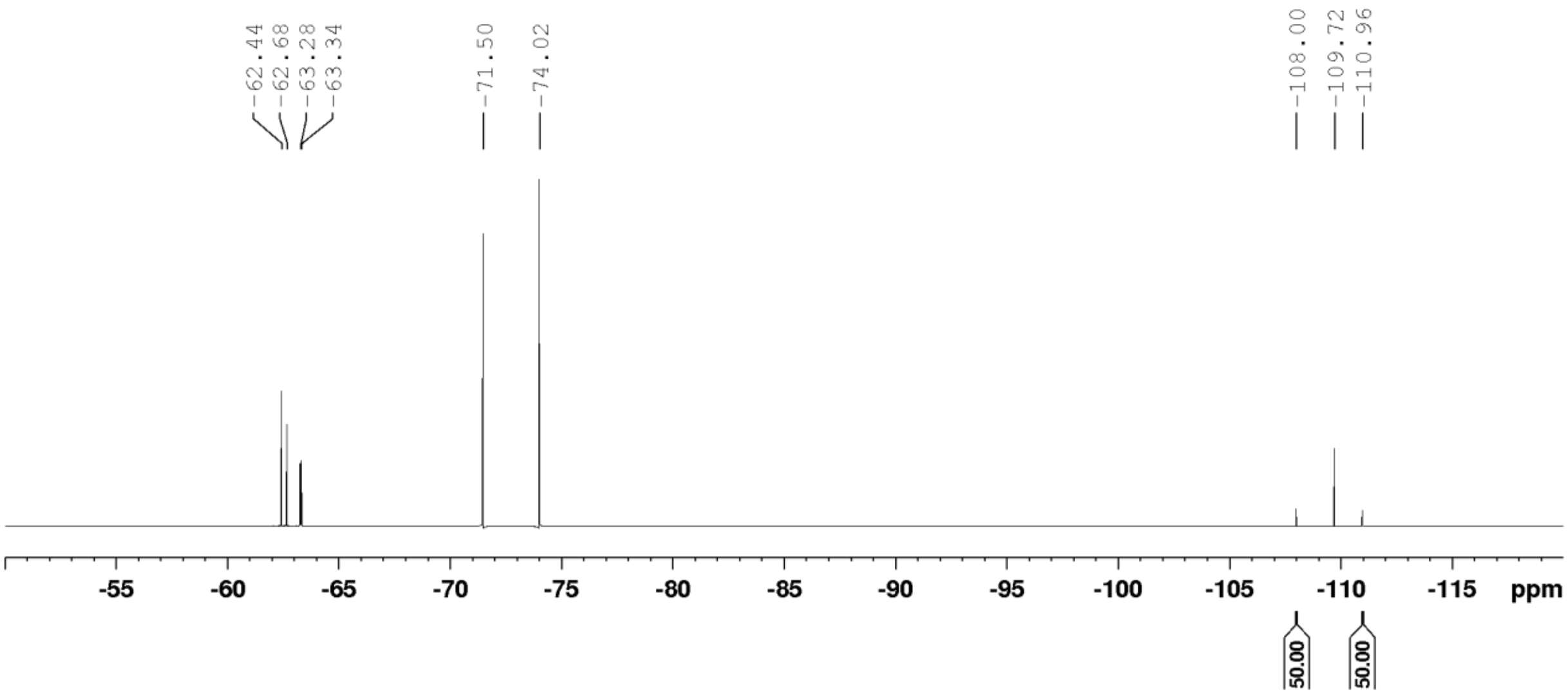

Λ-(*S*)-**Ruaux3b** resulting from enantioenriched Λ-**Ru3** (67% ee):

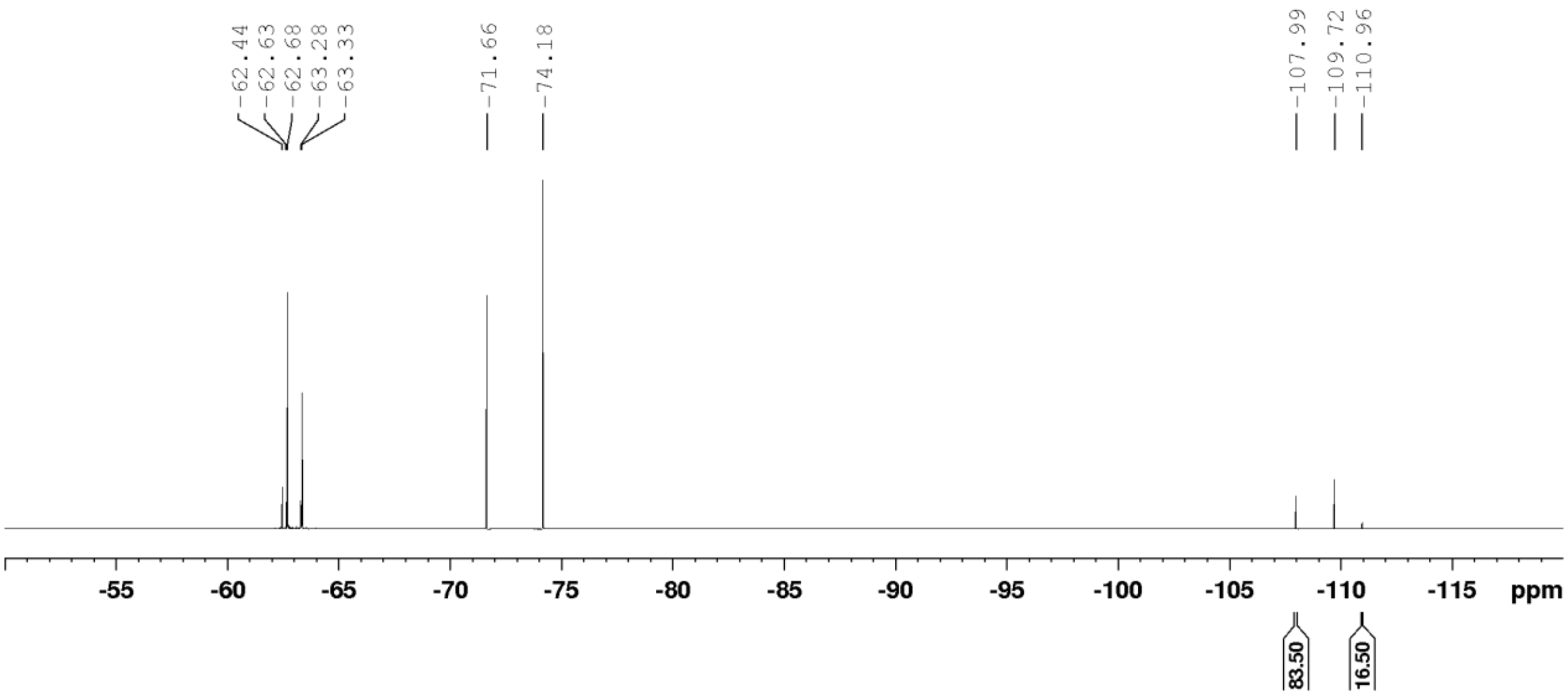

Δ-(*S*)-**Ruaux3b** resulting from enantioenriched Δ-**Ru3** (98% ee):

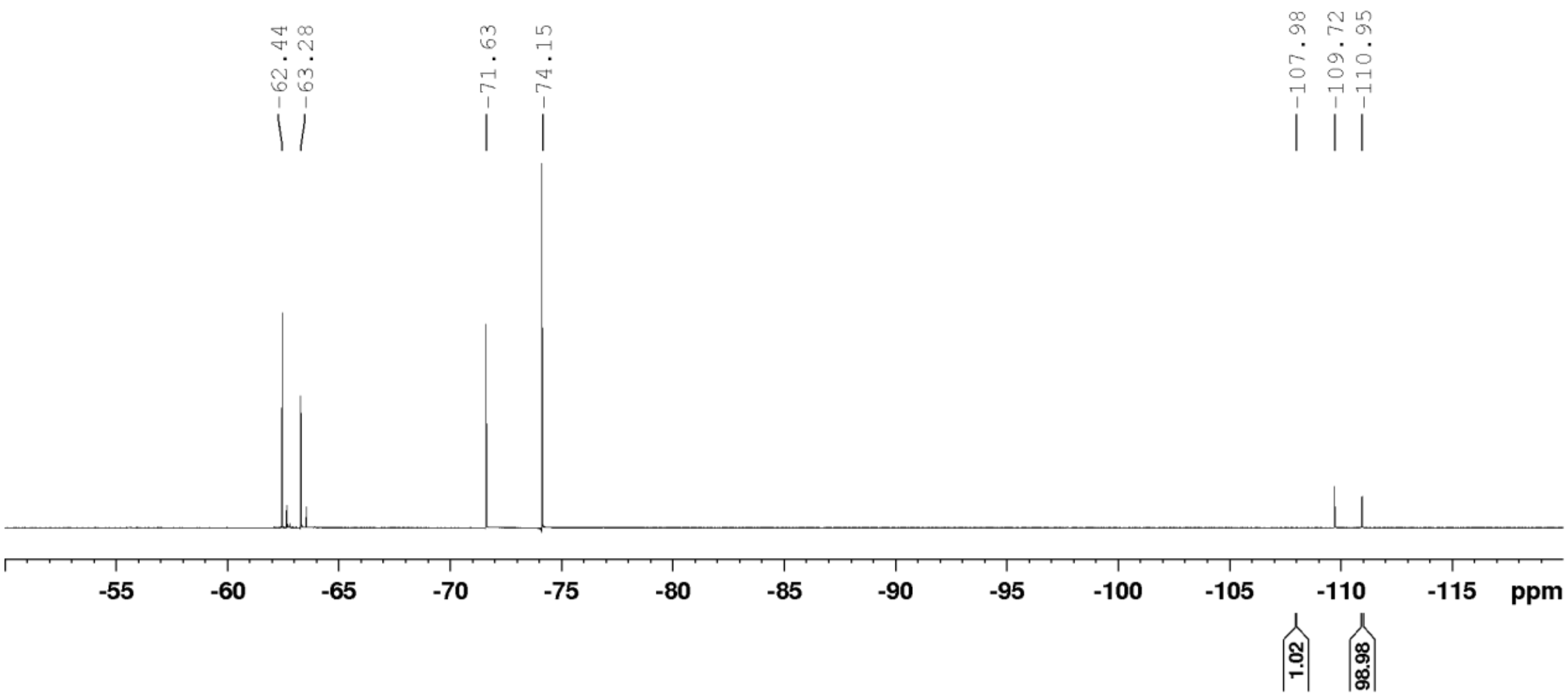

6. Investigation of the Electronic Properties of the Pyrazolium Ligands

The σ-donor strength of the pyrazolin-5-ylidene NHC ligands was tentatively estimated by the determination of the $^{1}J_{CH}$ coupling constant of the azolium carbon atom within the pyrazolium salts **4a-d** as direct NHC precursor.[9,10] The coupling constants were obtained from the ^{13}C satellites of the ^{1}H NMR spectra (300 MHz, $CDCl_3$, 300 K) and are in the range of 195.2-199.2 Hz. Comparison with other bidentate NHC ligands, which are based on *N*-(2-pyridyl)-substituted *N*-heterocyclic framework, shows that the pyrazolin-5-ylidene ligands are stronger σ-donors than benzimidazolin-2-ylidene and imidazolin-2-ylidene ligands (nNHC) and even the triazolin-5-ylidene ligands (MIC) (Figure S2).[11]

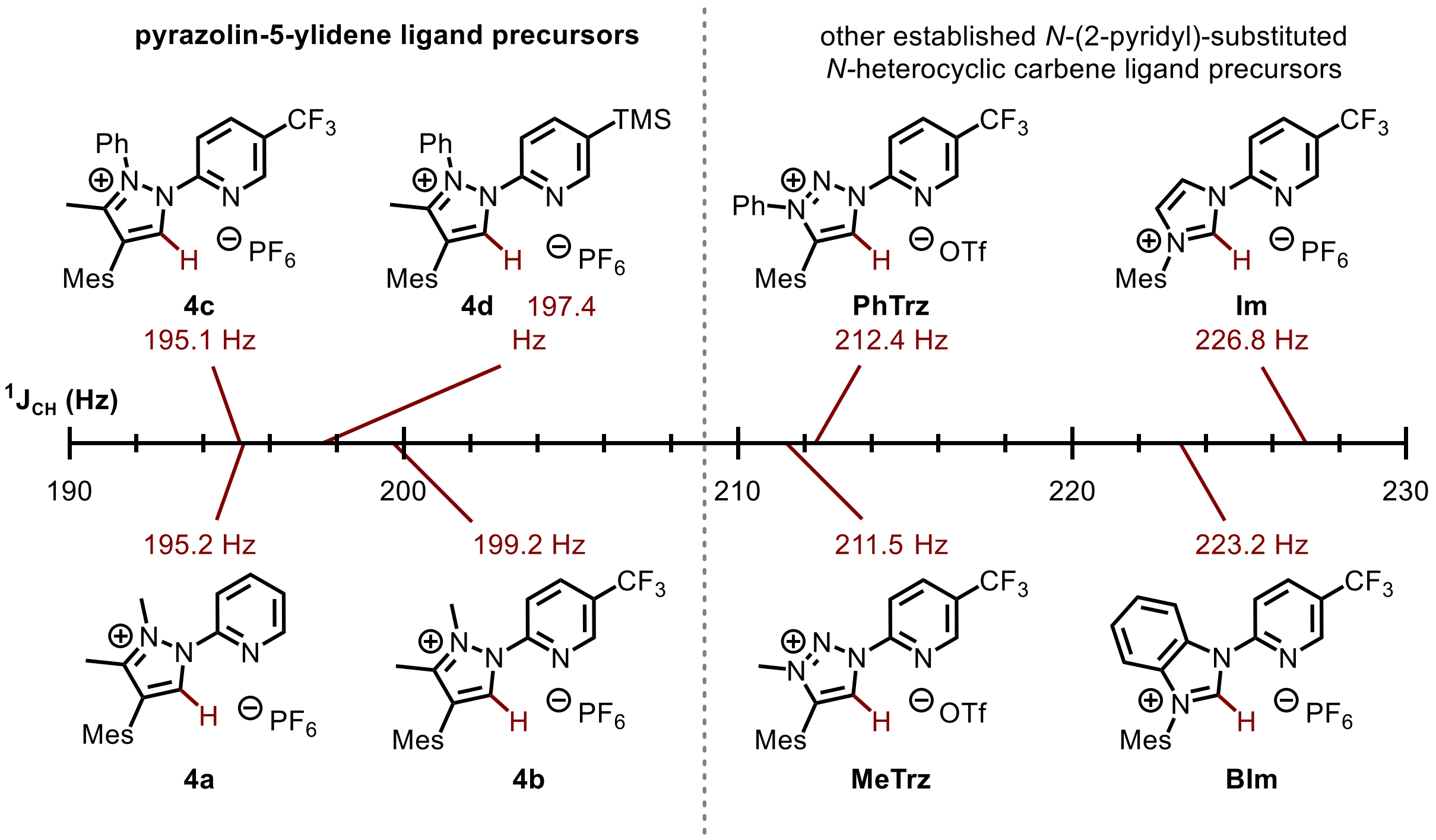

Figure S2: Comparison of the $^{1}J_{CH}$ coupling of the azolium salts of the synthesized ligand precursor **4a-d** with selected examples for other established *N*-(2-pyridyl)-substituted *N*-heterocyclic carbene ligand precursors (**PhTrz**: phenylated triazolium; **MeTrz**: methylated triazolium; **Bim**: benzimidazolium; **Im**: imidazolium).[11]

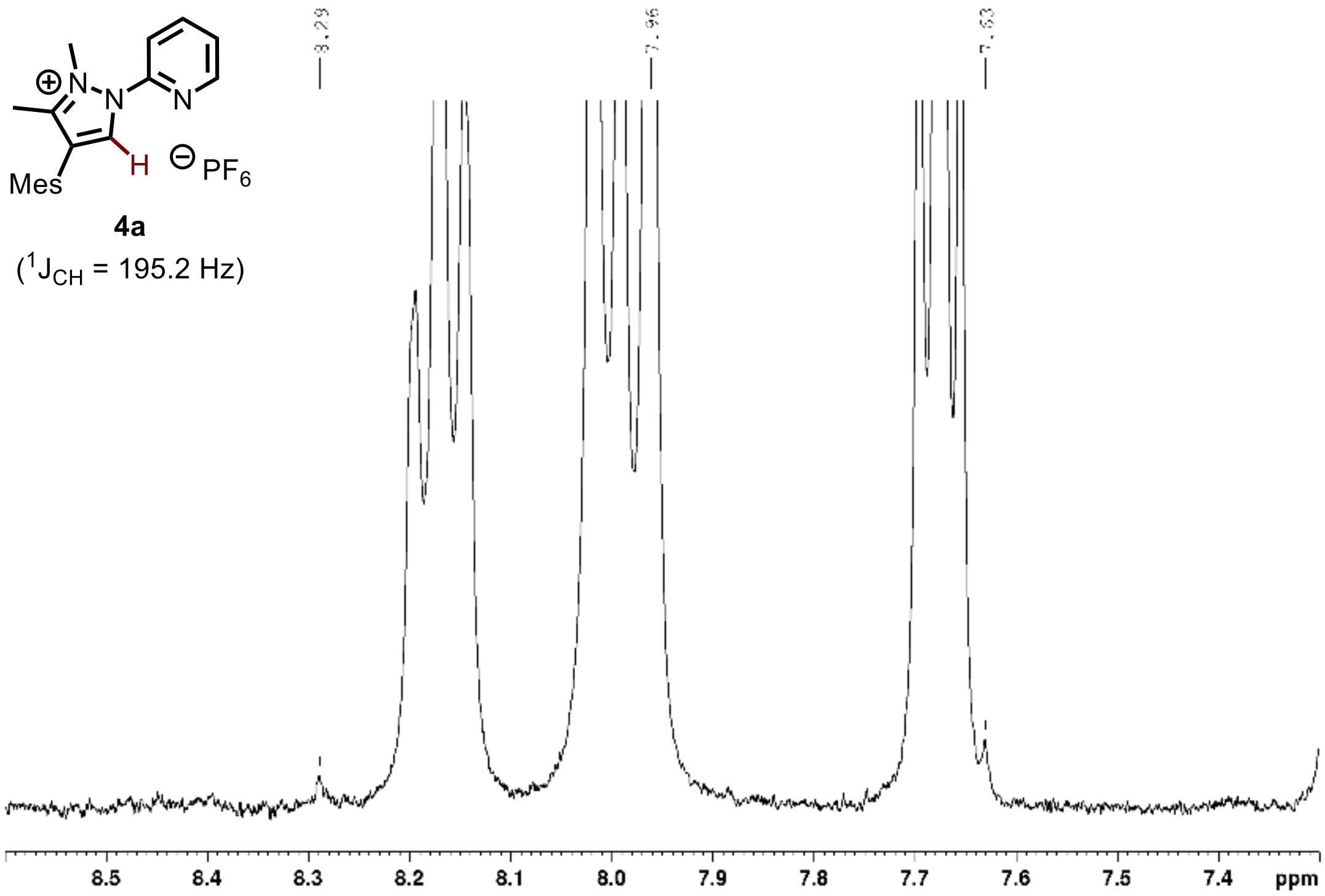

Figure S3: Excerpt of the ^{1}H NMR spectrum of **4a** (300 MHz, $CDCl_3$, 300 K).

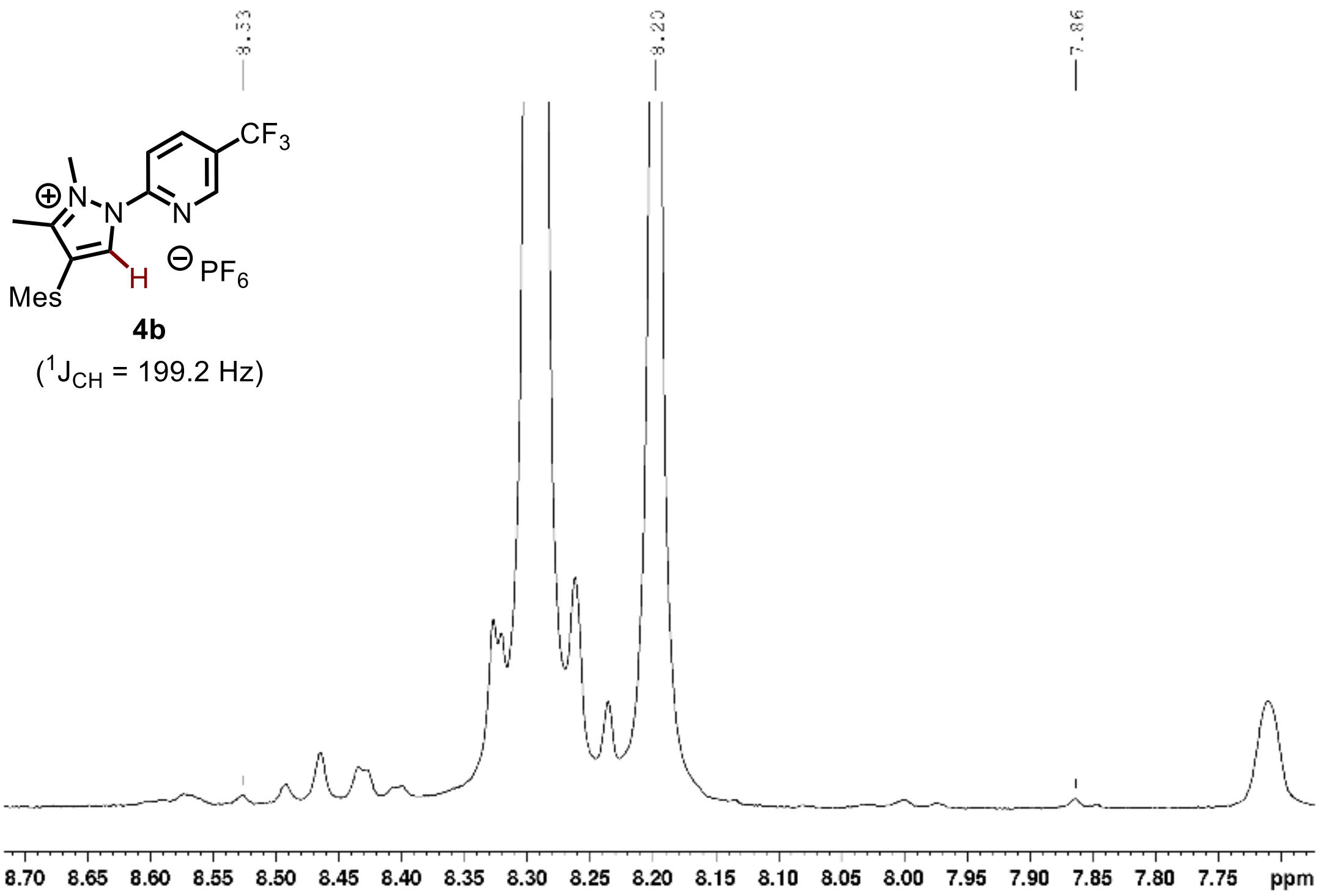

Figure S4: Excerpt of the ^{1}H NMR spectrum of **4b** (300 MHz, $CDCl_3$, 300 K).

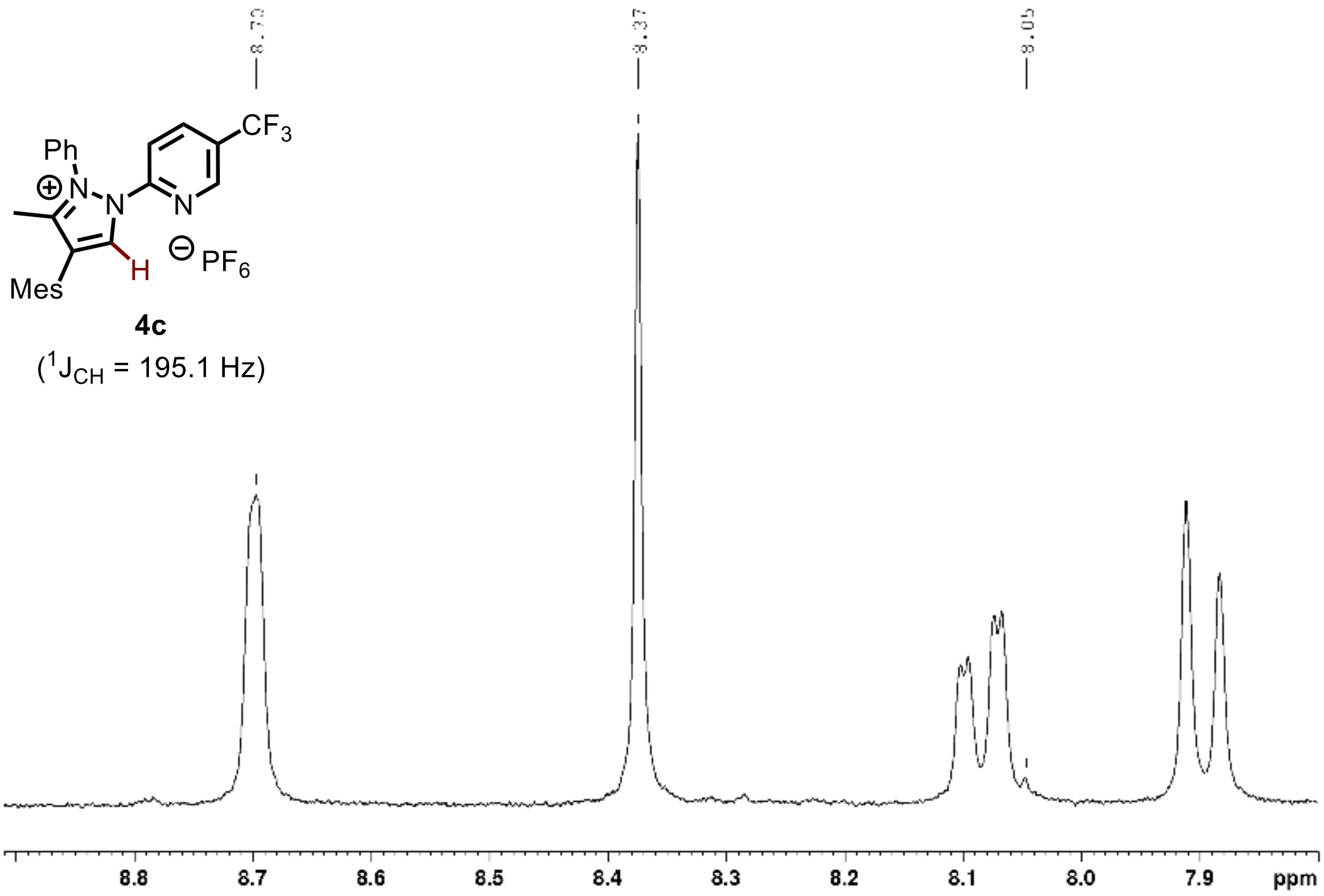

Figure S5: Excerpt of the ^{1}H NMR spectrum of **4c** (300 MHz, $CDCl_3$, 300 K).

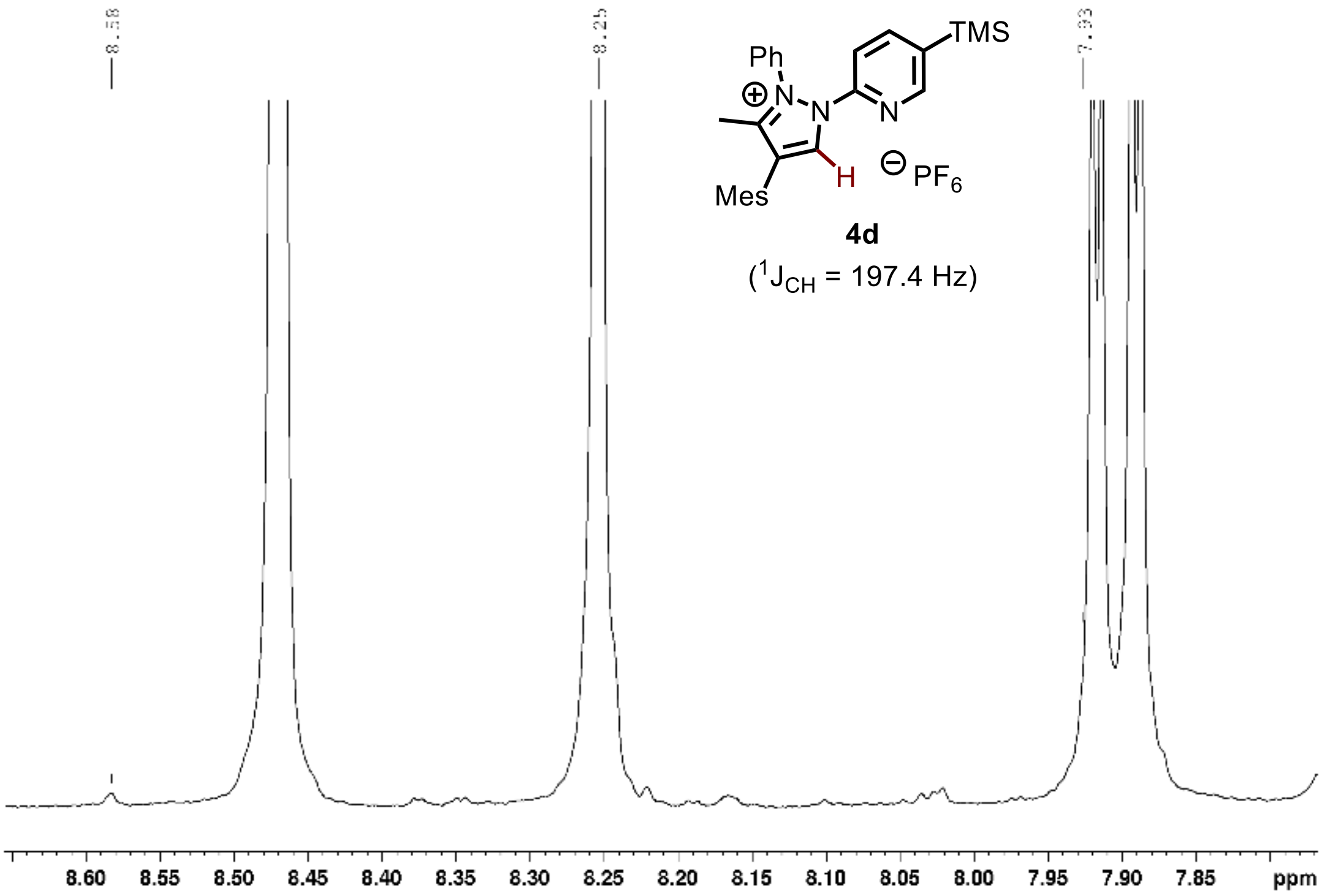

Figure S6: Excerpt of the ^{1}H NMR spectrum of **4d** (300 MHz, $CDCl_3$, 300 K).

7. Stability Investigations of *rac*-Ru1 in CD_3CN

Similar to results obtained at room temperature, *rac*-**Ru1** as well as the other pyrazolin-5-ylidene ruthenium complexes show a high stability in acetonitrile as coordinating solvent under air. For examination by ^1H NMR, a sample of *rac*-**Ru1** (Figure S7, a) in CD_3CN was stored under air at 4 °C and measured after 7 days (b) and 4 weeks (c), while no indications of decomposition could be found.

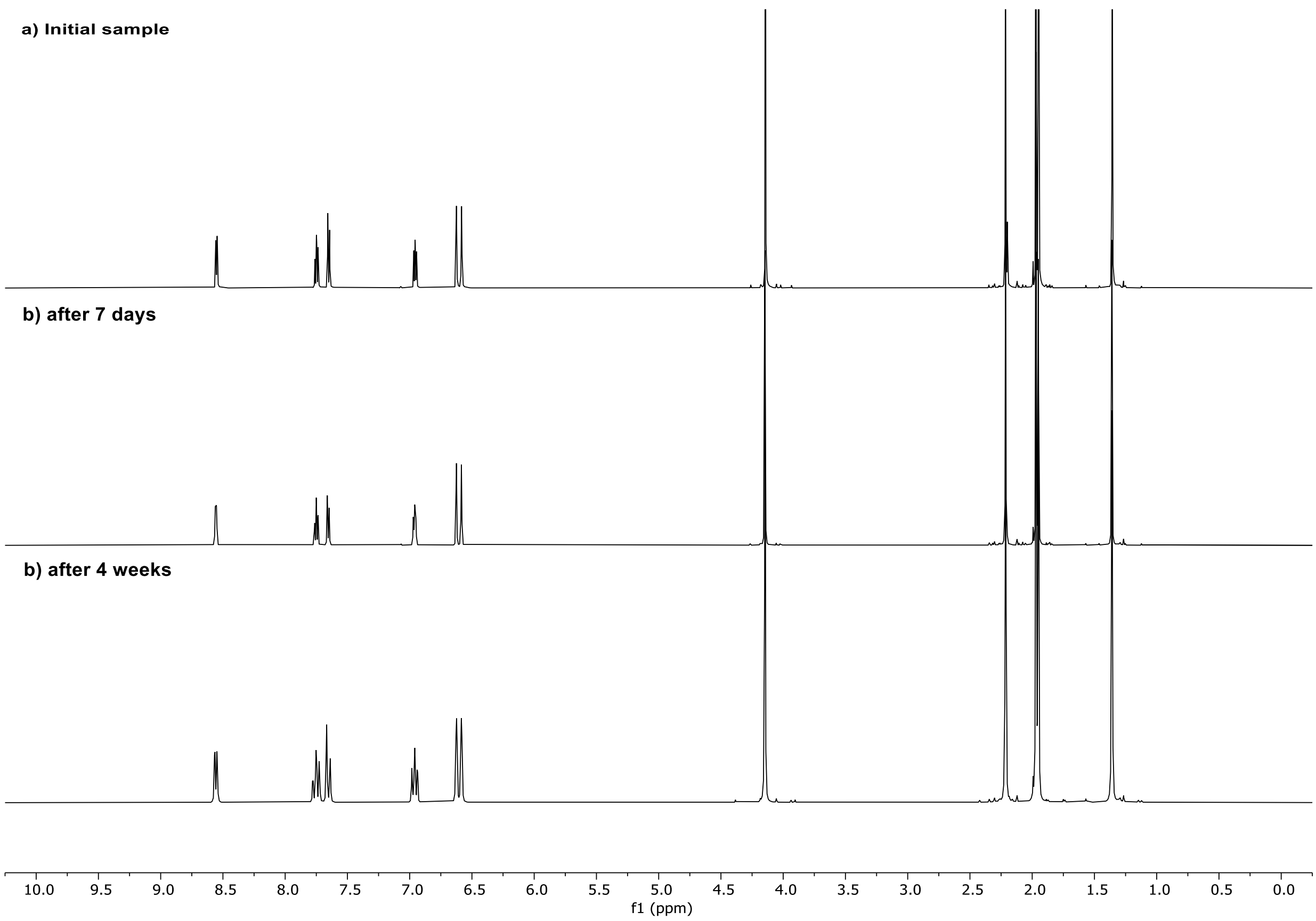

Figure S7: ^1H NMR spectra (300 or 600 MHz, 300 K) of *rac*-**Ru1** after different periods of storage in CD_3CN at 4 °C under air.

8. Substrate Synthesis

(*E*)-3-Hydroxy-1-methyl-1-(3-phenylbut-2-en-1-yl)urea (S2)

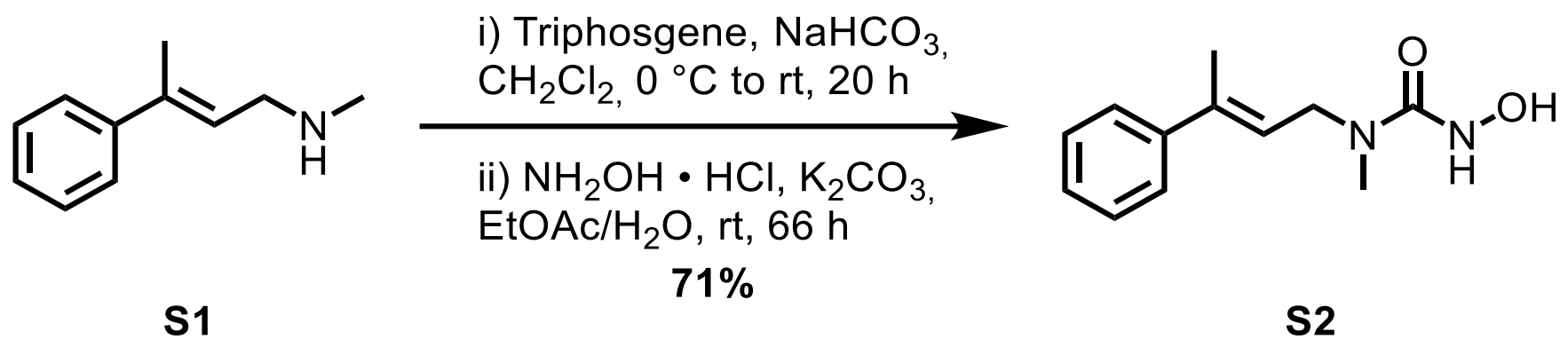

Following a modified procedure of Ren *et al.*,[12] triphosgene (1.54 g, 5.18 mmol, 0.70 eq.) and $NaHCO_3$ (1.24 g, 14.8 mmol, 2.00 eq.) were suspended in dichloromethane (45.0 mL) under an atmosphere of nitrogen and the mixture was cooled to 0 °C. Then, a solution of amine **S1** (1.09 g, 7.40 mmol, 1.00 eq.) in dichloromethane (15.0 mL) was added dropwise and the solution was allowed to warm to room temperature and stirred for 20 h. The reaction mixture was filtered over celite and the solvent was removed under reduced pressure. The residue was suspended in a mixture of ethyl acetate/water (40:1, 30.8 mL) and NH_2OH · HCl (823 mg, 11.8 mmol, 1.60 eq.) and potassium carbonate (1.53 g, 11.1 mmol, 1.50 eq.) were added at room temperature. The reaction mixture was stirred at room temperature for 66 h (***Synthetic note***: long reaction time may not be necessary) and then dried over Na_2SO_4 and filtered. The solvent was removed under reduced pressure and the crude product was purified by column chromatography on silica (*n*-hexane/ethyl acetate 95:5 → 50:50) to obtain urea **S2** (1.17 g, 5.29 mmol, 71%) as colorless solid.

^{1}H NMR (300 MHz, $CDCl_3$, 300 K): δ = 7.39 – 7.24 (m, 5H), 7.01 (s, 1H), 5.70 (td, J = 6.7 Hz, 1H), 4.09 (d, J = 6.6 Hz, 2H), 2.91 (s, 3H), 2.10 (s, 3H) ppm. **^{13}C NMR** (75 MHz, $CDCl_3$, 300 K): δ = 161.8, 142.6, 139.4, 128.5, 127.6, 125.9, 122.3, 46.9, 33.6, 16.2 ppm. **IR** (neat): $\tilde{\nu}$ = 3233 (w), 2930 (w), 2247 (w), 1678 (s), 1599 (m), 1583 (w), 1492 (m), 1445 (s), 1403 (m), 1359 (m), 1266 (s), 1180 (m), 1157 (w), 1101 (m), 1066 (m), 1025 (m), 957 (m), 910 (m), 848 (w), 759 (s), 728 (s), 690 (s), 646 (m), 588 (s) cm^{-1}. **HRMS** (ESI+): *m/z* calc. for $C_{12}H_{16}N_2O_2Na$ [M + Na]$^+$: 243.1104, found: 243.1101.

(*E*)-3-(Benzoyloxy)-1-methyl-1-(3-phenylbut-2-en-1-yl)urea (1)

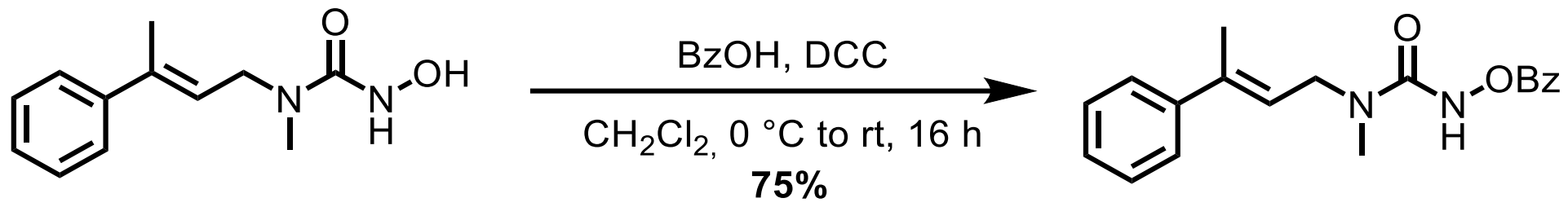

S2 **5**

Following a modified procedure of Jarrige *et al.*,[13] urea **S2** (265 mg, 1.20 mmol, 1.00 eq.) and benzoic acid (343 mg, 1.32 mmol, 1.10 eq.) were dissolved in dichloromethane (3.00 mL) and the mixture was cooled to 0 °C. Then, a solution of DCC (273 mg, 1.32 mmol, 1.10 eq.) in dichloromethane (3.00 mL) was added dropwise and the solution was allowed to warm to room temperature and stirred for 16 h. Diethyl ether (12.0 mL) was added and the suspension was filtered over celite and rinsed with additional diethyl ether (50.0 mL). The solution was washed with water (2 x 50.0 mL) and the solvent was removed under reduced pressure. The crude product was purified by column chromatography on silica (n-hexane/ethyl acetate 0:100 → 60:40) to obtain urea *N*-benzoyl urea **5** (292 mg, 899 µmol, 75%) as colorless solid.

^{1}H NMR (300 MHz, $CDCl_3$): δ = 8.53 (s, 1H), 8.13 – 8.10 (m, 2H), 7.64 – 7.58 (m, 1H), 7.49 – 7.40 (m, 4H), 7.37 – 7.27 (m, 3H), 5.80 (td, *J* = 6.8, 1.4 Hz, 1H), 4.17 (d, *J* = 6.8 Hz, 2H), 3.04 (s, 3H), 2.14 (s, 3H) ppm. **^{13}C NMR** (75 MHz, $CDCl_3$): δ = 166.9, 157.9, 142.7, 139.7, 134.1, 130.1, 128.7, 128.5, 127.6, 127.2, 125.9, 122.2, 47.2, 34.2, 16.3 ppm. **IR** (neat): $\tilde{\nu}$ = 3213 (w), 3059 (w), 2925 (w), 2249 (w), 1755 (m), 1667 (m), 1600 (w), 1491 (m), 1451 (m), 1382 (w), 1316 (w), 1240 (m), 1178 (w), 1151 (w), 1081 (m), 1065 (m), 1016 (m), 964 (w), 910 (w), 891 (w), 857 (w), 798 (w), 759 (m), 732 (m), 696 (s), 647 (w), 617 (w), 551 (w), 479 (w), 443 (w), 409 (w) cm^{-1}. **HRMS** (ESI+): *m/z* calc. for $C_{19}H_{20}N_2O_3Na$ $[M + Na]^+$: 347.1366, found: 347.1359.

9. Ruthenium Catalyzed Enantioselective Aziridination

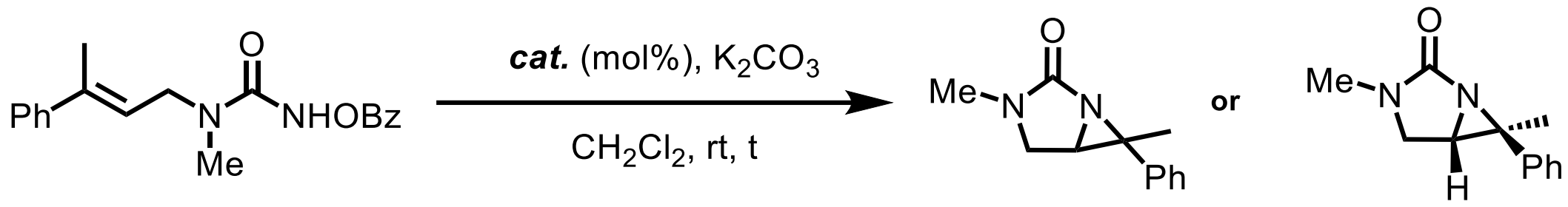

5 rac-6 (*S*,*S*)-6

An oven-dried Schlenk flask was charged with substrate **5** (32.6 mg, 100 µmol, 1.00 eq.) and K_2CO_3 (41.7 mg, 301 µmol, 3.00 eq.) under nitrogen atmosphere and catalyst (*racemic* or enantioenriched, 0.5–1.0 mol%) and dichloromethane (1.00 mL) or a freshly prepared stock solution of the catalyst in dichloromethane (for entries with enantioenriched catalyst and 0.1 or 0.05 mol% catalyst loading) was added. The resulting solution was stirred for the indicated time at the indicated temperature, before *n*-pentane (6.00 mL) was added. The suspension was filtered over celite and rinsed with a mixture of *n*-pentane and dichloromethane (4:1, 20.0 mL). The solvent was removed under reduced pressure and conversion and yield were determined by ^{1}H NMR spectroscopy of the crude product with 1,3,5-trimethoxybenzene as internal standard. The enantiomeric excess was determined by HPLC analysis of the crude product on a chiral stationary phase.

Table S1: Summary of the catalysis entries following the general procedure with the respective ruthenium catalyst (*rac*-**Ru1-4**, *rac*-**RuIm,** Δ-**Ru2** or Δ-**Ru3**).

Entry	Catalyst (mol%)	t (h)	Conversion (%)	Yield (%)	ee (%)
**1**	*rac*-**Ru1** [1.0]	3	>99	90	-
**2**	*rac*-**Ru2** [1.0]	3	>99	91	-
**3**	*rac*-**Ru3** [1.0]	3	>99	95	-
**4**	*rac*-**Ru4** [1.0]	3	>99	57	-
**5**	*rac*-**RuIm** [1.0]	3	18	13	-
**6**	Δ-**Ru2** [1.0]	3	>99	91	98
**7**	Δ-**Ru2** [0.5]	3	88	76	98
**8**	Δ-**Ru2** [0.1]	68	98	71	98
**9**	Δ-**Ru2** [0.05]	68	69	32	98
**10**	Δ-**Ru3** [1.0]	3	>99	95	98
**11**[a]	Δ-**Ru3** [1.0]	3	>99	94	98
**12**[b]	Δ-**Ru3** [1.0]	16	>99	95	98
**13**	Δ-**Ru3** [0.1]	68	>99	76	98
**14**[c]	Δ-**Ru3** [0.1]	48	>99	76	98

[a] Reaction was performed under air in HPLC-grade solvent. [b] Recycled catalyst was used (no additional purification conducted). [c] Reaction was performed at 40 °C instead of room temperature.

Analytical data for *rac*-**6**/(*S*,*S*)-**6**:

^{1}H NMR (300 MHz, $CDCl_3$): δ = 7.47 – 7.43 (m, 2H), 7.35 – 7.22 (m, 3H), 3.84 (dd, *J* = 10.7, 6.9 Hz, 1H), 3.40 (dd, *J* = 10.7, 2.0 Hz, 1H), 2.85 (s, 3H), 2.82 (dd, *J* = 6.9, 2.0 Hz, 1H), 1.54 (s, 3H) ppm. **^{13}C NMR** (75 MHz, $CDCl_3$): δ = 165.7, 142.1, 128.4, 127.5, 126.5, 48.8, 47.6, 44.9, 30.0, 13.3 ppm. **IR** (neat): $\tilde{\nu}$ = 3524 (w), 3483 (w), 3058 (w), 3028 (w), 3028 (w), 2920 (w), 2876 (w), 2874 (w), 1702 (s), 1603 (w), 1494 (m), 1428 (m), 1402 (m), 1389 (m), 1271 (s), 1197 (w), 1148 (m), 1071 (m), 1027 (w), 997 (m), 957 (w), 918 (w), 858 (w), 822 (m), 786 (w), 752 (m), 699 (m), 644 (w), 544 (w), 508 (w) cm^{-1}. **HRMS** (ESI+): *m/z* calc. for $C_{19}H_{20}N_2O_3Na$ $[M + Na]^+$: 347.1366, found: 347.1359.

HPLC conditions: Daicel Chiralpak IB N-5 column, 250 x 4.6 mm, absorbance at 230 nm, *n*-hexane/*i*PrOH 80:20, isocratic flow, flow rate 1.0 mL/min, 25 °C, t_r (minor) = 12.5 min, t_r (major) = 20.0 min.

10. NMR Spectra

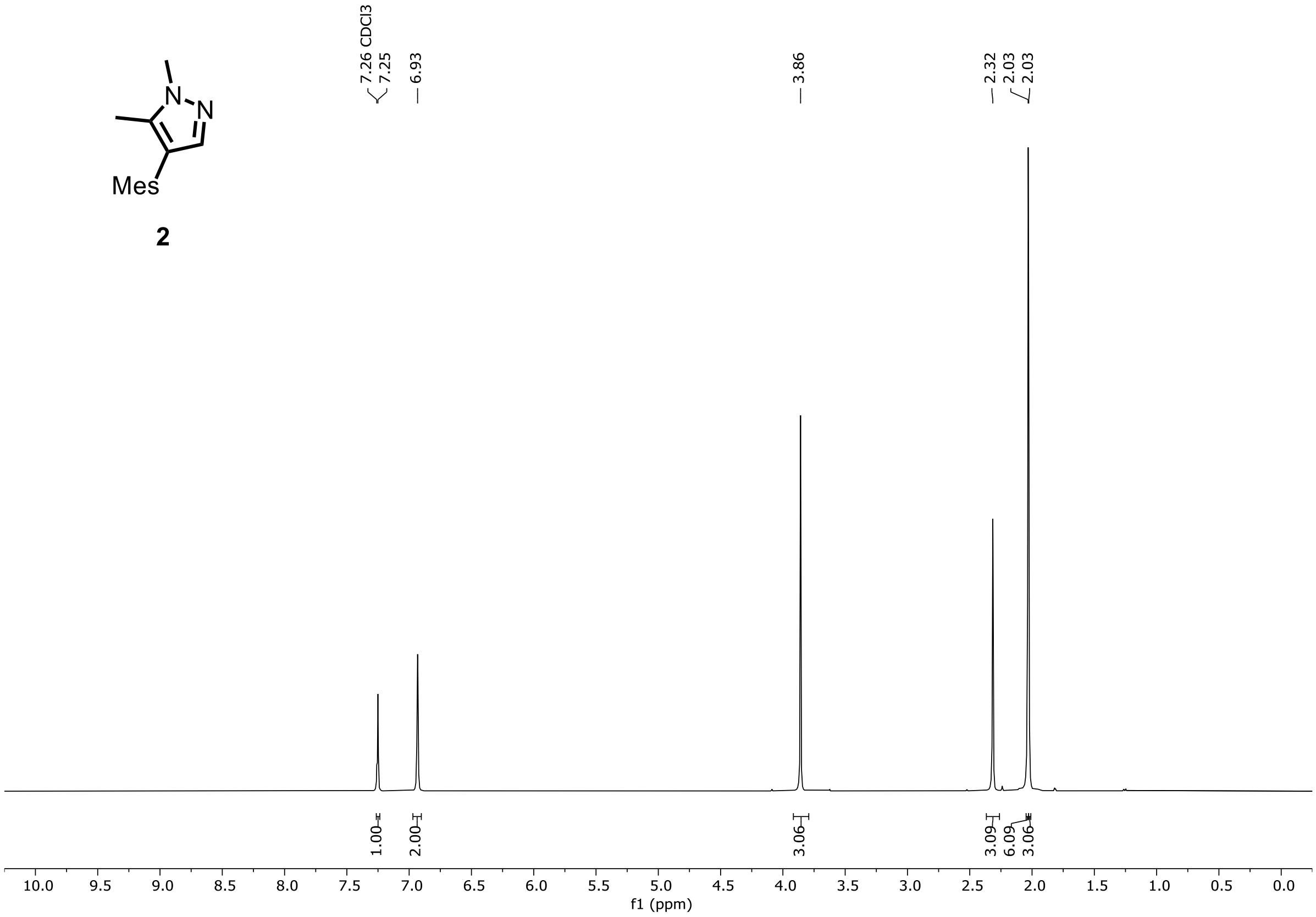

Figure S8: ^{1}H NMR spectrum of **2** (300 MHz, $CDCl_3$, 300 K).

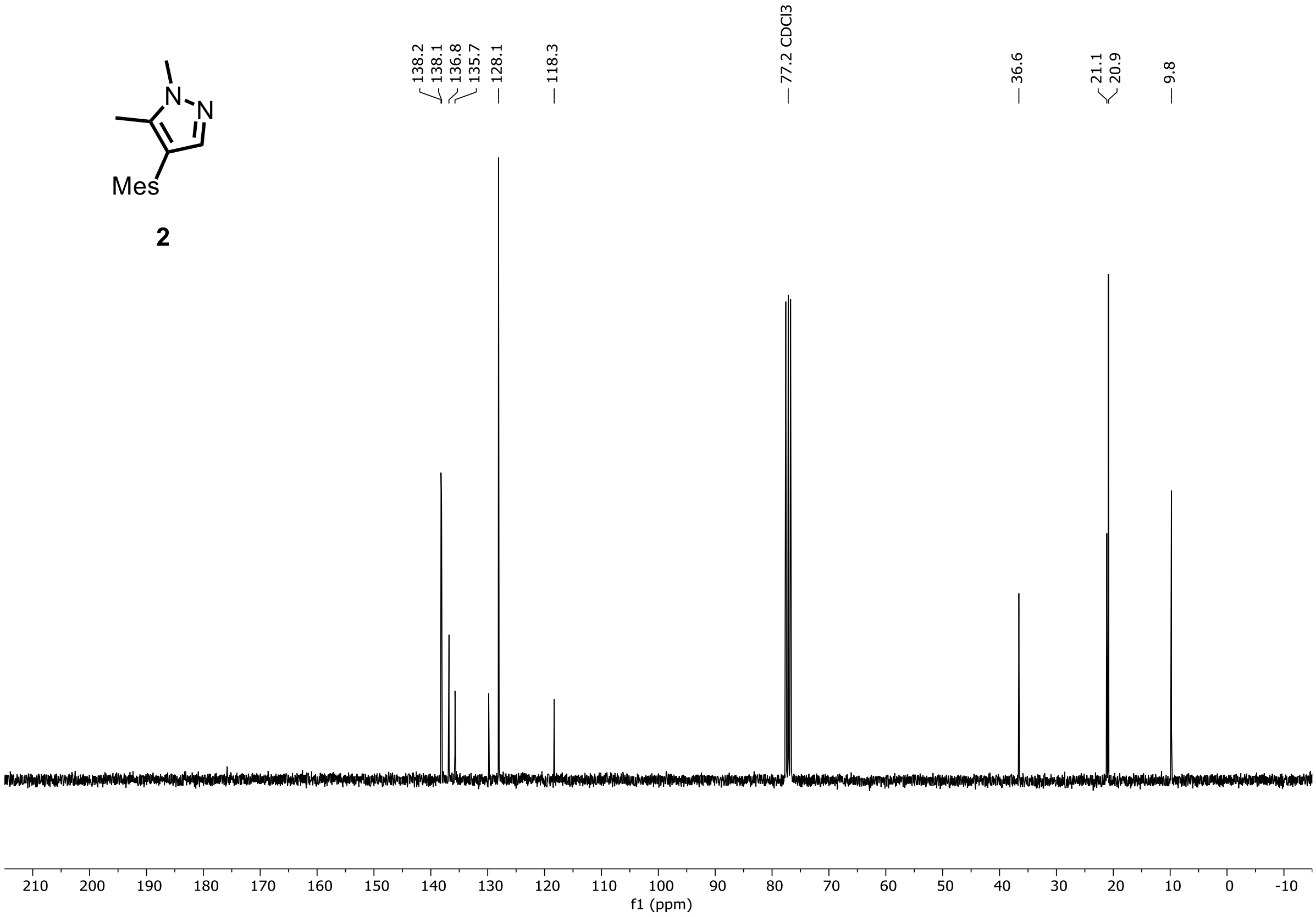

Figure S9: ^{13}C NMR spectrum of **2** (75 MHz, $CDCl_3$, 300 K).

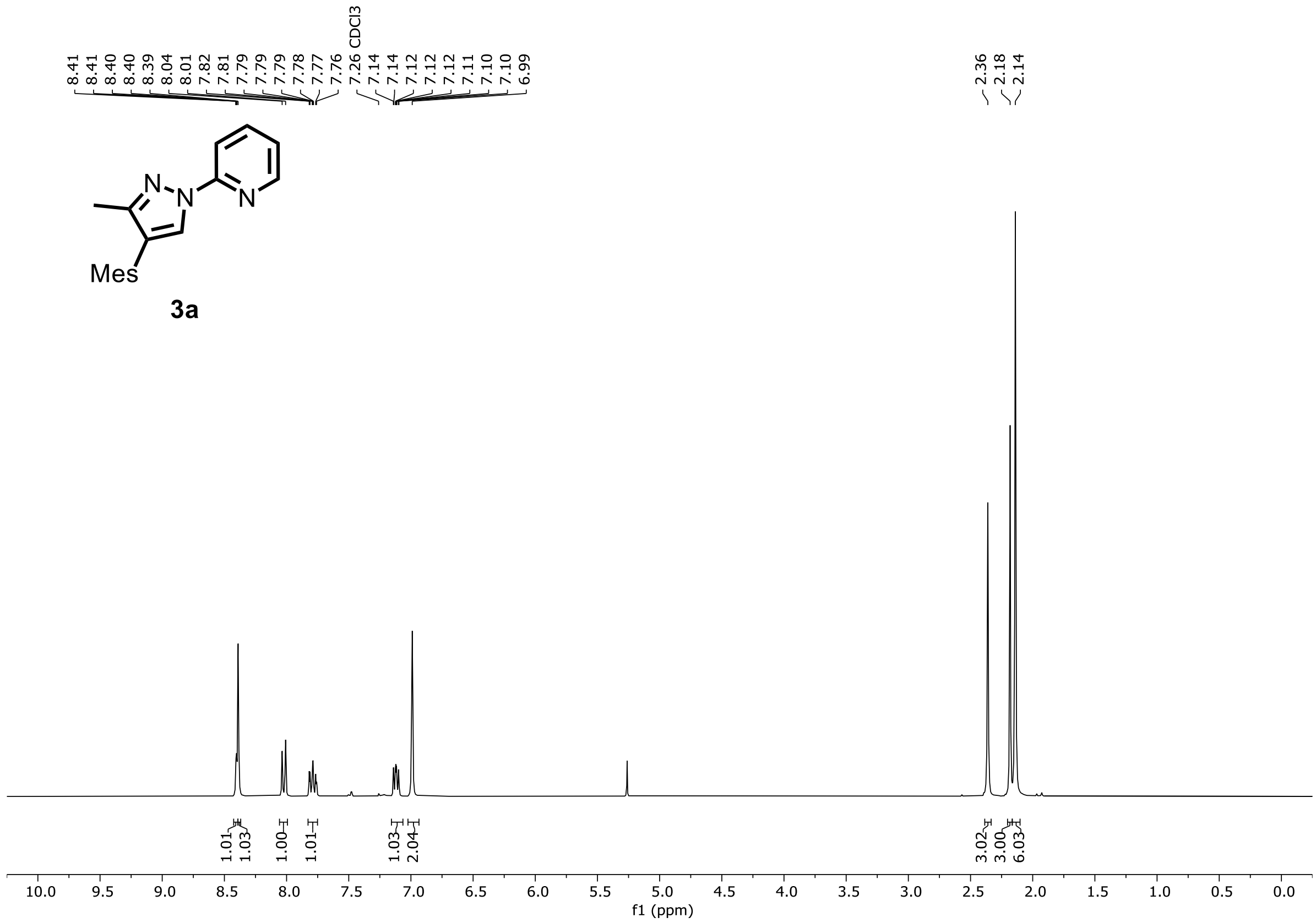

Figure S10: ^{1}H NMR spectrum of **3a** (300 MHz, $CDCl_3$, 300 K).

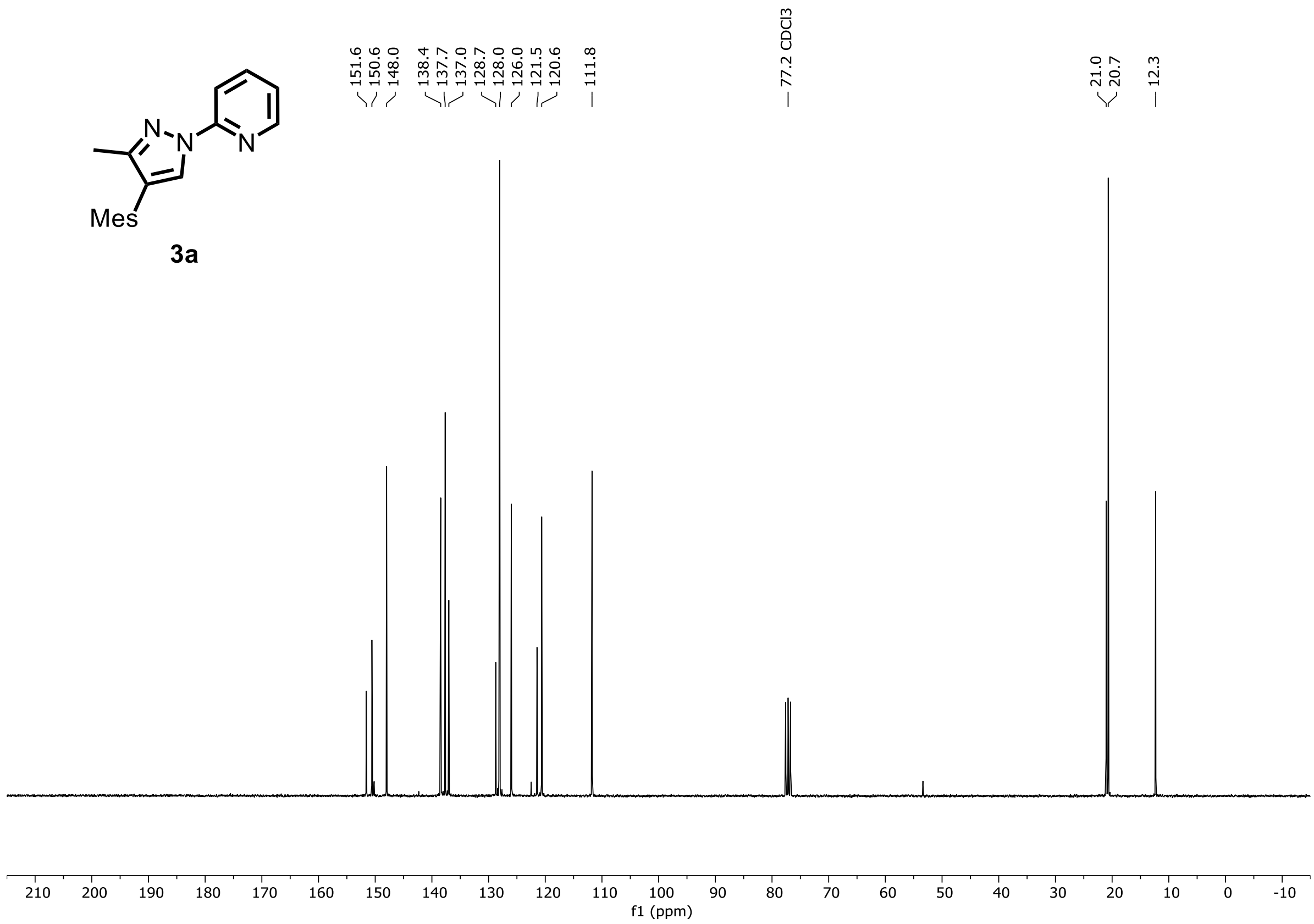

Figure S11: ^{13}C NMR spectrum of **3a** (75 MHz, $CDCl_3$, 300 K).

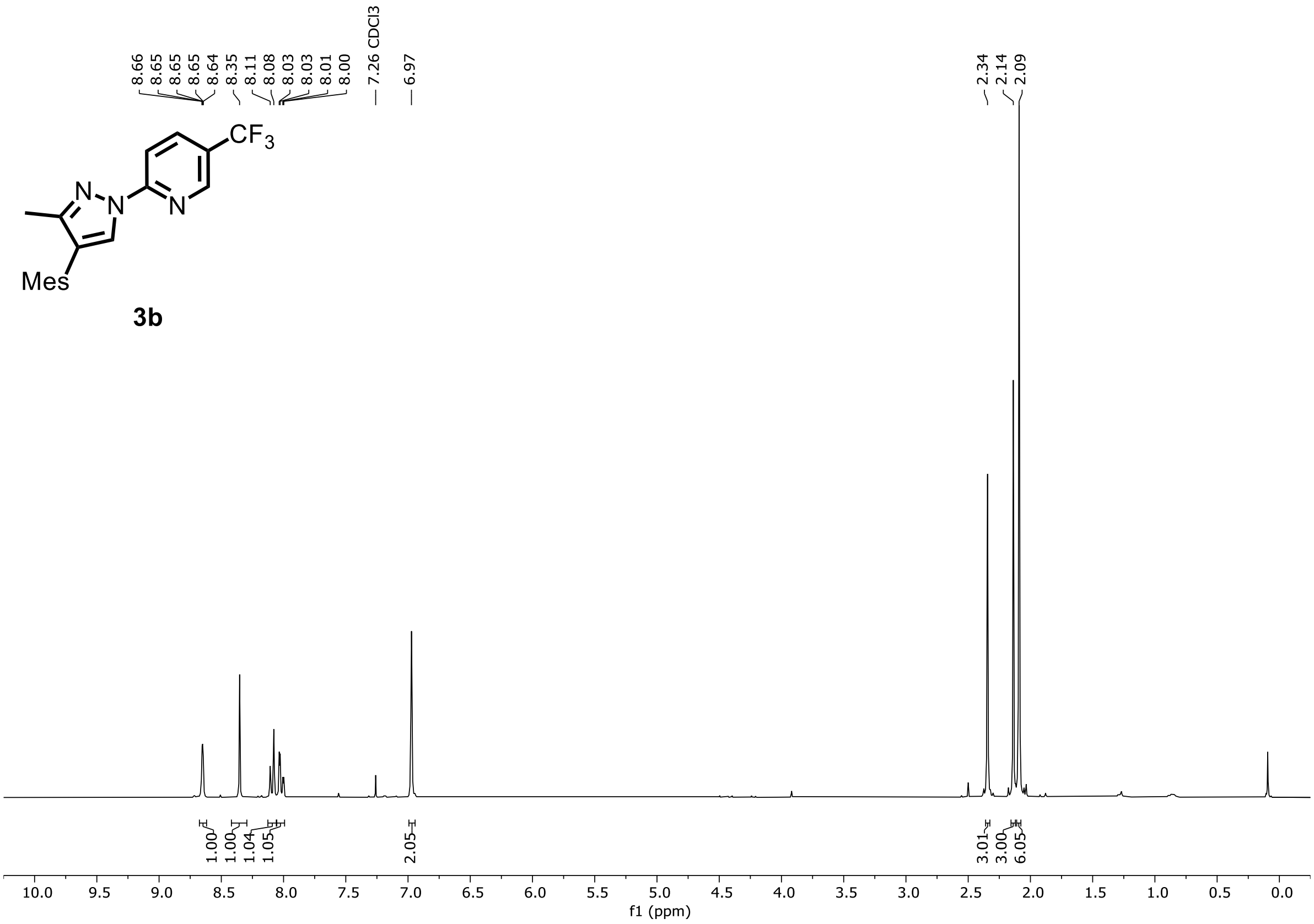

Figure S12: ^{1}H NMR spectrum of **3b** (300 MHz, $CDCl_3$, 300 K).

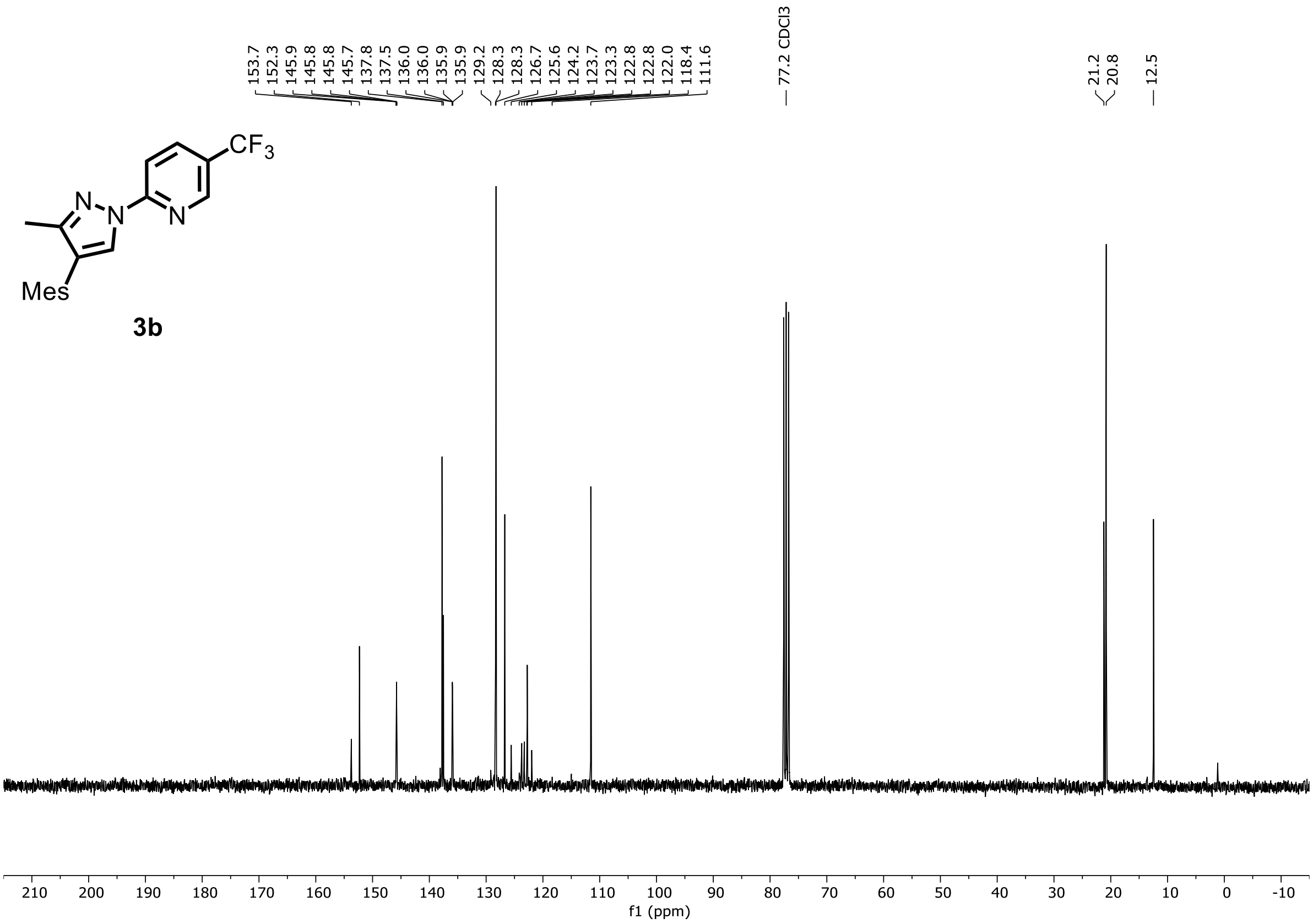

Figure S13: ^{13}C NMR spectrum of **3b** (75 MHz, $CDCl_3$, 300 K).

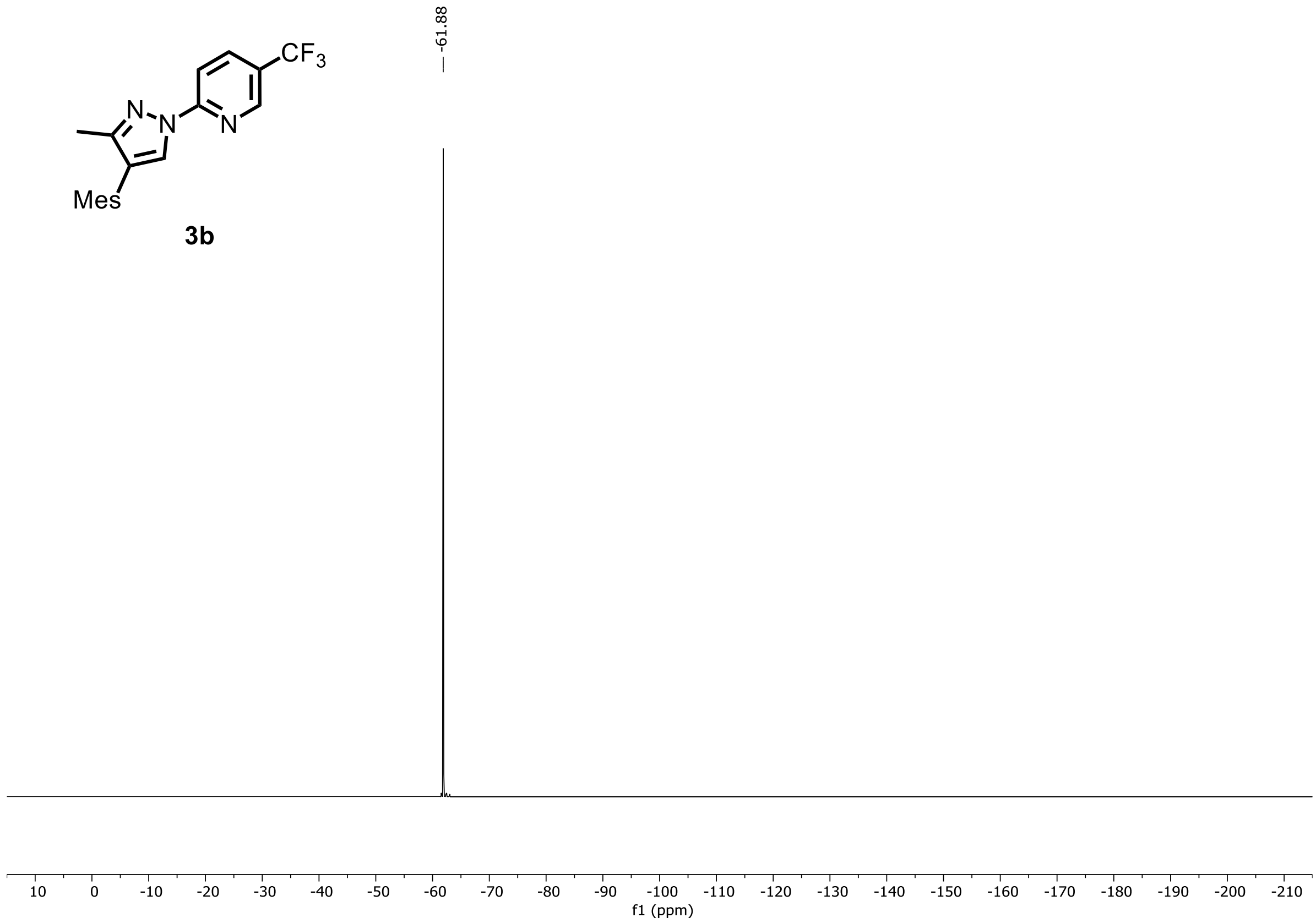

Figure S14: ^{19}F NMR spectrum of **3b** (282 MHz, $CDCl_3$, 300 K).

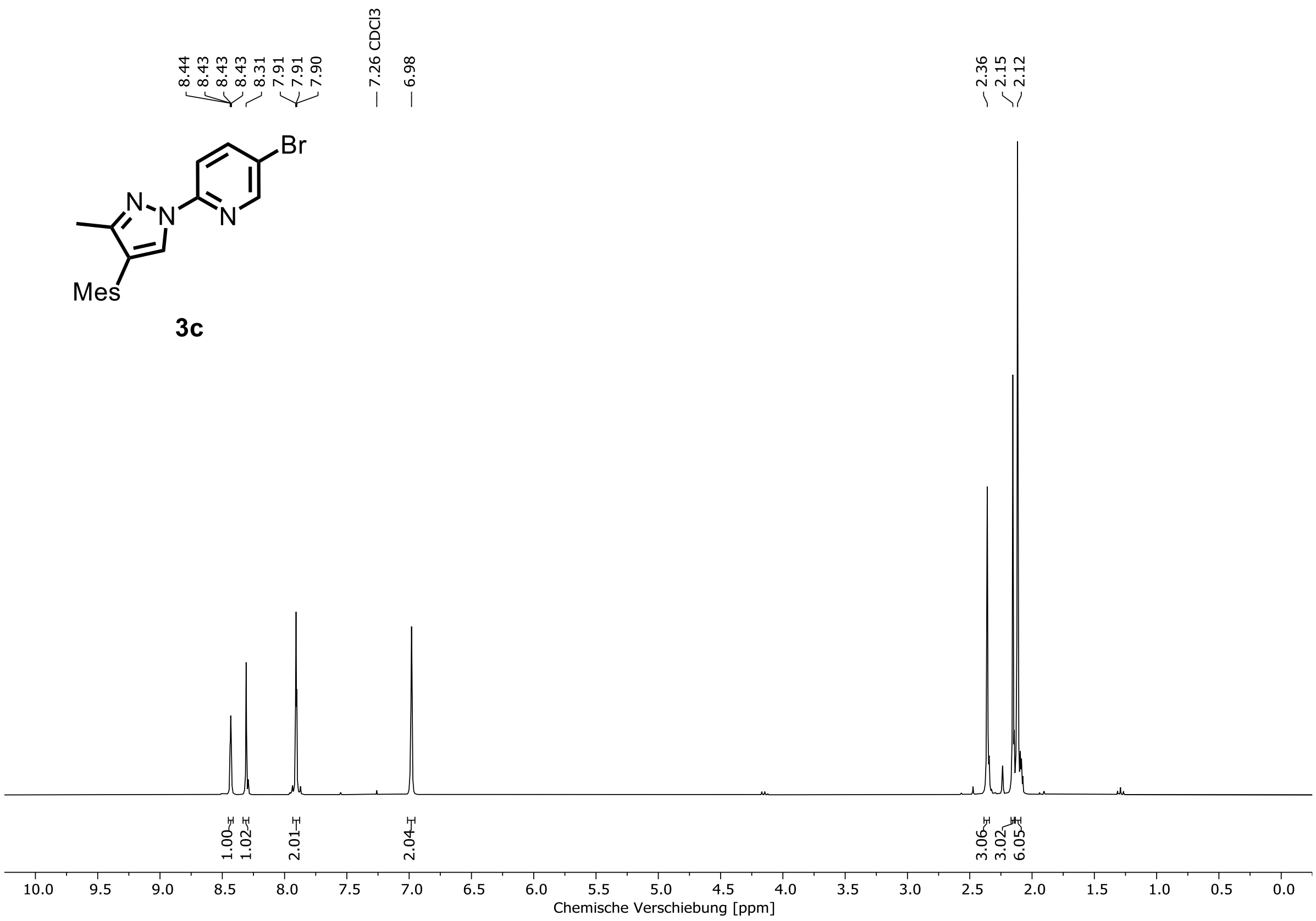

Figur S15: ^{1}H NMR spectrum of **3c** (300 MHz, $CDCl_3$, 300 K).

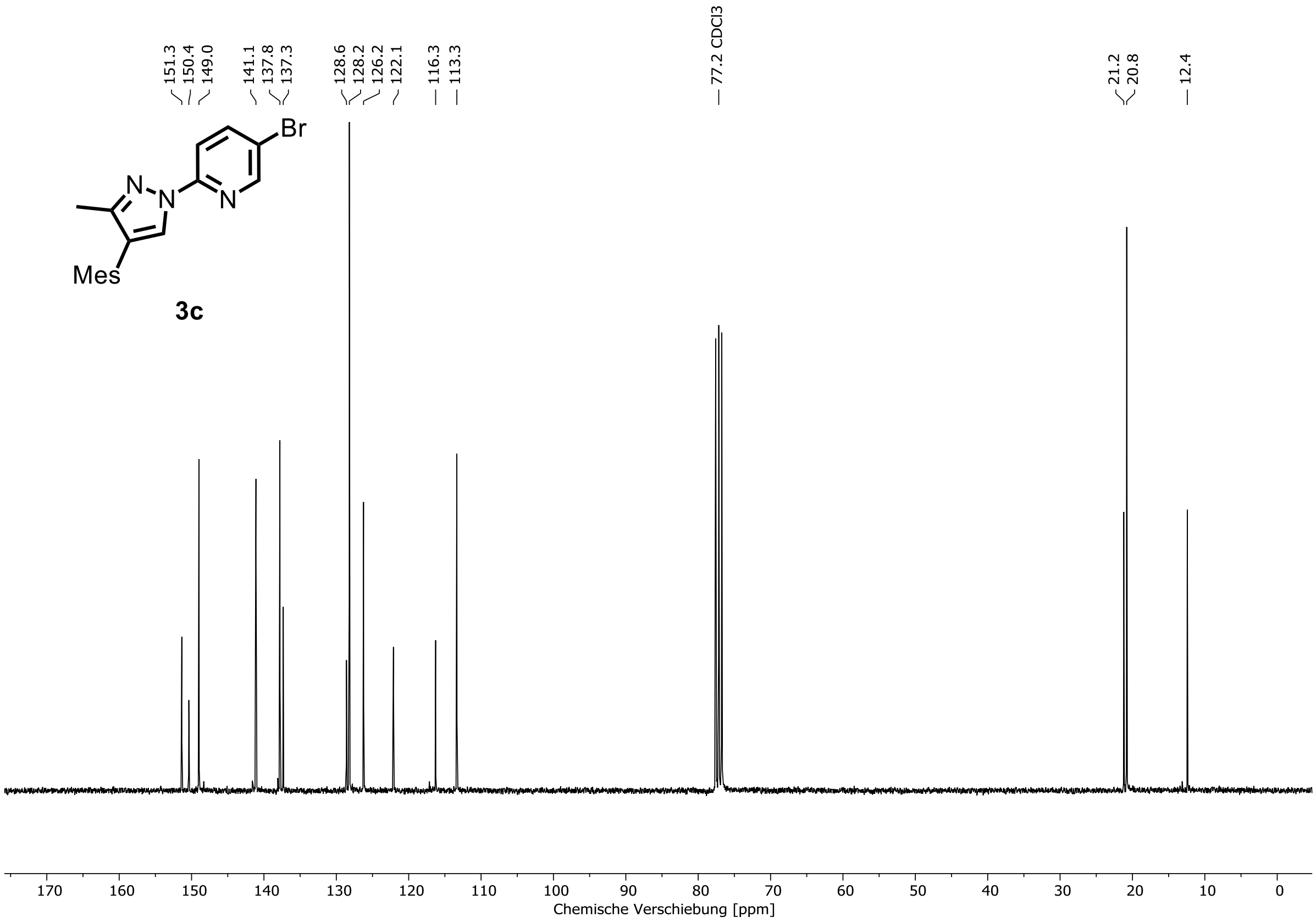

Figure S16: ^{13}C NMR spectrum of **3c** (75 MHz, $CDCl_3$, 300 K).

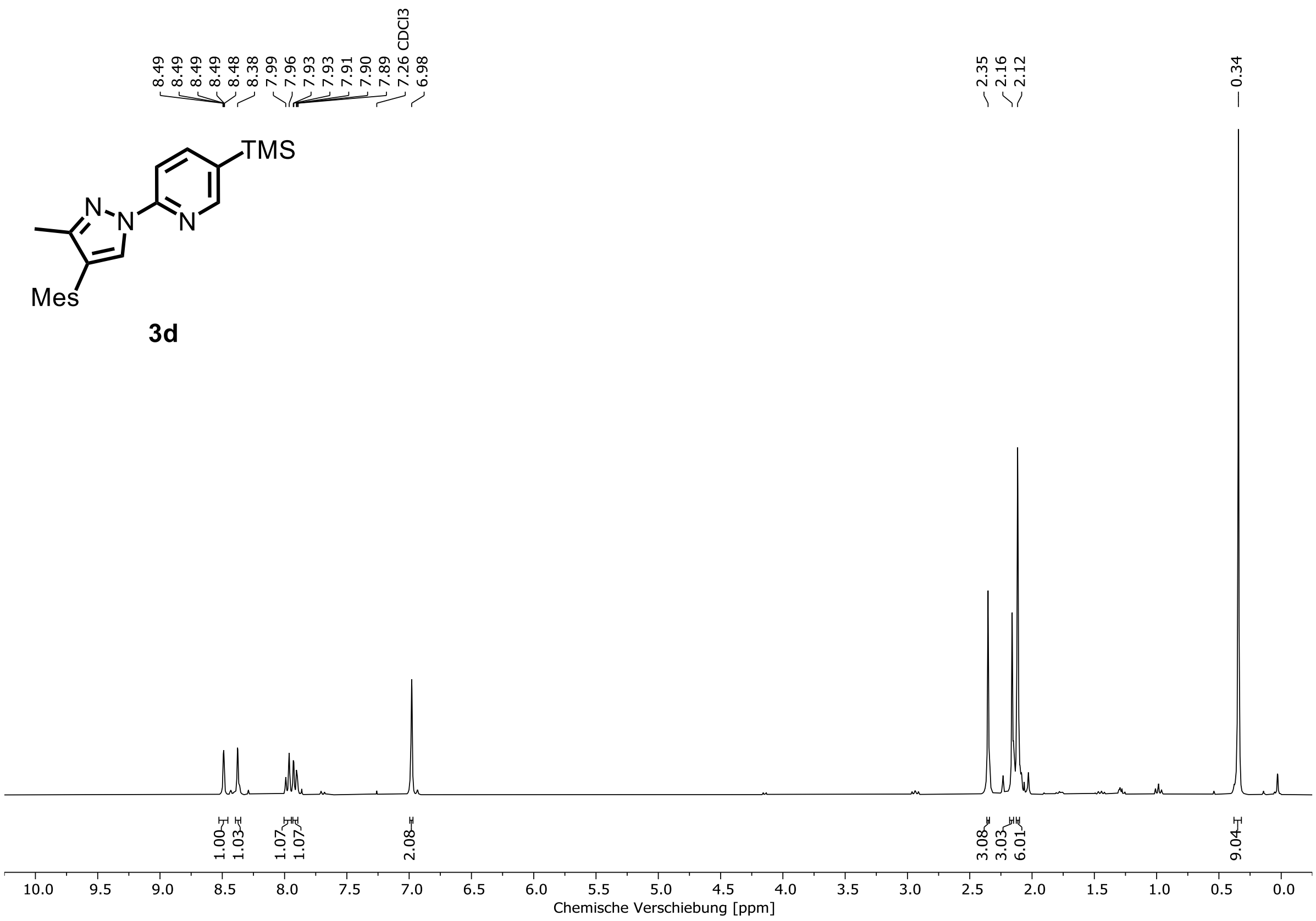

Figure S17: ^{1}H NMR spectrum of **3d** (300 MHz, $CDCl_3$, 300 K).

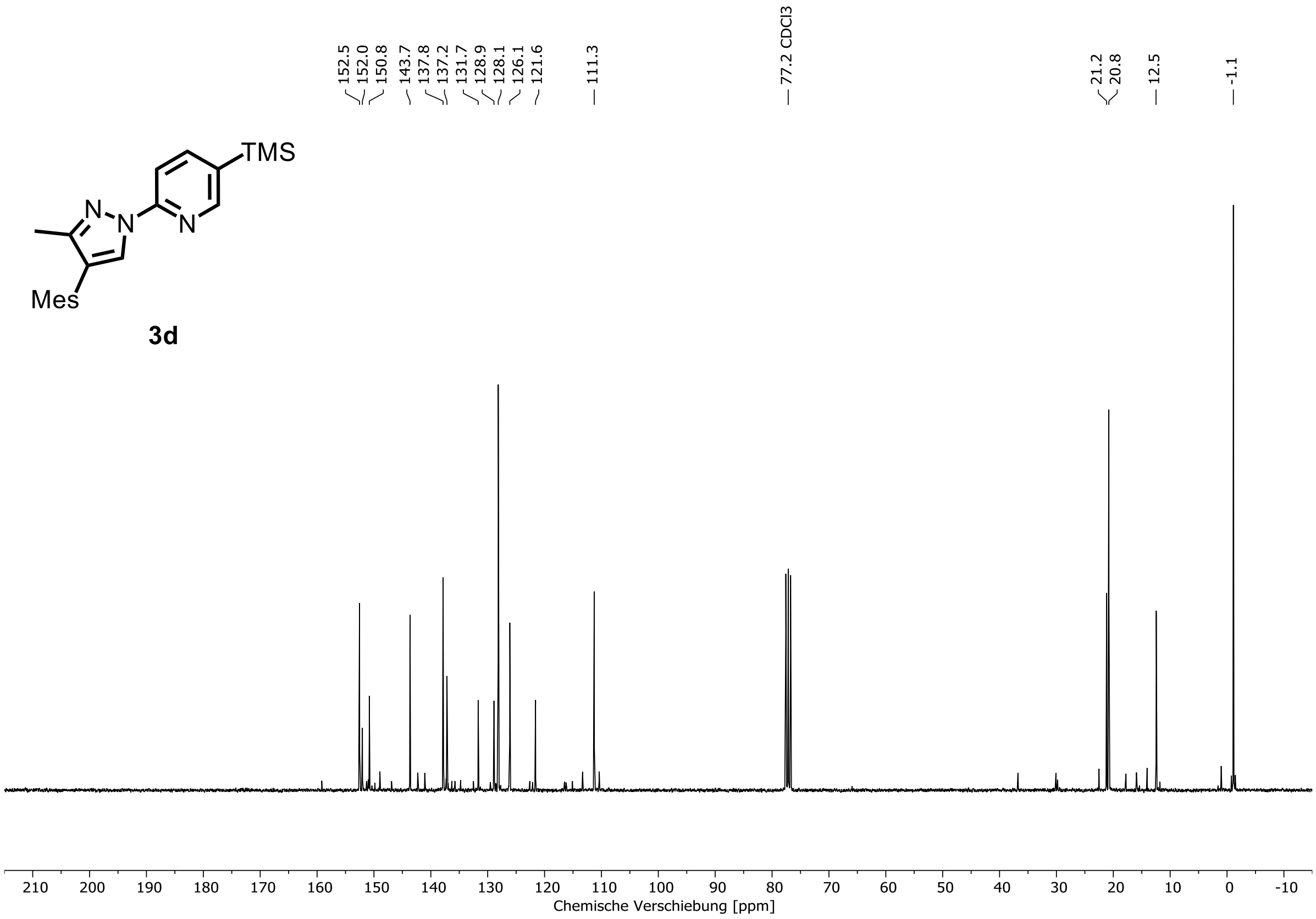

Figure S18: ^{13}C NMR spectrum of **3d** (75 MHz, $CDCl_3$, 300 K).

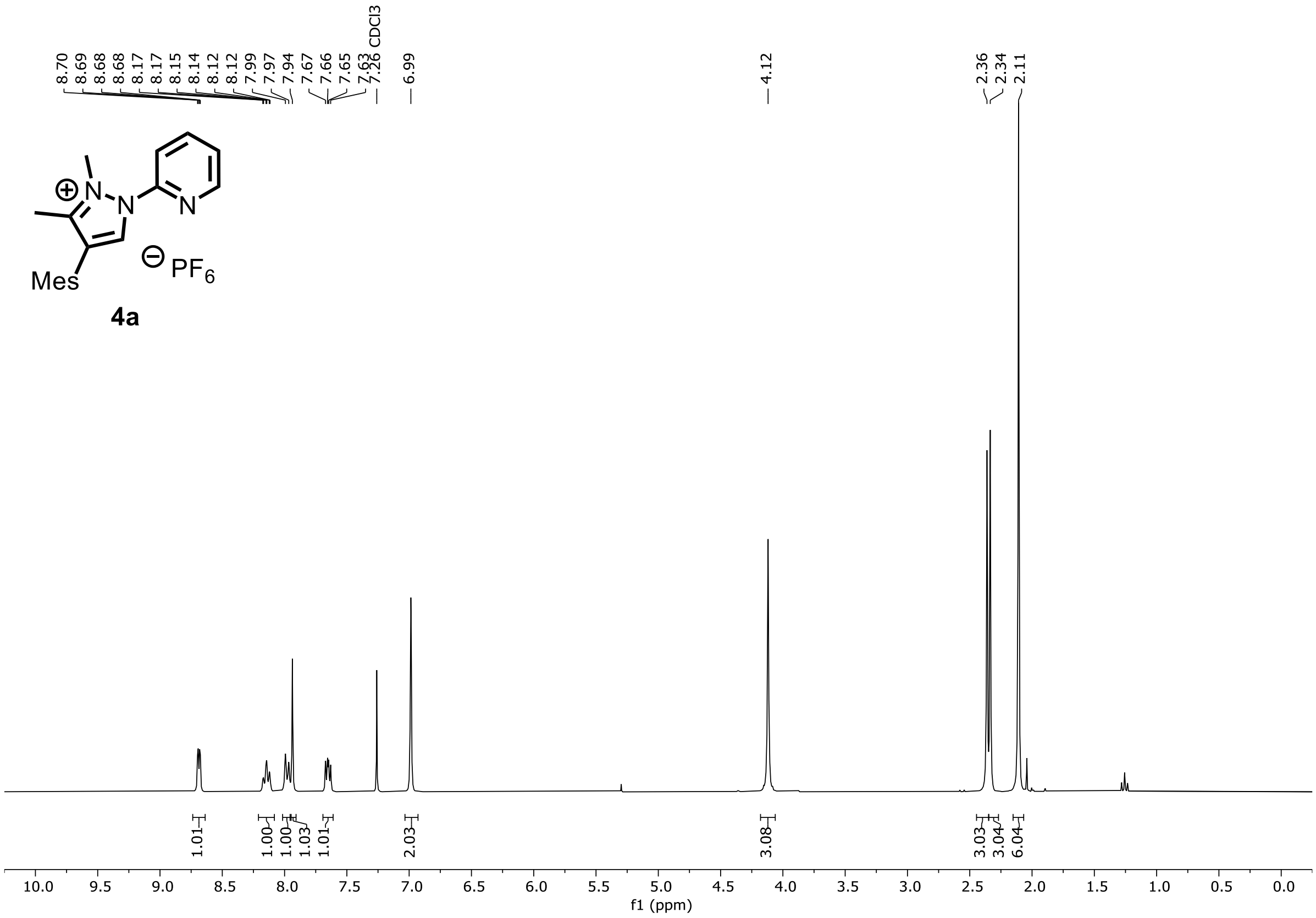

Figure S19: ^{1}H NMR spectrum of **4a** (300 MHz, $CDCl_3$, 300 K).

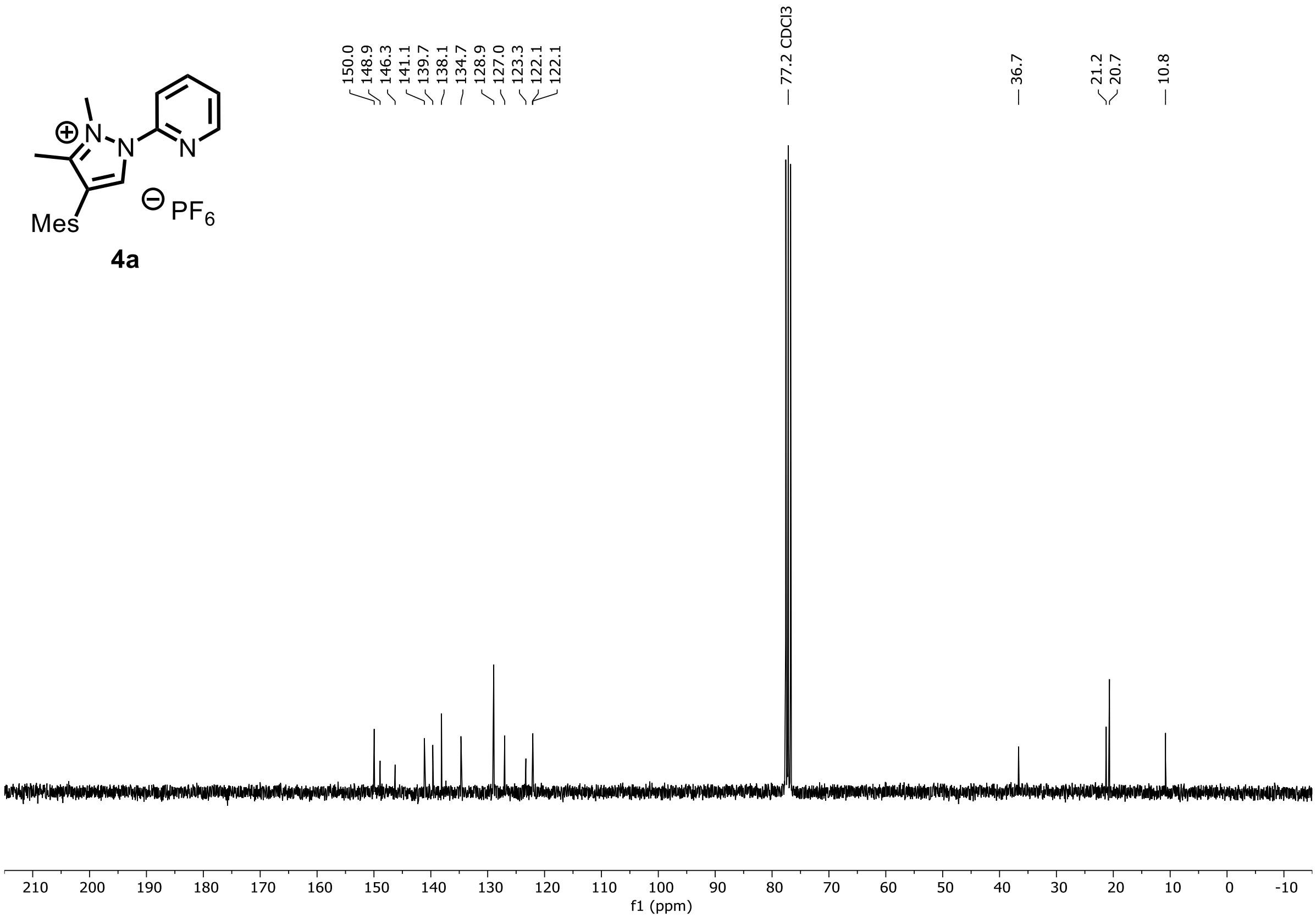

Figure S20: ^{13}C NMR spectrum of **4a** (75 MHz, $CDCl_3$, 300 K).

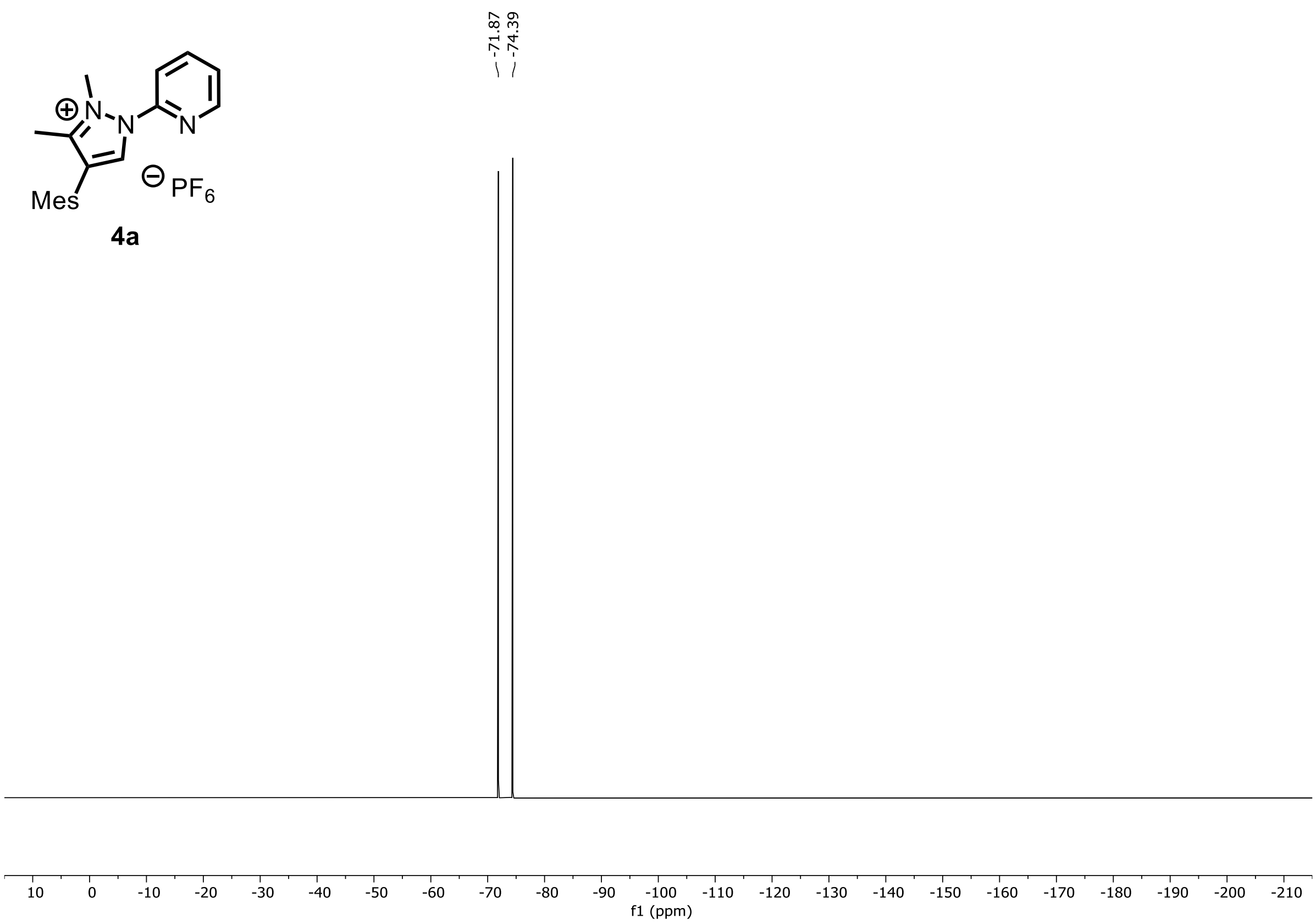

Figure S21: ^{19}F NMR spectrum of **4a** (282 MHz, $CDCl_3$, 300 K).

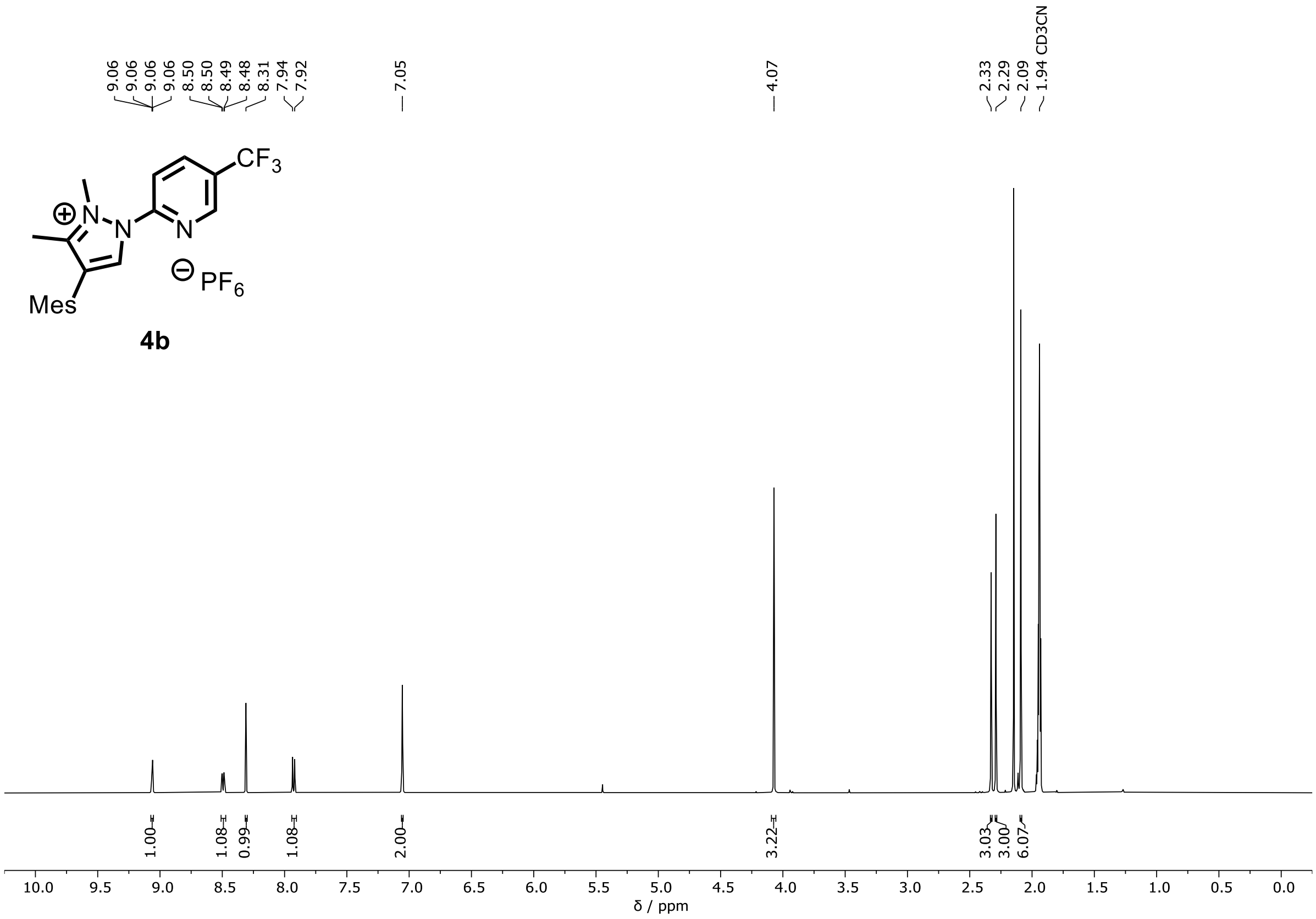

Figure S22: ^{1}H NMR spectrum of **4b** (500 MHz, CD_3CN, 300 K).

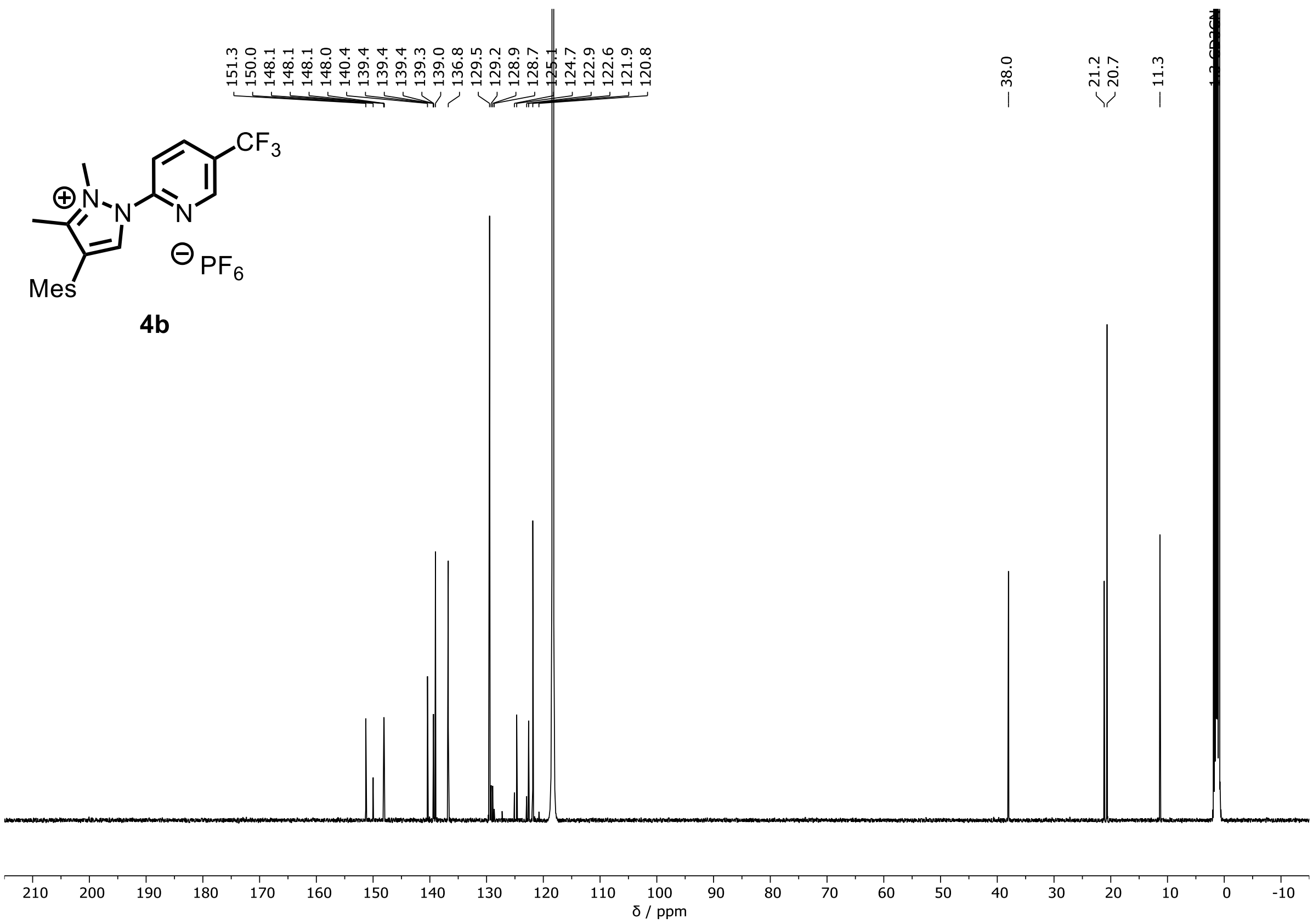

Figure S23: ^{13}C NMR spectrum of **4b** (126 MHz, CD_3CN, 300 K).

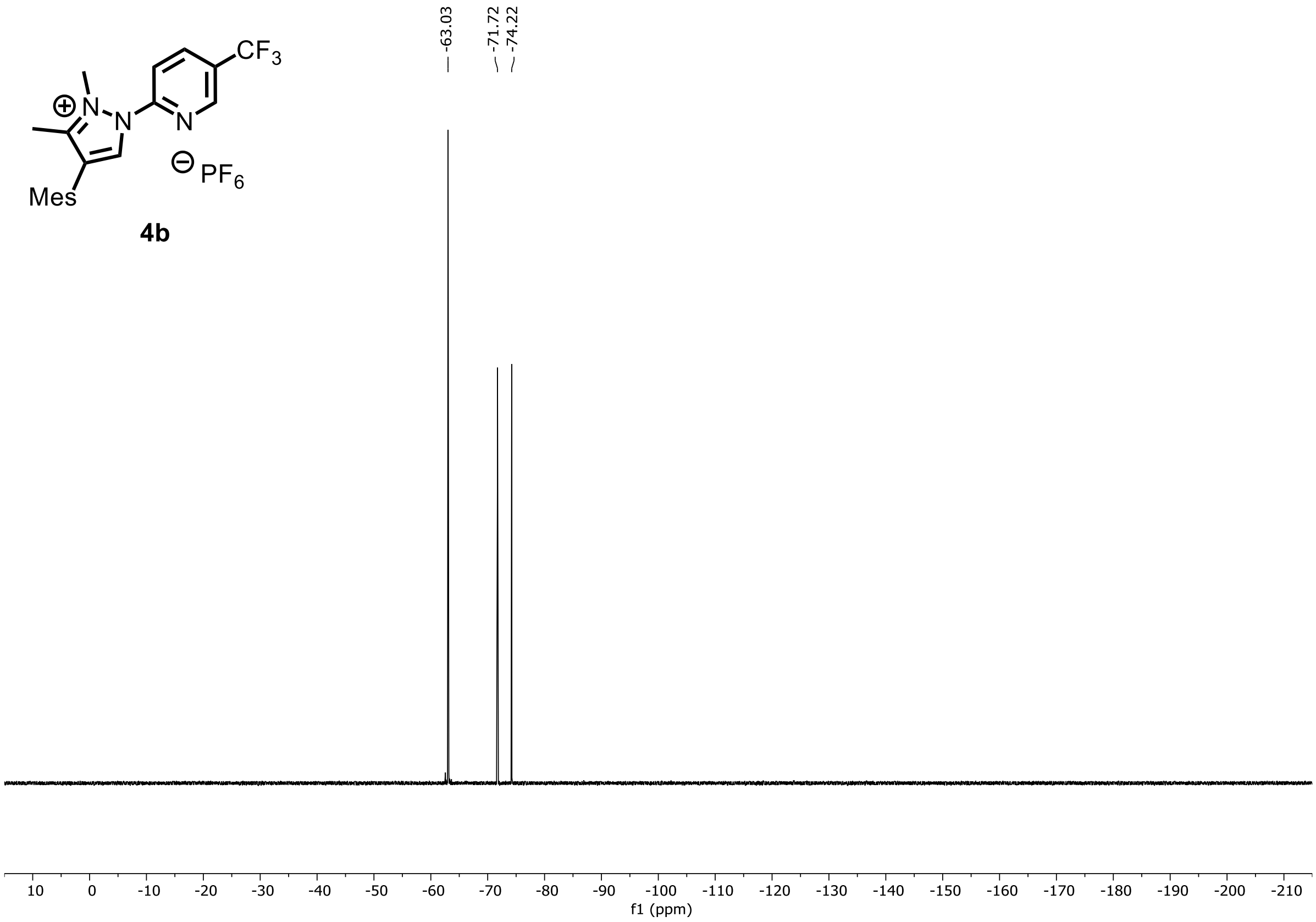

Figure S24: ^{19}F NMR spectrum of **4b** (282 MHz, CD_3CN, 300 K).

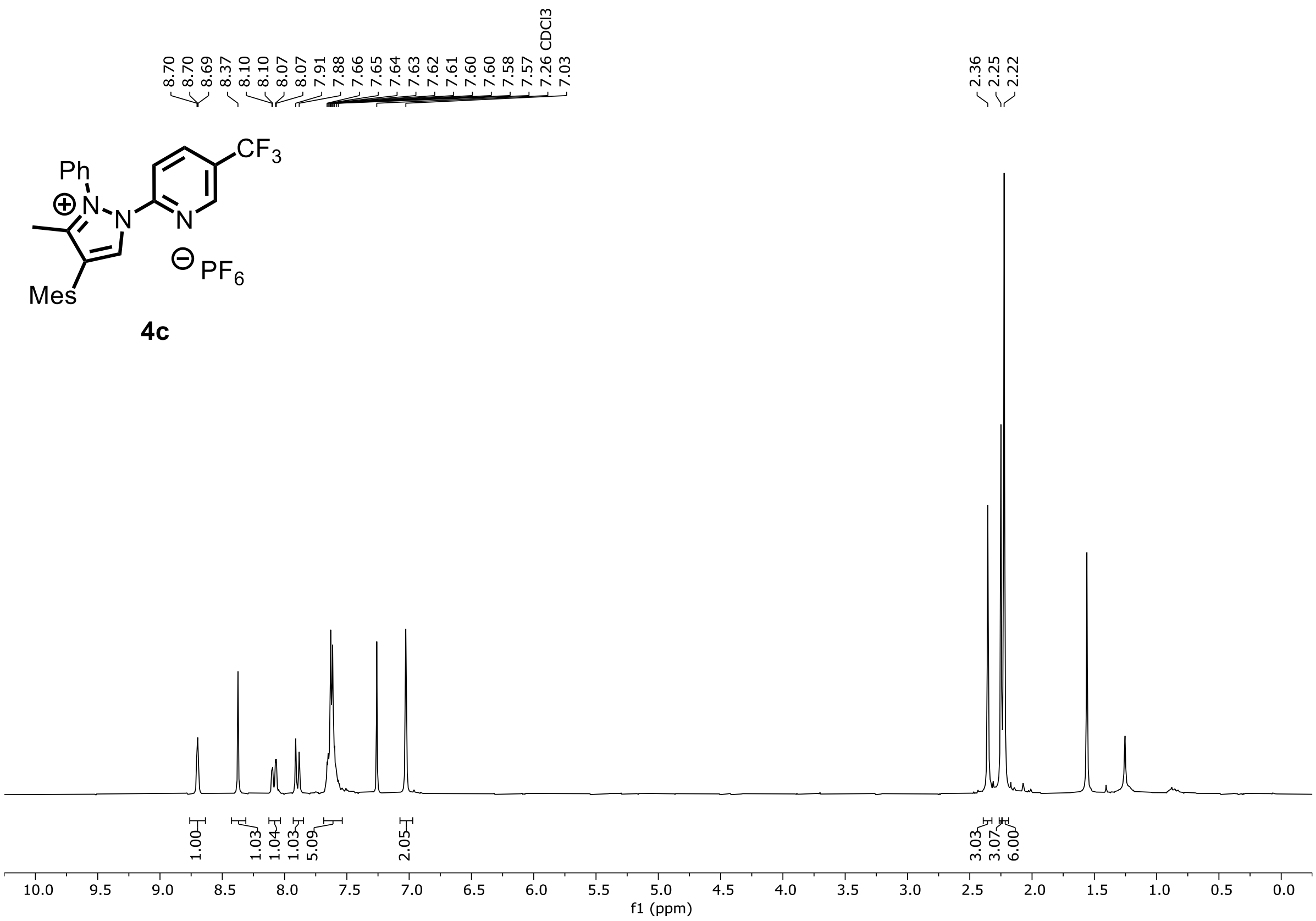

Figure S25: ^{1}H NMR spectrum of **4c** (300 MHz, $CDCl_3$, 300 K).

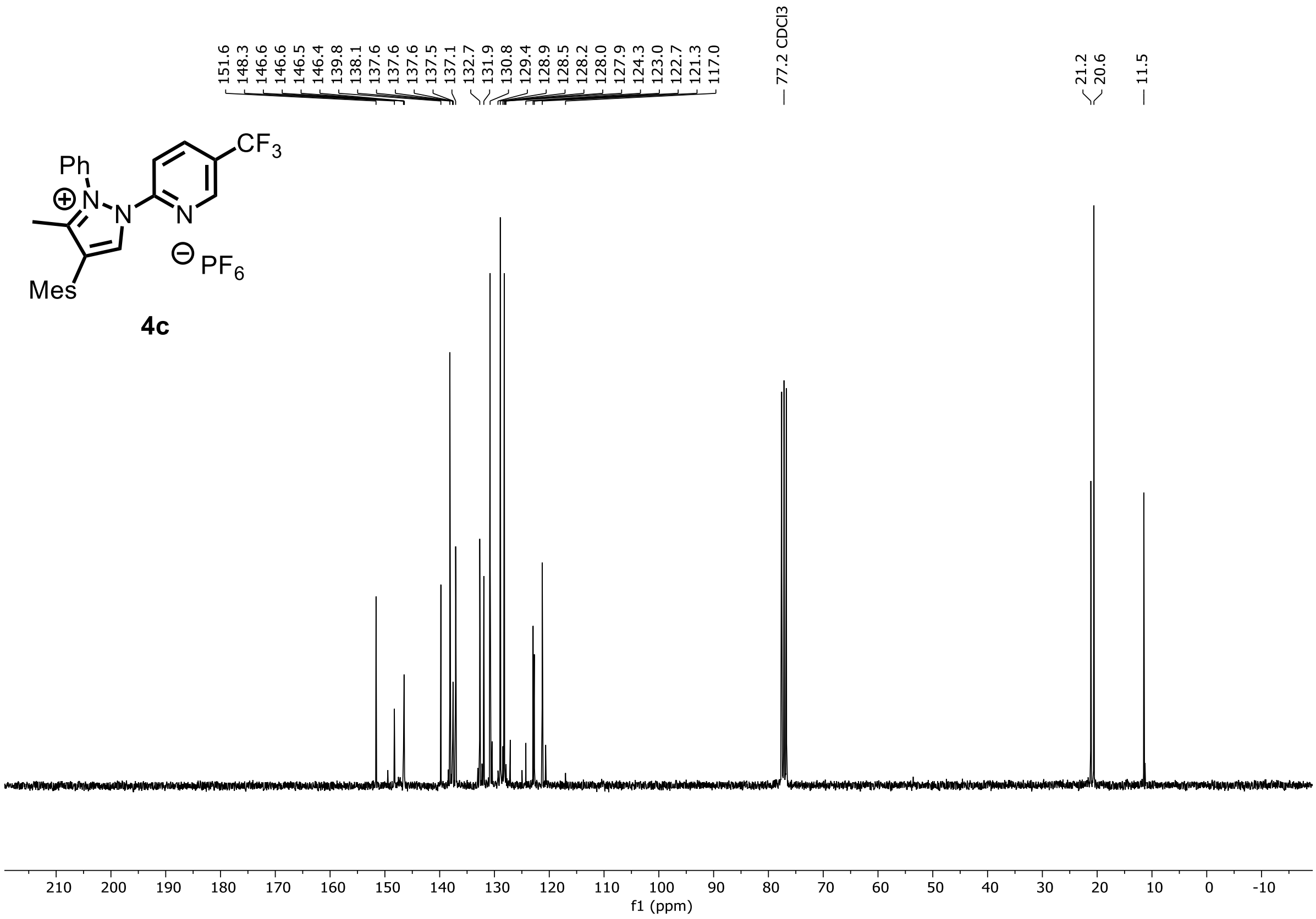

Figure S26: ^{13}C NMR spectrum of **4c** (75 MHz, $CDCl_3$, 300 K).

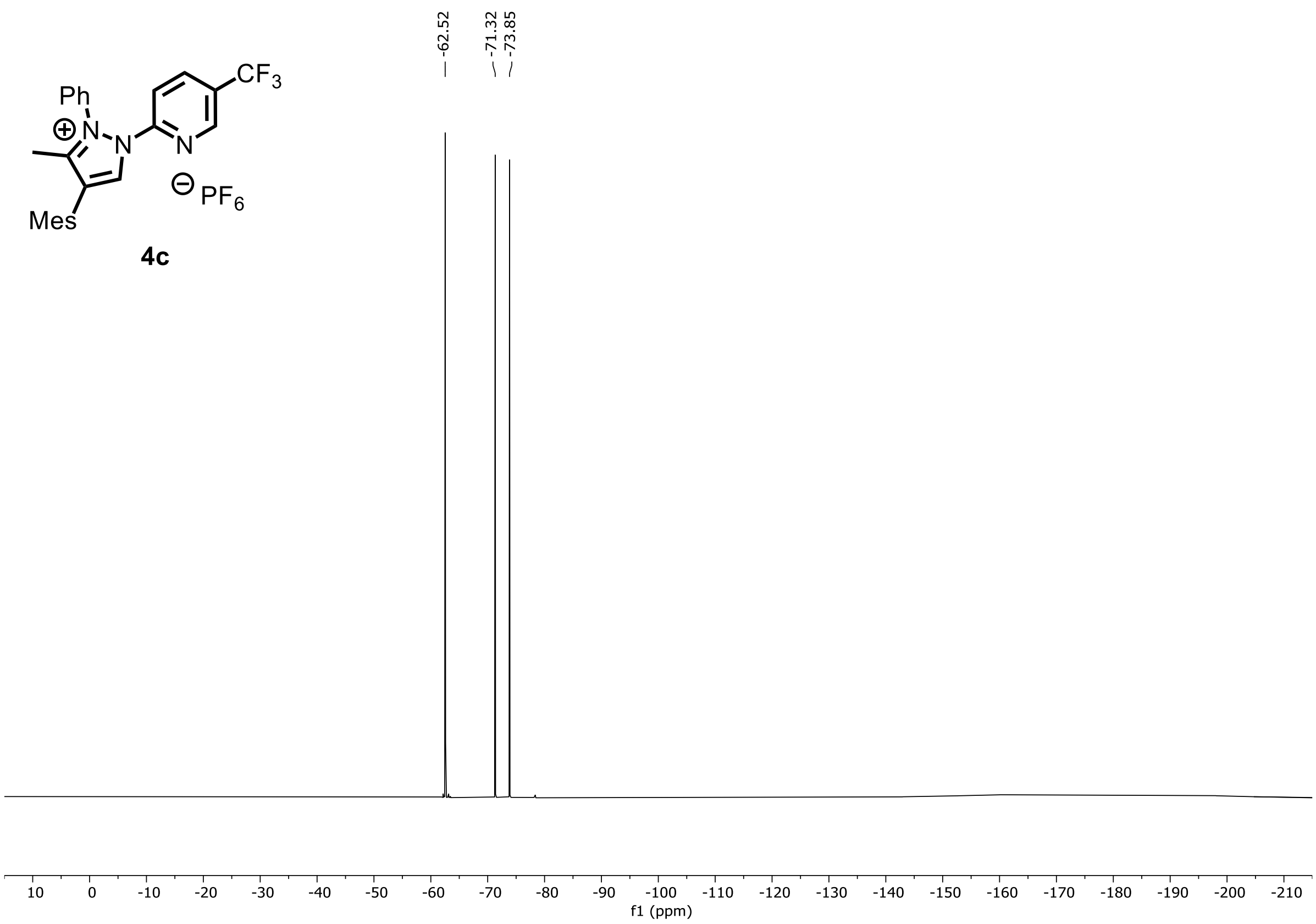

Figure S27: ^{19}F NMR spectrum of **4c** (282 MHz, $CDCl_3$, 300 K).

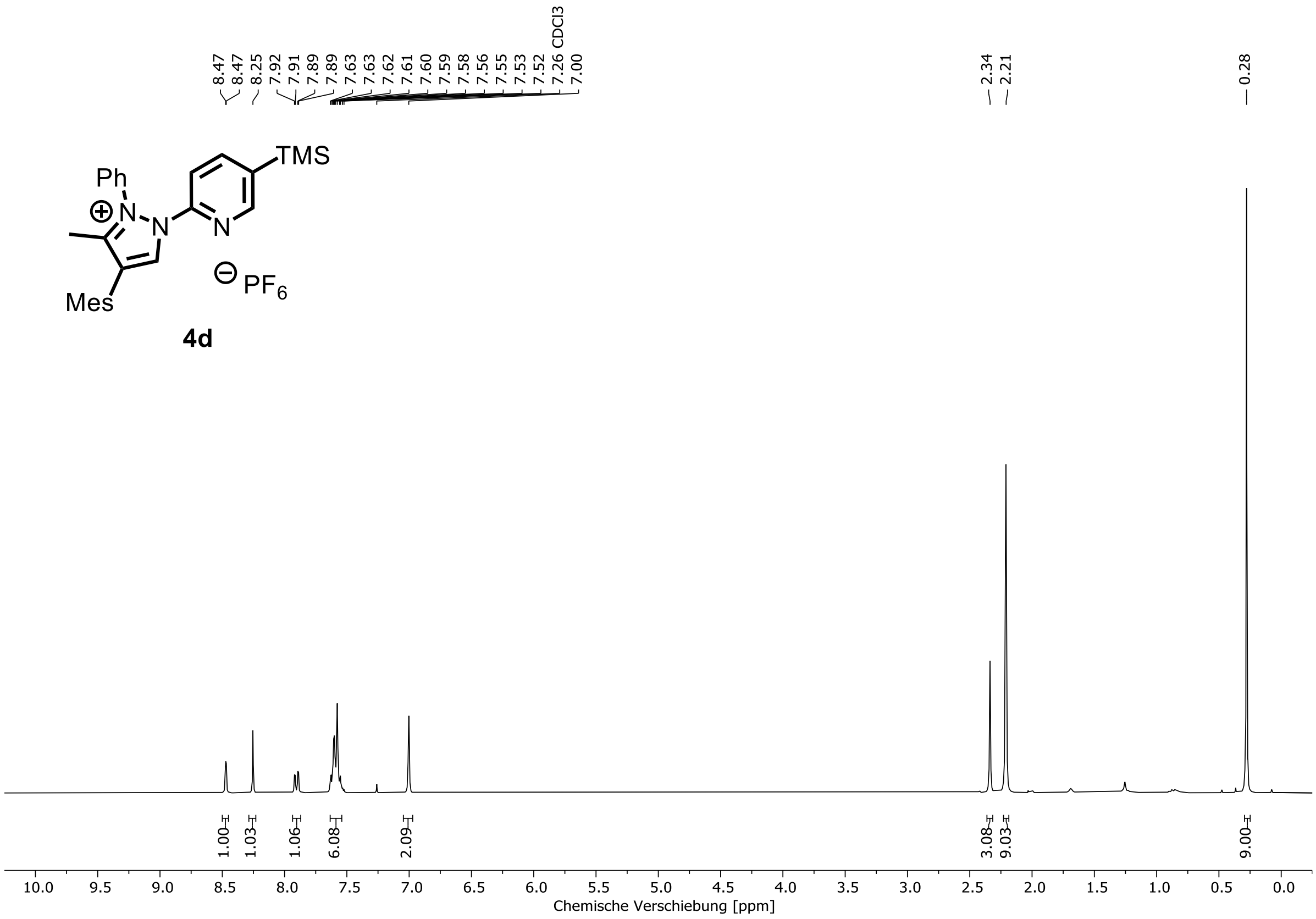

Figure S28: ^{1}H NMR spectrum of **4d** (300 MHz, $CDCl_3$, 300 K).

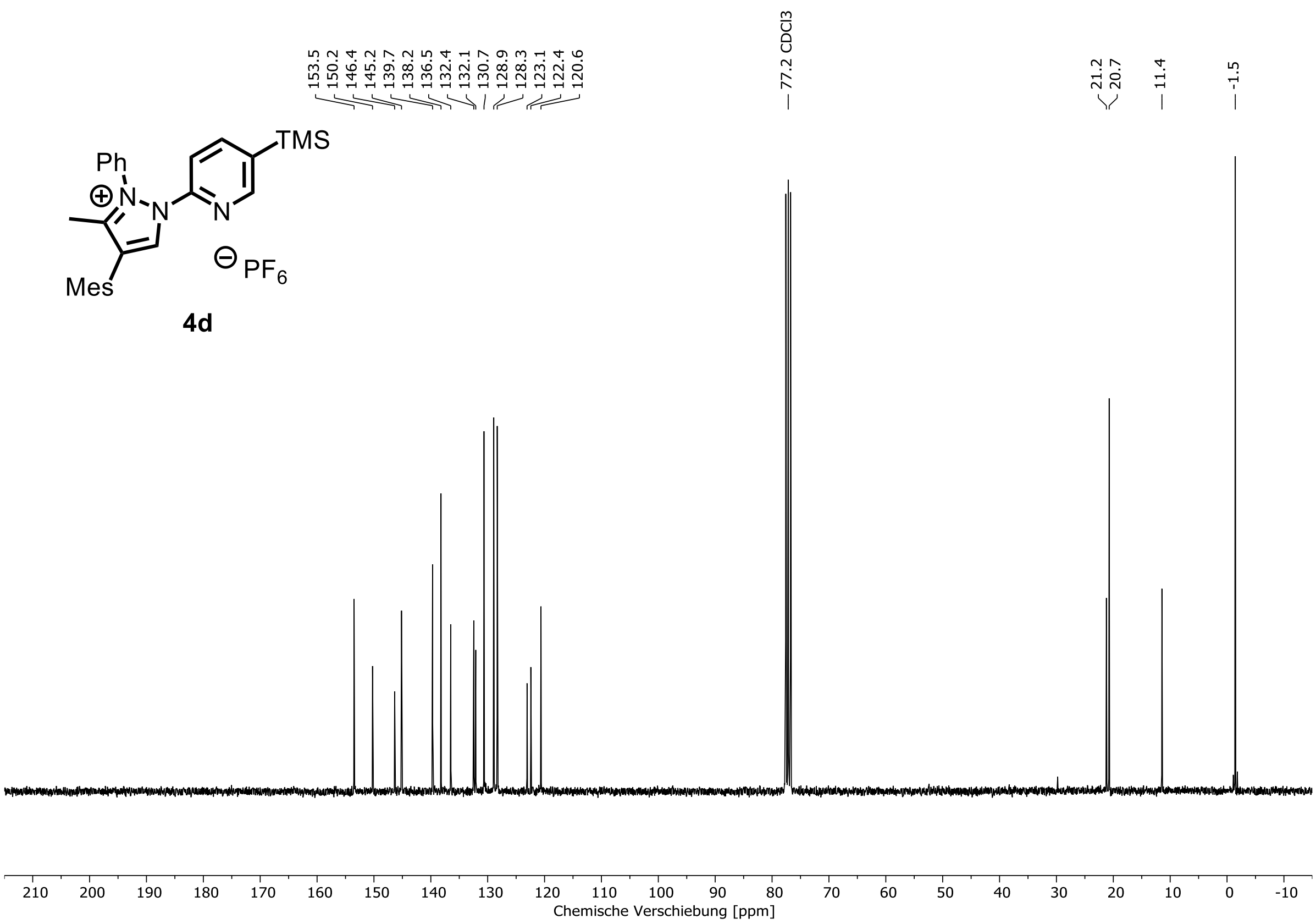

Figure S29: ^{13}C NMR spectrum of **4d** (75 MHz, $CDCl_3$, 300 K).

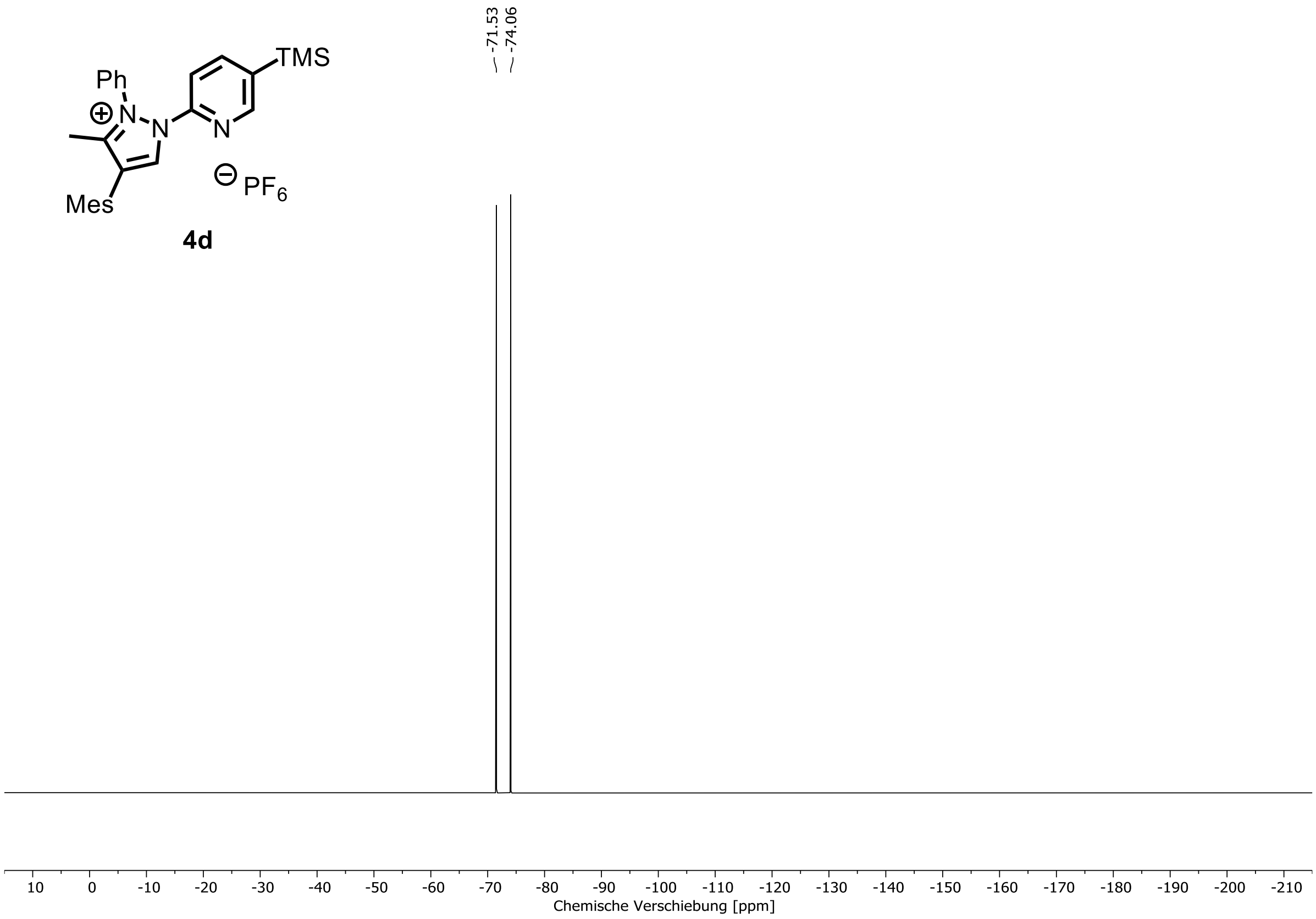

Figure S30: ^{19}F NMR spectrum of **4d** (282 MHz, $CDCl_3$, 300 K).

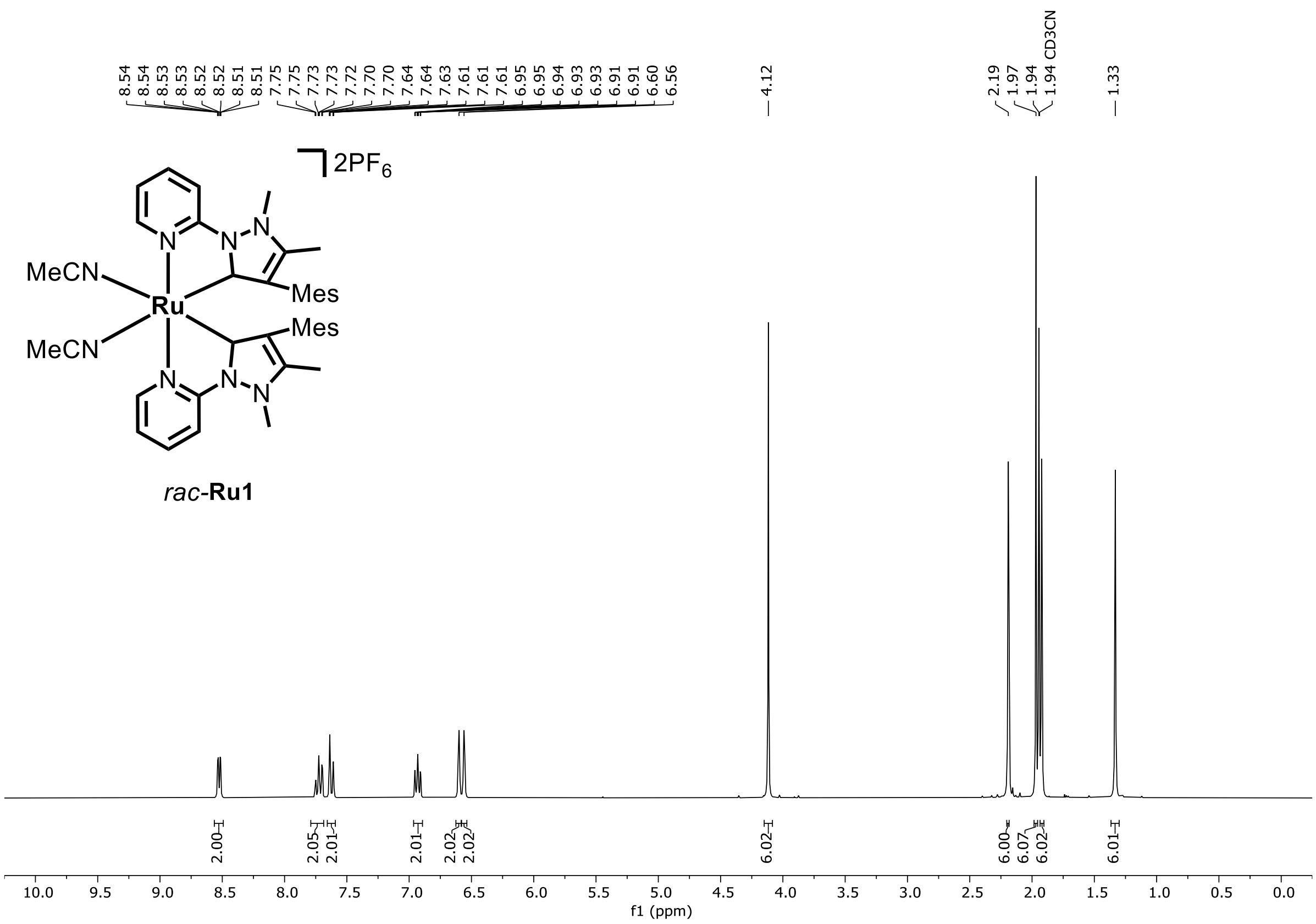

Figure S31: ^{1}H NMR spectrum of *rac*-**Ru1** (300 MHz, CD_3CN, 300 K).

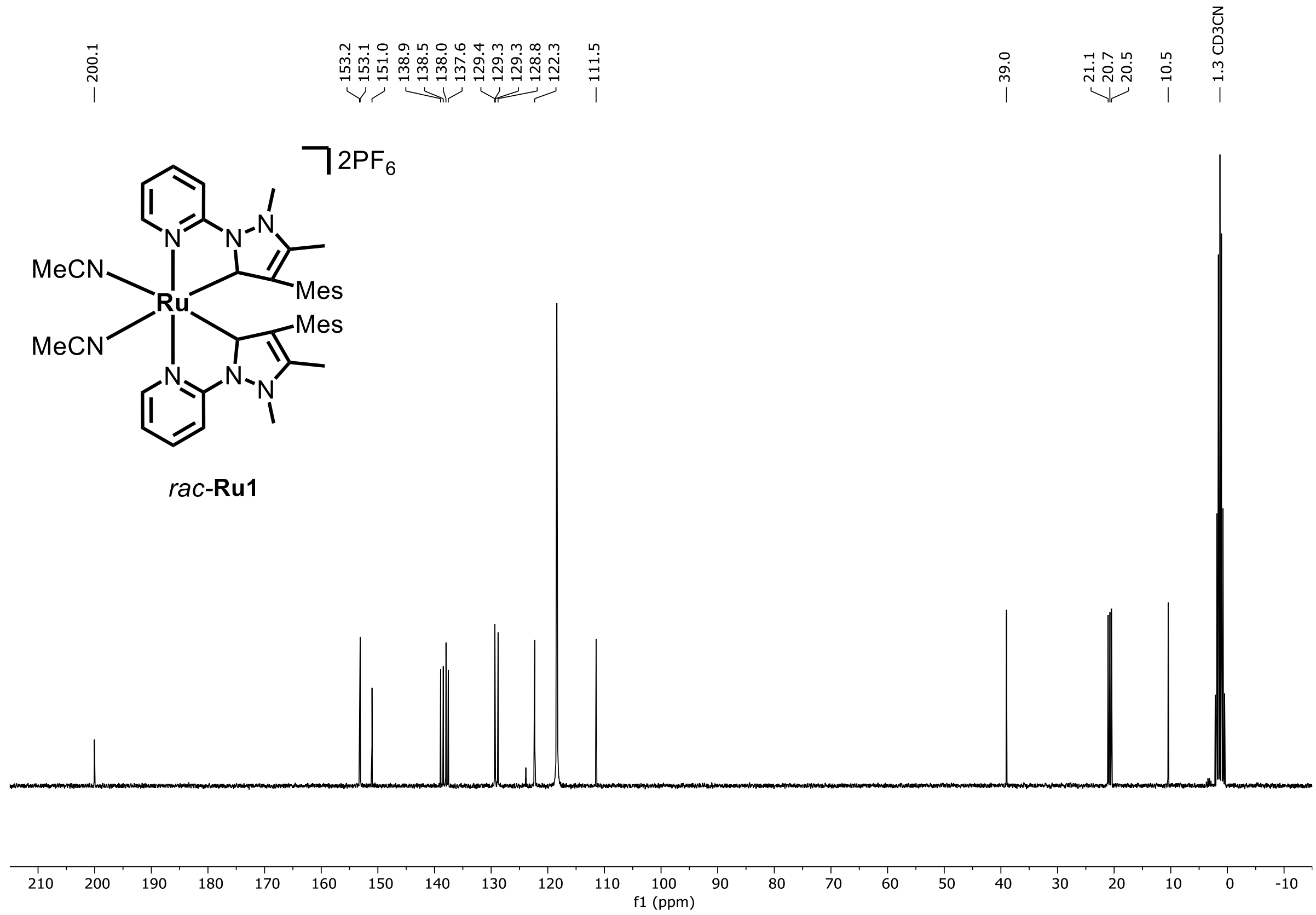

Figure S32: ^{13}C NMR spectrum of *rac*-**Ru1** (75 MHz, CD_3CN, 300 K).

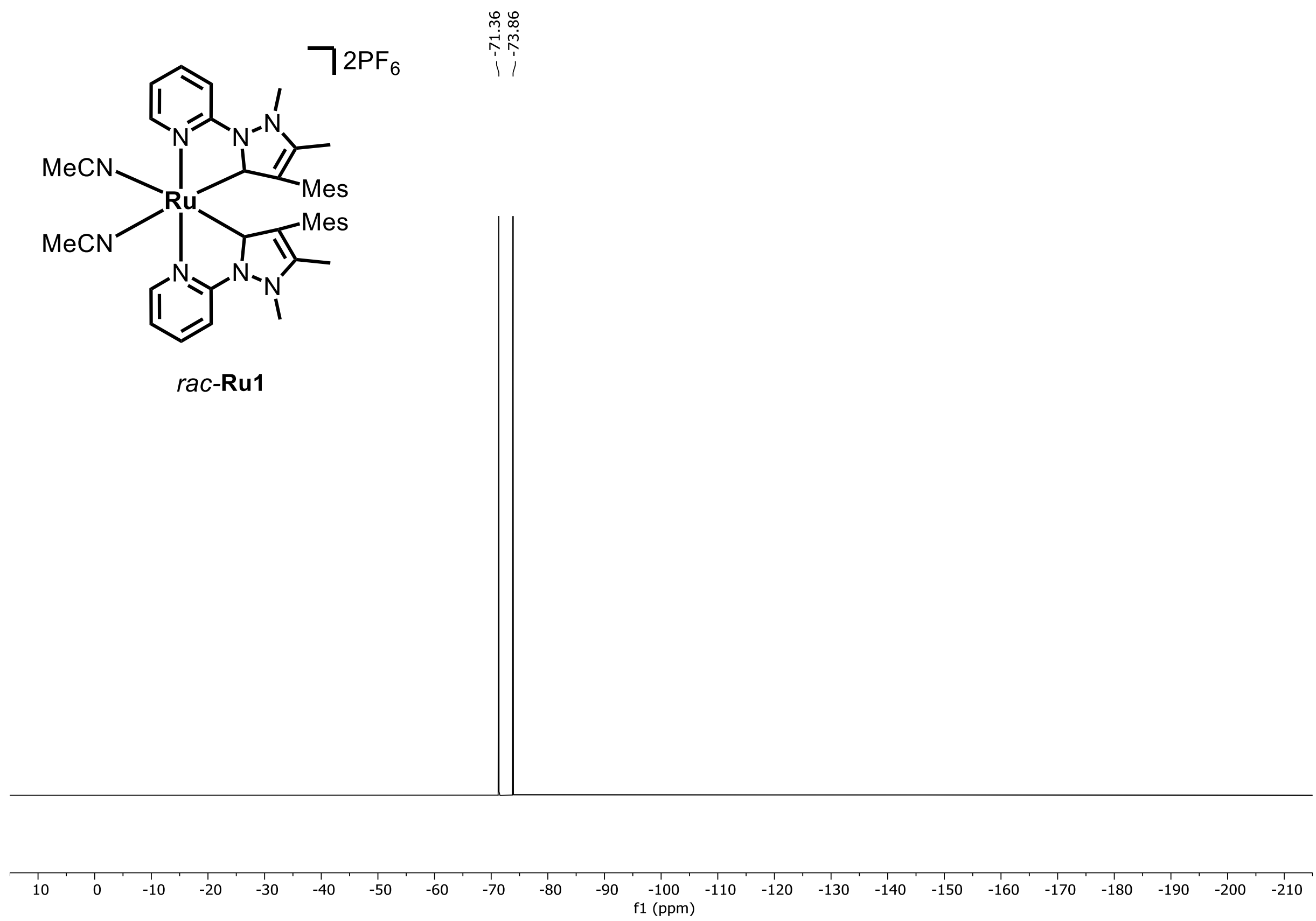

Figure S33: ^{19}F NMR spectrum of *rac*-**Ru1** (282 MHz, CD_3CN, 300 K).

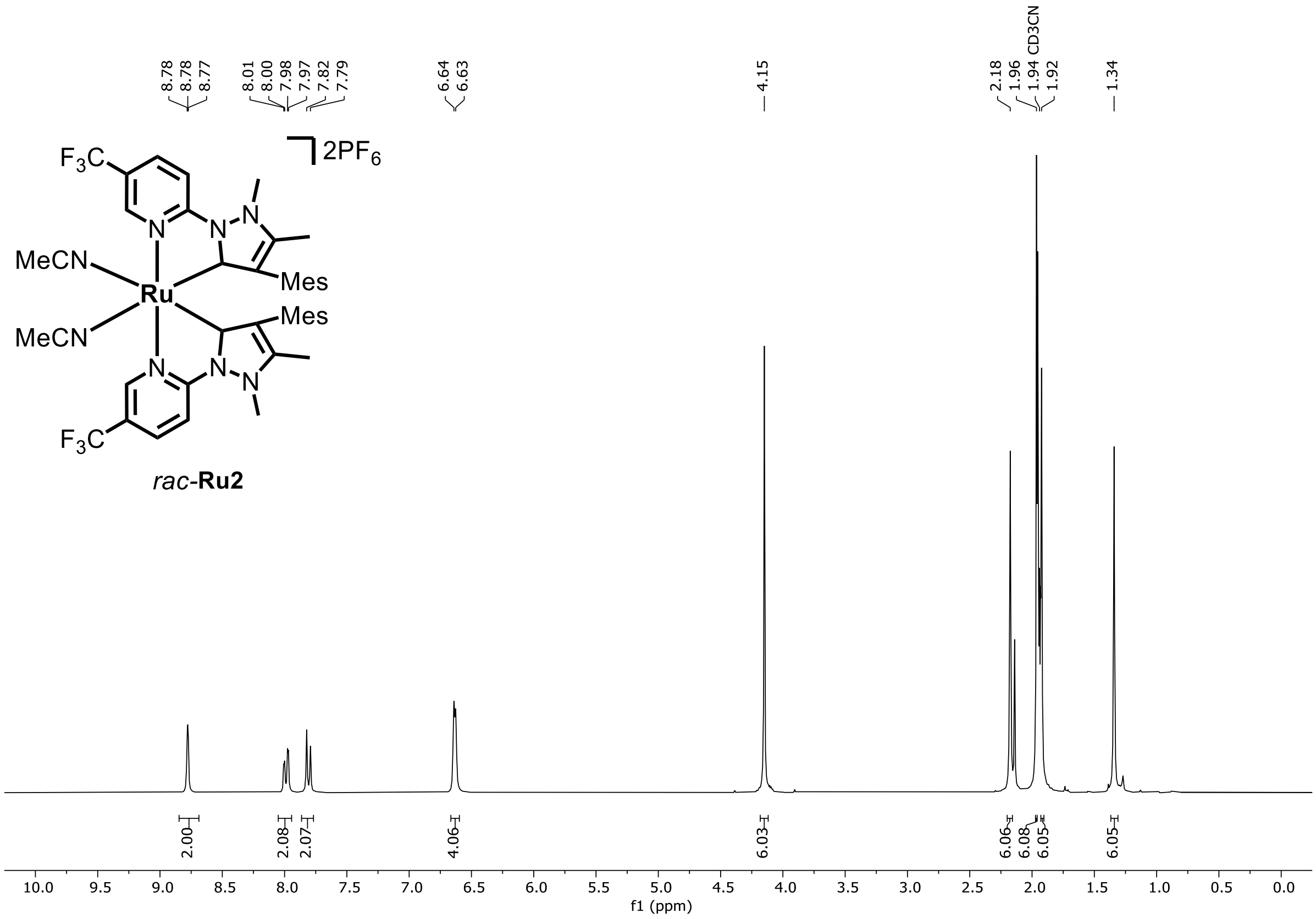

Figure S34: ^{1}H NMR spectrum of *rac*-**Ru2** (300 MHz, CD_3CN, 300 K).

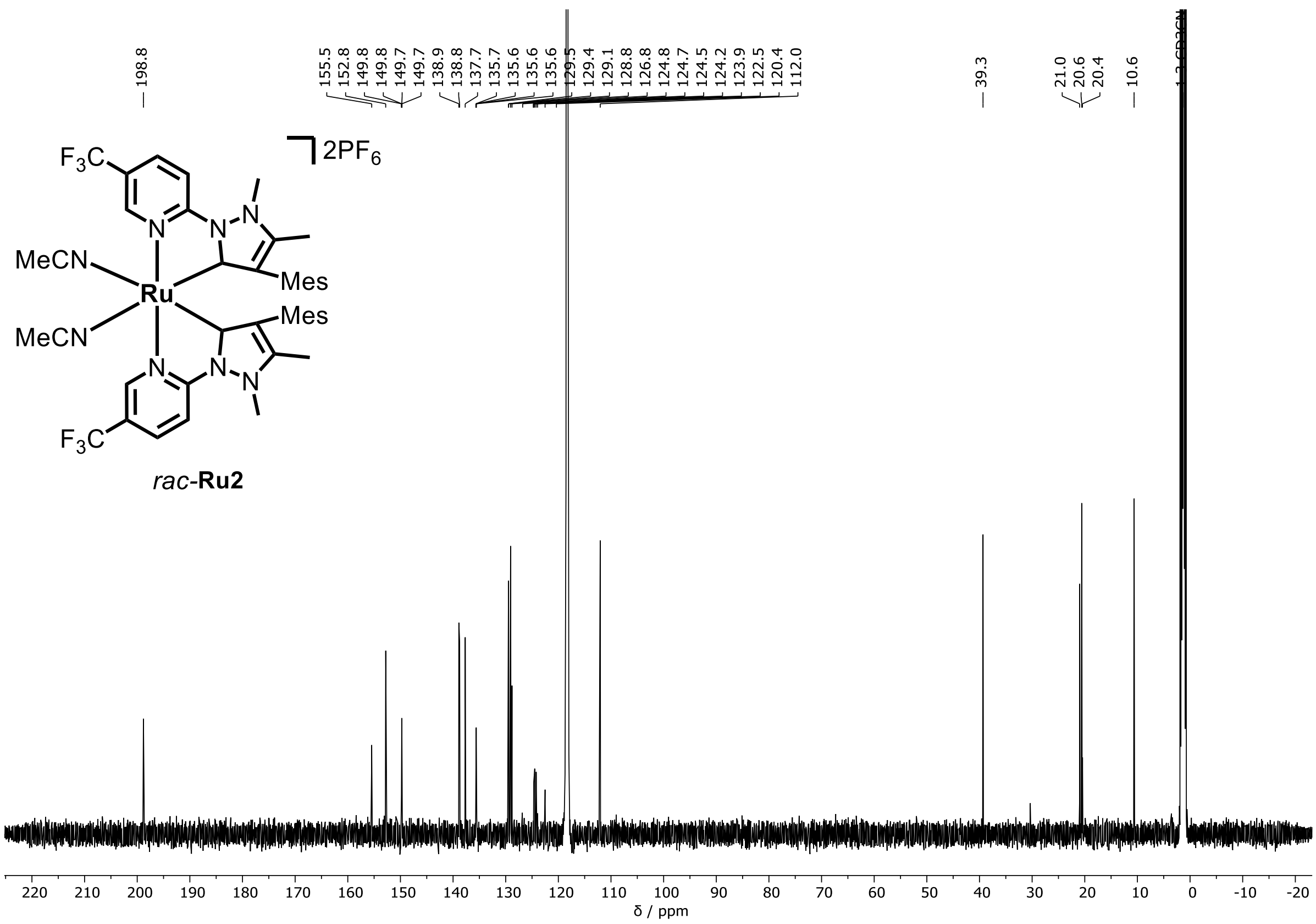

Figure S35: ^{13}C NMR spectrum of *rac*-**Ru2** (126 MHz, CD_3CN, 300 K).

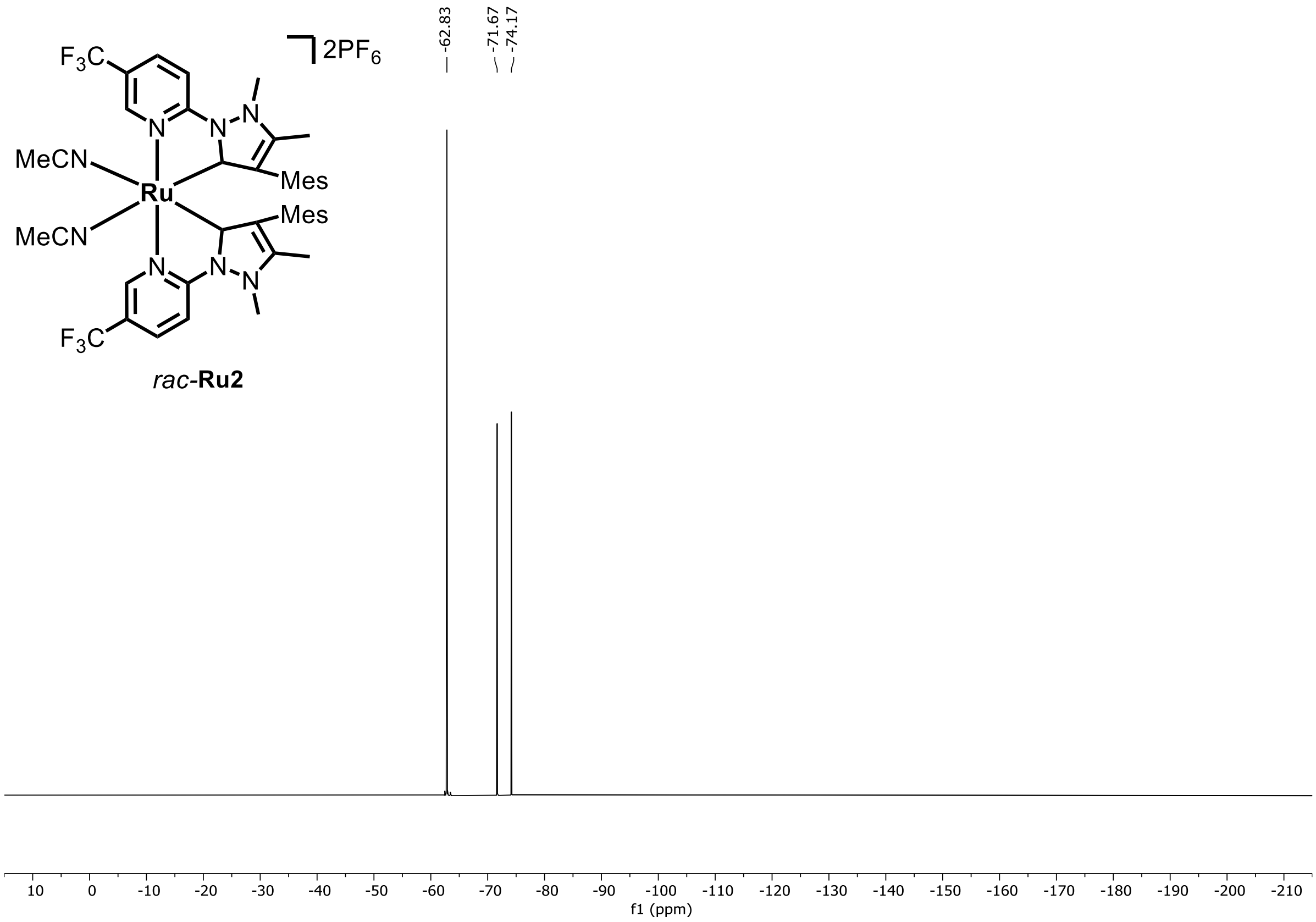

Figure S36: ^{19}F NMR spectrum of *rac*-**Ru2** (282 MHz, CD_3CN, 300 K).

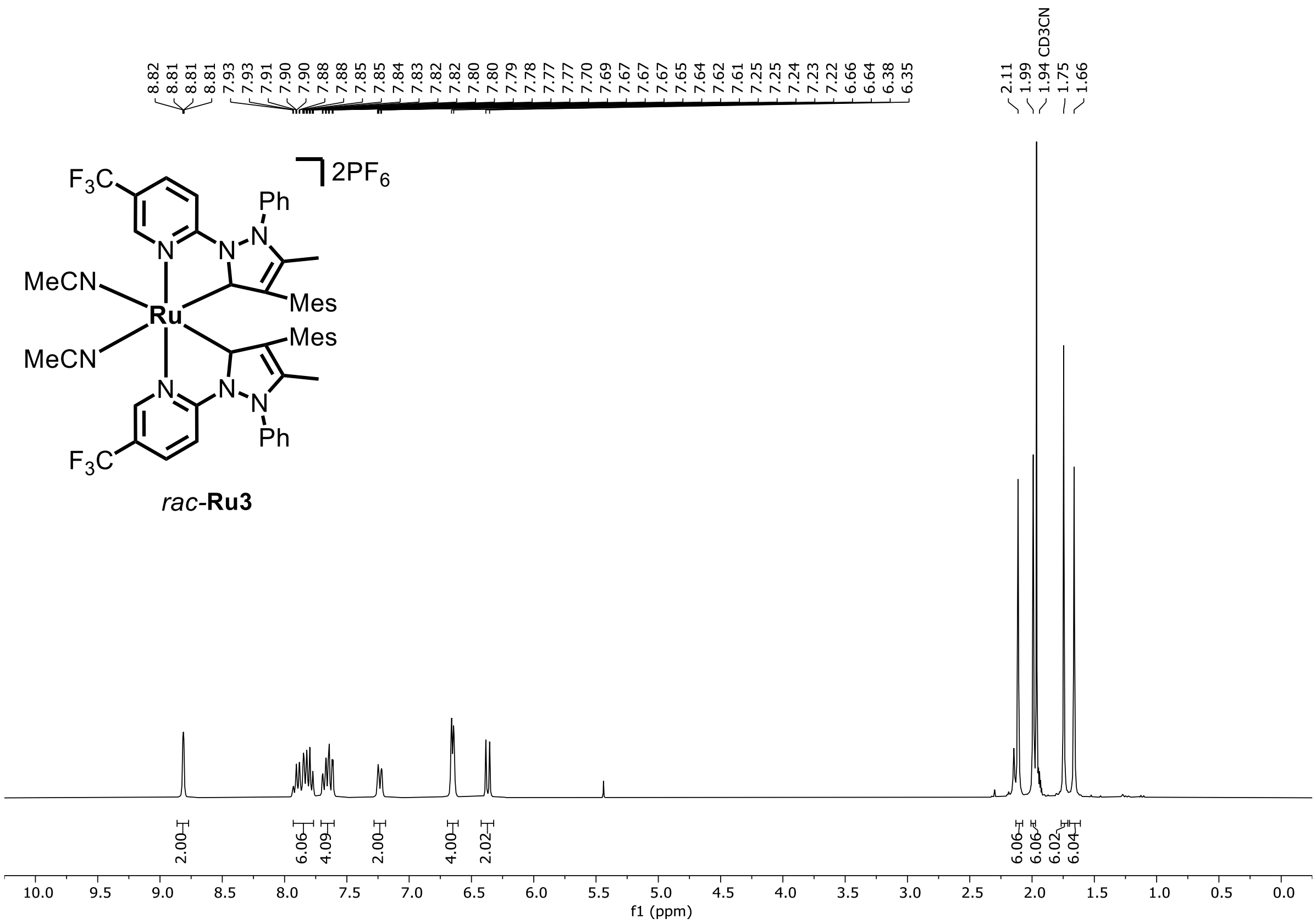

Figure S37: ^{1}H NMR spectrum of *rac*-**Ru3** (300 MHz, CD_3CN, 300 K).

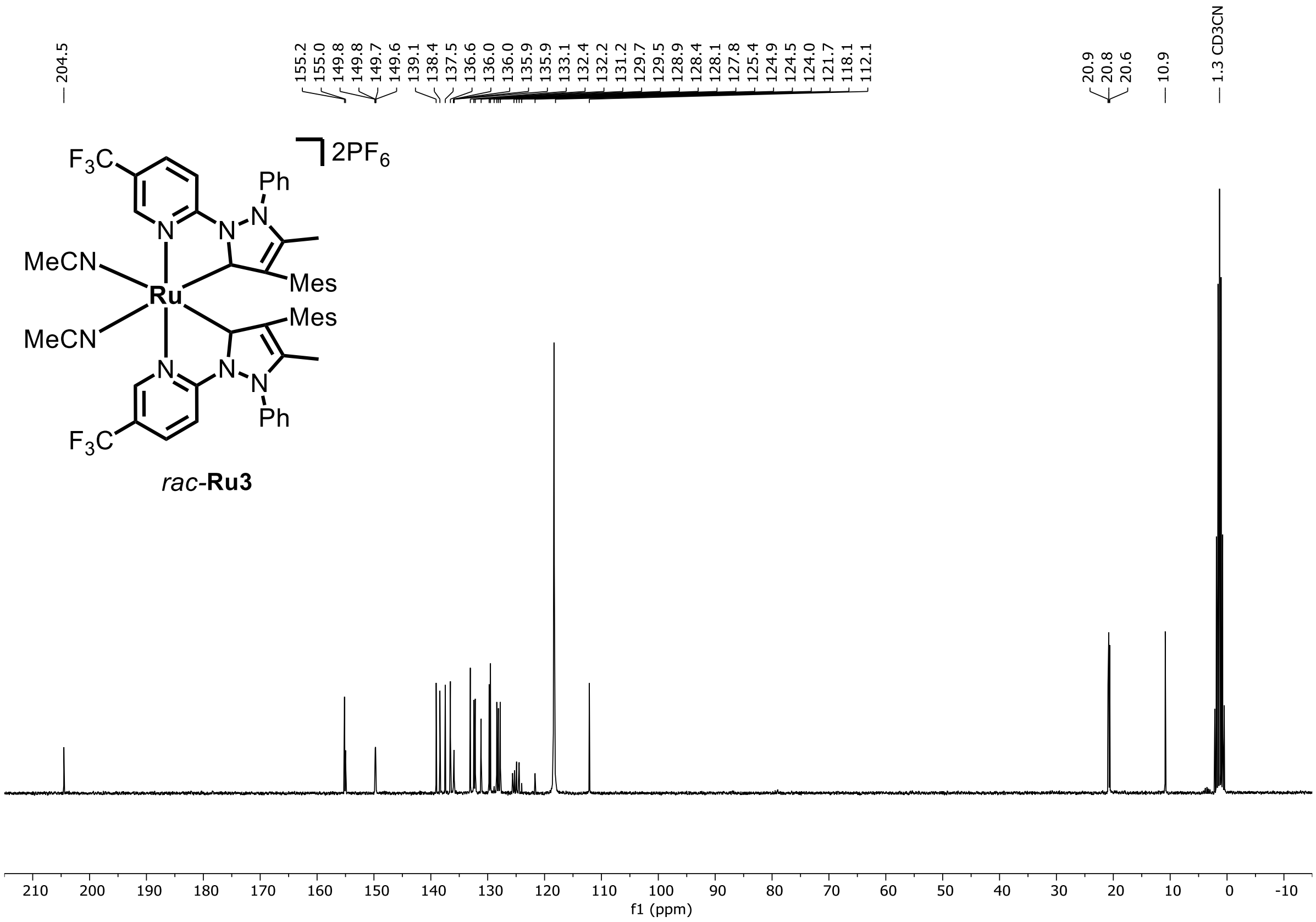

Figure S38: ^{13}C NMR spectrum of *rac*-**Ru3** (75 MHz, CD_3CN, 300 K).

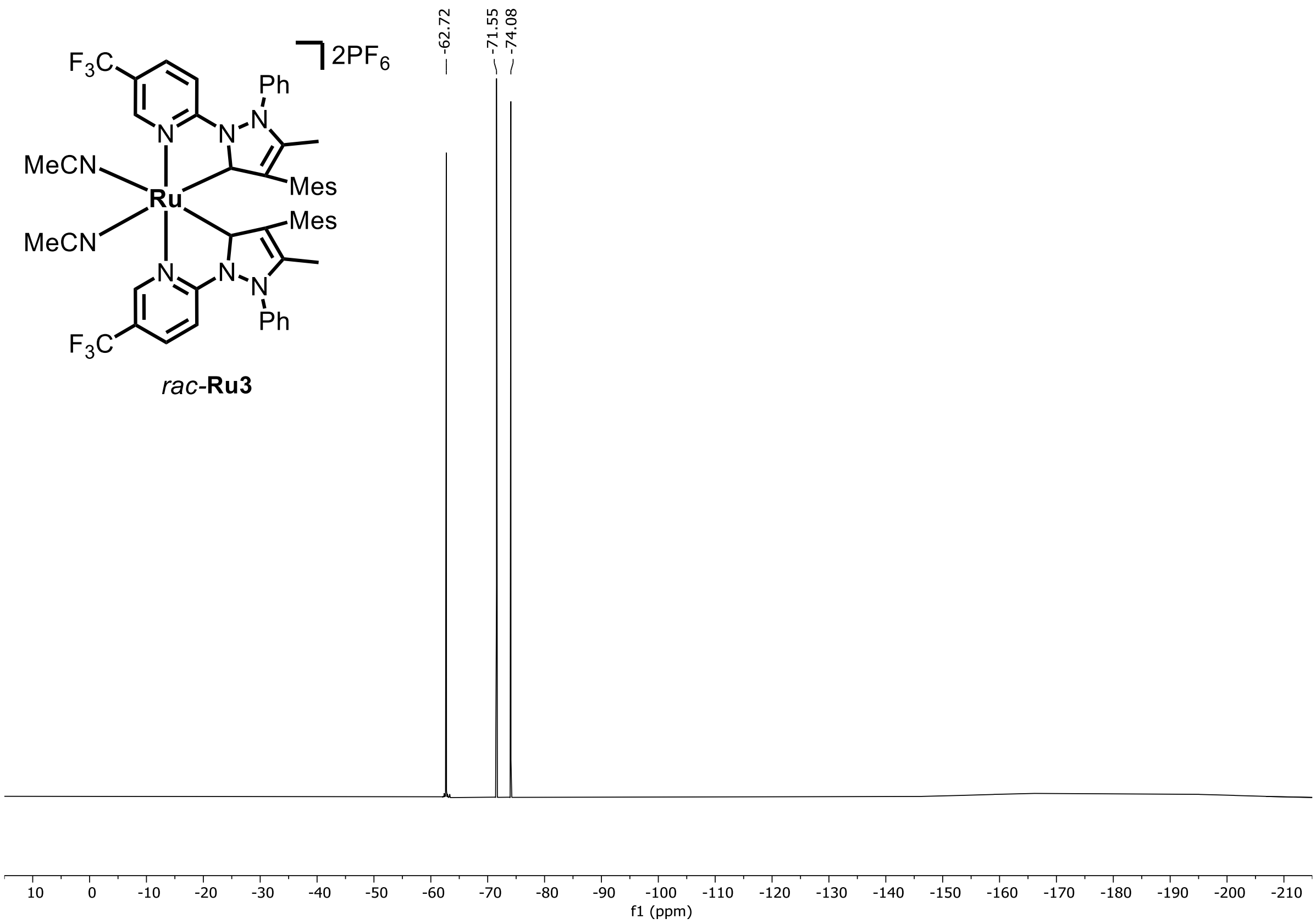

Figure S39: ^{19}F NMR spectrum of *rac*-**Ru3** (282 MHz, CD_3CN, 300 K).

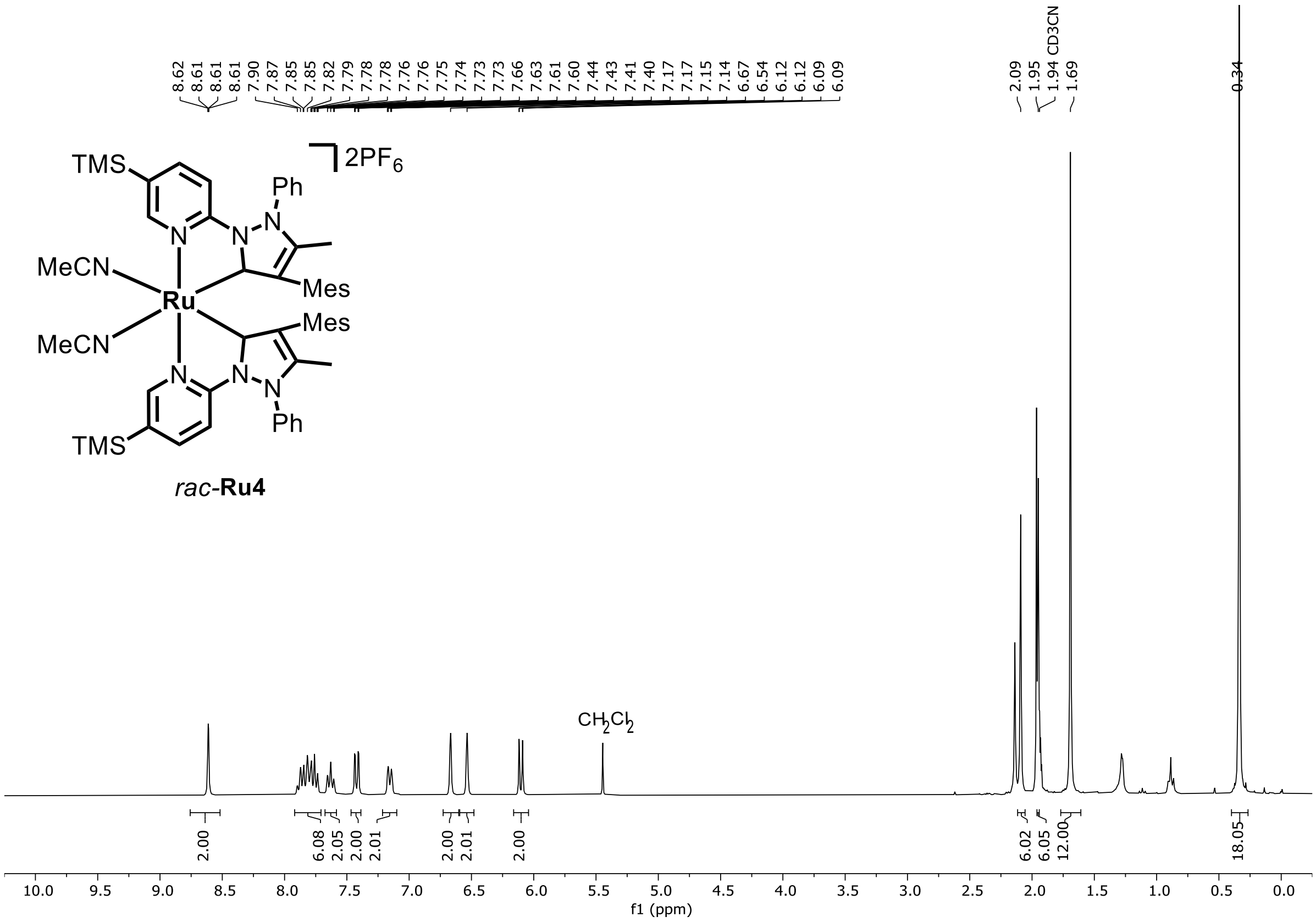

Figure S40: ^{1}H NMR spectrum of *rac*-**Ru4** (300 MHz, CD_3CN, 300 K).

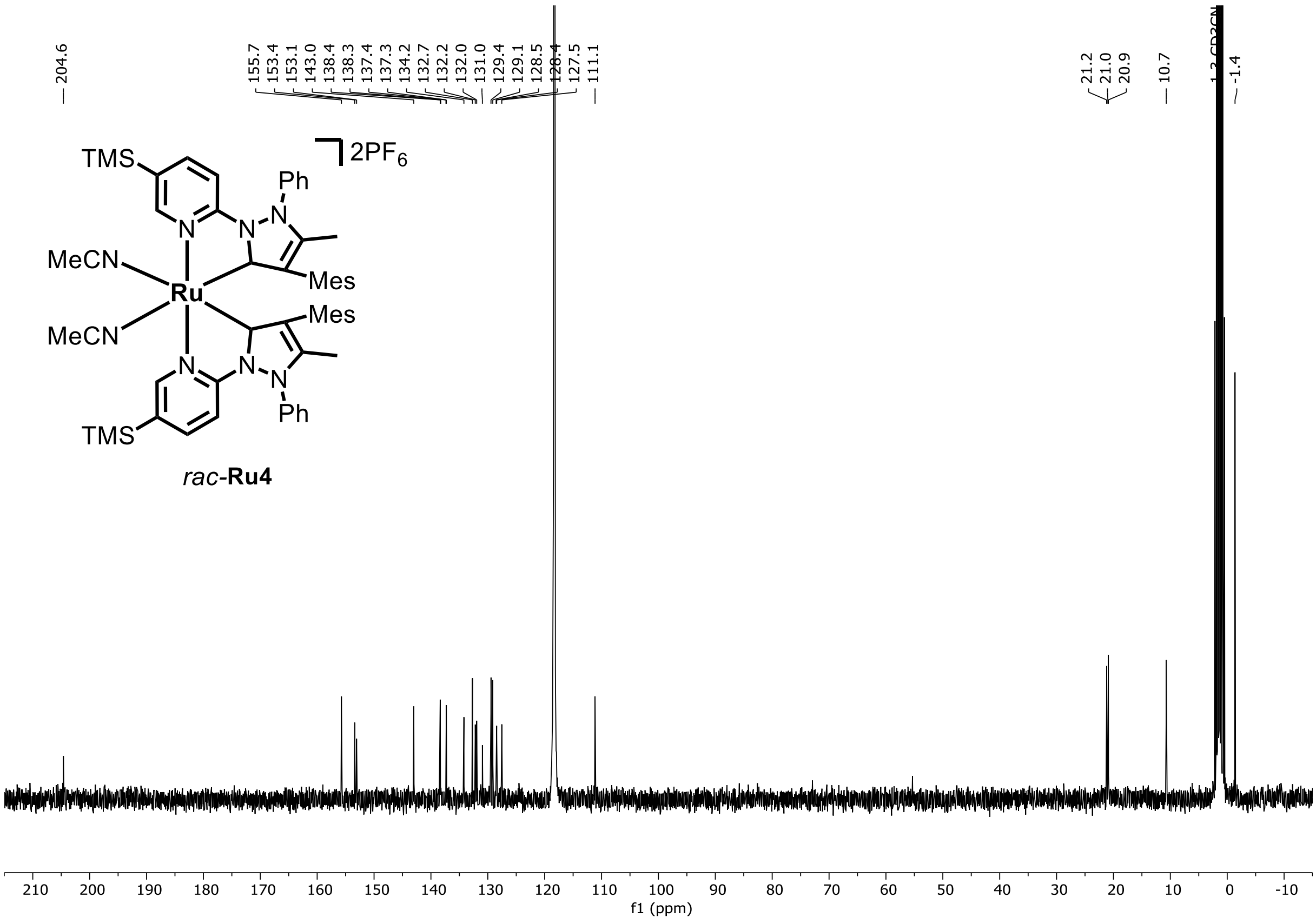

Figure S41: ^{13}C NMR spectrum of *rac*-**Ru4** (75 MHz, CD_3CN, 300 K).

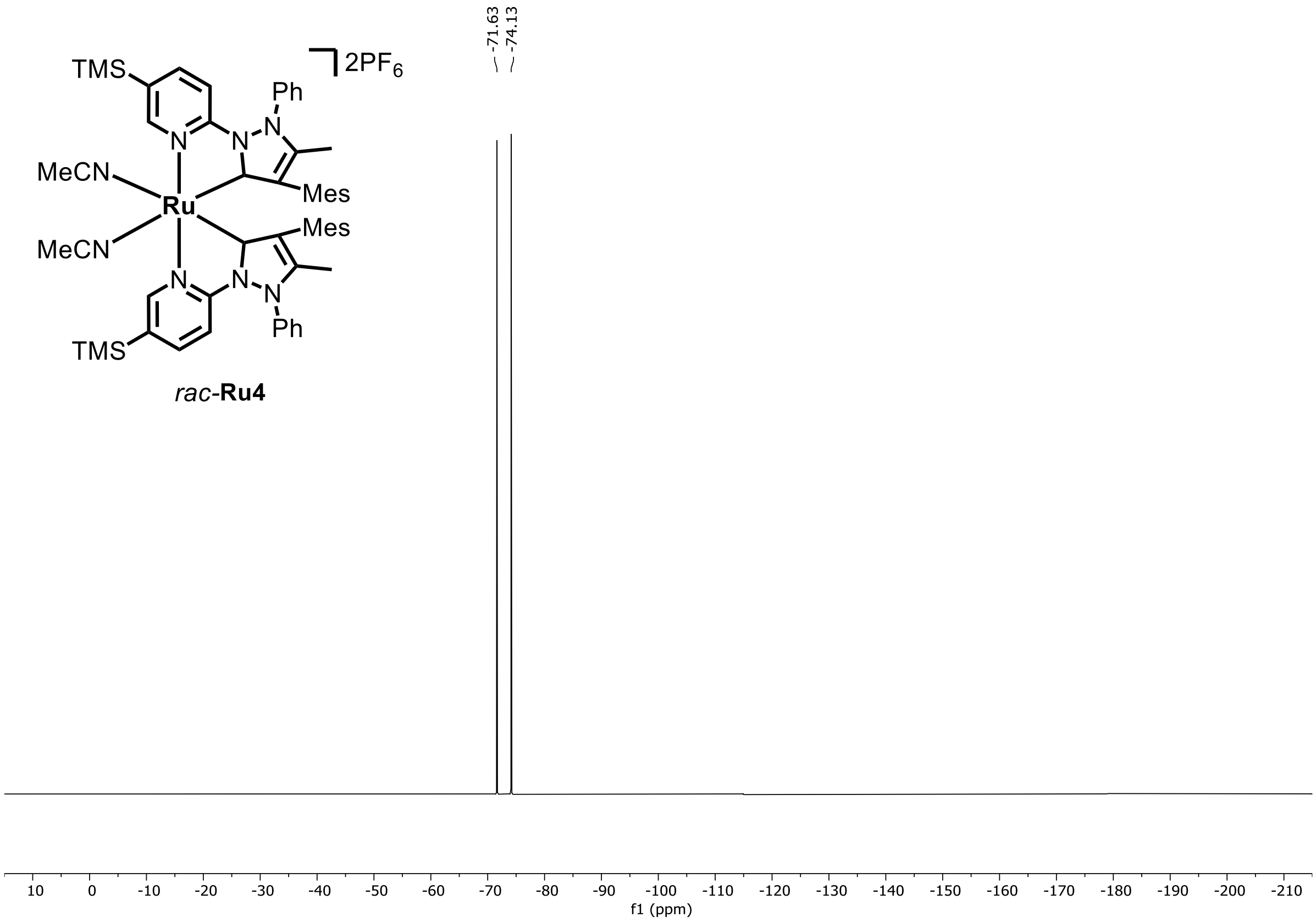

Figure S42: ^{19}F NMR spectrum of *rac*-**Ru4** (282 MHz, CD_3CN, 300 K).

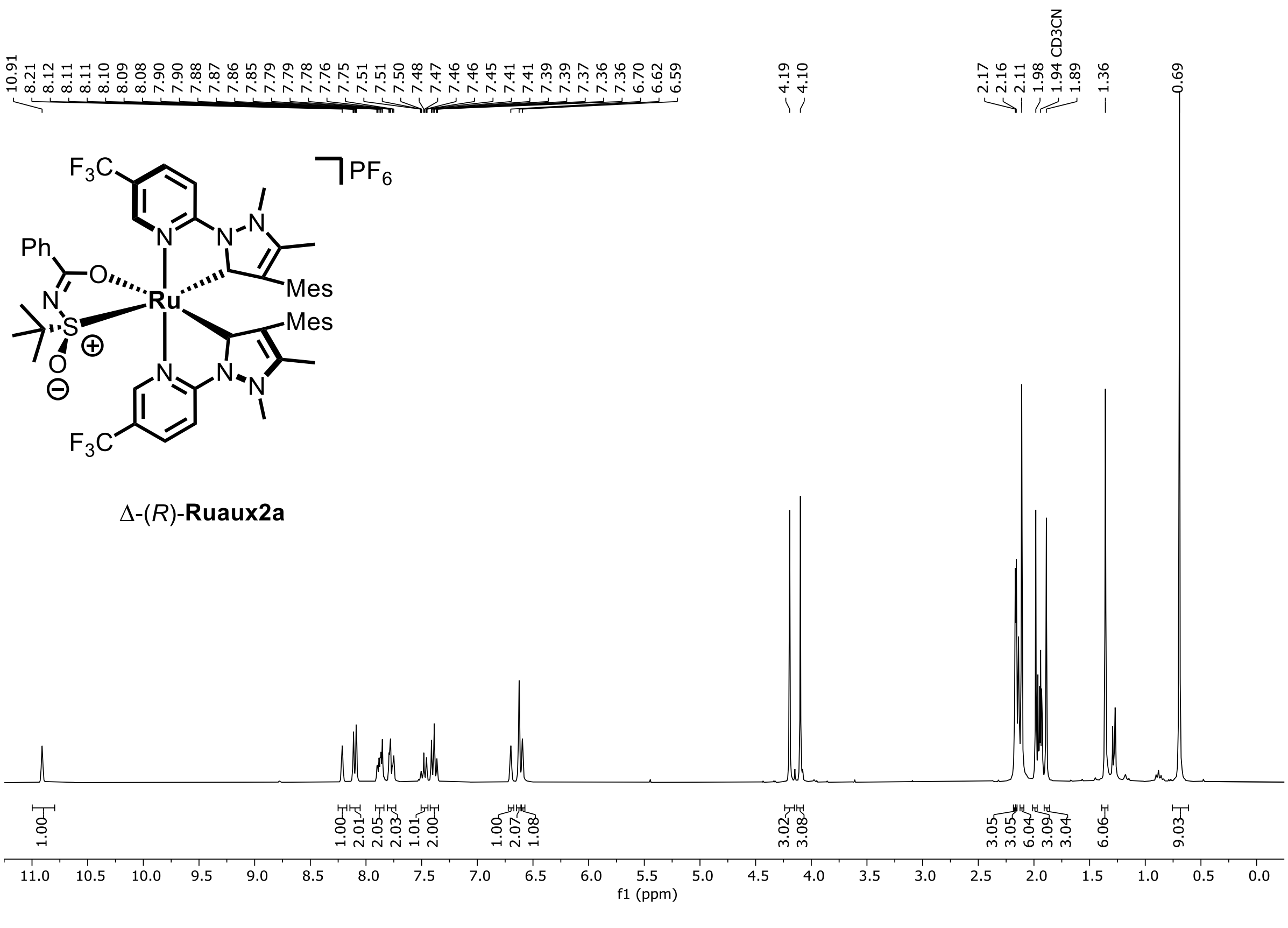

Figure S43: ^{1}H NMR spectrum of Δ-(*R*)-**Ruaux2a** (300 MHz, CD_3CN, 300 K).

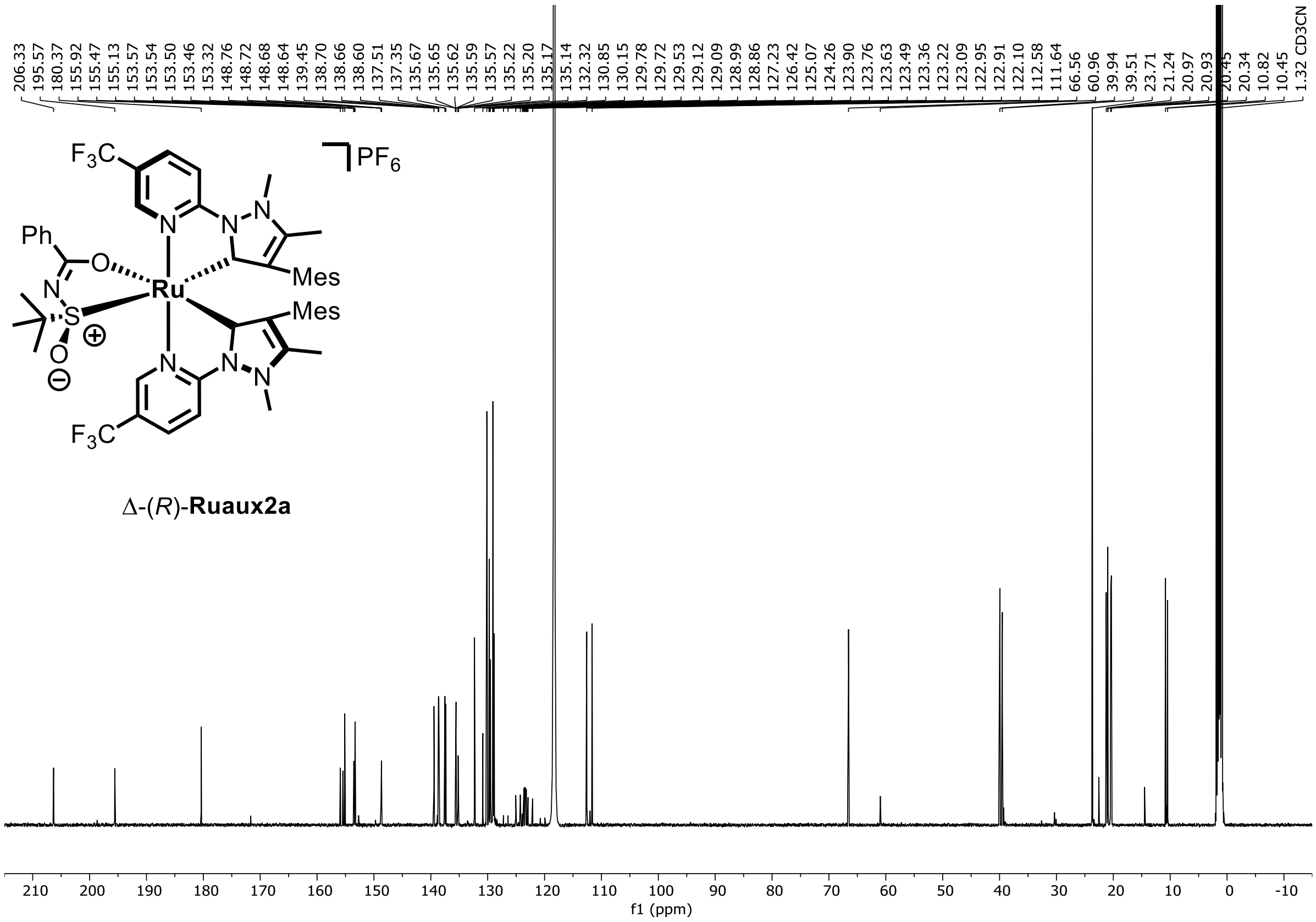

Figure S44: ^{13}C NMR spectrum of Δ-(*R*)-**Ruaux2a** (126 MHz, CD_3CN, 300 K).

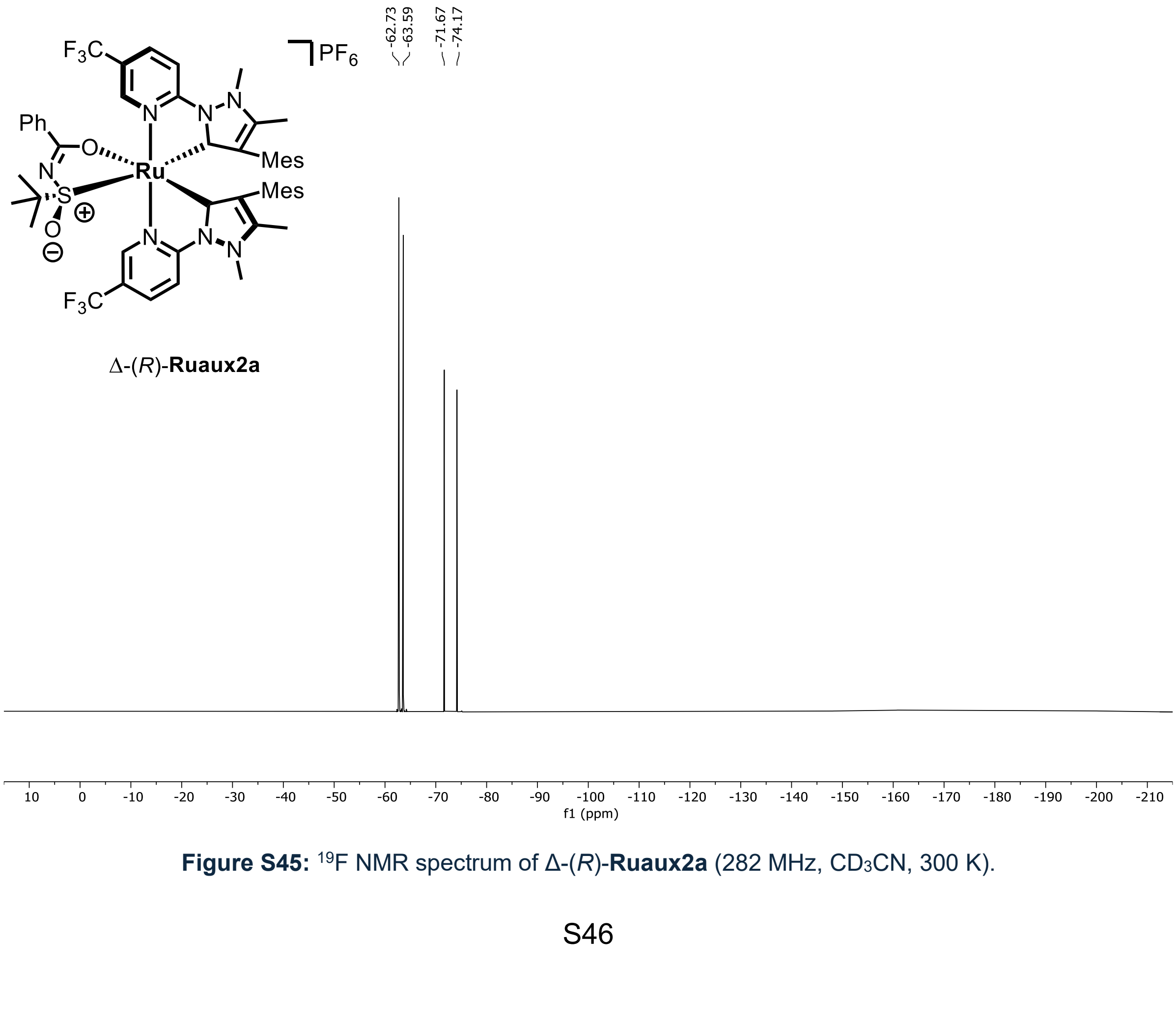

Figure S45: ^{19}F NMR spectrum of Δ-(*R*)-**Ruaux2a** (282 MHz, CD_3CN, 300 K).

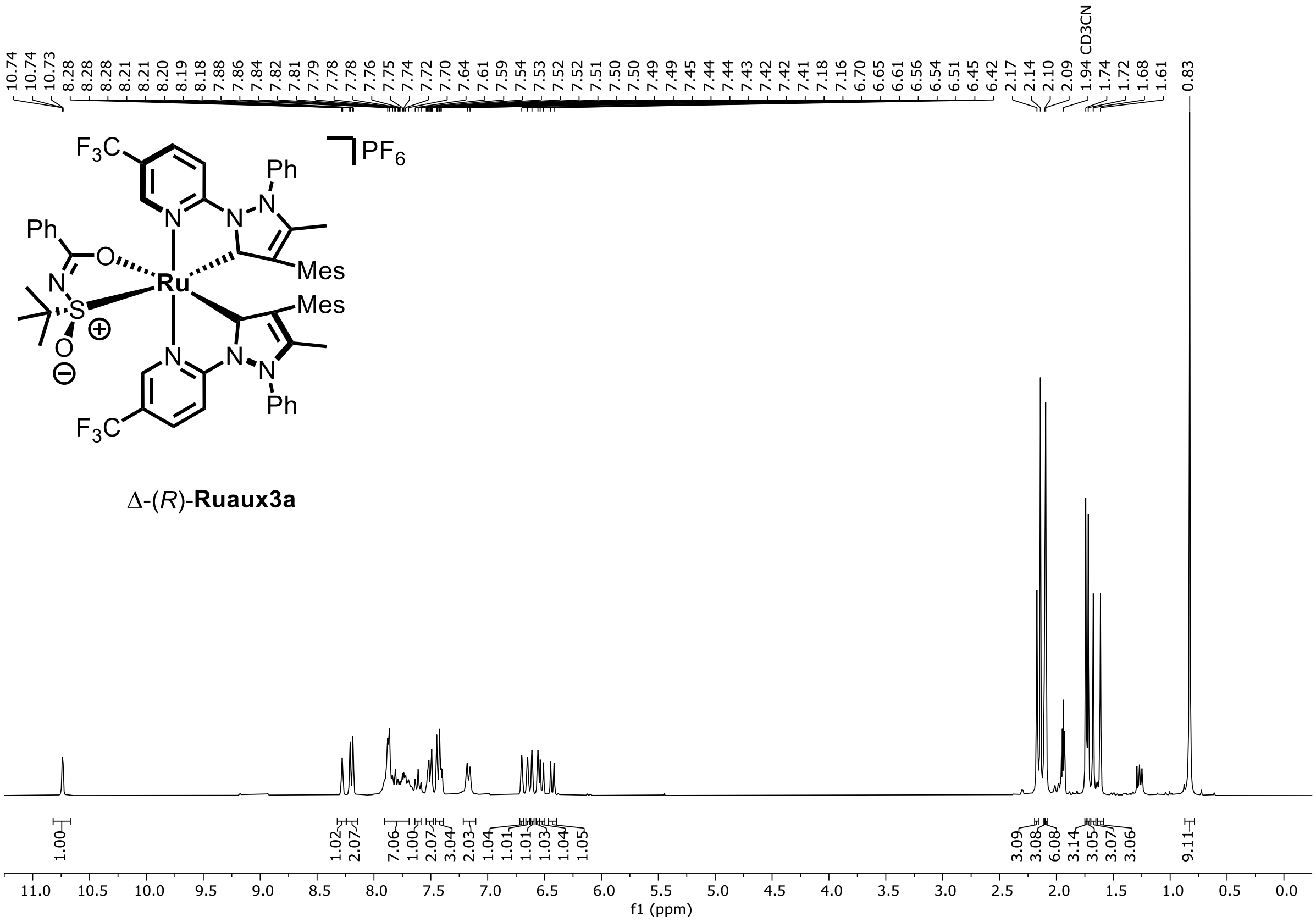

Figure S46: ^{1}H NMR spectrum of Δ-(*R*)-**Ruaux3a** (300 MHz, CD_3CN, 300 K).

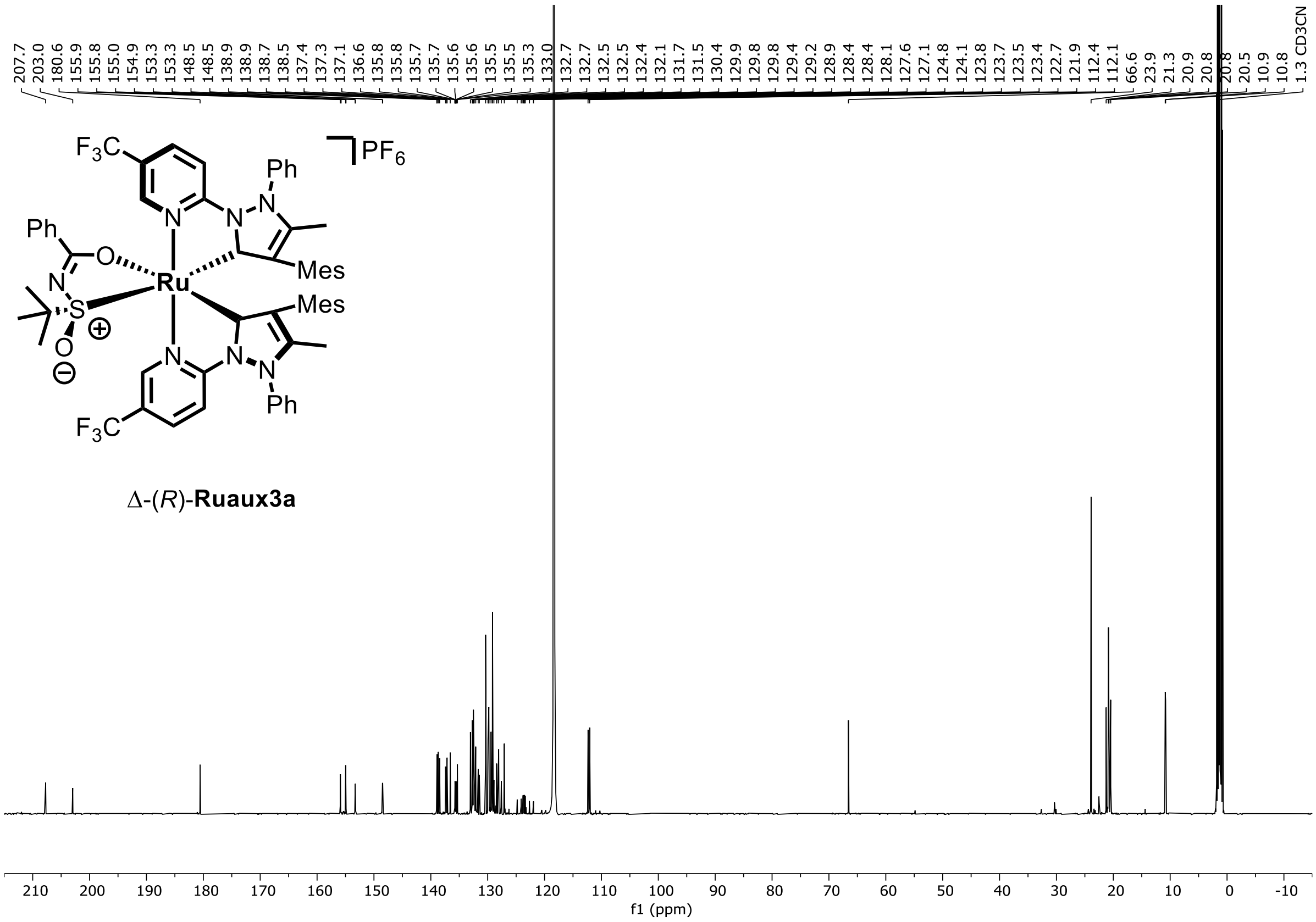

Figure S47: ^{13}C NMR spectrum of Δ-(*R*)-**Ruaux3a** (126 MHz, CD_3CN, 300 K).

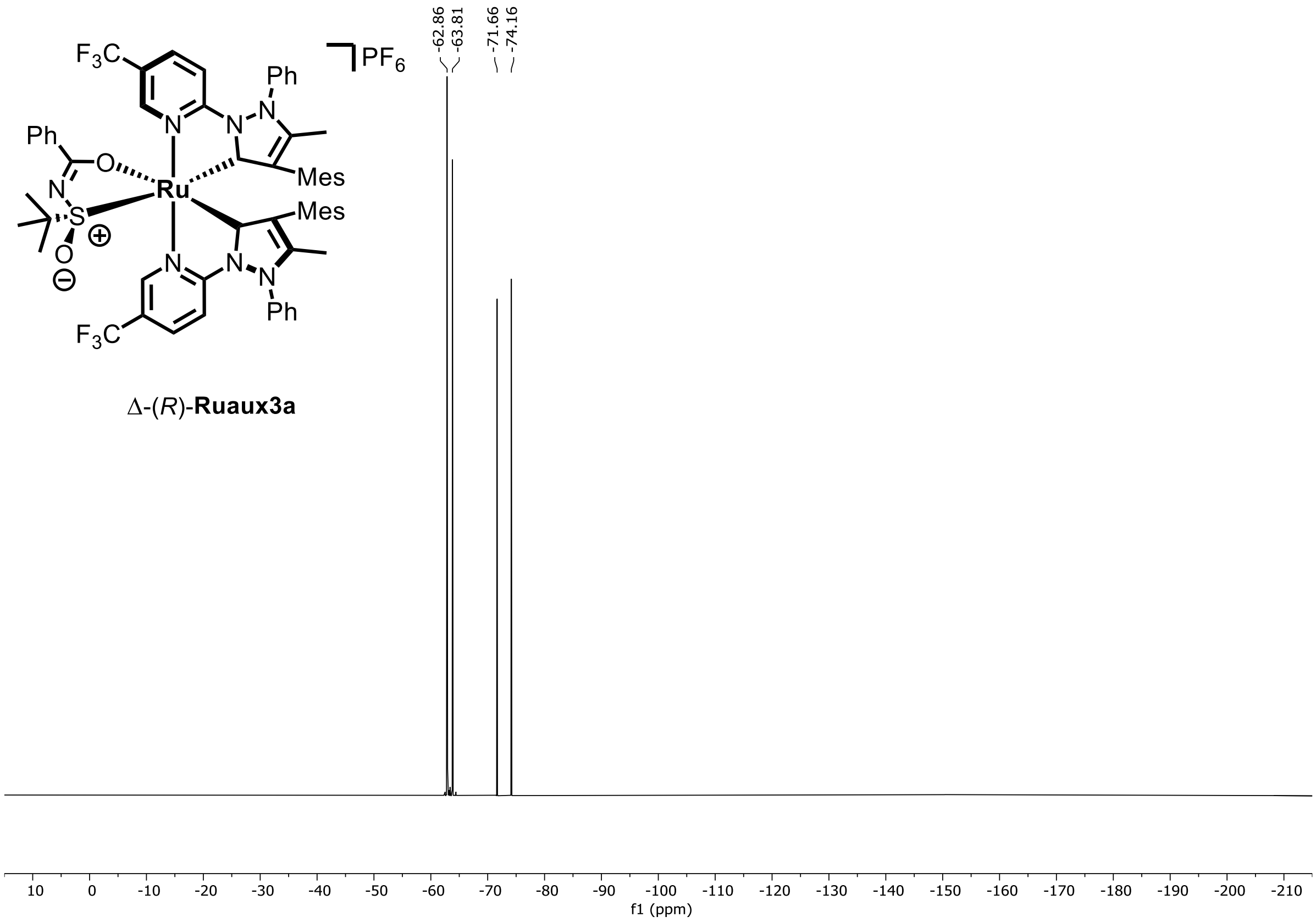

Figure S48: ^{19}F NMR spectrum of Δ-(*R*)-**Ruaux3a** (282 MHz, CD_3CN, 300 K).

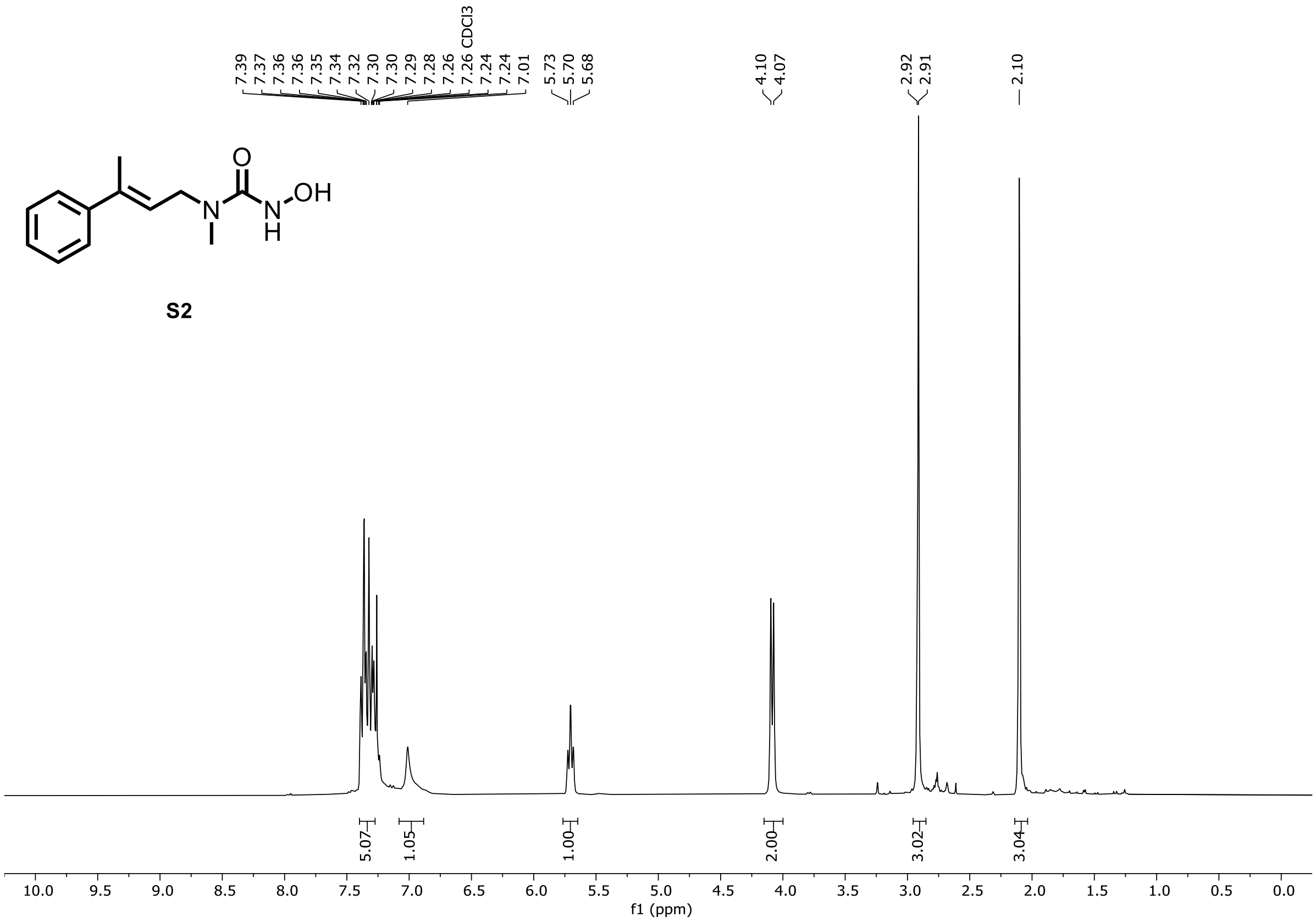

Figure S49: ^{1}H NMR spectrum of **S2** (300 MHz, $CDCl_3$, 300 K).

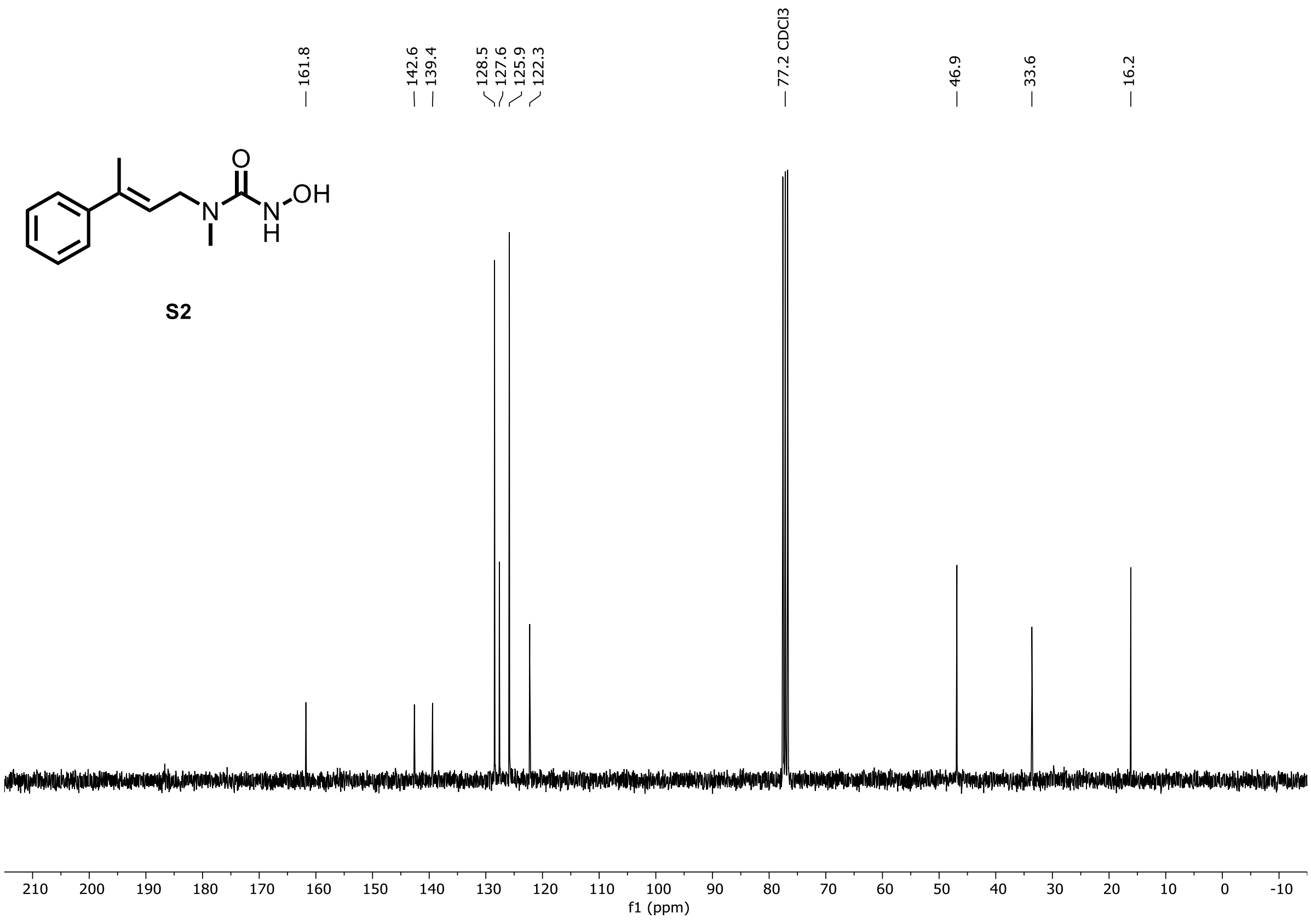

Figure S50: ^{13}C NMR spectrum of **S2** (75 MHz, $CDCl_3$, 300 K).

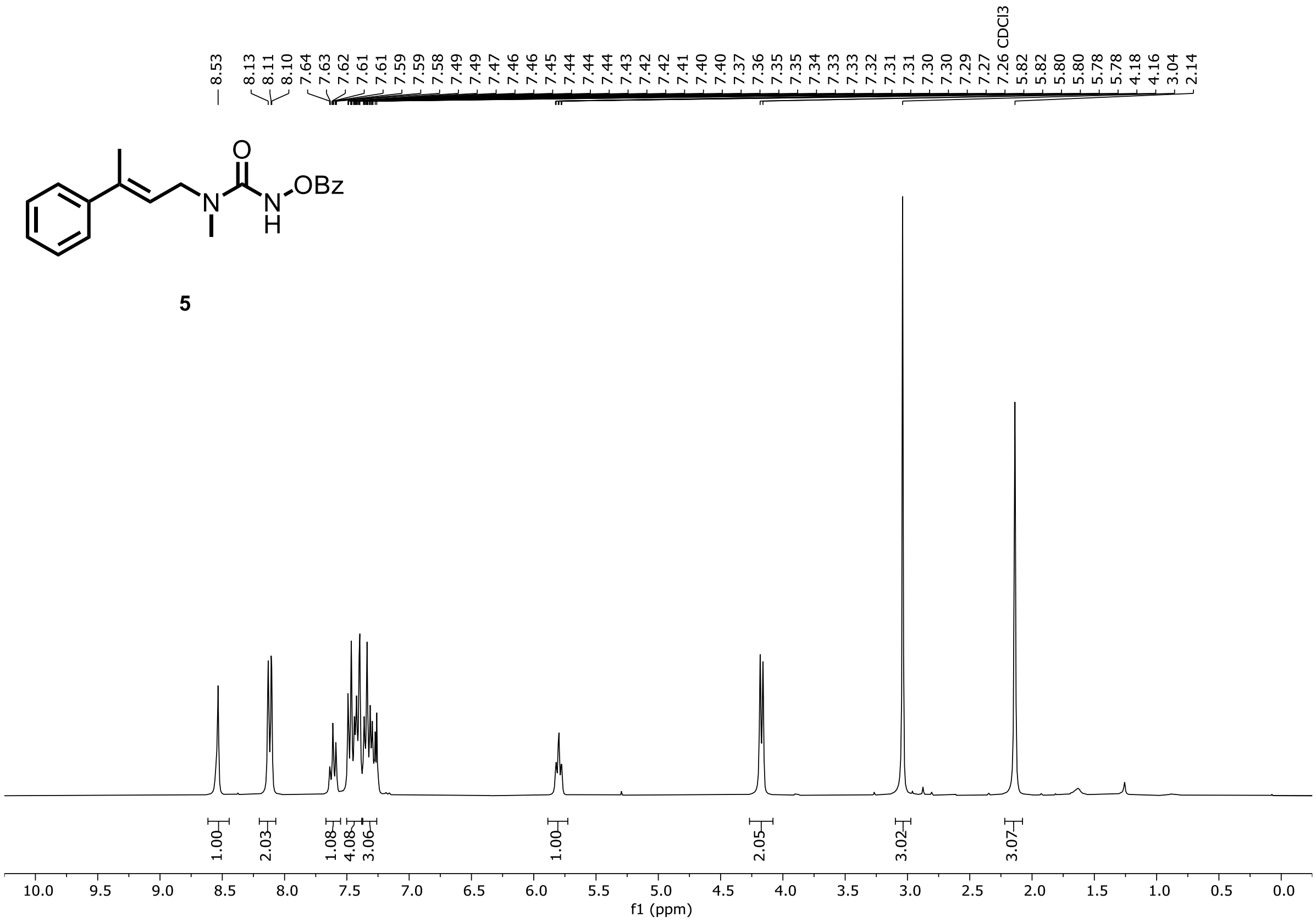

Figure S51: ^{1}H NMR spectrum of **5** (300 MHz, $CDCl_3$, 300 K).

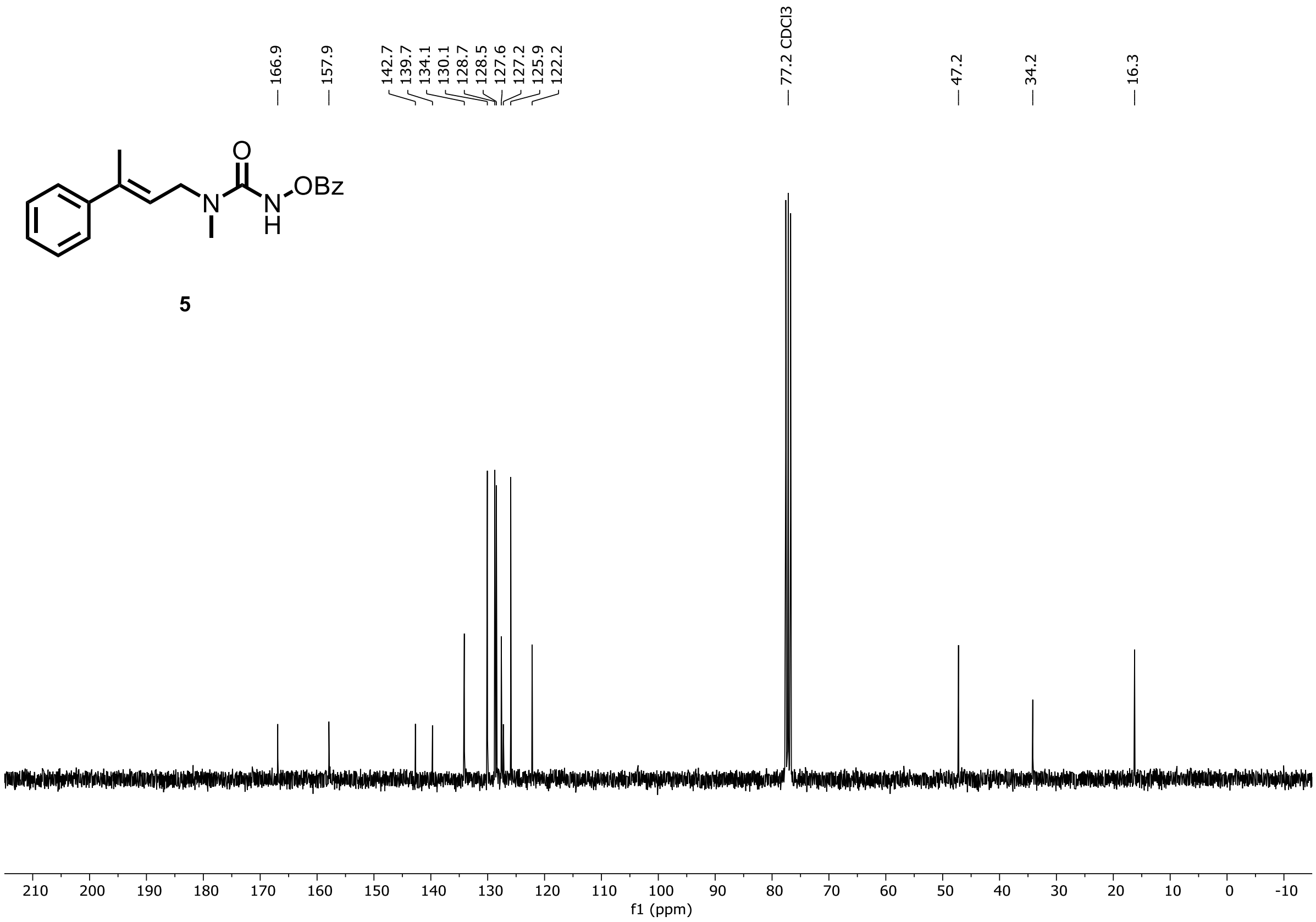

Figure S52: ^{13}C NMR spectrum of **5** (75 MHz, $CDCl_3$, 300 K).

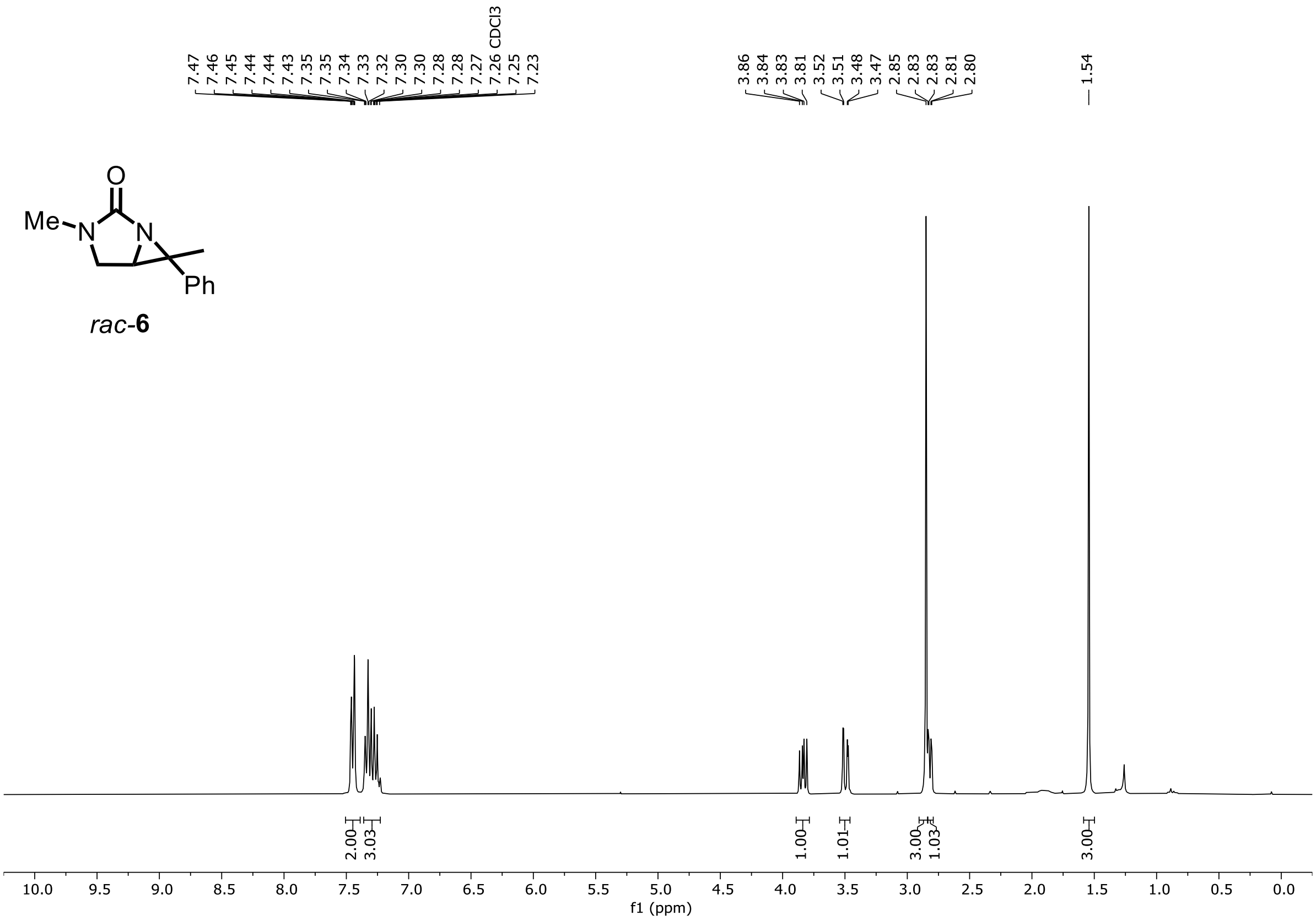

Figure S53: ^{1}H NMR spectrum of **6** (300 MHz, $CDCl_3$, 300 K).

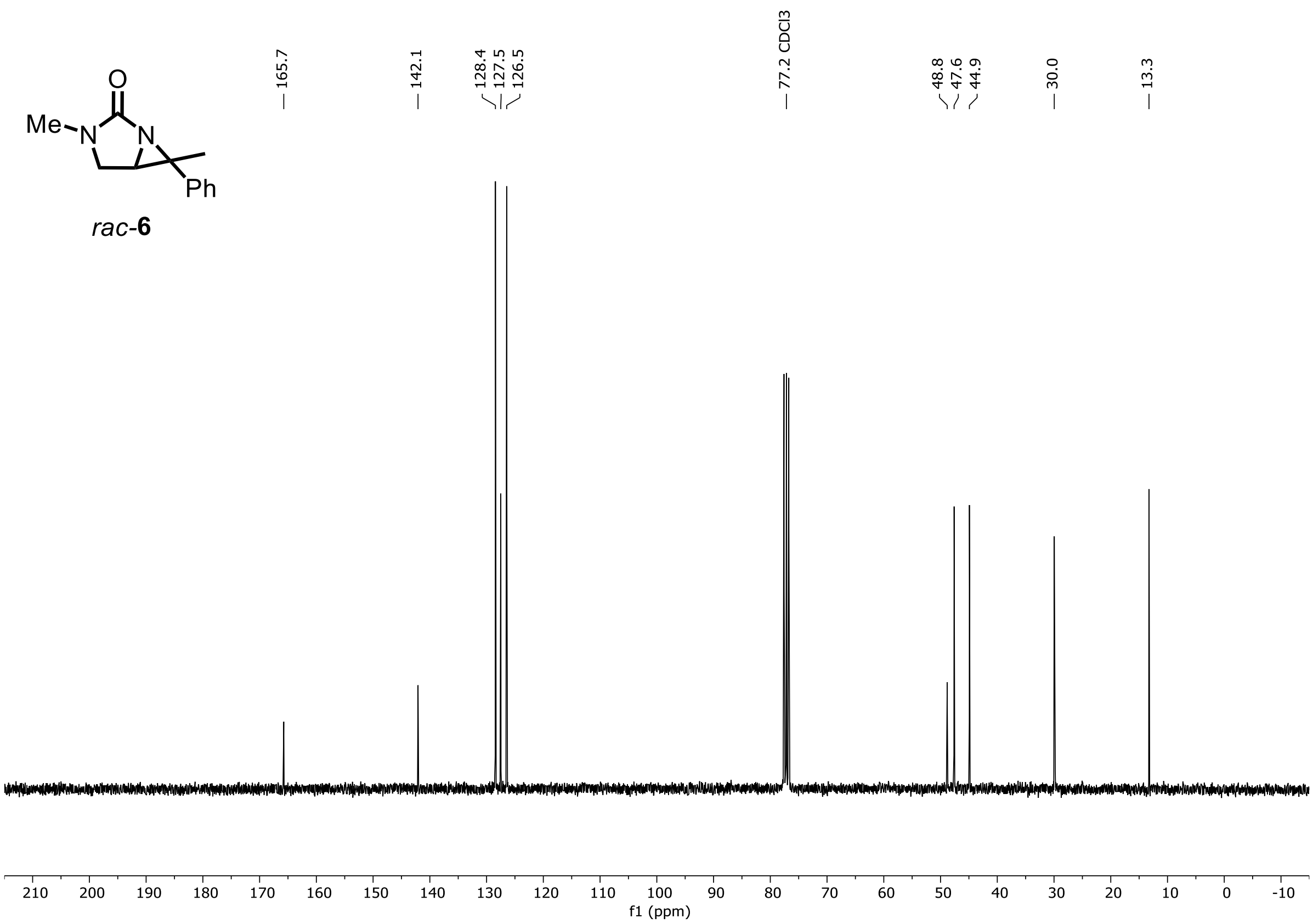

Figure S54: ^{13}C NMR spectrum of **5** (75 MHz, $CDCl_3$, 300 K).

11. Chiral HPLC Traces

Sample Information

Sample Name : MFP394-1_racemic

Conditions : IB-N 5, n-Hex/iPrOH 80:20, 1 mL/min, 230 nm, 25 °C, 12 μL

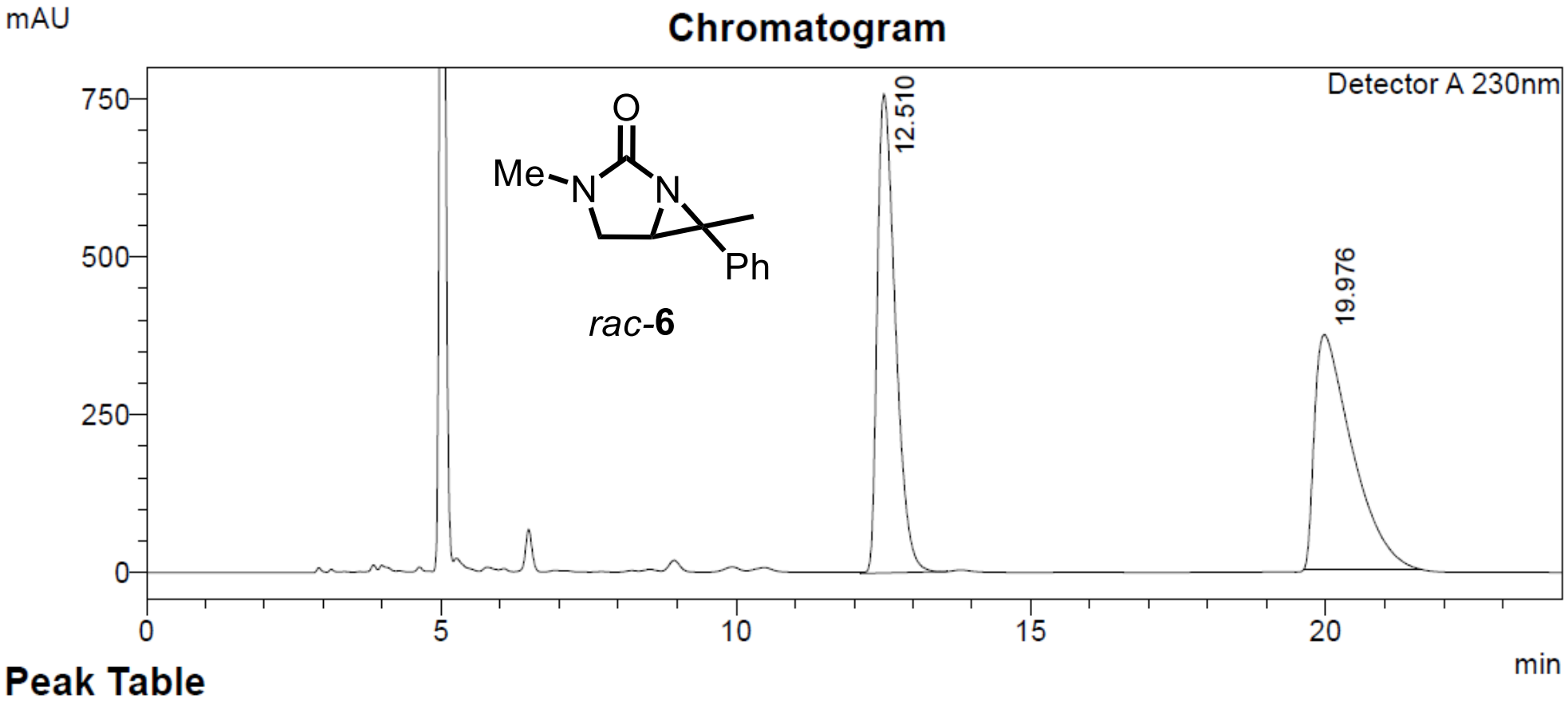

Peak Table

Peak#	Ret. Time	Area	Height	Area%
1	12.510	16430555	759386	49.96
2	19.976	16455074	370946	50.04
Total		32885630	1130332	100.00

Sample Information

Sample Name : MFP399-1

Conditions : IB-N 5, n-Hex/iPrOH 80:20, 1 mL/min, 230 nm, 25 °C, 12 μL

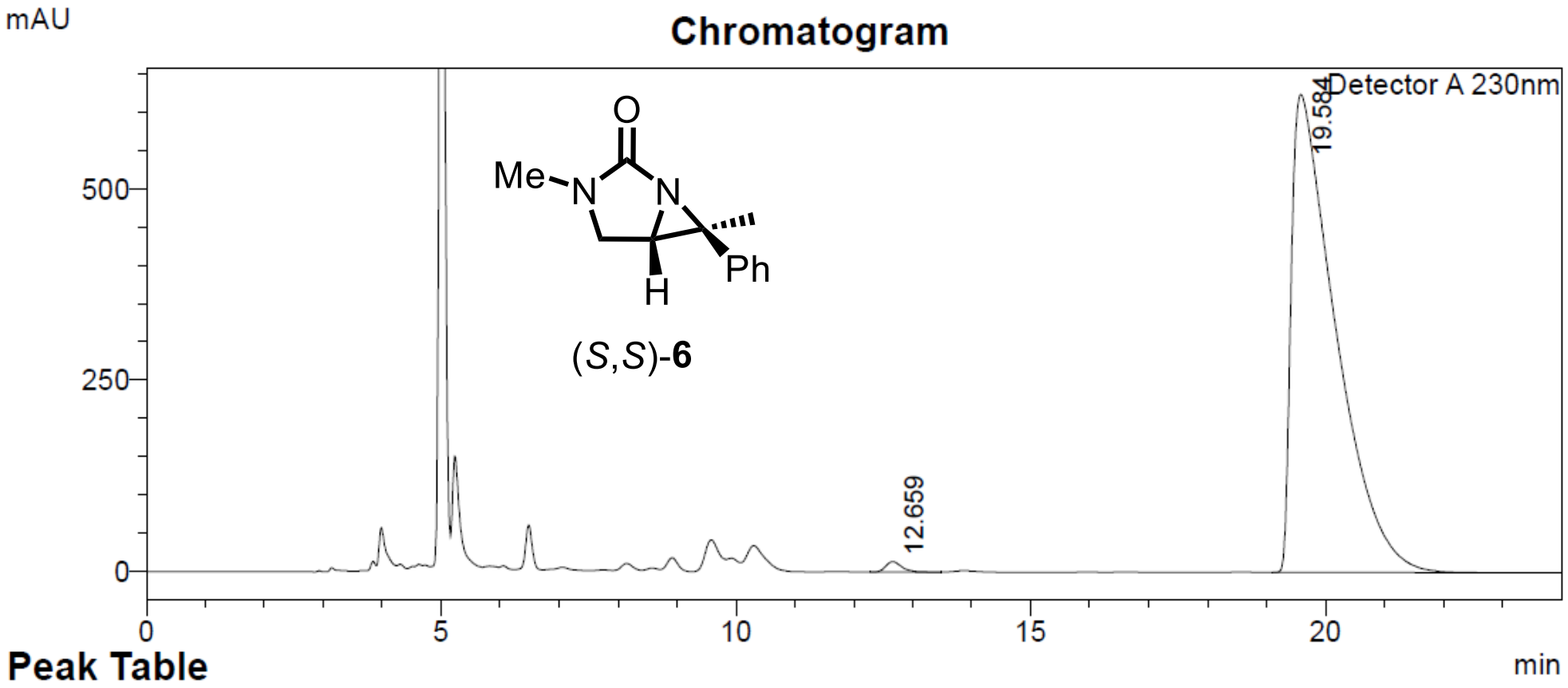

Peak Table

Peak#	Ret. Time	Area	Height	Area%
1	12.659	272775	13790	0.83
2	19.584	32659658	624313	99.17
Total		32932432	638102	100.00

Figure S55: HPLC traces of and *rac*-**6** and (*S*,*S*)-**6** (98%ee).

12. CD-Spectra

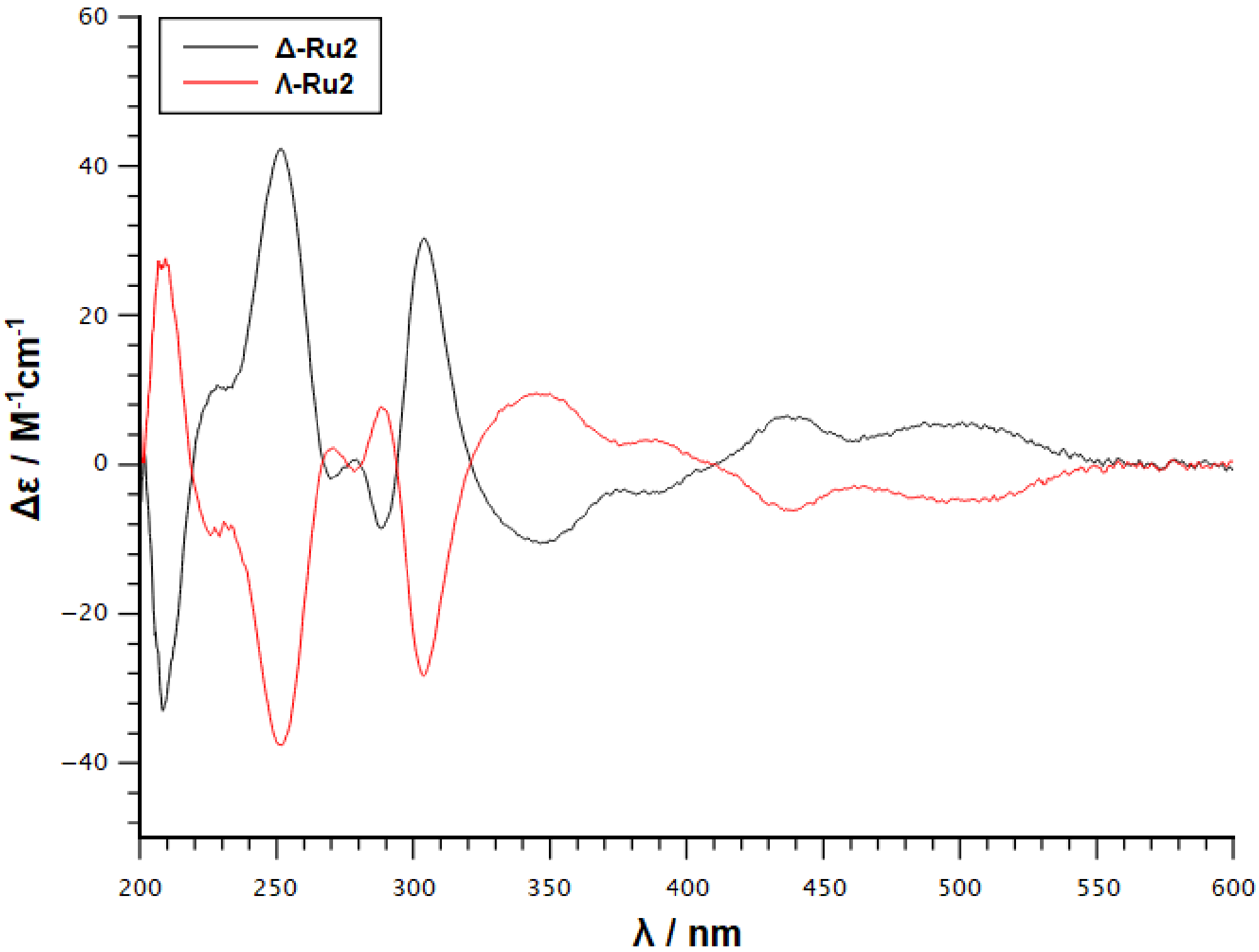

Figure S56: CD spectra of Δ-**Ru2** and Λ-**Ru2** in MeCN (0.25 mM).

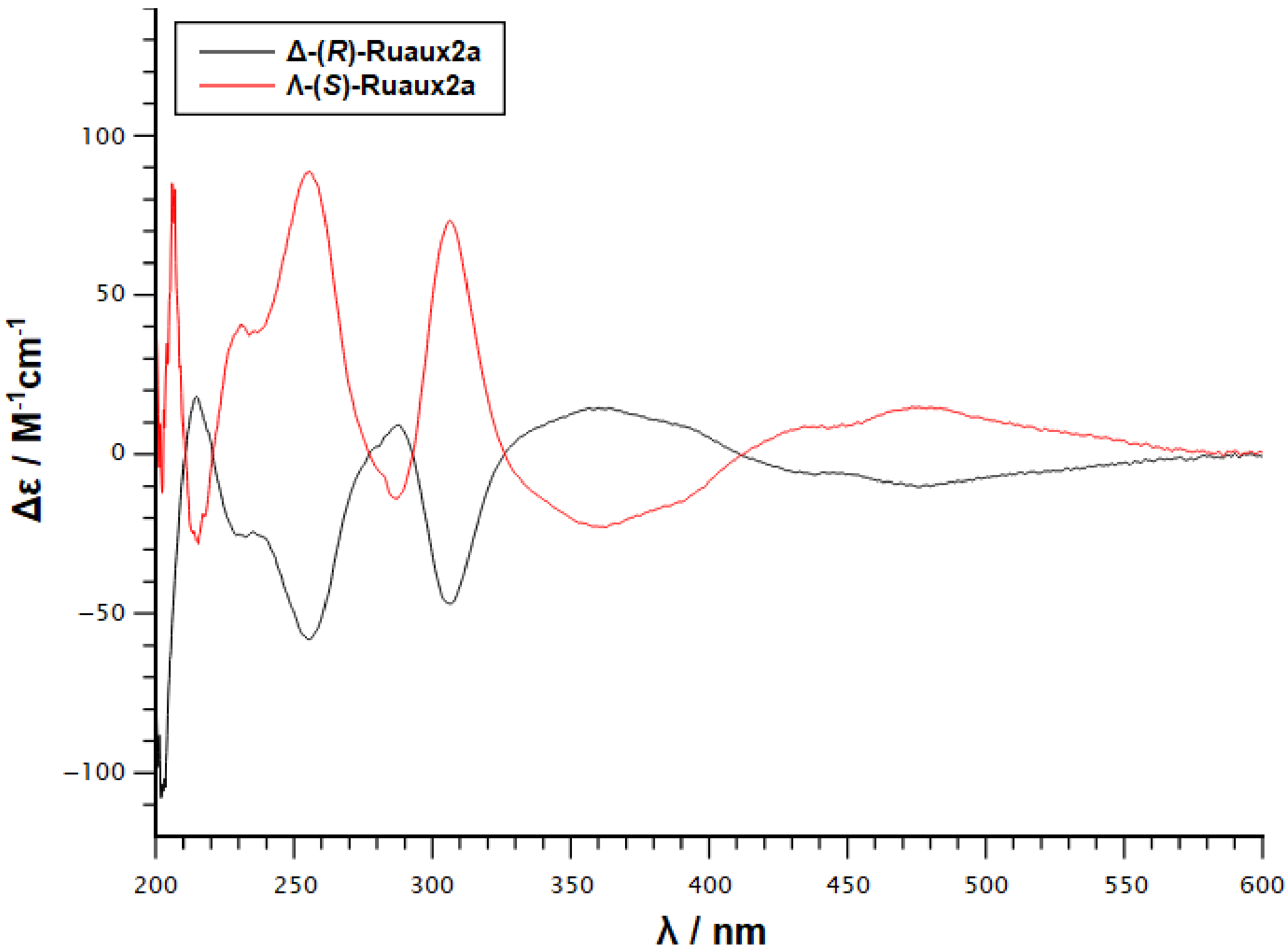

Figure S57: CD spectra of Δ-(*R*)-**Ruaux2a** and Λ-(*S*)-**Ruaux2a** in MeCN (0.25 mM).

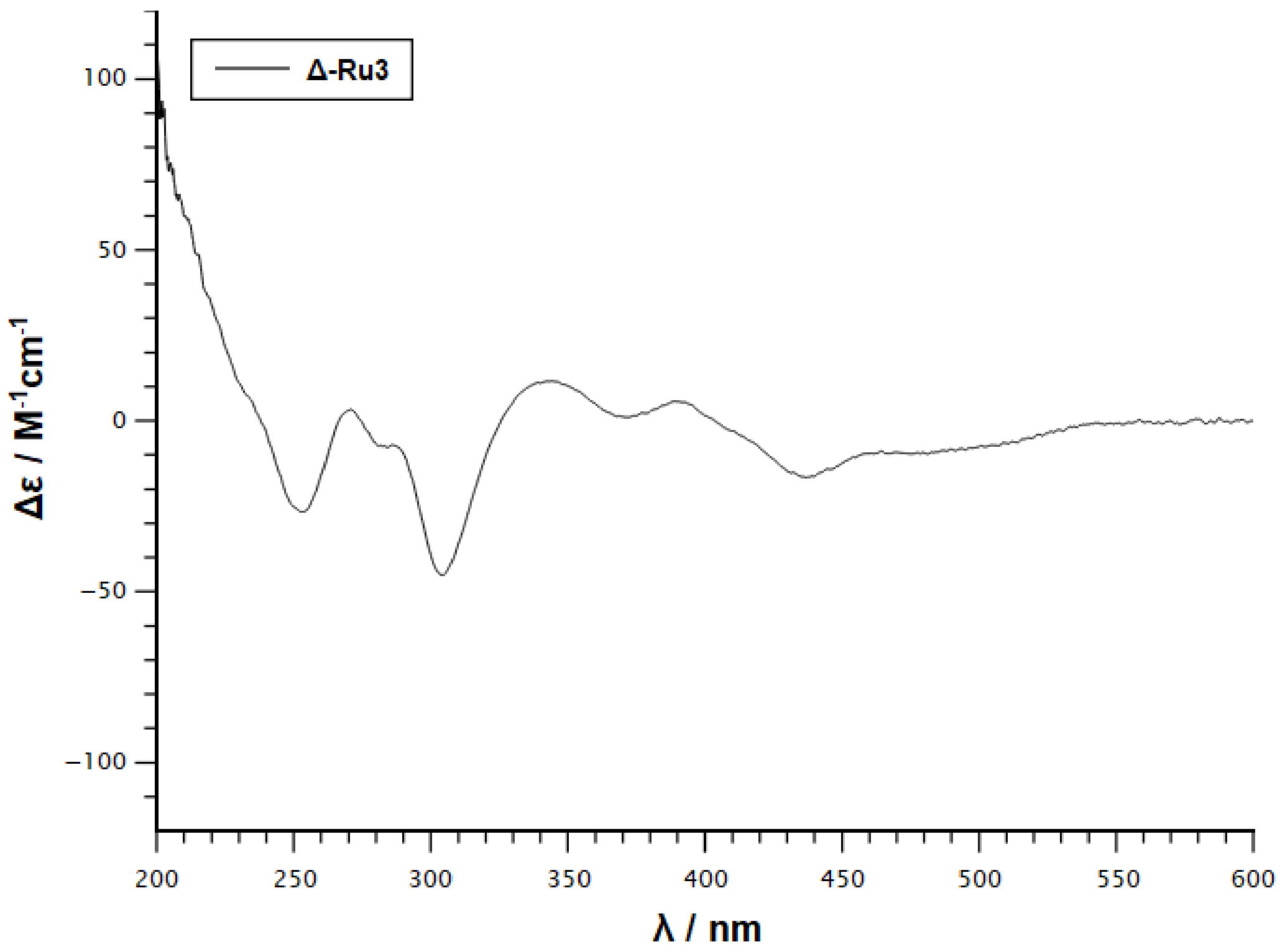

Figure S58: CD spectrum of Δ-**Ru3** in MeCN (0.25 mM).

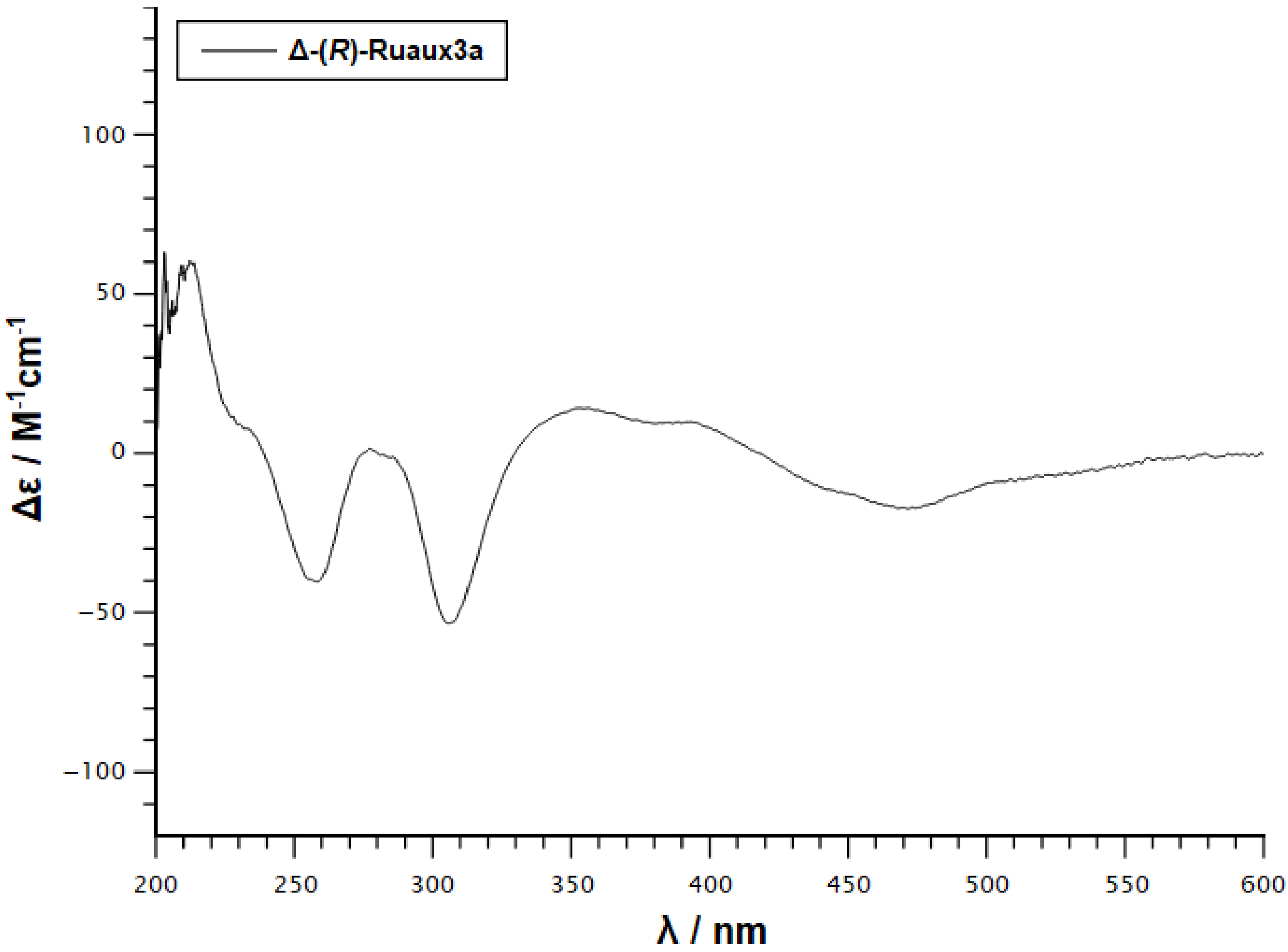

Figure S59: CD spectrum of Δ-(*R*)-**Ruaux3a** in MeCN (0.25 mM).

13. UV-Visible Spectra

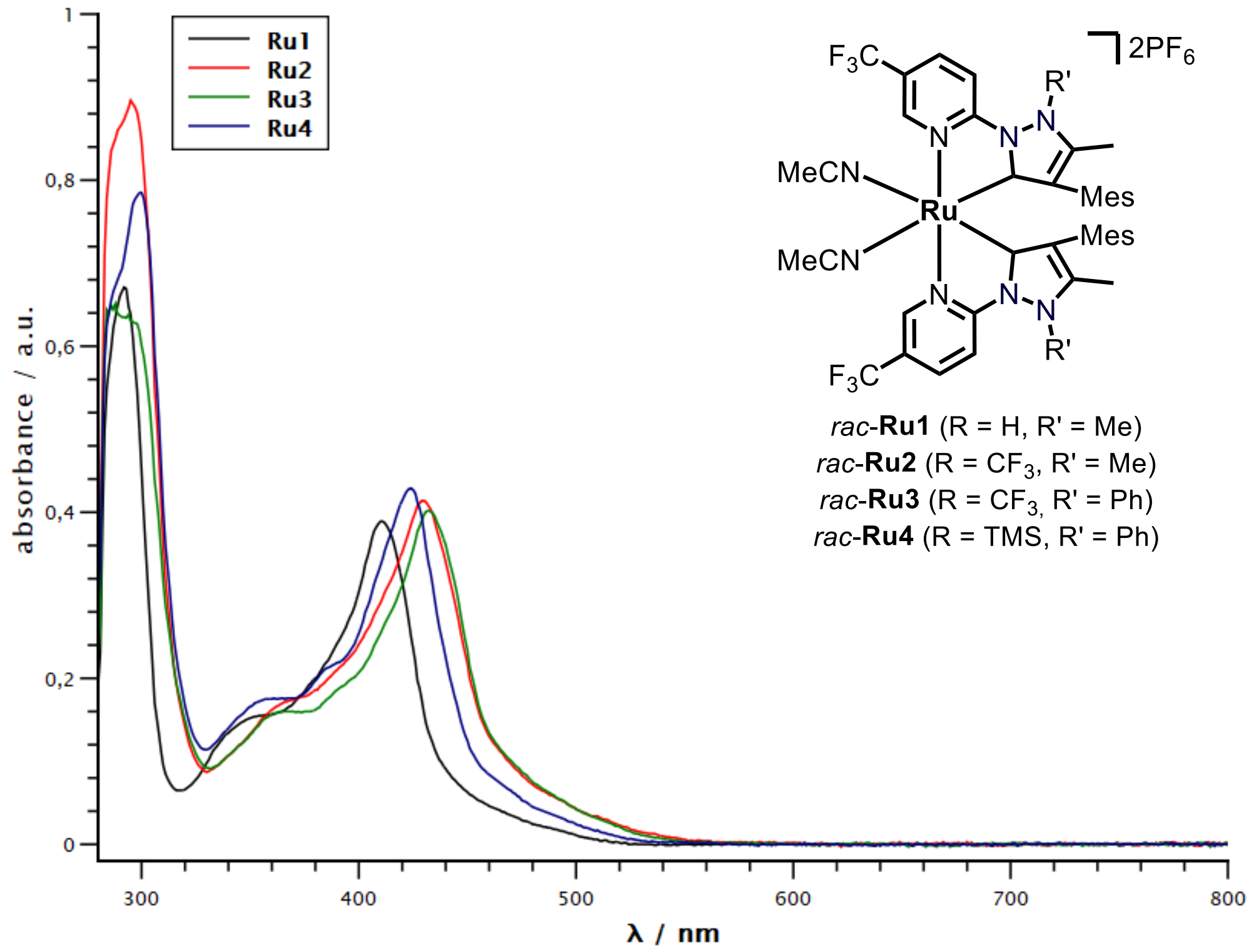

Figure S60: UV-Visible absorbance spectra of the complexes *rac*-**Ru1-4** in MeCN (0.025 mM, 25 °C).

14. Single Crystal X-Ray Diffraction

rac-Ru2

Single crystals were obtained by preparation in an NMR tube. *rac*-**Ru2** was dissolved in a minimal amount of CH_2Cl_2 (0.1 mL) and carefully layered with *n*-pentane. The NMR tube was left standing and room temperature until within two days the formation of single crystals was observed, which were subsequently measured.

A suitable crystal of $C_{44}H_{45}F_6N_8Ru(PF_6)_2 \cdot 2\ CH_2Cl_2$ was selected under inert oil and mounted using a MiTeGen loop. Intensity data of the crystal were recorded with a D8 Venture diffractometer (Bruker AXS). The instrument was operated with Mo-Kα radiation (0.71073 Å, microfocus source) and equipped with a PHOTON III C14 detector. Evaluation, integration and reduction of the diffraction data was carried out using the Bruker APEX 5 software suite.[14] Multi-scan and numerical absorption corrections were applied using the SADABS program.[15,16] The structure was solved using dual-space methods (SHELXT-2018/2) and refined against F2 (SHELXL-2019/1 using ShelXle interface).[17-19] All non-hydrogen atoms were refined with anisotropic displacement parameters. The hydrogen atoms were refined using the “riding model” approach with isotropic displacement parameters 1.2 times (1.5 times for terminal methyl groups) of that of the preceding carbon atom. Both $[PF_6]^-$ anions were refined disordered using the DSR plugin[20] implemented in ShelXle. CCDC 2536211 contains the supplementary crystallographic data for this paper. These data can be obtained free of charge from The Cambridge Crystallographic Data Centre via www.ccdc.cam.ac.uk/structures.

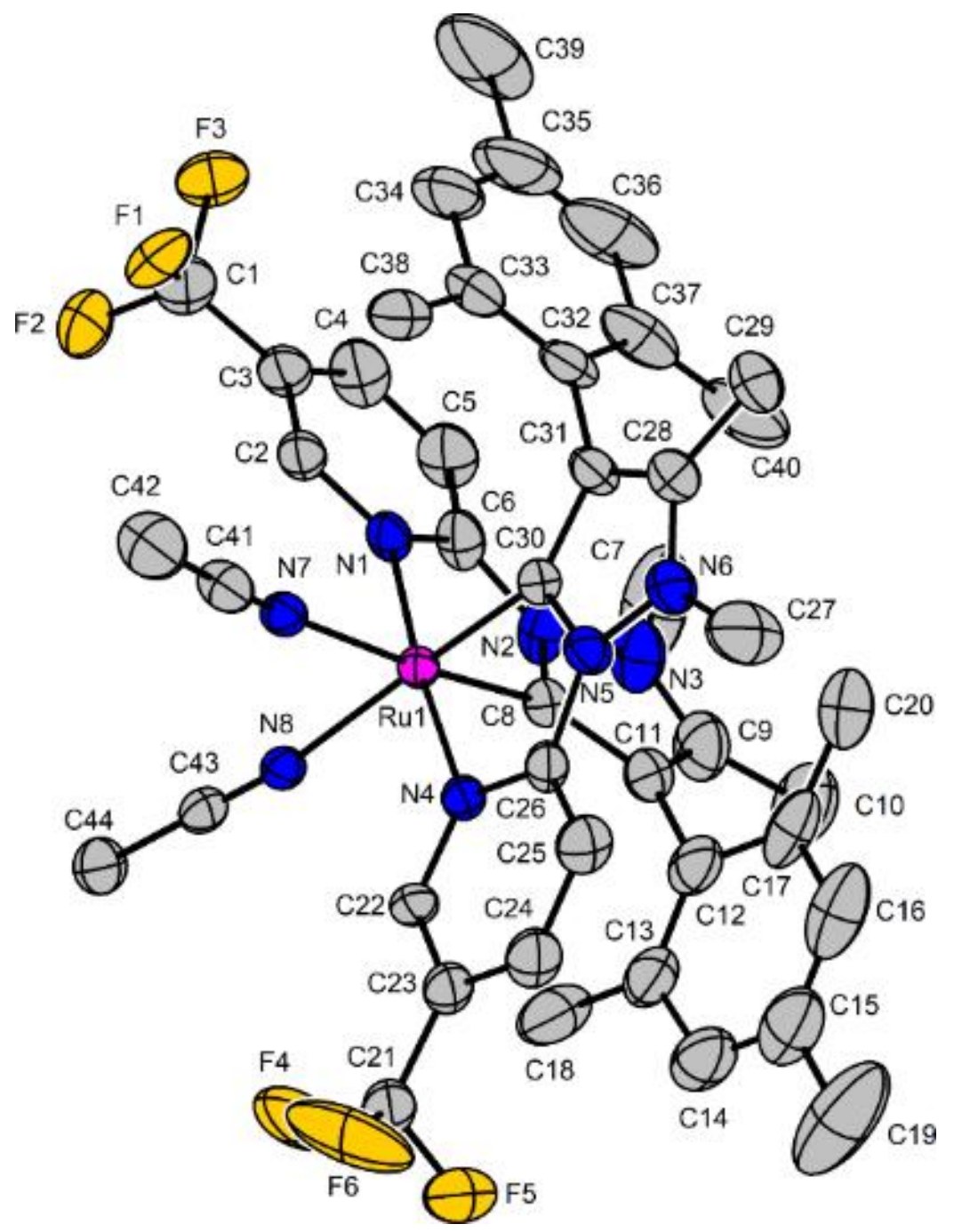

Figure 61: Crystal structure of *rac*-**Ru2**. The PF_6^- anions, the solvent molecules and hydrogen atoms are not shown. Displacement ellipsoids are shown at 50 % probability level at 100 K.

Table S2: Selected crystallographic data and details of the structure determination for $C_{44}H_{45}F_6N_8Ru(PF_6)_2 \cdot 2\ CH_2Cl_2$ (*rac*-**Ru2**).

Identification code	JS22
Empirical formula	$C_{46}H_{49}Cl_4F_{18}N_8P_2Ru$
Molar mass / g·mol^{-1}	1360.74
Space group (No.)	$P2_12_12_1$ (19)
a / Å	15.5121(4)
b / Å	17.1723(4)
c / Å	20.7748(4)
V / Å^3	5534.0(2)
Z	4
$\rho_{calc.}$ / g·cm^{-3}	1.633
μ / mm^{-1}	0.637
Color	orange
Crystal habitus	block
Crystal size / mm^3	0.099 x 0.059 x 0.048
T / K	100
λ / Å	0.71073 (Mo-K$_\alpha$)
θ range / °	1.961 to 28.315
Range of Miller indices	$-20 \leq h \leq 18$
	$-22 \leq k \leq 21$
	$-27 \leq l \leq 27$
Absorption correction	multi-scan and numerical
T_{min}, T_{max}	0.7099, 0.7457
R_{int}, R_σ	0.0408, 0.0212
Completeness of the data set	0.998
No. of measured reflections	147440
No. of independent reflections	13745
No. of parameters	852
No. of restraints	1360
S (all data)	1.099
$R(F)$ ($I \geq 2\sigma(I)$, all data)	0.0558, 0.0588
$wR(F^2)$ ($I \geq 2\sigma(I)$, all data)	0.1443, 0.1464
Extinction coefficient	not refined
Flack parameter x *	0.49(5)
$\Delta\rho_{max}$, $\Delta\rho_{min}$ / e·Å^{-3}	0.730, –1.071

* Refined as inversion twin.

rac-**Ru4**

Single crystals were obtained by preparation in an NMR tube. *rac*-**Ru4** was dissolved in a minimal amount of CH_2Cl_2 (0.1 mL) and carefully layered with *n*-hexane. The NMR tube was left standing and room temperature until within two days the formation of single crystals was observed, which were subsequently measured.

A suitable crystal of $C_{58}H_{68}N_8RuSi_2(PF_6)_2 \cdot 2CH_2Cl_2$ was selected under inert oil and mounted using a MiTeGen loop. Intensity data of the crystal were recorded with a D8 Quest diffractometer (Bruker AXS). The instrument was operated with Mo-Kα radiation (0.71073 Å, microfocus source) and equipped with a PHOTON III C14 detector. Evaluation, integration and reduction of the diffraction data was carried out using the Bruker APEX 5 software suite.[21] Multi-scan absorption correction was applied using the TWINABS program.[22] The structure was solved using dual-space methods (SHELXT-2018/2) and refined against F2 (SHELXL-2019/1 using ShelXle interface).[17-19] All non-hydrogen atoms were refined with anisotropic displacement parameters. The structure was refined as a non-merohedral twin. The hydrogen atoms were refined using the "riding model" approach with isotropic displacement parameters 1.2 times (1.5 times for terminal methyl groups) of that of the preceding carbon atom. The $[PF_6]^-$ anion and one C6-ring were refined disordered using the DSR plugin[20] implemented in ShelXle. CCDC 2536212 contains the supplementary crystallographic data for this paper. These data can be obtained free of charge from The Cambridge Crystallographic Data Centre via www.ccdc.cam.ac.uk/structures

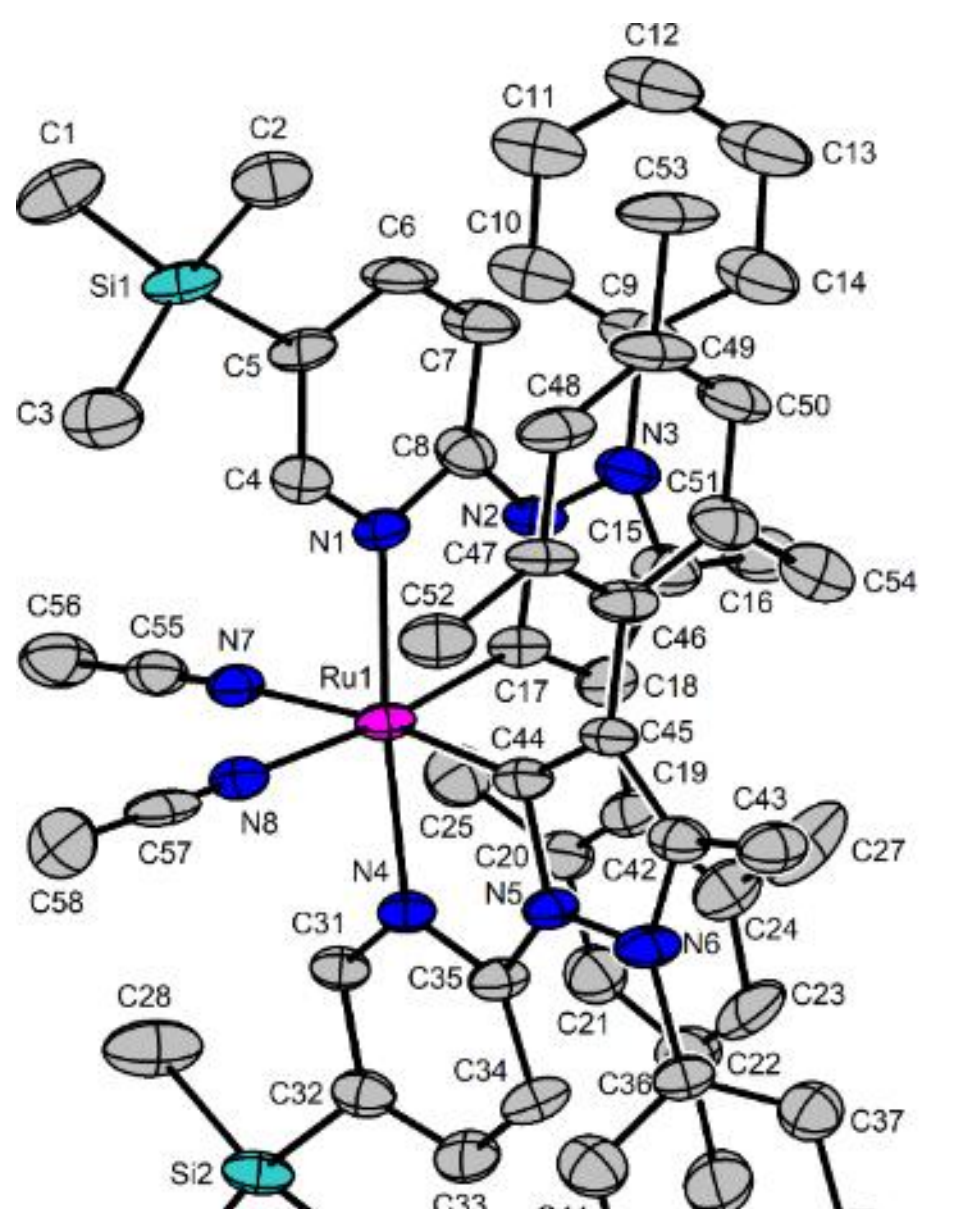

Figure 62: Crystal structure of *rac*-**Ru4**. The PF_6^- anions, the solvent molecules and the hydrogen atoms are not shown. No disorder is shown. Displacement ellipsoids are shown at 50 % probability level at 100 K.

Table S3: Selected crystallographic data and details of the structure determination for $C_{58}H_{68}N_8RuSi_2(PF_6)_2 \cdot 2CH_2Cl_2$ (*rac*-**Ru4**).

Identification code	LiPI19
Empirical formula	$C_{60}H_{72}Cl_4F_{12}N_8P_2RuSi_2$
Molar mass / g·mol^{-1}	1494.24
Space group (No.)	$P\bar{1}$ (2)
a / Å	12.239(4)
b / Å	15.643(6)
c / Å	18.438(6)
α / °	90.221(9)
β / °	99.147(8)
γ / °	100.557(9)
V / Å^3	3424(2)
Z	2
$\rho_{calc.}$ / g·cm^{-3}	1.449
μ / mm^{-1}	0.543
Color	yellow
Crystal habitus	block
Crystal size / mm^3	0.190 x 0.099 x 0.034
T / K	100
λ / Å	0.71073 (Mo-K$_\alpha$)
θ range / °	2.141 to 25.074
Range of Miller indices	$-14 \le h \le 14$
	$-18 \le k \le 18$
	$-21 \le l \le 21$
Absorption correction	multi-scan
T_{min}, T_{max}	0.59846, 0.74516
R_{int}, R_σ	0.0772, 0.0600
Completeness of the data set	0.976
No. of measured reflections	65041
No. of independent reflections	11948
No. of parameters	938
No. of restraints	682
S (all data)	1.046
$R(F)$ ($I \ge 2\sigma(I)$, all data)	0.0837, 0.1004
$wR(F^2)$ ($I \ge 2\sigma(I)$, all data)	0.1974, 0.2108
Extinction coefficient	not refined
$\Delta\rho_{max}$, $\Delta\rho_{min}$ / e·Å^{-3}	1.915, –1.065

Δ-(*S*)-**RuAux3b**

Single crystals were obtained by preparation in an NMR tube. A sample of Δ-(*S*)-**RuAux3b** in CD_2Cl_2, which was obtained from **Δ-Ru3** within the ee determination experiments, was concentrated to a volume of 0.1 mL and carefully layered with *n*-hexane. The NMR tube was left standing and room temperature until within two days the formation of single crystals was observed, which were subsequently measured.

A suitable crystal of $C_{60}H_{53}F_7N_7O_2Ru(PF_6)$ was selected under inert oil and mounted using a MiTeGen loop. Intensity data of the crystal were recorded with a D8 Quest diffractometer (Bruker AXS). The instrument was operated with Mo-Kα radiation (0.71073 Å, microfocus source) and equipped with a PHOTON III C14 detector. Evaluation, integration and reduction of the diffraction data was carried out using the Bruker APEX 5 software suite.[14] Multi-scan and numerical absorption corrections were applied using the SADABS program.[15,16] The structure was solved using dual-space methods (SHELXT-2018/2) and refined against F^2 (SHELXL-2019/1 using ShelXle interface).[17-19] All non-hydrogen atoms were refined with anisotropic displacement parameters. The hydrogen atoms were refined using the “riding model” approach with isotropic displacement parameters 1.2 times (1.5 times for terminal methyl groups) of that of the preceding carbon atom. CCDC 2536213 contains the supplementary crystallographic data for this paper. These data can be obtained free of charge from The Cambridge Crystallographic Data Centre via www.ccdc.cam.ac.uk/structures.

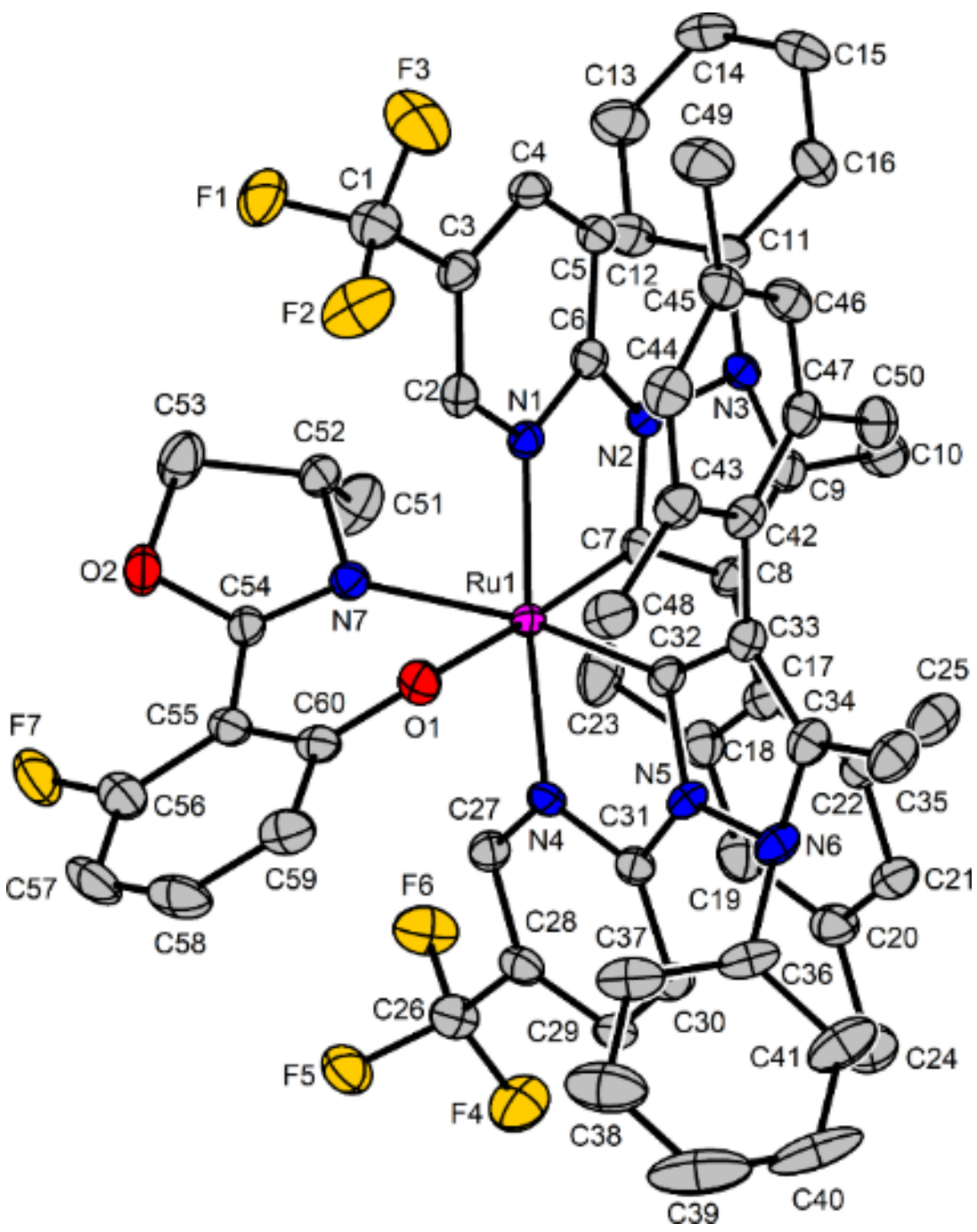

Figure 63: Crystal structure of Δ-(*S*)-**RuAux3b**. The PF_6^- anion and the hydrogen atoms are not shown. Displacement ellipsoids are shown at 50 % probability level at 100 K.

Table S4: Selected crystallographic data and details of the structure determination for $C_{60}H_{53}F_7N_7O_2Ru(PF_6)$ (Δ-(*S*)-**RuAux3b**).

Identification code	MFP372ee
Empirical formula	$C_{60}H_{53}F_{13}N_7O_2PRu$
Molar mass / g·mol^{-1}	1283.13
Space group (No.)	$P2_12_12_1$ (19)
a / Å	14.5516(5)
b / Å	17.3627(6)
c / Å	22.2126(8)
V / Å^3	5612.1(3)
Z	4
$\rho_{calc.}$ / g·cm^{-3}	1.519
μ / mm^{-1}	0.402
Color	red
Crystal habitus	needle
Crystal size / mm^3	0.382 x 0.065 x 0.061
T / K	100
λ / Å	0.71073 (Mo-K$_\alpha$)
θ range / °	2.043 to 28.312
Range of Miller indices	$-19 \leq h \leq 19$
	$-22 \leq k \leq 23$
	$-29 \leq l \leq 29$
Absorption correction	multi-scan and numerical
T_{min}, T_{max}	0.9115, 1.0000
R_{int}, R_σ	0.0504, 0.0349
Completeness of the data set	0.999
No. of measured reflections	93430
No. of independent reflections	13905
No. of parameters	766
No. of restraints	0
S (all data)	1.083
$R(F)$ ($I \geq 2\sigma(I)$, all data)	0.0349, 0.0401
$wR(F^2)$ ($I \geq 2\sigma(I)$, all data)	0.0670, 0.0687
Extinction coefficient	not refined
Flack parameter *x*	–0.009(7)
$\Delta\rho_{max}$, $\Delta\rho_{min}$ / e·Å^{-3}	0.425, –0.575

(*S*,*S*)-**6**

Single crystals were obtained by preparation in an NMR tube. A sample of (*S*,*S*)-**6** in $CDCl_3$, which was obtained in a catalytic reaction with Δ-**Ru3** as catalyst, was concentrated to a volume of 0.1 mL and carefully layered with *n*-hexane. The NMR tube was left standing and room temperature until within two days the formation of single crystals was observed, which were subsequently measured.

A suitable crystal of $C_{12}H_{14}N_2O$ was selected under inert oil and mounted using a MiTeGen loop. Intensity data of the crystal were recorded with a D8 Venture diffractometer (Bruker AXS). The instrument was operated with Cu-Kα radiation (1.54178 Å, microfocus source) and equipped with a PHOTON III C14 detector. Evaluation, integration and reduction of the diffraction data was carried out using the Bruker APEX 5 software suite.[14] Multi-scan and numerical absorption corrections were applied using the SADABS program.[15,16] The structure was solved using dual-space methods (SHELXT-2018/2) and refined against F^2 (SHELXL-2019/1 using ShelXle interface).[17-19] All non-hydrogen atoms were refined with anisotropic displacement parameters. The hydrogen atoms were refined using the "riding model" approach with isotropic displacement parameters 1.2 times (1.5 times for terminal methyl groups) of that of the preceding carbon atom. CCDC 2536214 contains the supplementary crystallographic data for this paper. These data can be obtained free of charge from The Cambridge Crystallographic Data Centre via www.ccdc.cam.ac.uk/structures.

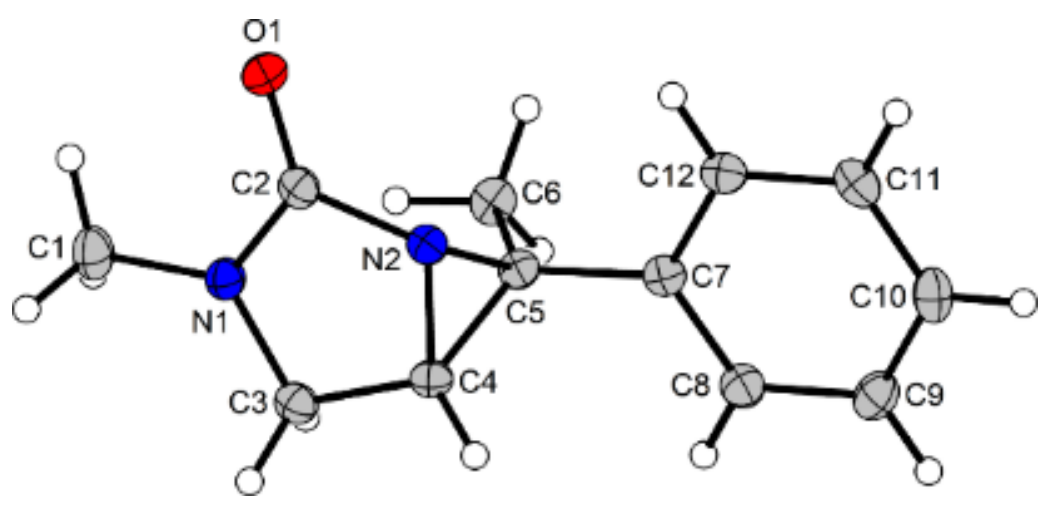

Figure 64: Crystal structure of (*S*,*S*)-**6**. Displacement ellipsoids are shown at 50 % probability level at 100 K. The hydrogen atoms are shown with arbitrary radii.

Table S5: Selected crystallographic data and details of the structure determination for $C_{12}H_{14}N_2O$ ((*S*,*S*)-**6**).

Identification code	MFP399
Empirical formula	$C_{12}H_{14}N_2O$
Molar mass / g·mol^{-1}	202.25
Space group (No.)	$P2_12_12_1$ (19)
a / Å	5.62410(10)
b / Å	10.5816(3)
c / Å	17.5371(4)
V / Å^3	1043.67(4)
Z	4
$\rho_{calc.}$ / g·cm^{-3}	1.287
μ / mm^{-1}	0.666
Color	colorless
Crystal habitus	needle
Crystal size / mm^3	0.364 x 0.096 x 0.059
T / K	100
λ / Å	1.54178 (Cu-K$_\alpha$)
θ range / °	4.881 to 74.473
Range of Miller indices	$-7 \leq h \leq 6$
	$-13 \leq k \leq 13$
	$-20 \leq l \leq 21$
Absorption correction	multi-scan and numerical
T_{min}, T_{max}	0.8211, 1.0000
R_{int}, R_σ	0.0315, 0.0121
Completeness of the data set	1.000
No. of measured reflections	28651
No. of independent reflections	2121
No. of parameters	139
No. of restraints	0
S (all data)	1.050
$R(F)$ ($I \geq 2\sigma(I)$, all data)	0.0238, 0.0245
$wR(F^2)$ ($I \geq 2\sigma(I)$, all data)	0.0614, 0.0621
Extinction coefficient	0.0044(7)
Flack parameter x	0.06(6)
$\Delta\rho_{max}$, $\Delta\rho_{min}$ / e·Å^{-3}	0.155, –0.126

15. References

1. X. Liu, Y. Yan, Y. Wang, C. Wang and J. Sun, "Highly Enantioselective Reduction of β-Amino Nitroolefins with a Simple N-Sulfinyl Urea as Bifunctional Catalyst" *Chemistry – A European Journal* **18**, (2012): 9204–9207.
2. D. Franco, M. Gómez, F. Jiménez, G. Muller, M. Rocamora, M. A. Maestro and J. Mahía, "Exo- and Endocyclic Oxazolinyl−Phosphane Palladium Complexes: Catalytic Behavior in Allylic Alkylation Processes" *Organometallics* **23**, (2004): 3197–3209.
3. Y. Sato, Y. Miyamoto, T. Matsui, Y. Sumida and H. Ohmiya, "Diastereoselective congested b-amido ketone synthesis via NHC-catalyzed radical-radical coupling" *Chem Catal*ysis **3**, (2023): 100736.
4. M. Bielawski and B. Olofsson, "EFFICIENT ONE-POT SYNTHESIS OF BIS(4-TERT-BUTYLPHENYL)IODONIUM TRIFLATE" *Organic Synthesis* **86**, (2009): 308.
5. V. Bagutski, A. Ros and V. K. Aggarwal, "Improved method for the conversion of pinacolboronic esters into trifluoroborate salts: facile synthesis of chiral secondary and tertiary trifluoroborates" *Tetrahedron* **65**, (2009): 9956-9960.
6. Y. Zheng, Y. Tan, K. Harms, M. Marsch, R. Riedel, L. Zhang and E. Meggers, "Octahedral Ruthenium Complex with Exclusive Metal-Centered Chirality for Highly Effective Asymmetric Catalysis" *Journal of the American Chemical Society* **139**, (2017): 4322−4325.
7. F. Stange, S. Tokura and M. Kira, "Ruthenium and rhenium complexes with silyl-substituted bipyridyl ligands" *Journal of Organometallic Chemistry* **612**, (2000): 117–124.
8. N. Demirel, P. Moths, X. Xie, S. I. Ivlev and E. Meggers, "Development of Chiral-At-Ruthenium Mesoionic Carbene Catalysts" *Chemistry – A European Journal* **31**, (2025): e202403792.
9. K. Verlinden, H. Buhl, W. Frank and C. Ganter, "Determining the Ligand Properties of N-Heterocyclic Carbenes from 77Se NMR Parameters" *European Journal of Inorganic Chemistry* **2015**, (2015): 2416–2425.
10. G. Meng, L. Kakalis, S. P. Nolan and M. Szostak, "A simple ^{1}H NMR method for determining the σ-donor properties of N-heterocyclic carbenes" *Tetrahedron Letters* **60**, (2019): 378–381.
11. N. Demirel, M. Dawor, G. Nadler, S. I. Ivlev and E. Meggers, "Stereogenic-at-iron mesoionic carbene complex for enantioselective C–H amidation" *Chemical Science* **15**, (2024): 15625–15631.
12. X. Ren, Q. Guo, J. Chen, H. Xie, Q. Xu and Z. Lu, "Visible-Light Promoted Distereodivergent Intramolecular Oxyamidation of Alkenes" *Chemistry – A European Journal* **22**, (2016): 18695–18699.

13. L. Jarrige, Z. Zhou, M. Hemming and E. Meggers, "Efficient Amination of Activated and Non-Activated C(sp^3)−H Bonds with a Simple Iron–Phenanthroline Catalyst" *Angewandte Chemie International Edition* **60**, (2021): 6314–6319.
14. *APEX5*, Bruker AXS Inc., Madison, Wisconsin, USA, (2023).
15. *SADABS*, Bruker AXS Inc., Madison, Wisconsin, USA, (2016).
16. L. Krause, R. Herbst-Irmer, G. M. Sheldrick and D. Stalke, "Comparison of silver and molybdenum microfocus X-ray sources for single-crystal structure determination" *Journal of Applied Crystallography* **48**, (2015): 3–10.
17. G. M. Sheldrick, "SHELXT – Integrated space-group and crystalstructure determination" *Acta Crystallographica Section A, Foundations and Advances* ***71,*** (2015): 3–8.
18. G. M. Sheldrick, "Crystal structure refinement with SHELXL" *Acta Crystallographica Section C, Structural Chemistry* **71**, (2015): 3–8.
19. C. B. Hübschle, G. M. Sheldrick and B. Dittrich, "ShelXle: a Qt graphical user interface for SHELXL" *Journal of Applied Crystallography* **44**, (2011): 1281–1284.
20. D. Kratzert and I. Krossing, "Recent improvements in DSR" *Journal of Applied Crystallography* **51**, (2018): 928–934.
21. *APEX3 v2019.11-2*, Bruker AXS Inc., Madison, Wisconsin, USA, (2019).
22. *TWINABS*, Bruker AXS Inc., Madison, Wisconsin, USA, (2012).
