# Supplementary material for: Chiral Pyrazolinylidene Complexes for Asymmetric Catalysis
Source: Chemistry. 2026 Jul 12;32(39):e71426. doi: 10.1002/chem.71426 (PMC13715465; doi:10.1002/chem.71426)

checkCIF/PLATON report

Structure factors have been supplied for datablock(s) MFP399

THIS REPORT IS FOR GUIDANCE ONLY. IF USED AS PART OF A REVIEW PROCEDURE FOR PUBLICATION, IT SHOULD NOT REPLACE THE EXPERTISE OF AN EXPERIENCED CRYSTALLOGRAPHIC REFEREE.

No syntax errors found. CIF dictionary Interpreting this report

Datablock: MFP399

Bond precision: C-C = 0.0019 A Wavelength=1.54178

Cell:	a=5.6241(1)	b=10.5816(3)	c=17.5371(4)
	alpha=90	beta=90	gamma=90
Temperature:	100 K		

	Calculated	Reported
Volume	1043.67(4)	1043.67(4)
Space group	P 21 21 21	P 21 21 21
Hall group	P 2ac 2ab	P 2ac 2ab
Moiety formula	C12 H14 N2 O	C12 H14 N2 O
Sum formula	C12 H14 N2 O	C12 H14 N2 O
Mr	202.25	202.25
Dx,g cm-3	1.287	1.287
Z	4	4
Mu (mm-1)	0.666	0.666
F000	432.0	432.0
F000’	433.23	
h,k,lmax	7,13,21	7,13,21
Nref	2122[1261]	2121
Tmin,Tmax	0.926,0.961	0.821,1.000
Tmin’	0.785	

Correction method= # Reported T Limits: Tmin=0.821 Tmax=1.000
AbsCorr = MULTI-SCAN

```
Data completeness= 1.68/1.00       Theta(max)= 74.473

R(reflections)= 0.0238( 2076)       wR2(reflections)= 0.0621( 2121)

S = 1.050                 Npar= 139
```

```
The following ALERTS were generated. Each ALERT has the format
       test-name_ALERT_alert-type_alert-level.
Click on the hyperlinks for more details of the test.
```

Alert level G

```
PLAT791_ALERT_4_G Model has Chirality at C4         (Sohncke SpGr)          S Verify
PLAT791_ALERT_4_G Model has Chirality at C5         (Sohncke SpGr)          S Verify
PLAT969_ALERT_5_G The 'Henn et al.' R-Factor-gap value ..........      4.689 Note
              Predicted wR2: Based on SigI**2  1.32 or SHELX Weight  5.91
PLAT978_ALERT_2_G Number C-C Bonds with Positive Residual Density.          6 Info
PLAT994_ALERT_1_G SHELXL .ins Contains no or MERG 0 Instruction ..          ! Note
```

```
   0 ALERT level A = Most likely a serious problem - resolve or explain
   0 ALERT level B = A potentially serious problem, consider carefully
   0 ALERT level C = Check. Ensure it is not caused by an omission or oversight
   5 ALERT level G = General information/check it is not something unexpected

   1 ALERT type 1 CIF construction/syntax error, inconsistent or missing data
   1 ALERT type 2 Indicator that the structure model may be wrong or deficient
   0 ALERT type 3 Indicator that the structure quality may be low
   2 ALERT type 4 Improvement, methodology, query or suggestion
   1 ALERT type 5 Informative message, check
```

It is advisable to attempt to resolve as many as possible of the alerts in all categories. Often the minor alerts point to easily fixed oversights, errors and omissions in your CIF or refinement strategy, so attention to these fine details can be worthwhile. It is up to the individual to critically assess their own results and, if necessary, seek expert advice.

PLATON version of 15/01/2026; check.def file version of 02/01/2026

---

duplicate check

No duplication found

Datablock MFP399 - ellipsoid plot

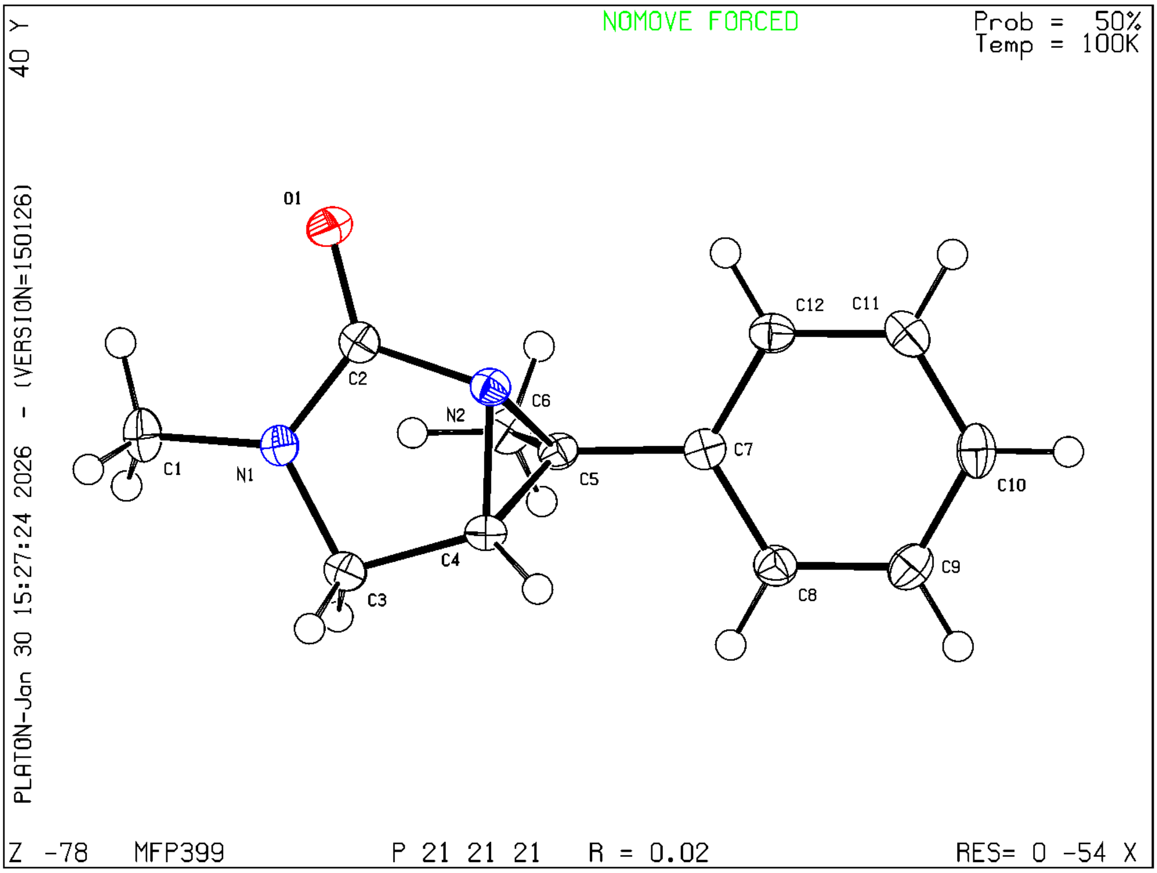

Supplement: Supplementary file 2 [file CHEM-32-e71426-s001.zip › Aziridine 6_checkcif_2.pdf]
