# Supplementary material for: Chiral Pyrazolinylidene Complexes for Asymmetric Catalysis
Source: Chemistry. 2026 Jul 12;32(39):e71426. doi: 10.1002/chem.71426 (PMC13715465; doi:10.1002/chem.71426)

checkCIF/PLATON report

Structure factors have been supplied for datablock(s) MFP372ee

THIS REPORT IS FOR GUIDANCE ONLY. IF USED AS PART OF A REVIEW PROCEDURE FOR PUBLICATION, IT SHOULD NOT REPLACE THE EXPERTISE OF AN EXPERIENCED CRYSTALLOGRAPHIC REFEREE.

No syntax errors found. CIF dictionary Interpreting this report

Datablock: MFP372ee

```
Bond precision:   C-C = 0.0050 A          Wavelength=0.71073

Cell:              a=14.5516(5)      b=17.3627(6)      c=22.2126(8)
                   alpha=90          beta=90           gamma=90
Temperature:       100 K
```

	Calculated	Reported
Volume	5612.1(3)	5612.1(3)
Space group	P 21 21 21	P 21 21 21
Hall group	P 2ac 2ab	P 2ac 2ab
Moiety formula	C60 H53 F7 N7 O2 Ru, F6 P	C60 H53 F7 N7 O2 Ru, F6 P
Sum formula	C60 H53 F13 N7 O2 P Ru	C60 H53 F13 N7 O2 P Ru
Mr	1283.14	1283.13
Dx,g cm-3	1.519	1.519
Z	4	4
Mu (mm-1)	0.402	0.402
F000	2616.0	2616.0
F000'	2612.90	
h,k,lmax	19,23,29	19,23,29
Nref	13983[7648]	13905
Tmin,Tmax	0.969,0.976	0.911,1.000
Tmin'	0.858	

```
Correction method= # Reported T Limits: Tmin=0.911 Tmax=1.000
AbsCorr = MULTI-SCAN
```

Data completeness= 1.82/0.99   Theta(max)= 28.312

R(reflections)= 0.0349(12943)   wR2(reflections)= 0.0687(13905)

S = 1.083   Npar= 766

The following ALERTS were generated. Each ALERT has the format
test-name_ALERT_alert-type_alert-level.
Click on the hyperlinks for more details of the test.

Alert level C

```
PLAT220_ALERT_2_C NonSolvent   Resd 1   C   Ueq(max)/Ueq(min)  Range        4.1 Ratio
```

Author Response: Due to CH3 groups having larger displacement ellipsoids.

```
PLAT911_ALERT_3_C Missing FCF Refl Between Thmin & STh/L=    0.600           4 Report
               15   9  0,    0  1  2,    0  3  6,    1 17 15,
```

Author Response: A few reflections could not be properly collected using the selected measurement strategy and the instrument.

Alert level G

```
PLAT242_ALERT_2_G Low   MainResAtom  Ueq as Compared to Neighbours         C1 Check
PLAT242_ALERT_2_G Low   MainResAtom  Ueq as Compared to Neighbours        C26 Check
PLAT244_ALERT_4_G Low  MinorResAtom  Ueq as Compared to Neighbours         P1 Check
PLAT380_ALERT_4_G Incorrectly? Oriented X(sp2)-Methyl Moiety .....        C24 Check
PLAT380_ALERT_4_G Incorrectly? Oriented X(sp2)-Methyl Moiety .....        C35 Check
PLAT398_ALERT_2_G Deviating  C-O-C   Angle From 120 for O2       .      106.9 Degree
PLAT432_ALERT_2_G Short Inter X...Y Contact  F7       ..C4       .       2.86 Ang.
                                        1/2+x,1/2-y,1-z  =      4_556 Check
PLAT432_ALERT_2_G Short Inter X...Y Contact  O2       ..C29      .       2.99 Ang.
                                       -1/2+x,1/2-y,1-z  =      4_456 Check
PLAT791_ALERT_4_G Model has Chirality at C52        (Sohncke SpGr)          S Verify
PLAT910_ALERT_3_G Missing FCF Reflection(s) Below Theta(Min)[Deg]=       2.04 Note
                1  1  0,   1  0  1,   0  1  1,   0  0  2,
PLAT912_ALERT_4_G Missing # of FCF Reflections Above STh/L=  0.600          4 Note
PLAT913_ALERT_3_G Missing # of Very Strong Reflections in FCF ....          1 Note
                0  1  2,
PLAT933_ALERT_2_G Number of HKL-OMIT Records in Embedded .res File          2 Note
                0  1  2,   0  3  6,
PLAT965_ALERT_2_G The SHELXL WEIGHT Optimisation has not Converged     Please Check
PLAT969_ALERT_5_G The 'Henn et al.' R-Factor-gap value ..........      2.563 Note
              Predicted wR2: Based on SigI**2  2.68 or SHELX Weight  6.34
PLAT978_ALERT_2_G Number C-C Bonds with Positive Residual Density.          4 Info
PLAT994_ALERT_1_G SHELXL .ins Contains no or MERG 0 Instruction ..          ! Note
```

---

0 **ALERT level A** = Most likely a serious problem - resolve or explain

```
 0 ALERT level B = A potentially serious problem, consider carefully
 2 ALERT level C = Check. Ensure it is not caused by an omission or oversight
17 ALERT level G = General information/check it is not something unexpected

 1 ALERT type 1 CIF construction/syntax error, inconsistent or missing data
 9 ALERT type 2 Indicator that the structure model may be wrong or deficient
 3 ALERT type 3 Indicator that the structure quality may be low
 5 ALERT type 4 Improvement, methodology, query or suggestion
 1 ALERT type 5 Informative message, check
```

It is advisable to attempt to resolve as many as possible of the alerts in all categories. Often the minor alerts point to easily fixed oversights, errors and omissions in your CIF or refinement strategy, so attention to these fine details can be worthwhile. It is up to the individual to critically assess their own results and, if necessary, seek expert advice.

PLATON version of 15/01/2026; check.def file version of 02/01/2026

---

duplicate check

No duplication found

Datablock MFP372ee - ellipsoid plot

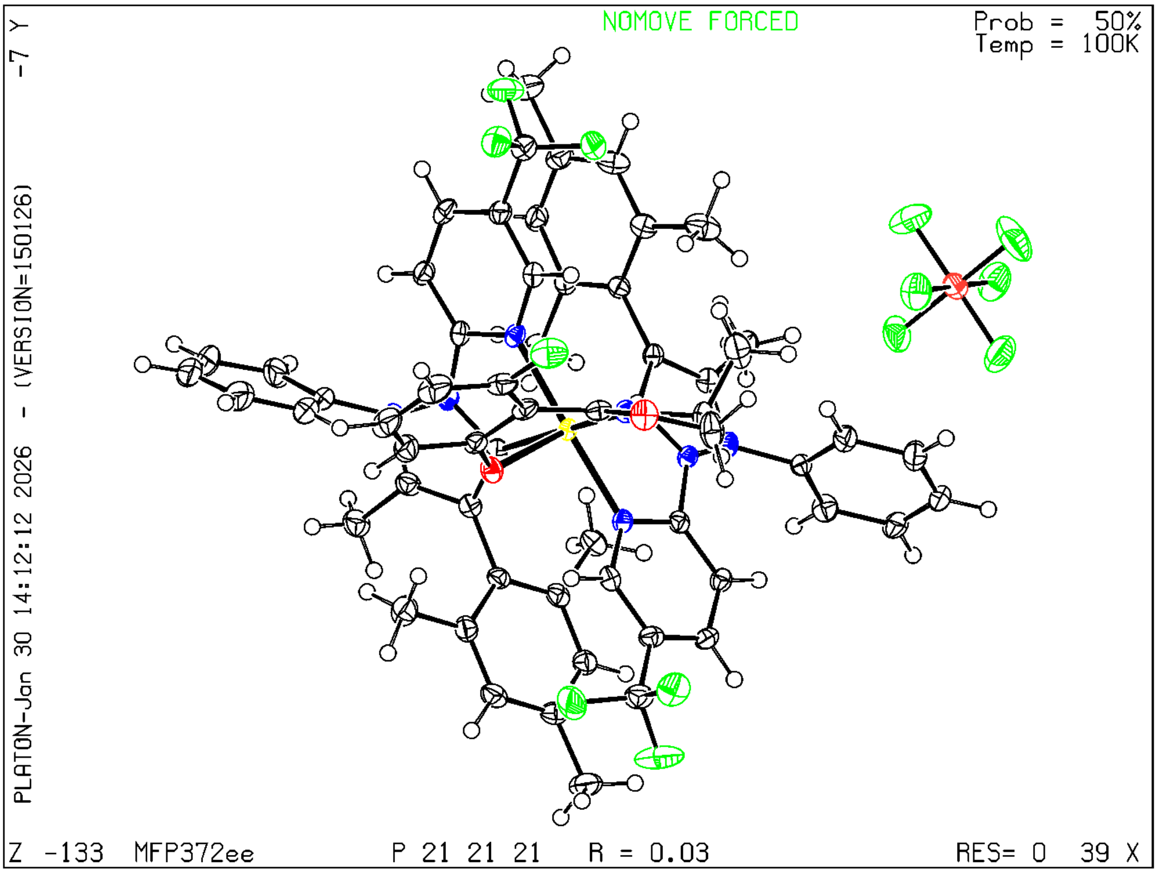

Supplement: Supplementary file 2 [file CHEM-32-e71426-s001.zip › delta-S-RuAux3b_checkcif_2.pdf]
