# Supplementary material for: Chiral Pyrazolinylidene Complexes for Asymmetric Catalysis
Source: Chemistry. 2026 Jul 12;32(39):e71426. doi: 10.1002/chem.71426 (PMC13715465; doi:10.1002/chem.71426)

checkCIF/PLATON report

Structure factors have been supplied for datablock(s) JS22

THIS REPORT IS FOR GUIDANCE ONLY. IF USED AS PART OF A REVIEW PROCEDURE FOR PUBLICATION, IT SHOULD NOT REPLACE THE EXPERTISE OF AN EXPERIENCED CRYSTALLOGRAPHIC REFEREE.

No syntax errors found. CIF dictionary Interpreting this report

Datablock: JS22

Bond precision: C-C = 0.0131 A Wavelength=0.71073

Cell:	a=15.5121(4)	b=17.1723(4)	c=20.7748(4)
	alpha=90	beta=90	gamma=90
Temperature:	100 K		

	Calculated	Reported
Volume	5534.0(2)	5534.0(2)
Space group	P 21 21 21	P 21 21 21
Hall group	P 2ac 2ab	P 2ac 2ab
Moiety formula	C44 H45 F6 N8 Ru, 2(F6 P), 2(C H2 Cl2)	C44 H45 F6 N8 Ru, 2(F6 P), 2(C H2 Cl2)
Sum formula	C46 H49 Cl4 F18 N8 P2 Ru	C46 H49 Cl4 F18 N8 P2 Ru
Mr	1360.74	1360.74
Dx,g cm-3	1.633	1.633
Z	4	4
Mu (mm-1)	0.637	0.637
F000	2740.0	2740.0
F000'	2739.80	
h,k,lmax	20,22,27	20,22,27
Nref	13770[7526]	13745
Tmin,Tmax	0.956,0.970	0.710,0.746
Tmin'	0.939	

Correction method= # Reported T Limits: Tmin=0.710 Tmax=0.746
AbsCorr = MULTI-SCAN

Data completeness= 1.83/1.00 Theta(max)= 28.315

R(reflections)= 0.0558(13048) wR2(reflections)= 0.1464(13745)

S = 1.103 Npar= 852

The following ALERTS were generated. Each ALERT has the format
test-name_ALERT_alert-type_alert-level.
Click on the hyperlinks for more details of the test.

Alert level C

STRVA01_ALERT_4_C Flack test results are ambiguous.
From the CIF: _refine_ls_abs_structure_Flack 0.490
From the CIF: _refine_ls_abs_structure_Flack_su 0.050

Author Response: Refined as an inversion twin.

PLAT213_ALERT_2_C Atom F3 has ADP max/min Ratio ..... 3.1 prolat

Author Response: Disordered PF6- anions. Other groups also have elongated displacement ellipsoids indicating a possibility of mild disorder.

PLAT213_ALERT_2_C Atom F6 has ADP max/min Ratio ..... 3.3 prolat

Author Response: Disordered PF6- anions. Other groups also have elongated displacement ellipsoids indicating a possibility of mild disorder.

PLAT213_ALERT_2_C Atom C39 has ADP max/min Ratio ..... 3.5 prolat

Author Response: Disordered PF6- anions. Other groups also have elongated displacement ellipsoids indicating a possibility of mild disorder.

PLAT220_ALERT_2_C NonSolvent Resd 1 C Ueq(max)/Ueq(min) Range 5.8 Ratio

Author Response: Disordered PF6- anions. Other groups also have elongated displacement ellipsoids indicating a possibility of mild disorder.

PLAT222_ALERT_3_C NonSolvent Resd 1 H Uiso(max)/Uiso(min) Range 7.0 Ratio

Author Response: Disordered PF6- anions. Other groups also have elongated displacement ellipsoids indicating a possibility of mild disorder.

PLAT230_ALERT_2_C Hirshfeld Test Diff for F1 --C1 . 5.7 s.u.

Author Response: Likely due to disorder.

PLAT234_ALERT_4_C Large Hirshfeld Difference C34 --C35 . 0.16 Ang.

Author Response: Likely due to disorder.

PLAT242_ALERT_2_C Low   'MainMol' Ueq as Compared to Neighbors of   N3 Check

Author Response: Likely due to disorder.

PLAT242_ALERT_2_C Low   'MainMol' Ueq as Compared to Neighbors of   C15 Check

Author Response: Likely due to disorder.

PLAT250_ALERT_2_C Large U3/U1 Ratio for <U(i,j)> Tensor(Resd   2)   2.6 Note

Author Response: Likely due to disorder.

PLAT250_ALERT_2_C Large U3/U1 Ratio for <U(i,j)> Tensor(Resd   4)   2.9 Note

Author Response: Likely due to disorder.

PLAT250_ALERT_2_C Large U3/U1 Ratio for <U(i,j)> Tensor(Resd   5)   2.5 Note

Author Response: Likely due to disorder.

PLAT260_ALERT_2_C Large Average Ueq of Residue Including   P1_2   0.101 Check

Author Response: Disordered PF6- anions.

PLAT260_ALERT_2_C Large Average Ueq of Residue Including   P1_4   0.130 Check

Author Response: Disordered PF6- anions.

PLAT342_ALERT_3_C Low Bond Precision on C-C Bonds ....   0.01305 Ang.

Author Response: Due to disorder.

PLAT413_ALERT_2_C Short Inter XH3 .. XHn   H10C   ..H19C   .   2.00 Ang.
1/2+x,1/2-y,1-z =   4_556 Check

Author Response: Hydrogen atoms refined using riding model, which not always produces correct results when carbon atoms are located in the vicinity of each other.

PLAT911_ALERT_3_C Missing FCF Refl Between Thmin & STh/L=   0.600   6 Report
1 0 3,   0 2 4,   1 0 5,   0 0 6,   0 2 7,   3 0 13,

Author Response: A few reflections were excluded from the refinement due to high systematic errors.

Alert level G

```
PLAT002_ALERT_2_G Number of Distance or Angle Restraints on AtSite         28 Note
PLAT003_ALERT_2_G Number of Uiso or U(i,j)  Restrained non-H-Atoms         28 Report
PLAT033_ALERT_4_G Flack x Value Deviates > 3.0 * Sigma from Zero .      0.490 Note
PLAT083_ALERT_2_G SHELXL Second Parameter in WGHT  Unusually Large      13.40 Why ?
PLAT175_ALERT_4_G The CIF-Embedded .res File Contains SAME Records          1 Report
PLAT176_ALERT_4_G The CIF-Embedded .res File Contains SADI Records          3 Report
PLAT178_ALERT_4_G The CIF-Embedded .res File Contains SIMU Records          2 Report
PLAT187_ALERT_4_G The CIF-Embedded .res File Contains RIGU Records          1 Report
PLAT242_ALERT_2_G Low    'MainMol' Ueq as Compared to Neighbors of         C1 Check
```

Author Response: Likely due to disorder.

```
PLAT242_ALERT_2_G Low    'MainMol' Ueq as Compared to Neighbors of        C21 Check
```

Author Response: Likely due to disorder.

```
PLAT302_ALERT_4_G Anion/Solvent/Minor-Residue Disorder (Resd    2)       100% Note
PLAT302_ALERT_4_G Anion/Solvent/Minor-Residue Disorder (Resd    3)       100% Note
PLAT302_ALERT_4_G Anion/Solvent/Minor-Residue Disorder (Resd    4)       100% Note
PLAT302_ALERT_4_G Anion/Solvent/Minor-Residue Disorder (Resd    5)       100% Note
PLAT304_ALERT_4_G Non-Integer Number of Atoms in ..... (Resd    2)       3.99 Check
PLAT304_ALERT_4_G Non-Integer Number of Atoms in ..... (Resd    3)       4.06 Check
PLAT304_ALERT_4_G Non-Integer Number of Atoms in ..... (Resd    4)       3.01 Check
PLAT304_ALERT_4_G Non-Integer Number of Atoms in ..... (Resd    5)       2.94 Check
PLAT432_ALERT_2_G Short Inter X...Y Contact  F5_2     ..C42      .       2.93 Ang.
                                        1-x,-1/2+y,3/2-z  =      3_646 Check
PLAT432_ALERT_2_G Short Inter X...Y Contact  F6_2     ..C46      .       2.78 Ang.
                                                   x,y,z  =      1_555 Check
PLAT860_ALERT_3_G Number of Least-Squares Restraints ............       1360 Note
PLAT910_ALERT_3_G Missing # of FCF Reflection(s) Below Theta(Min).          3 Note
                1  1  0,   1  0  1,   0  1  1,
PLAT912_ALERT_4_G Missing # of FCF Reflections Above STh/L=  0.600          7 Note
PLAT933_ALERT_2_G Number of HKL-OMIT Records in Embedded .res File          6 Note
                0  0  6,   3  0 13,   0  2  4,   0  2  7,   1  0  3,   1  0  5,
PLAT969_ALERT_5_G The 'Henn et al.' R-Factor-gap value ..........       9.378 Note
              Predicted wR2: Based on SigI**2  1.56 or SHELX Weight 13.27
PLAT978_ALERT_2_G Number C-C Bonds with Positive Residual Density.          0 Info
```

---

0 **ALERT level A** = Most likely a serious problem - resolve or explain
0 **ALERT level B** = A potentially serious problem, consider carefully
18 **ALERT level C** = Check. Ensure it is not caused by an omission or oversight
26 **ALERT level G** = General information/check it is not something unexpected

0 ALERT type 1 CIF construction/syntax error, inconsistent or missing data

```
22 ALERT type 2 Indicator that the structure model may be wrong or deficient
 5 ALERT type 3 Indicator that the structure quality may be low
16 ALERT type 4 Improvement, methodology, query or suggestion
 1 ALERT type 5 Informative message, check
```

It is advisable to attempt to resolve as many as possible of the alerts in all categories. Often the minor alerts point to easily fixed oversights, errors and omissions in your CIF or refinement strategy, so attention to these fine details can be worthwhile. In order to resolve some of the more serious problems it may be necessary to carry out additional measurements or structure refinements. However, the purpose of your study may justify the reported deviations and the more serious of these should normally be commented upon in the discussion or experimental section of a paper or in the "special_details" fields of the CIF. checkCIF was carefully designed to identify outliers and unusual parameters, but every test has its limitations and alerts that are not important in a particular case may appear. Conversely, the absence of alerts does not guarantee there are no aspects of the results needing attention. It is up to the individual to critically assess their own results and, if necessary, seek expert advice.

Publication of your CIF in IUCr journals

A basic structural check has been run on your CIF. These basic checks will be run on all CIFs submitted for publication in IUCr journals (*Acta Crystallographica*, *Journal of Applied Crystallography*, *Journal of Synchrotron Radiation*); however, if you intend to submit to *Acta Crystallographica Section C* or *E* or *IUCrData*, you should make sure that full publication checks are run on the final version of your CIF prior to submission.

Publication of your CIF in other journals

Please refer to the *Notes for Authors* of the relevant journal for any special instructions relating to CIF submission.

PLATON version of 02/02/2025; check.def file version of 02/02/2025

Datablock JS22 - ellipsoid plot

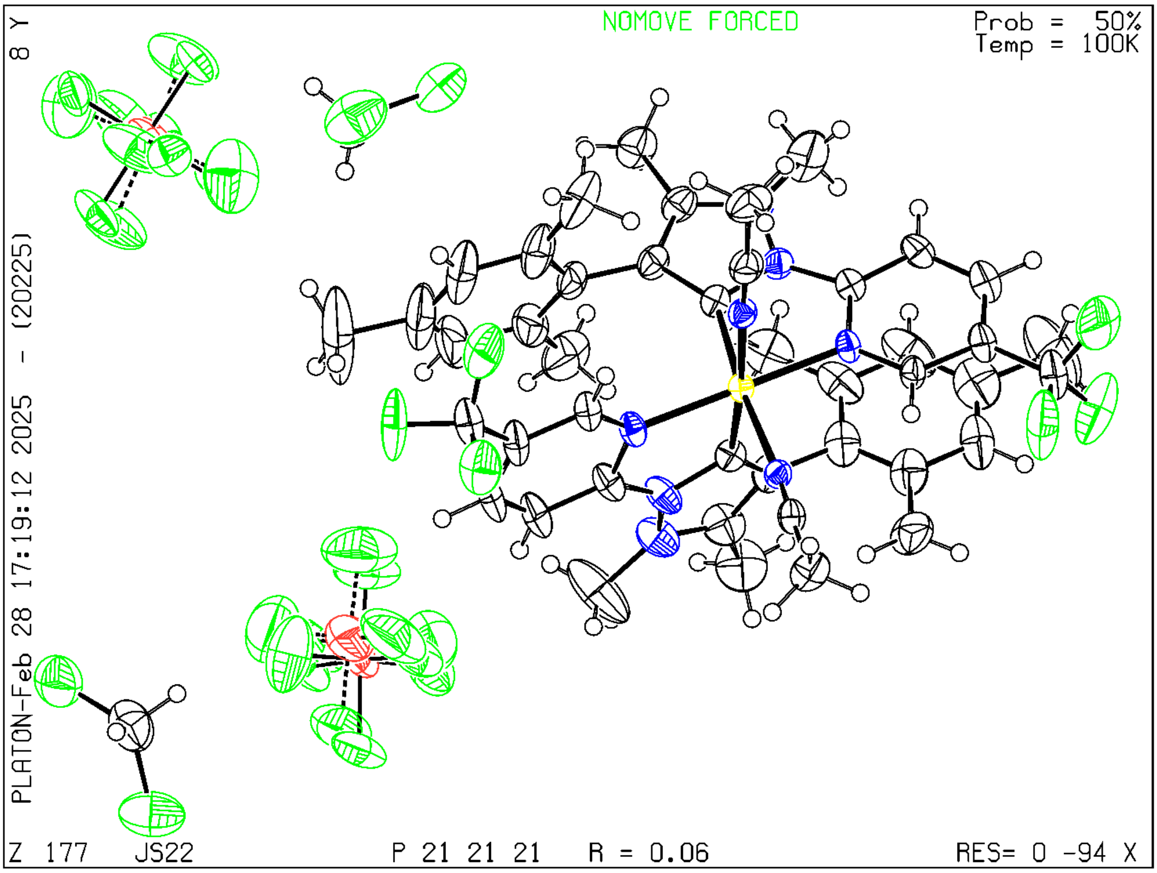

Supplement: Supplementary file 2 [file CHEM-32-e71426-s001.zip › rac-Ru2_checkcif_2.pdf]
