# Supplementary material for: Chiral Pyrazolinylidene Complexes for Asymmetric Catalysis
Source: Chemistry. 2026 Jul 12;32(39):e71426. doi: 10.1002/chem.71426 (PMC13715465; doi:10.1002/chem.71426)

checkCIF/PLATON report

Structure factors have been supplied for datablock(s) LiPl19

THIS REPORT IS FOR GUIDANCE ONLY. IF USED AS PART OF A REVIEW PROCEDURE FOR PUBLICATION, IT SHOULD NOT REPLACE THE EXPERTISE OF AN EXPERIENCED CRYSTALLOGRAPHIC REFEREE.

No syntax errors found.   CIF dictionary   Interpreting this report

Datablock: LiPl19

Bond precision:	C-C = 0.0134 A		Wavelength=0.71073
Cell:	a=12.239(4)	b=15.643(6)	c=18.438(6)
	alpha=90.221(9)	beta=99.147(8)	gamma=100.557(9)
Temperature:	100 K		

	Calculated	Reported
Volume	3424(2)	3424(2)
Space group	P -1	P -1
Hall group	-P 1	-P 1
Moiety formula	C58 H68 N8 Ru Si2, 2(F6 P), 2(C H2 Cl2)	C58 H68 N8 Ru Si2, 2(F6 P), 2(C H2 Cl2)
Sum formula	C60 H72 Cl4 F12 N8 P2 Ru Si2	C60 H72 Cl4 F12 N8 P2 Ru Si2
Mr	1494.25	1494.24
Dx,g cm-3	1.449	1.449
Z	2	2
Mu (mm-1)	0.543	0.543
F000	1532.0	1532.0
F000'	1532.09	
h,k,lmax	14,18,21	14,18,21
Nref	12176	11948
Tmin,Tmax	0.938,0.982	0.598,0.745
Tmin'	0.902	

```
Correction method= # Reported T Limits: Tmin=0.598 Tmax=0.745
AbsCorr = MULTI-SCAN

Data completeness= 0.981          Theta(max)= 25.074

R(reflections)= 0.0837( 10212)          wR2(reflections)= 0.2108( 11948)

S = 1.067                 Npar= 938
```

The following ALERTS were generated. Each ALERT has the format
test-name_ALERT_alert-type_alert-level.
Click on the hyperlinks for more details of the test.

Alert level C

PLAT029_ALERT_3_C _diffrn_measured_fraction_theta_full value Low . 0.976 Why?

Author Response: Low crystal quality and highly overlapping reflections difficult to resolve.

PLAT234_ALERT_4_C Large Hirshfeld Difference C57 --C58 . 0.18 Ang.

Author Response: Due to disorder and twinning. No other atom type is chemically plausible on those positions.

PLAT243_ALERT_4_C High 'Solvent' Ueq as Compared to Neighbors of C59 Check

Author Response: Due to disorder and twinning.

PLAT243_ALERT_4_C High 'Solvent' Ueq as Compared to Neighbors of C60 Check

Author Response: Due to disorder and twinning.

PLAT250_ALERT_2_C Large U3/U1 Ratio for <U(i,j)> Tensor(Resd 1) 2.3 Note

Author Response: Due to disorder and twinning.

PLAT250_ALERT_2_C Large U3/U1 Ratio for <U(i,j)> Tensor(Resd 2) 2.1 Note

Author Response: Due to disorder and twinning.

PLAT250_ALERT_2_C Large U3/U1 Ratio for <U(i,j)> Tensor(Resd 3) 2.2 Note

Author Response: Due to disorder and twinning.

```
PLAT250_ALERT_2_C Large U3/U1 Ratio for <U(i,j)> Tensor(Resd    4)        2.2 Note
```

Author Response: Due to disorder and twinning.

```
PLAT260_ALERT_2_C Large Average Ueq of Residue Including      P1_2      0.111 Check
```

Author Response: Due to disorder and twinning.

```
PLAT260_ALERT_2_C Large Average Ueq of Residue Including       Cl1      0.140 Check
```

Author Response: Due to disorder and twinning.

```
PLAT260_ALERT_2_C Large Average Ueq of Residue Including       Cl3      0.155 Check
```

Author Response: Due to disorder and twinning.

```
PLAT342_ALERT_3_C Low Bond Precision on  C-C Bonds ...............    0.01336 Ang.
```

Author Response: Due to disorder and twinning.

```
PLAT910_ALERT_3_C Missing FCF Reflection(s) Below Theta(Min)[Deg]=       2.14 Note
                1  0  0,  -1  1  0,   0  1  0,   0 -1  1,  -1  0  1,   0  0  1,
                0  1  1,
```

Author Response: A few reflections could not be collected using the selected measurement strategy and the instrument.

```
PLAT911_ALERT_3_C Missing FCF Refl Between Thmin & STh/L=    0.596        290 Report
               11  0  0, -13  1  0, -11  1  0, -13  2  0, -11  2  0, -13  3  0,
              -11  3  0, -13  4  0, -11  4  0, -11  5  0, -14  7  0,  10 10  0,
              -13 11  0, -12 13  0, -10 15  0,   1 18  0,   8-17  1,  13 -9  1,
               14 -7  1,  13 -6  1,  11 -5  1,  13 -5  1,  11 -4  1,  13 -4  1,
               11 -3  1,  13 -3  1,  11 -2  1,  13 -2  1,  11 -1  1,  13 -1  1,
                   (  260 More Missing: see the .ckf listing file)
```

Author Response: A few reflections could not be collected using the selected measurement strategy and the instrument.

Alert level G

```
PLAT002_ALERT_2_G Number of Distance or Angle Restraints on AtSite         26 Note
PLAT003_ALERT_2_G Number of Uiso or U(i,j)  Restrained non-H-Atoms         26 Report
PLAT083_ALERT_2_G SHELXL Second Parameter in WGHT  Unusually Large      18.57 Why ?
PLAT174_ALERT_4_G The CIF-Embedded .res File Contains FLAT Records          2 Report
PLAT175_ALERT_4_G The CIF-Embedded .res File Contains SAME Records          3 Report
PLAT176_ALERT_4_G The CIF-Embedded .res File Contains SADI Records          7 Report
```

```
PLAT178_ALERT_4_G The CIF-Embedded .res File Contains SIMU Records          4 Report
PLAT187_ALERT_4_G The CIF-Embedded .res File Contains RIGU Records          3 Report
PLAT230_ALERT_2_G Hirshfeld Test Diff for    N8       --C57      .        5.1 s.u.
PLAT244_ALERT_4_G Low    'Solvent' Ueq as Compared to Neighbors of         P1 Check
PLAT301_ALERT_3_G Main Residue  Disorder .............(Resd    1)          9% Note
PLAT302_ALERT_4_G Anion/Solvent/Minor-Residue Disorder (Resd    3)       100% Note
PLAT302_ALERT_4_G Anion/Solvent/Minor-Residue Disorder (Resd    4)       100% Note
PLAT304_ALERT_4_G Non-Integer Number of Atoms in ..... (Resd    3)       4.18 Check
PLAT304_ALERT_4_G Non-Integer Number of Atoms in ..... (Resd    4)       2.82 Check
PLAT432_ALERT_2_G Short Inter X...Y Contact  F3       ..C10_3    .       2.91 Ang.
                                                     x,y,z  =      1_555 Check
PLAT790_ALERT_4_G Centre of Gravity not Within Unit-Cell: Resd.  #          2 Note
              F6 P
PLAT790_ALERT_4_G Centre of Gravity not Within Unit-Cell: Resd.  #          5 Note
              C H2 Cl2
PLAT790_ALERT_4_G Centre of Gravity not Within Unit-Cell: Resd.  #          6 Note
              C H2 Cl2
PLAT860_ALERT_3_G Number of Least-Squares Restraints ............         682 Note
PLAT870_ALERT_4_G ALERTS Related to Twinning Effects Suppressed ..          ! Info
PLAT909_ALERT_3_G Percentage of I>2sig(I) Data at Theta(Max) Still        72% Note
PLAT931_ALERT_5_G CIFcalcFCF  Twin Law ( 0 1 0)        Est.d BASF       0.22 Check
PLAT933_ALERT_2_G Number of HKL-OMIT Records in Embedded .res File          2 Note
                0 -3  3,   1  0  3,
PLAT941_ALERT_3_G Average HKL Measurement Multiplicity ..........         1.0 Low
PLAT969_ALERT_5_G The 'Henn et al.' R-Factor-gap value ..........       4.035 Note
              Predicted wR2: Based on SigI**2  5.22 or SHELX Weight 19.76
```

```
   0 ALERT level A = Most likely a serious problem - resolve or explain
   0 ALERT level B = A potentially serious problem, consider carefully
  14 ALERT level C = Check. Ensure it is not caused by an omission or oversight
  26 ALERT level G = General information/check it is not something unexpected

   0 ALERT type 1 CIF construction/syntax error, inconsistent or missing data
  13 ALERT type 2 Indicator that the structure model may be wrong or deficient
   8 ALERT type 3 Indicator that the structure quality may be low
  17 ALERT type 4 Improvement, methodology, query or suggestion
   2 ALERT type 5 Informative message, check
```

It is advisable to attempt to resolve as many as possible of the alerts in all categories. Often the minor alerts point to easily fixed oversights, errors and omissions in your CIF or refinement strategy, so attention to these fine details can be worthwhile. It is up to the individual to critically assess their own results and, if necessary, seek expert advice.

PLATON version of 26/09/2025; check.def file version of 20/09/2025

---

duplicate check

No duplication found

Datablock LiPl19 - ellipsoid plot

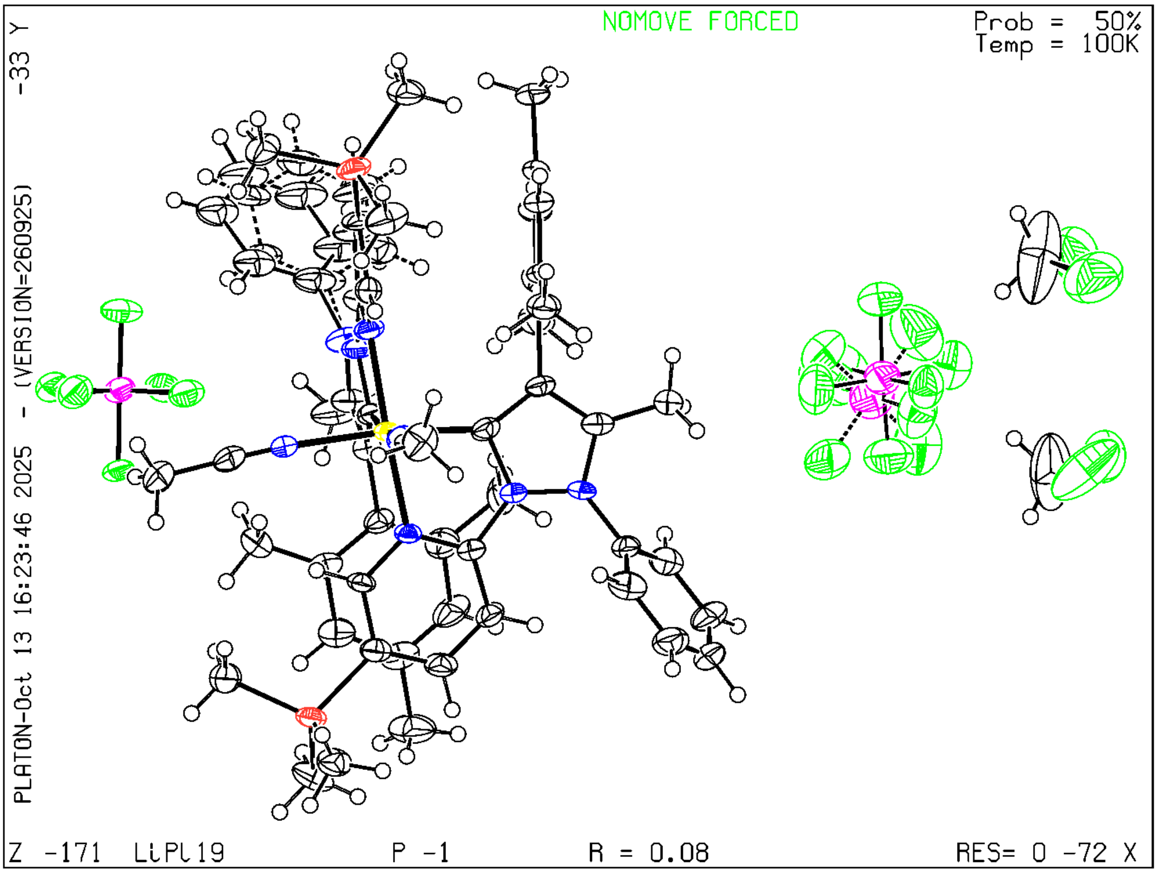

Supplement: Supplementary file 2 [file CHEM-32-e71426-s001.zip › rac-Ru4_checkcif_2.pdf]
